# Faecal immunochemical tests for patients with symptoms suggestive of colorectal cancer: An updated systematic review and multiple‐threshold meta‐analysis of diagnostic test accuracy studies

**DOI:** 10.1111/codi.17255

**Published:** 2024-12-17

**Authors:** Sue Harnan, Jean Hamilton, Emma Simpson, Mark Clowes, Aline Navega Biz, Sophie Whyte, Shijie Ren, Katy Cooper, Muti Abulafi, Alex Ball, Sally Benton, Richard Booth, Rachel Carten, Stephanie Edgar, Willie Hamilton, Matthew Kurien, Louise Merriman, Kevin Monahan, Laura Heathcote, Hayley E. Jones, Matt Stevenson

**Affiliations:** ^1^ SCHARR, University of Sheffield Sheffield UK; ^2^ Croydon Health Services NHS Trust London UK; ^3^ Sheffield Teaching Hospitals NHS Foundation Trust Sheffield UK; ^4^ Berkshire and Surrey Pathology Services Royal Surrey NHS Foundation Trust Guildford UK; ^5^ Buckinghamshire Healthcare NHS Trust Buckinghamshire UK; ^6^ GP Lead for FIT, South Yorkshire CCG Yorkshire UK; ^7^ University of Exeter Medical School Exeter UK; ^8^ St Mark's: National Bowel Hospital and Imperial College London UK; ^9^ Population Health Sciences University of Bristol Bristol UK

**Keywords:** adenomas, colorectal cancer, diagnostic test accuracy, faecal immunochemical test, inflammatory bowel disease, primary care, systematic review

## Abstract

**Aim:**

Extending faecal immunochemical tests for haemoglobin (FIT) to all primary care patients with symptoms suggestive of colorectal cancer (CRC) could identify people who are likely to benefit from colonoscopy and facilitate earlier treatment. The aim of this work was to investigate the diagnostic accuracy of FIT across different analysers at different thresholds, as a single test or in duplicate (dual FIT).

**Method:**

This systematic review and meta‐analysis searched 10 sources (December 2022). Diagnostic accuracy studies of HM‐JACKarc, OC‐Sensor, FOB Gold, QuikRead go, NS‐Prime and four Immunodiagnostik (IDK) tests in primary care patients were included. Risk of bias was assessed (QUADAS‐2). Statistical syntheses produced summary estimates of sensitivity and specificity at any chosen threshold for CRC, inflammatory bowel disease and advanced adenomas separately. Sensitivity analyses investigated reference standard and population type (high, low or all‐risk). Subgroup analyses investigated patient characteristics (e.g. anaemia, age, sex, ethnicity).

**Results:**

Thirty‐seven studies were included. At a threshold of 10 μg/g, pooled results for sensitivity and specificity (95% credible intervals) for CRC, respectively, were: HM‐JACKarc (*n* = 16 studies) 89.5% (84.6%–93.4%) and 82.8% (75.2%–89.6%); OC‐Sensor (*n* = 11 studies) 89.8% (85.9%–93.3%) and 77.6% (64.3%–88.6%); FOB Gold (*n* = 3 studies), 87.0% (67.3%–98.3%) and 88.4% (81.7%–94.2%). There were limited or no data on the other tests, dual FIT and relating to patient characteristics.

**Conclusion:**

Test sensitivity at a threshold of 10 μg/g highlights a requirement for adequate safeguards in test‐negative patients with ongoing symptoms. Further research is needed into the impact of patient characteristics and dual FIT.

**What does this paper add to the literature?**

This systematic review of faecal immunochemical tests informed the NICE NG12 guideline update in 2023. We synthesized accuracy across thresholds in a single analysis. Accuracy at 10 μg/g in primary care patients with NG12 signs/symptoms indicates far fewer referrals with few missed cancers. Thus, safety netting is required.

## INTRODUCTION

Early diagnosis and treatment of colorectal cancer (CRC) in people presenting to primary care with symptoms can improve survival and cure rates. However, identifying those at highest risk of CRC is essential to reduce unnecessary referrals to secondary care. There is evidence that quantitative faecal immunochemical tests (FITs) provide better discrimination of CRC risk in patients than symptoms alone and could result in fewer referrals of people without CRC.

FITs are designed to detect occult haemoglobin in stool samples using antibodies specific to human haemoglobin. In England, National Guideline 12 (NG12) recommended the use of FIT to guide referral in patients with low‐risk CRC symptoms in primary care, while recommending that referral to secondary care should be considered in those with high/medium‐risk CRC symptoms (see Data S1 for referral criteria). In 2022, the Association of Coloproctology of Great Britain and Ireland (ACPGBI) and the British Society of Gastroenterology (BSG) published guidance recommending the use of FIT in all patients with signs or symptoms of suspected CRC (i.e. both low risk and medium/high risk) [1]. This guidance was based on a systematic review and evidence synthesis [2] of the available evidence and expert opinion, and was agreed by consensus. The ACPGBI/BSG review also found little evidence relating to the accuracy of FIT in patients with specific characteristics that may affect performance, such as age, gender and ethnicity.

In late 2022, the National Institute of Health and Care Excellence (NICE) commissioned a Health Technology Assessment of FIT for patients with signs or symptoms of CRC, to include a systematic review and economic model [3]. A significant quantity of additional evidence had been published since the ACPGBI/BSG review, despite the short timeframe between the searches (less than a year). Here, we report the systematic review and evidence syntheses of diagnostic test accuracy studies that informed NICE's decision to recommend FIT for all patients with symptoms of CRC, using a threshold of 10 μg/g.

## METHOD

### Aims and objectives

This systematic review aimed to identify and synthesize evidence on the diagnostic test accuracy of FITs for CRC in patients with signs and symptoms of CRC. A number of additional questions were also addressed. (a) What is the diagnostic accuracy of dual FIT (defined as the preplanned use of the test in duplicate from different bowel movements to guide referral, and distinct from repeat FIT, a repeat test used to follow‐up continuing or worsening symptoms after a referral decision had been made, often done as part of ‘safety netting’). (b) Are alternative thresholds needed according to age, sex or ethnicity, in people with anaemia, people taking medications or with conditions which increase the risk of gastrointestinal bleeding, and in people with blood disorders (e.g. beta thalassaemia) that could affect the performance of the test. (c) Were estimates of accuracy affected by the reference standard used and the population (low risk, medium/high risk or any risk symptoms), as defined by NG12. (d) Within studies that report data for CRC, what is the diagnostic accuracy of FIT for inflammatory bowel disease (IBD) and advanced adenoma (AA)? The systematic review was conducted in accordance with Cochrane methodological guidelines [4, 5] and is reported in accordance with the PRISMA statement [6, 7]. The protocol was registered prospectively with PROSPERO (CRD42022383580).

### Search strategy

Searches were conducted in December 2022 and were based upon the ACPGBI/BSG review. Medline, Embase and the Cochrane database were searched, and supplemented with searches of trial registers, Health Technology Assessment sources and PROSPERO. Reference lists in included articles and relevant systematic reviews were checked for additional studies. Clinical experts were consulted to ensure that no relevant studies had been missed. The search terms and additional information are reported in Data S2. Retrieved records were downloaded to and deduplicated in Endnote.

### Study selection

Inclusion criteria are presented in Table 1. Titles and abstracts were considered for inclusion against the criteria by one reviewer. At the start, a minimum 10% sample was checked by a second reviewer in increments of 100 until 100% agreement that relevant abstracts were not missed was achieved. Full texts were considered for inclusion by one reviewer and decisions checked by a second; discrepancies were resolved through discussion.

**TABLE 1:** Study selection criteria.

Element	Inclusion criteria
Population	People presenting to or referred from primary care with symptoms or signs indicating a risk of CRC^a^
Intervention/index test	HM‐JACKarc FOB Gold OC‐Sensor NS Prime IDK TurbiFIT IDK haemoglobin ELISA IDK Hb/Hp complex ELISA QuikRead go iFOBT Tests could be used once (single FIT) or in duplicate (dual FIT)^b^
Comparator/reference standard	RCTs: usual care, for example referral to secondary care Test accuracy: Full colonic imaging via colonoscopy or CTC Index‐test‐dependent differential reference standard (e.g. imaging for FIT‐positive patients and records follow‐up for FIT‐negative patients)
Outcomes/target conditions^c^	CRC, AA and IBD diagnoses^d^ TP, FP, FN, FP or data that allow their calculation (e.g. sensitivity, specificity, total *N* and prevalence), for CRC, AA and IBD AEs Mortality Stage of detected cancers Test uptake and failure rates Time to diagnosis or colonoscopy
Study design	RCT studies Diagnostic test accuracy studies or comparative diagnostic test accuracy studies that avoided a case–control design English language, or non‐English if sufficient data could be extracted

Abbreviations: AA, advanced adenoma; AE, adverse event; CRC, colorectal cancer; CTC, computed tomography colonography; ELISA, enzyme‐linked immunosorbent assay; FIT, faecal immunochemical test; FN, false negative; FP, false positive; Hb/Hp, haemoglobin‐haptoglobin; IBD, inflammatory bowel disease; iFOBT, human haemoglobin immunochemical based faecal occult blood test; *N*, number; RCT, randomized controlled trial; TN, true negative; TP, true positive.

^a^ Signs and symptoms of CRC were defined as those described in NG12 (see Data S1); however, studies were not excluded if criteria were wider or narrower but were excluded if any were not from primary care;

^b^ Dual FIT is defined as the preplanned use of the test in duplicate from different bowel movements to guide referral, whereas repeat FIT is a repeat test used to follow‐up continuing or worsening symptoms after the referral decision.

^c^ A full list of the outcomes sought is provided in Data S3.

^d^ Since the main aim of this review was to synthesize data on CRC, data on AA and IBD were only included from studies that also reported CRC data.

### Data extraction and risk of bias assessment

A data extraction form was developed based on the ACPGBI/BSG group's form (see Data S4). QUADAS‐2 [8] was used to assess the quality of diagnostic test accuracy studies. Scoring criteria are provided in Data S4. Data extraction and quality assessment were performed by one reviewer and checked by a second. Disagreements were resolved through discussion and authors were contacted to clarify ambiguities.

### Synthesis

Study and patient characteristics were tabulated and are summarized narratively. For tests where data were available from more than one study, pooled estimates of diagnostic parameters were estimated using the modelling approach described in Jones et al. [9], separately for each test and target condition. The model accommodates estimates of sensitivity and specificity at more than one explicit diagnostic threshold per study. Pooled estimates are produced at all possible thresholds, even where data for a given threshold were unreported by an empirical study included in the review. Random effects meta‐analysis was used to account for heterogeneity between studies. Analyses were conducted in R [10] using the JAGS Markov chain Monte Carlo (MCMC) sampler and the RJAGS interface package. Full details of the implementation are provided in Data S5. Subgroup analyses were conducted to investigate the impact on test accuracy of the population recruited to the study, the reference standard used and specific patient characteristics that may affect the performance of FIT.

### Patient and public involvement

This review did not include any patient or public involvement.

## RESULTS

**FIGURE 1:**
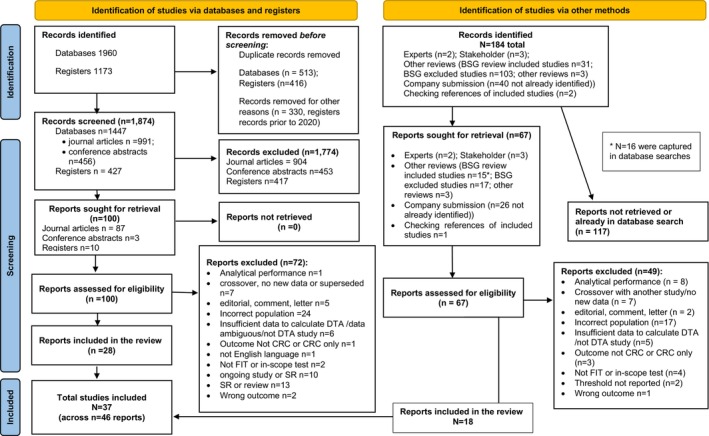
PRISMA flow diagram of the study selection process.

A total of 2058 records were retrieved (see Figure 1). A total of 1891 articles were excluded based on their title or abstract. The full texts of 167 were retrieved and assessed and 121 were excluded (see Data S6). Thirty‐seven studies were included, reported across 46 publications.

None of the included studies were RCTs. For diagnostic accuracy studies of single FIT, there were 16 HM‐JACKarc studies (19 publications) [11, 12, 13, 14, 15, 16, 17, 18, 19, 20, 21, 22, 23, 24, 25, 26, 27, 28, 29], 12 OC‐Sensor studies (12 publications) [11, 30, 31, 32, 33, 34, 35, 36, 37, 38, 39, 40], three FOB Gold studies [32, 41, 42] and one study each for QuikRead go [43], NS‐Prime, [32] IDK Hb [44] and IDK Hb/Hp. [44] No data were identified for IDK TurbiFIT or IDK Hb + Hb/Hp. Some of these studies included data on patient characteristics that may affect FIT, and an additional five studies (six publications) [45, 46, 47, 48, 49, 50] and one additional publication from a study already included [51] reported data on patient characteristic subgroups only. Four studies [18, 29, 52, 53] reported data on dual FIT, of which two [52, 53] were not already included in the single FIT analysis. Twenty reported data on other outcomes (test failures, repeats, uptake and time to diagnosis), two of which (three publications) [54, 55, 56] were not already included in the review.

Since diagnostic accuracy may be affected by the population recruited, studies were categorized as follows: population 1, patients recruited across high/medium‐risk and low‐risk criteria; population 2, high/medium‐risk patients; population 3, low‐risk patients; population 4, unclear recruitment criteria or did not fit into the other categories. This latter category included studies that recruited patients who had been referred to secondary care in England, since this population would be a mixture of high/medium‐risk patients as well as some low‐risk patients referred based on a positive FIT. It also included studies from other countries with different referral criteria, or where criteria were unclear or inappropriately limited in other ways.

### Risk of bias assessment

#### Risk of bias

Tables summarizing risk of bias assessment for all studies included in the review are provided in Data S5. In summary, no study scored as low risk for all items, and no item scored as low risk for all studies. The index test scored as low risk most often. Where patient selection scored as high risk, it was usually because a consecutive sample was not recruited and/or because inappropriate exclusions were made, such as excluding people based on not having had a colonoscopy. Where the reference standard was at unclear or high risk of bias this was usually because not all patients received a colonoscopy or CTC, or due to it being unclear if the reference standard was interpreted blind to the index test. Patient flow scored poorly, often due to a lack of clarity about the interval between the index test and the reference standard, patients receiving different reference standards depending on their FIT result or other factors and patients being missing from the study.

#### Applicability

There were concerns about the representativeness of the patients recruited to the studies compared with ‘all those presenting to primary care’ in nearly all studies due to either exclusion of some relevant patients (i.e. study population types 2, 3 and 4) or to a lack of clarity about who was included compared with the target population.

### Single FIT studies

Study characteristics are summarized in Table 2 and additional detail is provided in Data S7.

**TABLE 2:** Summary of studies included in the review, subgrouped by test and population type.

Number of studies, author and year of study	Location of study	Reference standards	Sample size range	Range of % with CRC	Range of median age (years)	Thresholds reported	Subgroup data?
**HM‐JACKarc**							
Population type 1 studies (recruited across medium/high‐ and low‐risk criteria)							
*n* = 5 [13, 18, 20, 21, 22, 23] D'Souza 2020 [13] Gerrard 2023 [18] Johnstone 2022 [20] MacDonald 2022 [21] Mowat 2021 [23] and 2019 [22]	England (*n* = 1) [13] Scotland (*n* = 4) [18, 20, 21, 22, 23]	Imaging (*n* = 2) [13, 18] Records follow‐up (*n* = 3) [20, 21, 22, 23]	298 [13] to 5381 [22, 23]	1.29% [20] to 4.03% [13]	59 [20] to 65 [18, 22]	2 (*n* = 2) 7 (*n* = 1) 10 (*n* = 5) 20 (*n* = 1) 50 (*n* = 1) 100 (*n* = 1) 150 (*n* = 2) 200 (*n* = 1) 250 (*n* = 1) 300 (*n* = 1) 350 (*n* = 1) 400 (*n* = 2)	Anaemia (*n* = 2) [18, 20]
Population type 2 studies (medium/high risk)							
*n* = 4 [12, 13, 14, 16, 29] D'Souza 2020 [13] and 2021 [12, 14] Farrugia 2020 [16] Turvill 2018 [29]	England (*n* = 4) [12, 13, 14, 16, 29]	Imaging (*n* = 4) [12, 13, 14, 16, 29]	160 [13] to 7194 [14]	3.57% [12] to 6.36% [16]	66 [12] to 69 [16, 29]	2 (*n* = 2) 10 (*n* = 3) 12 (*n* = 12) 150 (*n* = 1)	None
Population type 3 (low risk)							
*n* = 2 [13, 25, 28] D'Souza 2020 [13] Withrow 2022 [28] (overlaps with Nicholson 2020 [25])	England (*n* = 2) [13, 25, 28]	Imaging [13] Records follow‐up [25, 28]	138 [13] and 166 [28]	0.84% [28] and 1.45% [13]	NR [13] and 61 [25, 28]	2 (*n* = 2), 10 (*n* = 2)	Anaemia (*n* = 1) [25, 28] Sex (*n* = 1) [25, 28]
Population type 4 (unclear/unrepresentative of all presenting to primary care)							
*n* = 8 [11, 15, 17, 19, 24, 25, 26, 27] Chapman 2021 [11] Elbeltagi 2022 [15] Faux 2022 [17] Godber 2016 [19] Nicholson (2018) [24] and 2020 [25] (overlaps with Withrow 2022 [28]) Tang 2022 [26] Turvill 2021 [27]	England (*n* = 6) [11, 15, 17, 24, 25, 27, 28] Wales (*n* = 1) [26] Scotland (*n* = 1) [19]	Imaging (*n* = 5) [15, 17, 19, 26, 27] Records follow‐up (*n* = 2) [24, 25] Referral to secondary care (*n* = 1) [11]	175 [17] to 9896 [25]	1.06% [25, 28] to 5.24% [15]	60 [25, 28] to 72 [15]	2 (*n* = 1) 4 (*n* = 1) 7 (*n* = 2) 10 (*n* = 6) 20 (*n* = 2) 22.6 (*n* = 1) 50 (*n* = 2) 100 (*n* = 1) 120 (*n* = 1) 150 (*n* = 2) 29 thresholds between 6 and 401 at varying intervals (*n* = 1)	Sex (*n* = 1) [25] Anaemia (*n* = 1) [26]
**OC‐Sensor**							
Population type 1 studies (recruited across medium/high‐ and low‐risk criteria)							
*n* = 3 [33, 34, 35] Crooks 2023 [34] Cama 2022 [33] Georgiou Delisle 2022 [35]	England (*n* = 3) [33, 34, 35]	Records follow‐up (*n* = 3) [33, 34, 35]	4187 [35] to 37216 [34]	1.38% [34] to 1.46% [35]	61 [33] to 65 [35] years (NR in one study)	4 (*n* = 3) 10 (*n* = 3) 20 (*n* = 1) 40 (*n* = 1) 100 (*n* = 2) 150 (*n* = 1)	None
Population type 2 studies (medium/high risk)							
*n* = 1 Benton 2022 [32]	England	Imaging	233	3.00%	NR	1, 10, 100	None
Population type 3 (low risk)							
*n* = 1 Ball 2022 [31] (additional data by personal communication)	England	Imaging	2892	0.6%	NR	10, 20, 50, 80, 100, 120, 150	Men; women
Population type 4 (unclear/unrepresentative of all presenting to primary care)							
*n* = 7 [11, 30, 36, 37, 38, 39, 40] Archer 2022 [30] Chapman 2021 [11] Juul 2018 [36] Laszlo 2021 [37] Maclean 2021 [38] Mowat 2016 [39] Pin Vieito 2021 [40]	England (*n* = 3) [11, 30, 38] Scotland (*n* = 1) [39] UK (*n* = 1) [37] Denmark (*n* = 1) [36] Spain (*n* = 1) [40]	Imaging (*n* = 4) [11, 30, 37, 39] Records follow‐up (*n* = 3) [36, 38, 40]	166 [30] to 4543 [40]	1.56% [36] to 6.62% [30]	64 [39] to 71.1 [11] NR in 5 studies	4 (*n* = 3) 6 (*n* = 1) 10 (*n* = 7) 20 (*n* = 2) 50 (*n* = 1) 60 (*n* = 1) 80 (*n* = 1) 100 (*n* = 3) 120 (*n* = 1) 150 (*n* = 2) 200 (*n* = 1)	Unexplained anaemia [36]
**FOB Gold**							
Population type 2 studies (medium/high risk)							
*n* = 1 Benton 2022 [32]	England	Colonoscopy	233	3.00%	NR	2 (*n* = 1) 10 (*n* = 1) 100 (*n* = 1)	None
Population type 4 (unclear/unrepresentative of all presenting to primary care)							
*n* = 2 [41, 42] MacLean 2022 [42] Jordaan 2023 [41]	England (*n* = 2) [41, 42]	Imaging (*n* = 1) [42] Records follow‐up (*n* = 1) [41]	553 [42] to 3349 [41]	0.90% [41] to 2.53% [42]	NR	10 (*n* = 2) 100 (*n* = 1) 150 (*n* = 1)	None
**QuikRead go**							
Population type 2 studies (medium/high risk)							
*n* = 1 MacLean 2021 [43]	England	Colonoscopy	553	2.53%	NR	10 (*n* = 1) 100 (*n* = 1) 150 (*n* = 1)	None
**NS‐Prime**							
Population type 2 studies (medium/high risk)							
*n* = 1 [32] Benton 2022 [32]	England	Colonoscopy	233	3.00%	NR	3 (*n* = 1) 10 (*n* = 1) 100 (*n* = 1)	
**IDK Hb ELISA and IDK Hb/Hp complex**							
Population type 4 (unclear/unrepresentative of all presenting to primary care)							
*n* = 1 Sieg 1999 [44]	Germany	Colonoscopy	621	3.70%	59	2 (*n* = 1)	None

Abbreviation: NR, not reported.

The majority of studies were from the UK, with one each from Denmark [36], Spain [40] and Germany [44]. There were studies in each of the population categories for HM‐JACKarc and OC‐Sensor, but not for FOB Gold, QuikRead go, NS‐Prime and the IDK tests. Across all tests, the proportion of patients with CRC roughly followed the expectation that population 3 studies (low risk) would have lower CRC rates (range 0.6% [31] to 1.45% [13]) than population 2 studies (medium/high risk, range 2.53% [43] to 6.36% [16]), while population 1 studies (low and medium/high risk criteria) ranged from 1.29% to 4.03%. Population 4 studies had the widest range (0.90% [41] to 6.62% [30]), reflecting the heterogeneous recruitment criteria.

Three studies conducted a comparison of two or more tests: Chapman et al. [11] reported on OC‐Sensor DIANA and HM JACKarc, Benton et al. [32] compared HM‐JACKarc, OC‐Sensor PLEDIA, FOB Gold Wide/SENTiFIT 270 and NS‐Prime, and MacLean et al. [42] compared FOB Gold Wide and QuikRead go. An analysis of comparative test accuracy was not performed due to the small number of studies and data points and high clinical heterogeneity between studies. Further details are provided in Data S8.

All sensitivity and specificity data entering the analyses are provided in Data S9.

### Accuracy of tests in diagnosis of CRC

#### Each test individually

**FIGURE 2:**
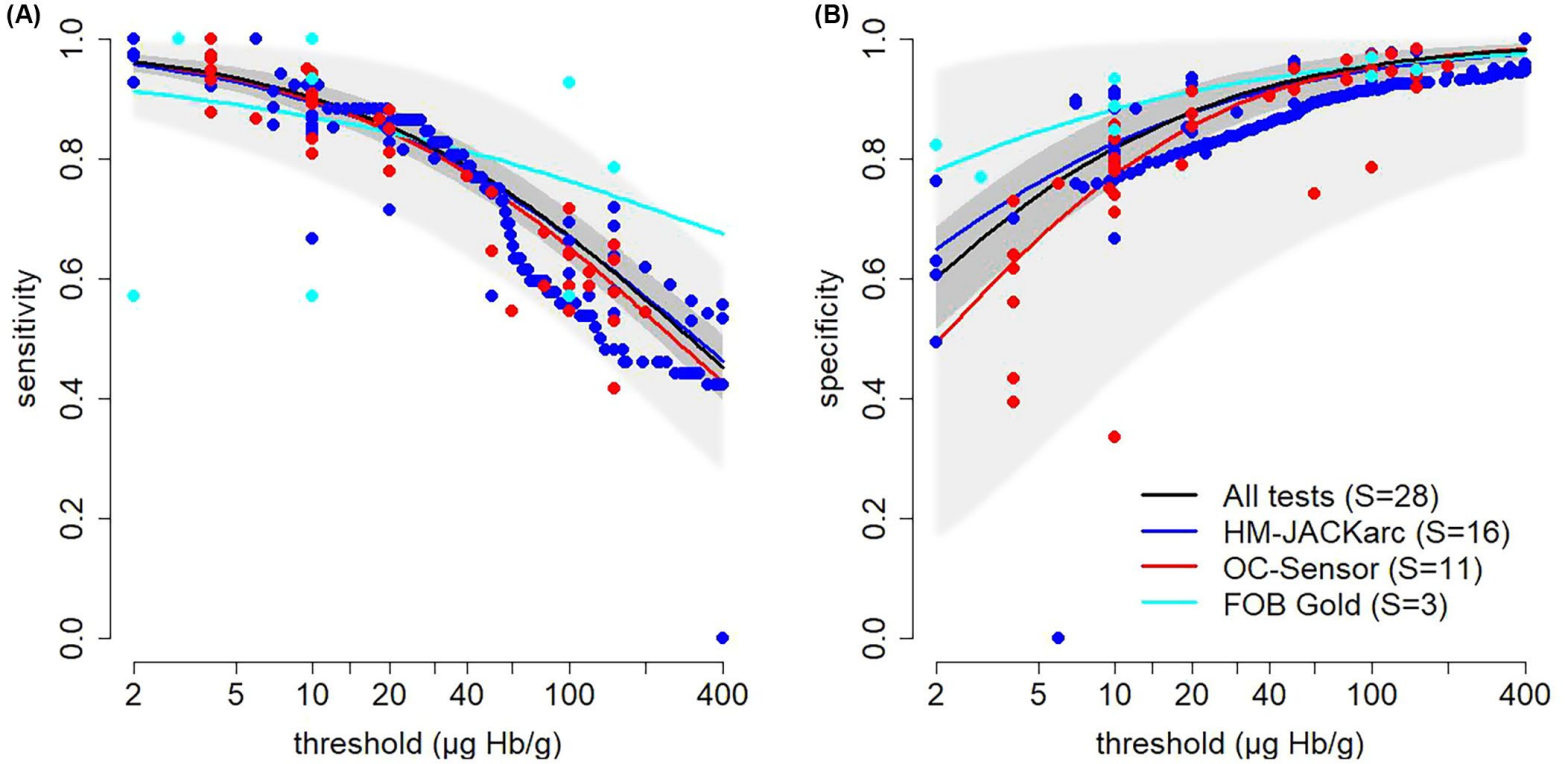
Sensitivity and specificity for all tests. The 95% credible intervals and predictive intervals for summary sensitivity and specificity of analysis including all tests are shown by the dark and light grey regions. Individual points are study specific data. Note that the number of studies in the ‘all tests’ category does not equal the sum of all the studies since some studies were excluded to avoid double counting patients and some studies reported data for more than one test.

Statistical synthesis was only possible for HM‐JACKarc, OC‐Sensor and FOB Gold, and results are presented in Figure 2 and Table 3 (additional details in Data S10–, S12) alongside data from individual studies for the remaining tests and an analysis pooling eligible data from all tests.

**TABLE 3:** Summary sensitivity and specificity at selected thresholds for detecting CRC.

Threshold (μg/g)	HM‐JACKarc (*S* = 16)^a^		OC‐Sensor (*S* = 11)^a^		FOB Gold (*S* = 3)^a^		All tests (*S* = 28)^a^		QuikRead go (*S* = 1)^b^		NS‐prime (*S* = 1)^b^		IDK Hb (*S* = 1)^b^		IDK Hb/Hp (*S* = 1)^b^	
	Sens (95% CrI)	Spec (95% CrI)	Sens (95% CrI)	Spec (95% CrI)	Sens (95% CrI)	Spec (95% CrI)	Sens (95% CrI)	Spec (95% CrI)	Sens (95% CI)	Spec (95% CI)	Sens (95% CI)	Spec (95% CI)	Sens (95% CI)	Spec (95% CI)	Sens (95% CI)	Spec (95% CI)
2	**95.9 (92.7, 97.9)**	**65.1 (55.6, 74.8)**	NR	NR	**91.4 (71.6, 99.6)**	**78.1 (70, 86)**	**96.4 (94.7, 97.7)**	**60.3 (51.6, 68.8)**	NR	NR	NR	NR	**87 (84.4, 89.6)** ^b^	**88.1 (85.6, 90.6)** ^b^	**82.6 (79.6, 85.6)** ^**b**^	**80.8 (77.7%, 83.9)** ^b^
2.5	95.3 (91.8, 97.5)	68 (58.8, 77.3)	NR	NR	90.9 (71.1, 99.5)	79.9 (71.9, 87.5)	95.8 (94, 97.2)	63.8 (55.5, 72.1)	NR	NR	NR	NR	NR	NR	NR	NR
3	94.7 (91.1, 97.2)	70.3 (61.3, 79.3)	NR	NR	90.5 (70.6, 99.4)	81.2 (73.4, 88.6)	95.3 (93.4, 96.9)	66.7 (58.6, 74.7)	NR	NR	**85.70 (48.7, 97.4)** ^b^	**31.90 (26.1, 38.2)** ^b^	NR	NR	NR	NR
4	93.8 (89.8, 96.5)	73.7 (65.1, 82.2)	**94.2 (91.2, 96.7)**	**62.7 (47.4, 77.2)**	89.8 (69.8, 99.2)	83.2 (75.6, 90.2)	94.4 (92.3, 96.2)	70.9 (63.2, 78.4)	NR	NR	NR	NR	NR	NR	NR	NR
7	**91.4 (86.8, 94.8)**	**79.6 (71.7, 87.1)**	**91.8 (88.2, 94.9)**	**72.3 (58.1, 84.8)**	**88.2 (68.4, 98.7)**	**86.5 (79.5, 92.8)**	**92.1 (89.6, 94.3)**	**78.1 (71.3, 84.6)**	NR	NR	NR	NR	NR	NR	NR	NR
10	**89.5 (84.6, 93.4)**	**82.8 (75.2, 89.6)**	**89.8 (85.9, 93.3)**	**77.6 (64.3, 88.6)**	**87 (67.3, 98.3)**	**88.4 (81.7, 94.2)**	**90.2 (87.4, 92.7)**	**82 (75.7, 87.8)**	**92.90 (68.5–98.7)** ^b^	**70.10 (66.1–73.8)** ^b^	**71.40 (35.9, 91.8)** ^b^	**83.60 (78.2, 87.9)** ^b^	**NR**	**NR**	**NR**	**NR**
20	**84.7 (79.1, 89.6)**	**87.9 (81.1, 93.4)**	**84.7 (80.3, 89)**	**85.6 (74.5, 93.6)**	**84.5 (65.1, 97.1)**	**91.3 (85.4, 96.2)**	**85.4 (82.2, 88.5)**	**88 (82.7, 92.5)**	NR	NR	NR	NR	NR	NR	NR	NR
50	75.8 (69.4, 82)	92.6 (87, 96.5)	75 (70.2, 80)	92.5 (84.3, 97.3)	80.3 (61.3, 94.7)	94.2 (89.3, 97.8)	76.3 (72.5, 80.1)	93.2 (89.3, 96.2)	NR	NR	NR	NR	NR	NR	NR	NR
100	67 (60, 74.2)	94.9 (90.3, 97.8)	65.3 (60.2, 70.7)	95.5 (89.4, 98.6)	76.4 (57.2, 92.5)	95.7 (91.6, 98.6)	67.2 (62.8, 71.5)	95.7 (92.7, 97.7)	71.40 (45.4–88.3)^b^	94.60 (92.4–96.2)^b^	57.10 (25.1, 84.2)^b^	97.30 (94.3–98.8)^b^	NR	NR	NR	NR
120	64.5 (57.2, 71.9)	95.4 (91, 98.1)	62.5 (57.2, 68)	96.1 (90.4, 98.9)	75.3 (55.8, 91.9)	96.1 (92.1, 98.8)	64.5 (60, 68.9)	96.2 (93.4, 98)	NR	NR	NR	NR	NR	NR	NR	NR
150	61.3 (53.7, 68.9)	96 (91.9, 98.4)	58.9 (53.4, 64.7)	96.7 (91.6, 99.1)	73.9 (53.8, 91.2)	96.4 (92.6, 98.9)	61.1 (56.4, 65.7)	96.7 (94.1, 98.4)	57.10 (32.6–78.6)^b^	95.90 (93.9–97.3)^b^	NR	NR	NR	NR	NR	NR
200	57 (48.9, 64.9)	96.6 (92.8, 98.7)	54.2 (48.4, 60.2)	97.3 (92.9, 99.3)	NR	NR	56.5 (51.6, 61.4)	97.3 (95, 98.7)	NR	NR	NR	NR	NR	NR	NR	NR
400	46.3 (37.4, 54.9)	97.7 (94.7, 99.2)	NR	NR	NR	NR	45.2 (39.7, 50.6)	98.3 (96.6, 99.2)	NR	NR	NR	NR	NR	NR	NR	NR

*Note*: Thresholds in bold are those with the highest clinical relevance, based on feedback from clinical advisors to the project and to NICE. Columns in grey are from the statistical synthesis (see also footnotes a and b). The 95% confidence interval for a single study should not be directly compared with the 95% credible intervals (CrIs) from the meta‐analysis since estimates of error for these studies appear comparatively narrower than those from the synthesis of multiple studies due to being derived from one study only. The number of patients included in each study was: QuikRead go (Type 2 study), *n* = 553; NS‐Prime (Type 2 study), *n* = 233; IDK tests (Type 4 study), *n* = 621.

Abbreviations: CI, confidence interval; CrI, credible interval; Sens, sensitivity; spec, specificity; *S*, number of studies.

^a^ Summary estimates from the meta‐analysis model. Note that the number of studies in the ‘all tests’ category does not equal the sum of all the studies since some studies were excluded to avoid double counting patients and some studies reported data for more than one test.

^b^ Individual study estimates.

For HM‐JACKarc there were *n* = 44 299 patients from 16 studies and 151 pairs of sensitivity and specificity, with thresholds ranging from 2 to 400 μg/g. OC‐Sensor PLEDIA, OC‐Sensor iO and OC‐Sensor DIANA tests were considered interchangeable. There were *n* = 58 749 patients from 11 studies and 44 pairs of sensitivity and specificity, with thresholds ranging from 4 to 200 μg/g (note that data were reported at 1 μg/g [32], but this study was excluded from the synthesis to avoid double counting of patients). For FOB Gold there were *n* = 902 patients from three studies and eight pairs of sensitivity and specificity, with a threshold ranging from 2 to 150 μg/g.

The results of the syntheses at selected thresholds are reported in Table 3. The pooled sensitivity [95% credible interval (CrI)] and specificity (95% CrI) at the lowest thresholds were as follows: 96% (93%–98%) and 65% (56%–75%) at 2 μg/g for HM‐JACKarc; 94% (91%–97%) and 63% (47%–77%) at 4 μg/g for OC‐Sensor; and 91% (72%–100%) and 78% (70%–86%) at 2 μg/g for FOB Gold.

At a threshold of 10 μg/g, the pooled results for sensitivity and specificity, respectively, were as follows: HM‐JACKarc, 89% (85%–93%) and 83% (75%–90%); OC‐Sensor, 90% (86%–93%) and 78% (64%–89%); FOB Gold, 87% (67%–98%) and 88% (82%–94%).

Subgroup analyses by population type were feasible for HM‐JACKarc and OC‐Sensor. Based on the overlap of 95% CrI, test accuracy did not differ according to population type for either test (see Data S10 and S11).

#### Sensitivity analyses

Two sensitivity analyses were conducted using data from all tests together to investigate the impact of population type (population types 1, 2 and 3) and reference standard (≥90% imaging versus <90% imaging) on test accuracy. Results were similar to the main analysis in both cases. More details can be found in Data S13 and S14.

### Patient characteristics subgroup analyses

Subgroup analyses were conducted for: anaemia, age, sex, medications that might cause gastrointestinal bleeding, ethnicity and people with blood disorders (other than anaemia) that might affect FIT. Details are provided in Data S15. Across patient characteristic subgroups [anaemia (11 studies) [14, 18, 20, 26, 27, 28, 36, 45, 46, 48, 50], age (three studies) [27, 28, 51], sex (three studies) [25, 27, 31] and people taking medications which may affect FIT results (three studies) [27, 47, 49] evidence was limited and sometimes inconsistent. No studies were identified on ethnicity or for people with other blood disorders.

### Accuracy of tests in advanced adenoma and inflammatory bowel disease

Nine studies (10 publications) [12, 13, 18, 21, 22, 23, 36, 39, 43, 44] reported test accuracy for AAs and IBD, providing 15 pairs of sensitivity and specificity at thresholds between 2 and 150 μg/g for each outcome. Analyses are provided in Data S16 and summarized in Tables 4 and 5. Sensitivity and specificity were overall lower than for CRC. There was a large amount of heterogeneity between studies, as illustrated by the wide 95% CrIs.

**TABLE 4:** Summary sensitivity and specificity at selected thresholds for detecting advanced adenoma.

Threshold (μg/g)	All tests (*S* = 9)		HM‐JACKarc (*S* = 6)		OC‐Sensor (*S* = 2)	
	Sensitivity (CrI)	Specificity (CrI)	Sensitivity (CrI)	Specificity (CrI)	Sensitivity (CrI)	Specificity (CrI)
2	80.4 (55.8, 98.3)	51.6 (31.6, 71.1)	59.1 (50, 92)	55.9 (35.1, 80.6)	NR	NR
2.5	78 (55.2, 97.4)	55.4 (36.1, 74.4)	58.4 (50, 90.5)	58.1 (38.4, 82.9)	NR	NR
3	75.9 (54.7, 96.4)	58.4 (39.6, 77.1)	57.7 (50, 89.3)	59.9 (41, 84.6)	NR	NR
4	72.2 (53.7, 93.8)	63.1 (45.6, 81)	56.7 (49.8, 86.8)	62.8 (45.1, 87.3)	93.9 (51.5, 100)	46.8 (9.5, 90.3)
7	63.9 (51.4, 84.6)	71.7 (55.3, 87.7)	54.7 (48.2, 81.2)	68.5 (50.8, 91.7)	84.6 (27.8, 100)	70.7 (27.2, 96.7)
10	57.7 (48.6, 76.7)	76.5 (60.3, 90.9)	53.2 (45.9, 77.6)	71.9 (52, 93.8)	73.2 (10.1, 99.9)	82.2 (41.6, 98.7)
20	47.4 (26.1, 64.4)	84.2 (68.1, 95.3)	50.9 (37.3, 71.6)	77.9 (53.7, 96.5)	NR	NR
50	34.1 (5.6, 53.2)	91.1 (75.7, 98.2)	49.8 (24.3, 65.7)	84.4 (55.4, 98.5)	NR	NR
100	25 (1.4, 48.9)	94.4 (80.2, 99.2)	48.7 (16, 61.9)	88.2 (56.3, 99.2)	NR	NR
120	22.8 (1, 48.3)	95 (81.3, 99.3)	48.3 (14.2, 61.1)	89.1 (56.6, 99.4)	NR	NR
150	20.4 (0.6, 47.5)	95.7 (82.5, 99.5)	47.8 (12.3, 60.1)	90.1 (56.9, 99.5)	NR	NR

Abbreviations: CrI, credible interval; NR, not reported; *S*, number of studies.

**TABLE 5:** Summary sensitivity and specificity at selected thresholds for detecting inflammatory bowel disease.

Threshold (μg/g)	All tests (*S* = 9)		HM‐JACKarc (*S* = 6)		OC‐Sensor (*S* = 2)	
	Sensitivity (CrI)	Specificity (CrI)	Sensitivity (CrI)	Specificity (CrI)	Sensitivity (CrI)	Specificity (CrI)
2	85.7 (70, 96.7)	53.8 (33.1, 75.5)	86.8 (68.4, 98.5)	57 (36.4, 78.7)	NR	NR
2.5	84.3 (68.5, 96)	57.2 (37.2, 78.4)	85.8 (67.3, 98.1)	59.4 (39.2, 80.6)	NR	NR
3	83.1 (67.2, 95.3)	60 (40.5, 80.6)	84.9 (66.4, 97.8)	61.3 (41.3, 82.1)	NR	NR
4	81 (65.1, 94)	64.2 (45.8, 84)	83.4 (64.7, 97.1)	64.3 (44.8, 84.4)	67 (24.7, 97.9)	46.4 (7.4, 92)
7	76.3 (60.4, 90.7)	72 (54.7, 89.4)	80.1 (61.2, 95.3)	69.9 (50.8, 88.1)	59.8 (16.4, 95.5)	70.3 (22.3, 97.5)
10	72.9 (57.1, 88.2)	76.4 (59.2, 92.1)	77.6 (58.6, 94)	73.3 (53.3, 90.2)	55.1 (12.2, 93.1)	81.9 (35.3, 99)
20	65.3 (49.2, 82.9)	83.6 (66.3, 95.8)	72.3 (52.2, 91.1)	79.2 (57.3, 93.4)	NR	NR
50	54.4 (33.6, 75.5)	90.3 (73.7, 98.3)	64.9 (35.1, 86.9)	85.4 (61.7, 96.2)	NR	NR
100	46.3 (21.7, 69.7)	93.6 (78, 99.2)	59.2 (21.3, 83.4)	89.1 (64.3, 97.5)	NR	NR
120	44.2 (19, 68.1)	94.3 (79, 99.3)	57.7 (18.3, 82.4)	89.9 (64.9, 97.8)	NR	NR
150	41.7 (15.9, 66.1)	95 (80.2, 99.5)	55.7 (15.2, 81.2)	90.8 (65.8, 98.1)	NR	NR

Abbreviations: CrI, credible interval; NR, not reported; *S*, number of studies.

### Dual FIT

In three studies [29, 52, 53] patients had already been referred to secondary care (likely similar to high‐risk patients). In one additional study [18] referrals had also been made, but based on criteria that most closely match high‐ and low‐risk criteria. A synthesis was not performed due to insufficient data points relating to any one test. Data are summarized in Data S17 and Table 6. When interpreting a positive test as either test being positive, sensitivity was higher and specificity lower compared with using single FIT.

**TABLE 6:** Dual FIT studies sensitivity and specificity.

Author, year	No. with CRC (or IBD or AA)/no. in analysis	Either positive			Both positive			Single FIT			
		Thr	Sensitivity (95% CI)	Specificity (95% CI)	Thr	Sensitivity (95% CI)	Specificity (95% CI)	*N* with CRC (or IBD or AA)/*N* in analysis	Thr	Sensitivity (95% CI)	Specificity (95% CI)
**HM‐JACKarc**											
Gerrard 2023 [18]	CRC: 88/2637 (3.34%)	10	96.60 (90.4–99.3)	71.2 (69.4–73.0)	NR	NR	NR	CRC: 135/3426 (3.94%)	10	93.3 (87.7–96.9)	78.0 (76.6–79.4)
	IBD: 33/2637 (1.39%)		90.90 (75.7–98.1)	69.70 (67.9–71.5)	NR	NR	NR	55/3426 (1.61%)		90.90 (80.0–97.0)	76.30 (74.8–77.7)
	AA: 97/2637 (3.68%)		68.00 (57.8–77.1)	70.40 (68.5–72.1)	NR	NR	NR	136/3426 (4.00%)		54.4 (45.6–63.0)	76.40 (45.7–63.0)
Turvill 2018 [29]	27/476 (5.67%)	43	87.50 (NR)	90.70 (NR)	2	91.70	85.20	CRC: 27/505 (5.35%)	12	84.60	88.50
**OC‐Sensor**											
Hunt 2022 [52]	317/28 622 (1.11%)	10	98 (95.5–98.9)	66.20 (65.7–66.7)	10	92 (87.9–94.1)	81.60 (81.1–82.0)	NA	NA	NR	NR
**QuikRead go**											
Tsapournas 2020 [53]	13/242 (5.37%)	10	100.00 (NR)	71.40 (65.5–77.3)		NR	NR	CRC: 13/242 (5.37%)	10	92.30 (77.8–100)	77.30 (71.9–82.7)
		15	92.30 (77.8–100)	76.80 (71.3–82.3)		NR	NR		15	92.30 (77.8–100)	81.70 (76.7–86.7)
		20	92.30 (77.8–100)	81.70 (76.6–86.8)		NR	NR		20	84.60 (65.0–100)	86.50 (82.1–90.9)

Abbreviations: AA, advanced adenoma; CRC, colorectal cancer; IBD, inflammatory bowel disease; NA, not applicable; NR, not reported; Thr, threshold in μg/g.

### Other outcomes

Other outcomes included test uptake and repeat tests, ‘time to’ outcomes (e.g. time to diagnosis) and other outcomes such as stage at diagnosis. These data, from 20 publications, [13, 18, 20, 21, 22, 23, 26, 29, 31, 33, 35, 36, 39, 41, 46, 52, 53, 54, 55, 56] are provided in Data S18.

#### Test failure rates

Test failure rates were generally between 2% and 5%, [20, 21, 22, 23, 33, 36, 46] for single FIT. Problems reported included buffer loss, labelling errors, incorrect containers, no date of collection, volume errors and laboratory accidents [35, 41].

#### Uptake

Where single FIT was used as part of the diagnostic pathway, 3631/38 920 (9.3%) [57] FIT requests were not returned. All dual FIT studies took place in secondary care, with nonreturn rates from 5% [52] to 31% [18].

#### Repeat tests

Five studies (seven references) [22, 23, 31, 41, 54, 55, 56] reported repeat FIT rates, which ranged from 0.7% [31] to 17% [56].

#### ‘Time to’ outcomes and other outcomes

These data were only sought from studies that were included in the diagnostic test accuracy review. The data are summarized in Data S18.

## DISCUSSION

We have synthesized data in this extensive systematic review of the accuracy of FIT in patients with signs or symptoms of CRC. These analyses were considered by NICE in 2023 when forming its recommendation to extend the use of FIT in patients with signs and symptoms of CRC in primary care from low‐risk patients only to include medium/high‐risk patients.

Although there were directly relevant data for seven tests, most of the evidence pertained to HM‐JACKarc (*n* = 44 299 patients from 16 studies) and OC‐Sensor (*n* = 58 749 patients from 11 studies), with a limited evidence base for the other analysers.

Statistical syntheses for HM‐JACKarc, OC‐Sensor and FOB Gold produced similar summary estimates of sensitivity across the three tests at a threshold of 10 μg/g (pooled estimates range 87%–90%). These values indicate that for every 1000 patients completing a FIT, assuming a prevalence of 3% and a sensitivity of about 90%, approximately 27 cancers would be detected, and three patients with CRC would be undiagnosed at this threshold (see Table 7). At the lowest thresholds reported (2 μg/g for HM‐JACKarc and FOB Gold; 4 μg/g for OC‐Sensor, where false negatives are minimized) sensitivities ranged from 91% to 96%, resulting in between one and three CRC cases per 1000 patients tested below this threshold. If a lower prevalence of 1% is assumed, the number of missed diagnoses reduces to one per 1000 tests at a threshold of 10 μg/g and 0.4–1 at the respective lowest threshold reported. Therefore, to minimize the delay to diagnosis in missed cases, adequate safety netting strategies are needed (e.g. advice to return, repeat FIT, clinician review) [52], especially in those with new or persistent symptoms.

**TABLE 7:** Numbers of positive FITs, negative FITs, CRCs detected, CRCs missed and positive FITs needed to detect one CRC at selected thresholds for a cohort of 1000 patients.

Threshold (μg/g)	FIT+	FIT−	CRC detected	CRC missed	FIT+ to detect one CRC	FIT+	FIT−	CRC detected	CRC missed	FIT+ to detect one CRC
CRC prevalence	1%	1%	1%	1%	1%	3%	3%	3%	3%	3%
**2**	**355**	**645**	**10**	**0.4**	**37**	**367**	**633**	**29**	**1**	**13**
7	211	789	9	1	23	225	775	27	3	8
**10**	**179**	**821**	**9**	**1**	**20**	**194**	**806**	**27**	**3**	**7**
20	128	872	8	2	15	143	857	25	5	6
50	81	919	8	2	11	95	905	23	7	4
100	57	943	7	3	9	70	930	20	10	3
120	52	948	6	4	8	64	936	19	11	3
150	46	954	6	4	7	57	943	18	12	3

*Note*: Thresholds in bold are discussed in the discussion text.

Abbreviations: CRC, colorectal cancer; FIT+, number with a positive FIT test; FIT−, number with a negative FIT test; FIT, faecal immunochemical test.

Specificity can be used to calculate the expected number of patients who will be referred to secondary care who do not have CRC. It is desirable to minimize such referrals since colonoscopy carries a risk to patients (e.g. bowel perforation) and has cost and capacity implications. The pooled estimates of specificity were more variable (range 78%–88%, with wider confidence intervals). Again, at a threshold of 10 μg/g and assuming a prevalence of 3%, for every 1000 tests between 113 and 217 patients would be referred who do not have CRC. Therefore, for every 5–10 positive FIT tests, one CRC would be diagnosed. However, if the lowest thresholds for each test were used in an effort to minimize missed diagnoses (pooled estimates range 63%–78%), between 212 and 339 patients would be referred who do not have CRC, and one CRC would be detected in every 10–14 positive FIT tests, indicating a higher burden on colonoscopy capacity, but still a risk of missing CRC. When assuming a CRC prevalence of 1%, the conversion rate of referrals to CRC diagnoses is lower. At a threshold of 10 μg/g, there would be 115 to 222 false positives, meaning one CRC is diagnosed per 14–26 positive FIT tests. At the lowest reported thresholds for each test, 217 to 369 false positives mean one CRC is diagnosed per 25–40 positive FIT tests. The reduction in missed diagnoses therefore comes at the cost of an increase in referrals of patients who do not have CRC, and this is particularly the case when prevalence of CRC is low.

Among those not diagnosed with CRC, some will have IBD or AA. These diagnoses, although not the primary purpose, are a significant benefit of investigating for potential CRC. Data relating to the diagnostic test accuracy of FIT for IBD and AA were limited (*n* = 9 studies; we did not aim to include studies on IBD and AA unless they reported test accuracy data for CRC as well). At a threshold of 10 μg/g, the pooled sensitivity for IBD was 73% and the specificity 76%. These values emphasize that if IBD is being considered FIT alone is insufficient—faecal calprotectin is the superior primary care test. For AA, the values were 57% and 76%, respectively. Therefore, FIT should be considered primarily as a triage tool for those with possible CRC who require further, more invasive, investigation, while accepting it may uncover some (but not all) alternative diagnoses such as AA and IBD, which may benefit from early detection.

### Comparison with existing work

The estimates of sensitivity are similar to those reported in the ACPGBI/BSG data synthesis for HM‐JACKarc and OC‐Sensor. For example, at a threshold of 10 μg/g, the ACPGBI/BSG review found a pooled sensitivity for HM‐JACKarc of 90.6% (95% CI 87.6%–92.9%) compared with 89.5% (95% CrI 84.6%–93.4%) in our analysis. The estimate of specificity was a little different at 78.2% (95% CI 69.2%–85.2%) compared with 82.8% (95% CrI 75.2%–89.6%); however, in both cases the point estimates were within the bounds of the confidence/credible intervals for the other analysis. To investigate whether the analytical method (stratified bivariate model versus multiple thresholds model) affected the estimates we conducted a bivariate meta‐analysis for HM‐JACKarc at a threshold of 10 μg/g. This produced a similar pooled sensitivity of 89.2% (95% CrI 85.7%–92.0%). The specificity was 79.4% (95% CrI 75.0%–83.3%), which is within the confidence intervals reported by the multiple threshold analysis and may be due to additional studies that entered the analysis, and/or the methods of the multiple threshold model.

### Strengths and limitations

Our analysis was conducted to high standards, including comprehensive searches across databases complemented with nominations from companies, NICE specialist committee members and clinical experts. The analysis used an advanced statistical model to synthesize available data across thresholds. This has several advantages over performing separate bivariate meta‐analyses at selected thresholds, including making use of all the available data, increasing precision, ensuring consistency of pooled results and producing summary estimates at all thresholds of interest. We excluded test accuracy studies with a case–control design, which are thought to be at high risk of overestimation of accuracy. We did not restrict ourselves to studies that recruited according to low‐ or medium/high‐risk criteria, or to studies that had used an imaging reference standard for all patients. We investigated the impact of these factors on results, and while these analyses indicated no detectible effect, we caution that in the presence of multiple sources of heterogeneity it is difficult to isolate the effects of these factors. We believe the question of the impact of population remains only partially investigated, and further caution that the generalizability of the study results to populations outside of the scope, especially very low‐risk patients and patients less than 50 years of age, should not be assumed.

Our analysis also has other limitations. First, we did not compare the tests directly with each other due to insufficient studies directly comparing devices; all three comparative studies identified concluded that there were differences in tests. Further head‐to‐head studies are needed. Second, we did not investigate the timing of the reference standard and cannot draw conclusions about what the optimal follow‐up should be to avoid interval cancers affecting the false‐negative rate, nor for how long a FIT test can be considered ‘valid’. Thirdly, all studies were at some risk of bias.

We identified evidence gaps and uncertainties. There were insufficient and inconsistent data relating to patient characteristics (anaemia, age, sex, etc.) and, consistent with the ACPGBI/BSG guideline, no conclusions could be drawn on whether different thresholds should be used in these patients. The recently published COLOFIT study 58 helps resolve some of these issues as it tested these and other factors in a predictive algorithm and found most made little difference. The COLOFIT authors recommend further external validation of their algorithm before implementation. None of the studies on dual FIT were conducted in primary care patients before their referral decision was made, creating uncertainty about return rates in that context, and potentially about test accuracy, since three studies recruited largely high‐risk patients. Reporting of the inclusion of patients with symptoms that historically led to immediate referral (rectal or anal mass or anal ulceration) was often missing, and some studies excluded patients with rectal bleeding. This should be borne in mind when interpreting the evidence base.

### Impact on policy

The results of this analysis were used in a cost‐effectiveness analysis (to be reported in a subsequent publication) to assess both cost‐effectiveness and clinical effectiveness. This model also estimated the likely impact on waiting times for colonoscopies and time to diagnosis. It formed part of the NICE committee's deliberations and led to a change in policy (Diagnostic Guidance 56, which has been incorporated into the current version of NG12), which aligned with the ACPGBI/BSG guideline to use the test at a threshold of 10 μg/g, albeit in patients with specific signs and symptoms suggestive of CRC.

## CONCLUSIONS

This meta‐analysis updated and concurred with the findings of the ACPGBI/BSG guidelines on the use of FIT in patients with signs and symptoms of CRC, using a multiple threshold meta‐analysis methodology which made use of more of the available evidence and reported test accuracy at any threshold required. The sensitivity of around 90% for the most frequently reported tests (HM‐JACKarc and OC‐Sensor) indicates that adequate safeguards are needed to identify missed diagnoses. More data are required on patient characteristics that may affect FIT results, the expansion of the test to younger age groups and lower risk criteria, comparative test accuracy and on the use of dual FIT.

## AUTHOR CONTRIBUTIONS

**Sue Harnan:** Conceptualization; data curation; methodology; project administration; investigation; writing – original draft; writing – review and editing; supervision. **Jean Hamilton:** Conceptualization; methodology; data curation; formal analysis; writing – original draft; writing – review and editing; supervision; visualization. **Emma Simpson:** Methodology; data curation; investigation; writing – original draft; writing – review and editing. **Mark Clowes:** Methodology; data curation; writing – original draft; writing – review and editing. **Aline Navega Biz:** Conceptualization; methodology; data curation; formal analysis; investigation; writing – original draft; writing – review and editing. **Sophie Whyte:** Conceptualization; methodology; writing – original draft; writing – review and editing; data curation; formal analysis; investigation; visualization; supervision. **Shijie Ren:** Methodology; data curation; formal analysis; writing – original draft; writing – review and editing. **Katy Cooper:** Conceptualization; writing – review and editing. **Muti Abulafi:** Investigation; resources; writing – review and editing. **Alex Ball:** Resources; writing – review and editing. **Sally Benton:** Resources; writing – review and editing. **Richard Booth:** Investigation; resources; writing – review and editing. **Rachel Carten:** Investigation; writing – review and editing; resources. **Stephanie Edgar:** Resources; writing – review and editing. **Willie Hamilton:** Resources; writing – review and editing. **Matthew Kurien:** Resources; writing – review and editing. **Louise Merriman:** Resources; writing – review and editing. **Kevin Monahan:** Investigation; resources; writing – review and editing. **Laura Heathcote:** Investigation; writing – original draft; writing – review and editing; visualization. **Hayley E. Jones:** Methodology; validation; writing – original draft; writing – review and editing. **Matt Stevenson:** Conceptualization; methodology; data curation; formal analysis; investigation; writing – original draft; writing – review and editing; visualization; supervision.

## FUNDING INFORMATION

This report was commissioned by the NIHR Evidence Synthesis Programme as project number NIHR135637.

## CONFLICT OF INTEREST STATEMENT

None of the authors have any financial conflicts of interest to declare. Muti Abulafi, Alex Ball, Sally Benton, Richard Booth, Rachel Carten, Willie Hamilton, Matt Kurien and Kevin Monahan were all authors on studies included in the review or on the review we updated.

## ETHICS STATEMENT

Ethics approval was not required for this work since it is entirely based on secondary data in the public domain.

## PATIENT CONSENT STATEMENT

No patients were involved in this research since it is entirely based on secondary data in the public domain.

## PREVIOUS COMMUNICATIONS

This paper is based on work that was submitted to the National Institute of Health and Care Excellence (NICE) and was included in the relevant committee papers on NICE's website. The work will also be the subject of a Health Technology Assessment monograph, and was presented as a poster at the Health Technology Assessment International (HTAi) meeting in Seville in June 2024.

## REGISTRATION

The protocol was registered prospectively with PROSPERO (CRD42022383580).

## Supporting information


Data S1.

Data S2.

Data S3.

Data S4.

Data S5.

Data S6.

Data S7.

Data S8.

Data S9.

Data S10.

Data S11.

Data S12.

Data S13.

Data S14.

Data S15.

Data S16.

Data S17.

Data S18.

## Data Availability

We have made much of the relevant data available as online supplements to this work, and further data may be made available upon request to the corresponding author.
