# Supplementary material for: Faecal immunochemical tests for patients with symptoms suggestive of colorectal cancer: An updated systematic review and multiple‐threshold meta‐analysis of diagnostic test accuracy studies
Source: Colorectal Dis. 2024 Dec 17;27(1):e17255. doi: 10.1111/codi.17255 (PMC11683176; doi:10.1111/codi.17255)
Supplement: Supplementary file 1 — Data S1. [file CODI-27-0-s008.docx]

**National guideline 12 (NG12) high/medium-risk and diagnostic guideline 30 (DG30) low-risk patients**

National guideline 12 (NG12) describes the diagnostic pathway for patients presenting to primary care with symptoms suggestive of CRC^1^ (Figure 1). Up until mid 2023, this guideline stated that patients with the following symptoms (referred to as NG12 high/medium-risk patients in this assessment) should be referred to secondary care with an urgent 2 week wait (2WW) suspected CRC referral. NG12^1^ stated:

“Refer adults using a suspected cancer pathway referral (for an appointment within 2 weeks) for CRC if:

- they are aged 40 and over with unexplained weight loss and abdominal pain or
- they are aged 50 and over with unexplained rectal bleeding or
- they are aged 60 and over with:
  - iron‑deficiency anaemia or
  - changes in their bowel habit, or
- tests show occult blood in their faeces.

Consider a suspected cancer pathway referral (for an appointment within 2 weeks) for colorectal cancer in adults with a rectal or abdominal mass

Consider a suspected cancer pathway referral (for an appointment within 2 weeks) for colorectal cancer in adults aged under 50 with rectal bleeding and any of the following unexplained symptoms or findings:

- abdominal pain
- change in bowel habit
- weight loss
- iron deficiency anaemia.”

© NICE 2015 and 2021. Suspected cancer: recognition and referral. Previously available from the [NICE website](https://www.nice.org.uk/guidance/ng12). Subject to [Notice of rights](https://www.nice.org.uk/terms-and-conditions#notice-of-rights).

In July 2017, NG12^1^ was partially updated by Diagnostics guidance 30 (DG30).^2^ In this update, the guaiac faecal occult blood test (fOBT), which had been recommended for use in low-risk patients, was replaced with FIT. NG12 stated:

“Offer testing with quantitative faecal immunochemical tests (see the NICE diagnostics guidance on quantitative faecal immunochemical tests to guide referral for colorectal cancer in primary care) to assess for colorectal cancer in adults without rectal bleeding who:

- are aged 50 and over with unexplained:
- abdominal pain or
- weight loss, or
- are aged under 60 with:
- changes in their bowel habit or
- iron-deficiency anaemia, or
- are aged 60 and over and have anaemia even in the absence of iron deficiency.”

© NICE 2021. Suspected cancer: recognition and referral. Available from the [NICE website](https://www.nice.org.uk/guidance/ng12). Subject to [Notice of rights](https://www.nice.org.uk/terms-and-conditions#notice-of-rights).

1. NICE. Suspected cancer: recognition and referral. 2015.

2. NICE. Quantitative faecal immunochemical tests to guide referral for colorectal cancer in primary care. Diagnostics guidance [DG30]; 2017.
