# Supplementary material for: Faecal immunochemical tests for patients with symptoms suggestive of colorectal cancer: An updated systematic review and multiple‐threshold meta‐analysis of diagnostic test accuracy studies
Source: Colorectal Dis. 2024 Dec 17;27(1):e17255. doi: 10.1111/codi.17255 (PMC11683176; doi:10.1111/codi.17255)
Supplement: Supplementary file 2 — Data S2. [file CODI-27-0-s010.docx]

## Literature search strategies

### Clinical review search strategy

**Ovid MEDLINE(R) ALL <1946 to December 06, 2022>**

1 f?ecal immunochemical test.mp. 1259

2 f?ecal occult blood.mp. 4447

3 f?ecal h?emoglobin.mp. 269

4 ((immunochromatographic or immuno-chromatographic or immunochem$ or immuno-chem$ or immunohistochem$ or immuno-histochem$ or immunol$ or immunoassay or immuno* assay or immunoturbidimetric or immunosorbent or elisa) adj4 (f?ecal or f?eces or stool or stools or FIT)).mp. 3598

5 (iFOBT or qFIT).mp. 208

6 or/1-5 7290

7 F?ecal h?emoglobin.ti,ab,ot,hw. 256

8 H?emoccult.ti,ab,ot,hw. 728

9 FOBT.ti,ab,ot,hw. 1429

10 7 or 8 or 9 2335

11 (f?ecal or f?eces or stool or stools).ti,ab,ot,hw. 211912

12 occult blood/ or occult blood.ti,ab,ot,hw. 8924

13 (test$ or measur$ or screen$ or exam$).ti,ab,ot,hw. 10705030

14 11 and 12 and 13 6023

15 6 or 10 or 14 8736

16 exp colorectal neoplasms/ 231240

17 exp cecal neoplasms/ 6041

18 ((colorect$ or rectal$ or rectum$ or colon$ or sigma$ or sigmo$ or rectosigm$ or bowel$ or anal or anus) adj3 (cancer$ or neoplas$ or oncolog$ or malignan$ or tumo?r$ or carcinoma$ or adenocarcinoma$ or sarcoma$ or adenom$ or lesion$)).ti,ab,ot,hw. 324030

19 CRC.ti,ab,ot. 43421

20 ((cecum or cecal or caecum or caecal or il?eoc?ecal or il?eoc?ecum) adj3 (cancer$ or neoplas$ or oncolog$ or malignan$ or tumo?r$ or carcinoma$ or adenocarcinoma$ or sarcoma$ or adenom$ or lesion$)).ti,ab,ot. 2755

21 (large intestin$ adj3 (cancer$ or neoplas$ or oncolog$ or malignan$ or tumo?r$ or carcinoma$ or adenocarcinoma$ or sarcoma$ or adenom$ or lesion$)).ti,ab,ot. 1839

22 (lower intestin$ adj3 (cancer$ or neoplas$ or oncolog$ or malignan$ or tumo?r$ or carcinoma$ or adenocarcinoma$ or sarcoma$ or adenom$ or lesion$)).ti,ab,ot. 34

23 16 or 17 or 18 or 19 or 20 or 21 or 22 335879

24 15 and 23 5937

25 limit 24 to yr="2022 -Current" 426

26 (FOB gold$ or FOBgold$ or SENTiFIT).mp. 38

27 (JACK-arc$ or JACKarc$ or HM-JACK$ or HM JACK$ or HMJACK$).mp. 23

28 (OC Sensor$ or OC-Sensor$ or OCSensor$ or Ceres).mp. 371

29 (OC Pledia$ or OC-Pledia$ or OCPledia or OC-iO).mp. 0

30 (NS-Prime or NSPrime or NS-Plus).mp. 37

31 (POC FIT QRG or POCFITQRG).mp. 0

32 (immundiagnostik or IDK or turbifit or turbitube).mp. 125

33 quikread.mp. 19

34 or/25-33 994

35 limit 34 to yr="2016 -Current" 740

36 exp animals/ not (exp animals/ and humans/) 5072762

37 35 not 36 729

**Embase <1974 to 2022 Week 49> searched 6^th^ December 2022**

1 f?ecal immunochemical test.mp. 2253

2 f?ecal occult blood.mp. 6908

3 f?ecal h?emoglobin.mp. 436

4 ((immunochromatographic or immuno-chromatographic or immunochem$ or immuno-chem$ or immunohistochem$ or immuno-histochem$ or immunol$ or immunoassay or immuno* assay or immunoturbidimetric or immunosorbent or elisa) adj4 (f?ecal or f?eces or stool or stools or FIT)).mp. 6067

5 (iFOBT or qFIT).mp. 392

6 or/1-5 11749

7 F?ecal h?emoglobin.ti,ab,ot,hw. 422

8 H?emoccult.ti,ab,ot,hw. 987

9 FOBT.ti,ab,ot,hw. 2786

10 7 or 8 or 9 4077

11 (f?ecal or f?eces or stool or stools).ti,ab,ot,hw. 279057

12 occult blood/ or occult blood.ti,ab,ot,hw. 18102

13 (test$ or measur$ or screen$ or exam$).ti,ab,ot,hw. 13879472

14 11 and 12 and 13 10328

15 6 or 10 or 14 14766

16 exp colorectal cancer/ or colon cancer/ or rectum cancer/ 316446

17 exp cecum tumor/ 2471

18 ((colorect$ or rectal$ or rectum$ or colon$ or sigma$ or sigmo$ or rectosigm$ or bowel$ or anal or anus) adj3 (cancer$ or neoplas$ or oncolog$ or malignan$ or tumo?r$ or carcinoma$ or adenocarcinoma$ or sarcoma$ or adenom$ or lesion$)).ti,ab,ot,hw. 506633

19 CRC.ti,ab,ot. 70056

20 ((cecum or cecal or caecum or caecal or il?eoc?ecal or il?eoc?ecum) adj3 (cancer$ or neoplas$ or oncolog$ or malignan$ or tumo?r$ or carcinoma$ or adenocarcinoma$ or sarcoma$ or adenom$ or lesion$)).ti,ab,ot. 3559

21 (large intestin$ adj3 (cancer$ or neoplas$ or oncolog$ or malignan$ or tumo?r$ or carcinoma$ or adenocarcinoma$ or sarcoma$ or adenom$ or lesion$)).ti,ab,ot. 1807

22 (lower intestin$ adj3 (cancer$ or neoplas$ or oncolog$ or malignan$ or tumo?r$ or carcinoma$ or adenocarcinoma$ or sarcoma$ or adenom$ or lesion$)).ti,ab,ot. 44

23 16 or 17 or 18 or 19 or 20 or 21 or 22 515018

24 15 and 23 9914

25 limit 24 to yr="2022 -Current" 649

26 (FOB gold$ or FOBgold$ or SENTiFIT).mp. 107

27 (JACK-arc$ or JACKarc$ or HM-JACK$ or HM JACK$ or HMJACK$).mp. 73

28 (OC Sensor$ or OC-Sensor$ or OCSensor$ or Ceres).mp. 774

29 (OC Pledia$ or OC-Pledia$ or OCPledia or OC-iO).mp. 0

30 (NS-Prime or NSPrime or NS-Plus).mp. 75

31 (POC FIT QRG or POCFITQRG).mp. 0

32 (immundiagnostik or IDK or turbifit or turbitube).mp. 411

33 quikread.mp. 52

34 or/25-33 1997

35 limit 34 to yr="2016 -Current" 1406

36 limit 35 to embase 732

37 limit 35 to conference abstracts 500

38 limit 35 to "preprints (unpublished, non-peer reviewed)" 7

**The Cochrane Library (searched 12^th^ December 2022)**

Search Name: DAP50 final

Date Run: 12/12/2022 18:29:15

ID Search Hits

#1 (fecal immunochemical test* or faecal immunochemical test*):ti,ab,kw (Word variations have been searched) 497

#2 (fecal occult blood or faecal occult blood):ti,ab,kw (Word variations have been searched) 1087

#3 (fecal hemoglobin or faecal hemoglobin or fecal haemoglobin or faecal haemoglobin):ti,ab,kw (Word variations have been searched) 298

#4 ((immunochromatographic or immuno-chromatographic or immunochem* or immuno-chem* or immunohistochem* or immuno-histochem* or immunol* or immunoassay or immuno* assay or immunoturbidimetric or immunosorbent or elisa) near/4 (fecal or faecal or feces or faeces or stool or stools or FIT)):ti,ab,kw (Word variations have been searched) 1041

#5 (iFOBT or qFIT):ti,ab,kw (Word variations have been searched) 37

#6 (Hemoccult or haemoccult):ti,ab,kw (Word variations have been searched) 129

#7 (FOBT):ti,ab,kw (Word variations have been searched) 411

#8 ((fecal or feces or faecal or faeces or stool or stools)):ti,ab,kw AND (occult blood):ti,ab,kw AND (test* or measur* or screen* or exam*):ti,ab,kw (Word variations have been searched) 1153

#9 #1 or #2 or #3 or #4 or #5 or #6 or #7 or #8 2191

#10 MeSH descriptor: [Colorectal Neoplasms] explode all trees 9373

#11 MeSH descriptor: [Cecal Neoplasms] explode all trees 21

#12 ((colorect* or rectal* or rectum* or colon* or sigma* or sigmo* or rectosigm* or bowel* or anal or anus) near/3 (cancer* or neoplas* or oncolog* or malignan* or tumo* or carcinoma* or adenocarcinoma* or sarcoma* or adenom* or lesion*)):ti,ab,kw (Word variations have been searched) 26503

#13 (CRC):ti,ab,kw (Word variations have been searched) 5111

#14 ((cecum or cecal or caecum or caecal or ileocecal or ileocecum or ileocaecal or ileocaecum) near/3 (cancer* or neoplas* or oncolog* or malignan* or tumo* or carcinoma* or adenocarcinoma* or sarcoma* or adenom* or lesion*)):ti,ab,kw (Word variations have been searched) 246

#15 (large intestin* near/3 (cancer* or neoplas* or oncolog* or malignan* or tumo* or carcinoma* or adenocarcinoma* or sarcoma* or adenom* or lesion*)):ti,ab,kw (Word variations have been searched) 172

#16 (lower intestin* near/3 (cancer* or neoplas* or oncolog* or malignan* or tumo* or carcinoma* or adenocarcinoma* or sarcoma* or adenom* or lesion*)):ti,ab,kw (Word variations have been searched) 182

#17 #10 or #11 or #12 or #13 or #14 or #15 or #16 27348

#18 #9 and #17 with Cochrane Library publication date Between Jan 2022 and Dec 2022 103

#19 (FOB gold* or FOBgold* or SENTiFIT):ti,ab,kw OR (JACK-arc* or JACKarc* or HM-JACK* or HM JACK* or HMJACK*):ti,ab,kw OR (OC Sensor* or OC-Sensor* or OCSensor* or Ceres or OC Pledia* or OC-Pledia* or OCPledia or OC-iO):ti,ab,kw OR (POC FIT QRG or POCFITQRG or immundiagnostik or IDK or turbifit or turbitube or quikread):ti,ab,kw OR (NS-Prime or NSPrime or NS-Plus):ti,ab,kw (Word variations have been searched) 175

#20 #18 or #19 with Cochrane Library publication date Between Jan 2016 and Dec 2022 224

**INAHTA (searched 13/12/2022 )**

Single word strings:

Faecal / fecal / colorectal / colon / cecal = 0 results

**NIHR HTA programme website (searched 13/12/2022)**

Searched website - only found a few blogs including references to the RECEDE study

**PROSPERO searched 13/12/2022**

This website only allows for simple searches:

Colorectal AND faecal (records added to PROSPERO since 1/1/22) = 15 results

Colorectal AND fecal = 20 results (including the 15 above)

fecal immunochemical test = 6 results

faecal immunochemical test = 6 results

faecal occult blood = 7 results

fecal occult blood = 11 results

FOBT = 8 results

MeSH Colorectal Neoplasms/ = 41 results

Faecal and test* = 51 results

Fecal and test* and cancer = 27 results

**ClinicalTrials.gov searched 13/12/2022**

(CTgov automatically expands the search to include synonyms and alternate spellings)

Colorectal cancer AND faecal = 341 results since 1/1/2016

Colorectal cancer AND FIT = 159 results since 1/1/2016

Colon cancer AND faecal = 90 results since 1/1/2016

Colon cancer AND FIT = 32 results “”

Rectal cancer AND FIT = 11 results “”

Rectal cancer AND faecal = 92 results “”

**EU Trials Register (searched 13/12/2022)**

0 results

**WHO ICTRP (13/12/2022)**

**colon cancer OR colorectal cancer OR rectal cancer OR cecal cancer**

**AND
faecal OR fecal OR FIT or FOBT or iFOBT**

32 results
