# Supplementary material for: Faecal immunochemical tests for patients with symptoms suggestive of colorectal cancer: An updated systematic review and multiple‐threshold meta‐analysis of diagnostic test accuracy studies
Source: Colorectal Dis. 2024 Dec 17;27(1):e17255. doi: 10.1111/codi.17255 (PMC11683176; doi:10.1111/codi.17255)
Supplement: Supplementary file 3 — Data S3. [file CODI-27-0-s011.docx]

**Outcomes listed in the NICE scope**

The NICE scope^1^ stated that intermediate outcomes of interest may include:

- Diagnostic accuracy at different FIT thresholds for CRC, AA and IBD
- Risk of CRC (and IBD and AAs) in relevant subgroups according to FIT threshold
- Test failure rates
- Prognostic implications of false-negative results
- Uptake (completion) of FIT in primary care
- Number/proportion of people referred to secondary care
- Number/proportion of people followed up in primary care
- Duration of validity of negative test (implications for follow-up)
- Number/proportion of urgent (2WW suspected cancer) specialist appointments
- Number/proportion of urgent (2WW suspected cancer) colonoscopy/CTCs
- Number/proportion of non-urgent colonoscopy/CTCs
- Time to colonoscopy/CTC
- Time to diagnosis of CRC or other conditions
- Number/proportion of colonoscopy/CTCs that do not detect CRC
- Number/proportion of colonoscopy/CTCs that do not detect significant bowel pathology
- Number/proportion of people presenting to emergency departments with symptoms of CRC.

The NICE scope^1^ stated that clinical outcomes for consideration may include:

- Number of CRC diagnoses
- Number/proportion of CRC diagnoses from urgent referrals
- Stage of detected cancers
- Number/proportion of people identified with other bowel pathologies
- Number/proportion of people with AAs detected, or detected and treated
- Morbidity including adverse events associated with colonoscopy
- Mortality.

1. NICE. Quantitative faecal immunochemical tests to guide colorectal cancer pathway referral in primary care. Final scope. 2022.
