# Supplementary material for: Faecal immunochemical tests for patients with symptoms suggestive of colorectal cancer: An updated systematic review and multiple‐threshold meta‐analysis of diagnostic test accuracy studies
Source: Colorectal Dis. 2024 Dec 17;27(1):e17255. doi: 10.1111/codi.17255 (PMC11683176; doi:10.1111/codi.17255)
Supplement: Supplementary file 4 — Data S4. [file CODI-27-0-s015.docx]

**Data extraction fields**

The data extraction form used by ACPGBI/BSG guideline group was used as a basis for a *de novo* data extraction form, which was piloted on three studies and adapted as necessary. Several fields were added including fields relating to study population type (see Section 4.2.2.1) and study and patient characteristics (see Section 3.3.4). Study recruitment dates and locations were extracted to aid an assessment of “crossover” with other studies to avoid double counting of patients. Data relating to diagnostic test accuracy were extracted as the absolute numbers of true positives (TPs), true negatives (TNs), false positives (FPs) and false negatives (FNs) where available, or as sensitivity and specificity which was later transformed into TP, TNs etc as described below. The final list of fields extracted included: first author and date; year of recruitment; location (which country, county and hospital patients were recruited from); study name (where a name was provided for the study); inclusion and exclusion criteria (this was summarised if extremely lengthy); age limit (e.g., where a study only recruited patients over a certain age) population characteristics (age (mean), sex (% Male), medications that increase GI bleeding (% by type), blood disorders (% by type); Test-analyser combination (the name of the index test and the name of the device (analyser) it was processed on); index test methods (details of how samples were handled and processed); reference standard (% colonoscopy and details if not 100% colonoscopy); Number (N) recruited; N missing from analysis; N analysed; outcome (CRC, AA or IBD); N with outcome; threshold (the µg/g at which a test was interpreted as positive); diagnostic accuracy metrics; and any additional outcomes as described in online supplement 3.

**Conversion of sensitivity and specificity to TP, TN, FP, FN**

When the absolute number of diagnostic counts (TP, TN, FN, FP) were not reported by a study, but data for the total number of patients, the total number of positive cases, sensitivity and specificity were available, the count data were calculated using the equations below.

TP = sensitivity x number of positive cases

FN = (1 - sensitivity) x number of positive cases

FN = (1 - specificity) x (total number - number of positive cases)

TN = specificity x (total number - number of positive cases)

**Risk of bias assessment**

This supplement contains the criteria used for scoring (Table 1) and the summary risk of bias scores (Table 2)

Table 1 Risk of bias scoring criteria, based on QUADAS-2^1^

| **Domain 1: Patient selection** |
| --- |
| **Was a consecutive or random sample of patients enrolled?** *o Score yes if states consecutive or random o Score no if states another method of patient sampling/selection  o Score unclear if unclear* |
| **Was a case-control design avoided?**  *o Score yes if not case control  o Score no if case control  o Score unclear if unclear  (there should be no case control studies in the included studies, but please double check)* |
| **Did the study avoid inappropriate exclusions?** *Score yes if the study only excluded bypass symptom patients Score no if the study has made inappropriate exclusions e.g. on basis of having had a colonoscopy, taking certian medications, having blood disorders, or on the basis of eventually being diagnosed with IBD (list not exhaustive) Score unclear if it is unclear* |
| **Risk of bias summary score: Could the selection of patients have introduced bias?** Low/High/Unclear  THIS IS A SUMMARY SCORE BASED ON ANSWERS TO QUESTIONS 1 TO 3.  Score Low if all domains are Yes Score High if one or more domain is No Anything in between score Unclear |
| **Applicability summary score: Is there concern that the included patients and settings do not match the review question?** *Score low if the study selected all patients presenting to primary care with symptoms of CRC as listed in DG30 and NG12. If the study recruited a wider population, i.e. patients who do not meet these criteria, please state unclear risk (wider).  Score High if the study missed some of the primary care patients, e.g. if only those referred to colonoscopy were recruited (unless all primary care NG12/DG30 are referred to secondary care)* Low/High/Unclear |
| **Domain 2: Index test(s)** |
| **Were the index test results interpreted without knowledge of the results of the reference standard?** *o Score yes if index test was interpreted blind to the reference standard or the index test was clearly interpreted before the reference standard was known, e.g. FIT before colonoscopy o Score no if results of reference standard were already known e.g. FIT done after colonoscopy o Score unclear if unclear* |
| **If a threshold was used, was it pre-specified?** *o Score yes if pre-specified cut off values were used (validation study) e.g. one or a range of cut-offs reported such as 10, 20 , 50 , 100 ug/g and these were not chosen on the basis of having the highest accuarcy o Score no if cut-off values were fitted to the data (derivation study) e.g. cut-off with highest accuracy reported o Score unclear if unclear  NB if study reports both the highest precision cut-off, and several other "round number" cut-offs, score yes/no* |
| **Risk of bias summary score: Could the conduct or interpretation of the index test have introduced bias?** Low/High/Unclear **THIS IS A SUMMARY SCORE BASED ON ANSWERS TO QUESTIONS 1 TO 2.  Score Low if all domains are Yes Score High if one or more domain is No Anything in between score Unclear** |
| **Applicability summary score: Is there concern that the index test, its conduct, or interpretation differ from the review question?** *We may need to ask Sally to help us know what is normal practice, so for now we will just extract data*  Low/High/Unclear |
| **Domain 3: Reference Standard** |
| **Is the reference standard likely to correctly classify the target condition?** ***Please note limitations/test type against score***  *Score* ***Yes*** *if all patients received either colonoscopy or CT colonography (CTC) Score* ***No*** *if the reference standard was not full colonic imaging (see yes criteria) for all patients Score* ***Unclear*** *if its unclear* |
| **Were the reference standard results interpreted without knowledge of the results of the index test?** *In the case of tiered testing, this is likely not to be the case.*  o Score yes if the reference standard was interpreted blind to the index test or the reference standard was clearly interpreted before the index test was known.  o Score no if the results of the index test were known, e.g. where patients were referred on the basis of a FIT result.  o Score unclear if unclear |
| **Risk of bias summary score: Could the reference standard, its conduct, or its interpretation have introduced bias?**  Low/High/Unclear (THIS IS A SUMMARY SCORE BASED ON ANSWERS TO QUESTIONS 1 TO 2) |
| **Applicability summary score: Is there concern that the target condition as defined by the reference standard does not match the review question?** *Score* ***low risk*** *if the target condition is CRC Score* ***high risk*** *if the target is not just CRC Score* ***unclear*** *if the target condition is unclear* ***NB: all studies should score low risk  NB: we are not scoring for AA and IBD* Low/High/Unclear** The reference standard may be free of bias, but the target condition that it defines may differ from the target condition specified in the review question. |
| **Domain 4: Flow and timing** |
| **Was there an appropriate interval between index test(s) and reference standard?** Score low risk if all patients received colonoscopy and this was conducted within 3 months of the index test, or if some patients received records follow-up, this should be for a minimum of 3 months Score high risk if colonoscopies were not conducted within 3 months, or follow-up is for less than 3 months but more than 12 months Score unclear if the time intervals were unclear NB: likely most won't report time interval for colonoscopy  Yes/No/Unclear |
| **Did all patients receive a reference standard?** *Score* ***yes*** *if all patients got a reference standard, even if these were different (see next question)* *Score* ***no*** *if a partial verification reference standard: only some participants get any reference standard, e.g. those who test negative at FIT don’t get followed up or any further tests (these studies should be excluded) Score* ***unclear*** *if it is unclear who received the reference standard* |
| **Did patients receive the same reference standard?**  *The following score “****no****”:*  *Complete index test-dependent differential verification reference standard: participants get a different reference standard according to the index test result, e.g. FIT positive get colonoscopy, FIT negative get records follow-up Differential verification dependent on other known or unknown factors: participants get a different reference standard according to some known or unknown factors, e.g. those with clinical signs or symptoms proceed to colonoscopy regardless of FIT, whilst reminder get records follow-up  The following score “****yes****”: All received the same reference standard, e.g. all get colonoscopy* |
| **Were all patients included in the analysis?**  *Score* ***yes*** *if all patients who were recruited/enrolled into the study were included in the analysis or if an acceptable explanation (i.e missing at random) is provided for any discrepancy Score* ***no*** *if there are participants excluded from the analysis and no/concerning explanation is given for any discrepancy Score* ***unclear*** *if insufficient information is given to assess whether any patients were excluded from the analysis.* |
| **Risk of bias summary score: Could the patient flow have introduced bias?** Low/High/Unclear (THIS IS A SUMMARY SCORE BASED ON ANSWERS TO QUESTIONS 1 TO 4)  Score Low if all domains are Yes Score High if one or more domain is No Anything in between score Unclear |

### **Summary of scores**

**Table 2: Reviewers’ assessment of risk of bias and applicability for all tests in all analyses using QUADAS-2^1^**

|  | **Analyses^a^** | **Risk of Bias items** | | | | **Applicability items** | | |
| --- | --- | --- | --- | --- | --- | --- | --- | --- |
|  |  | **Patient selection** | **Index test** | **Reference standard** | **Patient flow** | **Patients and setting** | **Index test** | **Reference standard** |
| **HM-JACKarc – all studies** | | | | | | | | |
| Benton 2022^2^ | 2 | High | Low | Unclear | Low | High | Low | Low |
| Chapman 2021^3^ | 4 | High | Low | Unclear | Unclear | High | Low | Low |
| Cunin 2020^4^ | 4; anaemia | Low | Low | High | High | High | Low | Low |
| D'Souza 2020a^5^ | 1, 2, 3 | High | Low | Unclear | Low | Unclear | Low | Low |
| D'Souza 2021a^6^ | 2; anaemia | Unclear | Low | Unclear | Unclear | High | Low | Low |
| D'Souza 2021c^7^, D’Souza 2021b^8^ | 2; age | Unclear | Low | Unclear | Unclear | High | Low | Low |
| Elbeltagi 2022^9^ | 4 | Unclear | Low | Unclear | High | High | Low | Low |
| Farrugia 2020^10^ | 2 | High | Low | Unclear | Unclear | High | Low | Low |
| Faux 2022^11^ | 4 | High | Low | High | High | High | Low | Low |
| Gerrard 2023^12^ | 1; single and Dual FIT; anaemia | High | Low | High | High | High | Low | Low |
| Godber 2016^13^ | 4 | High | Low | Unclear | Low | High | Low | Low |
| Johnstone 2022a^14^ | 1; Anaemia | Low | Low | Unclear | High | Low | Low | Low |
| MacDonald 2022^15^ | 1 | Low | Low | High | High | Unclear | Low | Low |
| Mowat 2021 & 2019^16, 17^ | 1 | Unclear | Low | High | High | Unclear | Low | Low |
| Nicholson (2018)^18a^ | 4 | Low | Low | High | High | High | High | Low |
| Nicholson (2020)^19^ | 4, sex | Low | Low | High | High | High | High | Low |
| Tang 2022^20^ | 4; anaemia | High | Low | Unclear | Unclear | High | Low | Low |
| Turvill 2021^21^ | 4; anaemia; age, medications, sex | High | High/Low | High | High | High | High/Low | Low |
| Turvill 2018^21^ | 2; single and Dual FIT | High | High | High | High | High | High | Low |
| Withrow 2022^22b^ | 3; anaemia; age | High | Low | High | High | High | Low | Low |
| **OC-Sensor – all studies** | | | | | | | | |
| Archer 2022^23^ | 4 | High | Low | Unclear | Unclear | High | Low | Low |
| Ayling 2019^24^ | Anaemia | High | Low | Low | High | High | Low | Low |
| Ball 2022^25^ (personal communication) | 3, sex | High | Low | High | High | High | Low | Low |
| Ball 2022^25^ | 4 | Low | Low | High | High | Low | Low | Low |
| Benton 2022^2^ | 2 | High | Low | Unclear | Low | High | Low | Low |
| Bujanda 2018^26^ | Aspirin | High | Low | Low | Unclear | High | Low | Low |
| Cama 2022^27^ | 1 | High | Low | High | High | High | Low | Low |
| Chapman 2021^3^ | 4 | High | Low | Unclear | Unclear | High | Low | Low |
| Crooks 2023^28^, Bailey J 2021a^29^ | 1 | High | Low | High | High | High | Low | Low |
| Georgiou Delisle 2022^30^ | 1 | High | Low | Unclear | Low | High | Low | Low |
| Hunt 2022^31^ | Dual FIT | High | Low | Unclear | Unclear | High | Low | Low |
| Juul 2018^32^ | 4, anaemia | High | Low | High | High | High | Low | Low |
| Laszlo 2021^33^ | 4 | High | Low | Low | High | High | Low | Low |
| Maclean 2021a^34^ | 4 | Low | Low | High | High | High | Low | Low |
| Morales-Arraez 2018^35^ | Anaemia | High | Low | Unclear | Unclear | High | Low | Low |
| Mowat 2016^36^ | 4 | High | High (LoD); Low (10ug/g) | Low | High | High | Low | Low |
| Pin Vieto 2020^37^ | 4 | Unclear | Low | High | High | Unclear | Low | Low |
| Rodriguez-Alonso 2018^38^, Rodriguez-Alonso 2019^39^ | Anaemia, PPIs | High | Unclear | Unclear | Unclear | High | Low | Low |
| **FOB-Gold – all studies** | | | | | | | | |
| Benton 2022^2^ | 2 | High | Low | Unclear | Low | High | Low | Low |
| MacLean 2022a^40^ | 2 | High | Low | Low | High | High | Low | Low |
| Jordaan 2023^41^ | 4 | Unclear | Low | High | High | High | Low | Low |
| **QuikRead Go – all studies** | | | | | | | | |
| Maclean 2021b^42^ | 2 | High | Low | Low | High | High | Low | Low |
| Tsapournas 2020^43^ | Dual FIT | High | Low | Low | High | High | Low | Low |
| **NS-Prime** | | | | | | | | |
| Benton 2022^2^ | 2 | High | Low | Unclear | Low | High | Low | Low |
| **IDK – all studies** | | | | | | | | |
| Sieg 1999^44^ | 4 | High | Hb: Low  Hb/Hp: High | Low | Unclear | High | High | Low |

RoB, Risk of bias

^a^ Numbers relate to population-type analyses

^b^ Nicholson 2020 and Withrow 2022 include some of the same patients, but it was not clear if the same methodology was used in both studies to select patients and conduct follow-up, so scores are provided for each study based on the information given for that study

1. Whiting P, Rutjes A, Westwood M, Mallett S, Deeks J, Reitsma J*, et al.* QUADAS-2: A Revised Tool for the Quality Assessment of Diagnostic Accuracy Studies. *Ann Intern Med* 2011;155:529-36.

2. Benton SC, Piggott C, Zahoor Z, O'Driscoll S, Fraser CG, D'Souza N*, et al.* A comparison of the faecal haemoglobin concentrations and diagnostic accuracy in patients suspected with colorectal cancer and serious bowel disease as reported on four different faecal immunochemical test systems. *Clinical Chemistry & Laboratory Medicine* 2022;60:1278-86.

3. Chapman CJ, Banerjea A, Humes DJ, Allen J, Oliver S, Ford A*, et al.* Choice of faecal immunochemical test matters: comparison of OC-Sensor and HM-JACKarc, in the assessment of patients at high risk of colorectal cancer. *Clinical Chemistry & Laboratory Medicine* 2021;59:721-8.

4. Cunin L, Khan AA, Ibrahim M, Lango A, Klimovskij M, Harshen R. FIT negative cancers: A right-sided problem? Implications for screening and whether iron deficiency anaemia has a role to play. *The Surgeon* 2021;19:27-32.

5. D'Souza N, Hicks G, Benton SC, Abulafi M. The diagnostic accuracy of the faecal immunochemical test for colorectal cancer in risk-stratified symptomatic patients. *Annals of the Royal College of Surgeons of England* 2020a;102:174-9.

6. D’Souza N, Delisle TG, Chen M, Benton SC, Abulafi M, the NFITSC. Faecal immunochemical testing in symptomatic patients to prioritize investigation: diagnostic accuracy from NICE FIT Study. *British Journal of Surgery* 2021a;108:804-10.

7. D'Souza N, Delisle TG, Chen M, Benton S, Abulafi M. Faecal immunochemical test is superior to symptoms in predicting pathology in patients with suspected colorectal cancer symptoms referred on a 2WW pathway: a diagnostic accuracy study. *Gut* 2021c;70:1130-8.

8. D’Souza N, Monahan K, Benton SC, Wilde L, Abulafi M, Group NFS*, et al.* Finding the needle in the haystack: the diagnostic accuracy of the faecal immunochemical test for colorectal cancer in younger symptomatic patients. *Colorectal Disease* 2021b;23:2539-49.

9. Elbeltagi A, Salama M, Boxall P, Roos J, Lim M. The Yield of Faecal Immunochemical Test in the Detection of Colorectal Cancer within a Fast-track Pathway at York, United Kingdom. *Turkish Journal of Colorectal Disease* 2022;32(3):178-85.

10. Farrugia A, Widlak M, Evans C, Smith SC, Arasaradnam R. Faecal immunochemical testing (FIT) in symptomatic patients: what are we missing? *Frontline Gastroenterol* 2020;11:28-33.

11. Faux JW, Cock K, Bromley R, Feldman M. Colorectal two-week wait service and quantitative FIT: it's not just about colon cancer. *Annals of the Royal College of Surgeons of England* 2022;104:257-60.

12. Gerrard AD, Maeda Y, Miller J, Gunn F, Theodoratou E, Noble C*, et al.* Double faecal immunochemical testing in patients with symptoms suspicious of colorectal cancer. *British Journal of Surgery* 2023;110:471-80.

13. Godber IM, Todd LM, Fraser CG, MacDonald LR, Younes HB. Use of a faecal immunochemical test for haemoglobin can aid in the investigation of patients with lower abdominal symptoms. *Clinical Chemistry & Laboratory Medicine* 2016;54:595-602.

14. Johnstone MS, Burton P, Kourounis G, Winter J, Crighton E, Mansouri D*, et al.* Combining the quantitative faecal immunochemical test and full blood count reliably rules out colorectal cancer in a symptomatic patient referral pathway. *International Journal of Colorectal Disease* 2022a;37:457-66.

15. MacDonald S, MacDonald L, Godwin J, Macdonald A, Thornton M. The diagnostic accuracy of the faecal immunohistochemical test in identifying significant bowel disease in a symptomatic population. *Colorectal Disease* 2022;24:257-63.

16. Mowat C, Digby J, Strachan JA, McCann R, Hall C, Heather D*, et al.* Impact of introducing a faecal immunochemical test (FIT) for haemoglobin into primary care on the outcome of patients with new bowel symptoms: a prospective cohort study. *BMJ Open Gastroenterology* 2019;6:e000293.

17. Mowat C, Digby J, Strachan JA, McCann RK, Carey FA, Fraser CG*, et al.* Faecal haemoglobin concentration thresholds for reassurance and urgent investigation for colorectal cancer based on a faecal immunochemical test in symptomatic patients in primary care. *Annals of Clinical Biochemistry* 2021;58:211-9.

18. Nicholson BD, James T, East JE, Grimshaw D, Paddon M, Justice S*, et al.* Experience of adopting faecal immunochemical testing to meet the NICE colorectal cancer referral criteria for low-risk symptomatic primary care patients in Oxfordshire, UK. *Frontline Gastroenterology* 2019;10:347-55.

19. Nicholson BD, James T, Paddon M, Justice S, Oke JL, East JE*, et al.* Faecal immunochemical testing for adults with symptoms of colorectal cancer attending English primary care: a retrospective cohort study of 14 487 consecutive test requests. *Alimentary Pharmacology & Therapeutics* 2020;52:1031-41.

20. Tang A, Chandler S, Torkington J, Harris DA, Dhruva Rao PK. Adapting the investigation of patients on urgent suspected cancer pathway with lower gastrointestinal symptoms across Wales during COVID-19. *Annals of the Royal College of Surgeons of England* 2022;26:26.

21. Turvill J, Mellen S, Jeffery L, Bevan S, Keding A, Turnock D. Diagnostic accuracy of one or two faecal haemoglobin and calprotectin measurements in patients with suspected colorectal cancer. *Scandinavian Journal of Gastroenterology* 2018;53:1526-34.

22. Withrow DR, Shine B, Oke J, Tamm A, James T, Morris E*, et al.* Combining faecal immunochemical testing with blood test results for colorectal cancer risk stratification: a consecutive cohort of 16,604 patients presenting to primary care. *BMC Medicine* 2022;20:116.

23. Archer T, Aziz I, Kurien M, Knott V, Ball A. Prioritisation of lower gastrointestinal endoscopy during the COVID-19 pandemic: outcomes of a novel triage pathway. *Frontline Gastroenterology* 2022;13:225-30.

24. Ayling RM, Lewis SJ, Cotter F. Potential roles of artificial intelligence learning and faecal immunochemical testing for prioritisation of colonoscopy in anaemia. *British Journal of Haematology* 2019;185:311-6.

25. Ball AJ, Aziz I, Parker S, Sargur RB, Aldis J, Kurien M. Fecal Immunochemical Testing in Patients With Low-Risk Symptoms of Colorectal Cancer: A Diagnostic Accuracy Study. *Journal of the National Comprehensive Cancer Network* 2022;20:989-96.e1.

26. Bujanda L, Sarasqueta C, Vega P, Salve M, Quintero E, Alvarez-Sánchez V*, et al.* Effect of aspirin on the diagnostic accuracy of the faecal immunochemical test for colorectal advanced neoplasia. *United European Gastroenterol J* 2018;6:123-30.

27. Cama R, Kapoor N, Sawyer P, Patel B, Landy J. Evaluation of 13,466 Fecal Immunochemical Tests in Patients Attending Primary Care for High- and Low-Risk Gastrointestinal Symptoms of Colorectal Cancer. *Digestive Diseases & Sciences* 2022;10:10.

28. Crooks C, Banerjea A, Jones J, Chapman C, Oliver S, West J*, et al.* Assessing empirical thresholds for investigation in people referred on a symptomatic colorectal cancer pathway: a cohort study utilising faecal immunochemical and blood tests in England. *medRxiv* 2023; 10.1101/2023.03.29.23287919:2023.03.29.23287919.

29. Bailey JA, Weller J, Chapman CJ, Ford A, Hardy K, Oliver S*, et al.* Faecal immunochemical testing and blood tests for prioritization of urgent colorectal cancer referrals in symptomatic patients: A 2-year evaluation. *BJS Open* 2021a;5(2) (no pagination).

30. Georgiou Delisle T, D'Souza N, Tan J, Najdawi A, Chen M, Ward H*, et al.* Introduction of an integrated primary care faecal immunochemical test referral pathway for patients with suspected colorectal cancer symptoms. *Colorectal Disease* 2022a;08:08.

31. Hunt N, Rao C, Logan R, Chandrabalan V, Oakey J, Ainsworth C*, et al.* A cohort study of duplicate faecal immunochemical testing in patients at risk of colorectal cancer from North-West England. *BMJ Open* 2022;12:e059940.

32. Juul JS, Hornung N, Andersen B, Laurberg S, Olesen F, Vedsted P. The value of using the faecal immunochemical test in general practice on patients presenting with non-alarm symptoms of colorectal cancer. *British Journal of Cancer* 2018;119(4):471-9.

33. Laszlo HE, Seward E, Ayling RM, Lake J, Malhi A, Stephens C*, et al.* Faecal immunochemical test for patients with 'high-risk' bowel symptoms: a large prospective cohort study and updated literature review. *British Journal of Cancer* 2022;126:736-43.

34. Maclean W, Limb C, Mackenzie P, Whyte MB, Benton SC, Rockall T*, et al.* Adoption of faecal immunochemical testing for 2-week-wait colorectal patients during the COVID-19 pandemic: an observational cohort study reporting a new service at a regional centre. *Colorectal Disease* 2021a;23(7):1622-9.

35. Morales Arraez D, Carrillo G, Adrian M, Gimeno Z, Quintero A. Role of faecal immunochemical testing in the diagnostic workup of patients with iron deficiency anaemia. *United Eur Gastroenterol J* 2018;6:A403–A4.

36. Mowat C, Digby J, Strachan JA, Wilson R, Carey FA, Fraser CG*, et al.* Faecal haemoglobin and faecal calprotectin as indicators of bowel disease in patients presenting to primary care with bowel symptoms. *Gut* 2016;65:1463-9.

37. Pin-Vieito N, Garcia Nimo L, Bujanda L, Roman Alonso B, Gutierrez-Stampa MA, Aguilar-Gama V*, et al.* Optimal diagnostic accuracy of quantitative faecal immunochemical test positivity thresholds for colorectal cancer detection in primary health care: A community-based cohort study. *United European Gastroenterology Journal* 2021;9:256-67.

38. Rodriguez-Alonso L, Rodriguez-Moranta F, Arajol C, Gilabert P, Serra K, Martin A*, et al.* Proton pump inhibitors reduce the accuracy of faecal immunochemical test for detecting advanced colorectal neoplasia in symptomatic patients. *PLoS One* 2018;13:e0203359.

39. Rodriguez-Alonso L, Rodriguez-Moranta F, Ruiz-Cerulla A, Arajol C, Serra K, Gilabert P*, et al.* The use of faecal immunochemical testing in the decision-making process for the endoscopic investigation of iron deficiency anaemia. *Clin Chem Lab Med* 2020;58:232-9.

40. MacLean W, Zahoor Z, O'Driscoll S, Piggott C, Whyte MB, Rockall T*, et al.* Comparison of the QuikRead go<sup></sup>point-of-care faecal immunochemical test for haemoglobin with the FOB Gold Wide<sup></sup>laboratory analyser to diagnose colorectal cancer in symptomatic patients. *Clinical Chemistry and Laboratory Medicine* 2022a;60(1):101-8.

41. Jordaan M, Welbourn H, Tyldsley K, Mustafa A, Mevada C, Harrison M*, et al.* Development of a primary care pathway for using a faecal immunochemical test (FIT) to triage patients presenting with bowel symptoms. In: Medicine PL, ed.; 2022.

42. Maclean W, Mackenzie P, Limb C, Zahoor Z, Whyte MB, Rockall T*, et al.* Diagnostic accuracy of point of care faecal immunochemical testing using a portable high-speed quantitative analyser for diagnosis in 2-week wait patients. *Colorectal Disease* 2021b;23:2376-86.

43. Tsapournas G, Hellström PM, Cao Y, Olsson LI. Diagnostic accuracy of a quantitative faecal immunochemical test vs. symptoms suspected for colorectal cancer in patients referred for colonoscopy. *Scandinavian Journal of Gastroenterology* 2020;55:184-92.

44. Sieg A, Thoms C, Lüthgens K, John MR, Schmidt-Gayk H. Detection of colorectal neoplasms by the highly sensitive hemoglobin-haptoglobin complex in feces. *International Journal of Colorectal Disease* 1999;14:267-71.
