# Supplementary material for: Faecal immunochemical tests for patients with symptoms suggestive of colorectal cancer: An updated systematic review and multiple‐threshold meta‐analysis of diagnostic test accuracy studies
Source: Colorectal Dis. 2024 Dec 17;27(1):e17255. doi: 10.1111/codi.17255 (PMC11683176; doi:10.1111/codi.17255)
Supplement: Supplementary file 5 — Data S5. [file CODI-27-0-s012.docx]

**Statistical methods for the meta-analysis**

For tests where data was available from more than one study, pooled estimates of diagnostic parameters were estimated using the modelling approach described in Jones *et al.*^8^. Random effects meta-analysis was used to account for the heterogeneity between studies.

Analyses were conducted in R^9^ using the JAGS Markov chain Monte Carlo (MCMC) sampler and the RJAGS interface package.^10^ Convergence to the target posterior distributions was assessed using the Gelman-Rubin statistic^11^ for three chains with different initial values. For all analyses, a burn in of 50,000 iterations of the Markov chain was used, with a further 30,000 iterations retained to estimate parameters after thinning by retaining every 10^th^ sample.

Model fit penalising for complexity was compared using the Deviance Information Criterion (DIC).^12^ Models with lower values of the DIC are preferred

**1. Statistical model**

The statistical model is briefly described following the notation in Jones et al. ^38^ True disease status is assumed to be known through application of a perfect gold standard test. Populations without and with CRC are indexed by $j=1, 2$ respectively. Each study, *i*, reports estimates of sensitivity and specificity, or directly reports count data, at$T_{i}$ distinct thresholds. Test results above a given threshold are considered positive.

The observed count data is modelled using multinomial likelihoods, reparametrized as conditional binomial distributions for computational convenience. The model assumes that some transformation, $g()$ of the continuous test results in population $j$ of study $i$ has a logistic distribution with mean $\mu_{ij}$ and scale parameter $\sigma_{ij}$. In our analyses we pre-specify $g\left( \right)=\log_{e} ()$. Jones et al. describe the more flexible (but computationally intensive) case where $g\left( \right)$ is in the set of Box-Cox transformation, defined by a parameter which is estimated alongside other model parameters.

*Within study model*

The probability of a positive test result at threshold $C_{it}$ in population $j$ of study $i$ is

| $logit\left( pr_{ijt} \right)=\frac{\left( \mu_{ij}-g(C_{it}) \right)}{\sigma_{ij}}$  For $j=1$ we have $pr_{i1t}$ (the false positive rate, FPR=1-sensitivity) and for $j=2$ we have $pr_{i2t}$ (the true positive rate, TPR=sensitivity) | (1) |
| --- | --- |

*Between study model*

The study specific location ($\mu_{ij}$) and scale ($\sigma_{ij}$) parameters are modelled as random effects. Across studies, $\mu_{ij}$, has mean $m_{\mu j}$ and standard deviation $\tau_{\mu j}$.while ${log(\sigma}_{ij})$has mean $m_{\sigma j}$ and standard deviation $\tau_{\sigma j}$

Different options for the correlation structure between these four sets of random effects are described in Jones *et al.*: i) Full correlation matrix, ii) structured correlations matrix iii) independence model with the four sets of random effects assumed to be independent of each other. Models with a structured correlation matrix and assuming independence were explored. Including additional parameters for between-study correlations did not improve the model fit according to the DIC (see Table 2), therefore the simpler independence model was used for all main analyses.

Prior distributions are required for the four hyperparameters : $m_{\mu j}$ , $\tau_{\mu j}$ , $m_{\sigma j}$ $\tau_{\sigma j}$ for $j=1,2$. For analyses with sufficient sample data, standard reference priors as used in Jones *et al.*were used. Normal (0, ${10}^{2}$) prior distribution were given to all means ($m_{\mu j}$, $m_{\sigma j}$), and Uniform (0,5) prior for between study standard deviations ($\tau_{\mu j}, \tau_{\sigma j}$)

**2. Prior distributions**

For analyses with small numbers of contributing studies, informative priors were used for the between study standard deviations. These were informed by fitting log-normal distributions to posterior samples from the analyses of all test types together. This was considered to be a conservative option. A truncation was also applied, based on the 95^th^ centile of the posterior distribution. Parameter values for all analyses are provided in Table 1.

**Table 1. Parameters used to inform priors for syntheses with less than 5 studies**

| **Parameter** | **CRC outcomes (S=28)** | | | **AA outcomes (S=9)** | | | **IBD outcomes (S=9)** | | |
| --- | --- | --- | --- | --- | --- | --- | --- | --- | --- |
|  | **mean** | **sd** | **truncation** | **mean** | **sd** | **truncation** | **mean** | **sd** | **truncation** |
| $\tau_{\mu1}$ | 0.2698 | 0.1543 | 1.703 | 0.2859 | 0.3432 | 2.478 | 0.4532 | 0.3561 | 3.027 |
| $\tau_{\mu2}$ | -0.8489 | 0.2995 | 0.67 | -0.4517 | 0.8583 | 2.174 | 0.3827 | 0.8695 | 3.92 |
| $\tau_{\sigma1}$ | -0.8863 | 0.1559 | 0.538 | -0.5228 | 0.415 | 1.247 | -0.4273 | 0.4077 | 1.345 |
| $\tau_{\sigma2}$ | -1.4215 | 0.2693 | 0.368 | 0.152 | 0.5711 | 3.079 | -0.8792 | 0.9243 | 1.339 |

**3. Model fit**

Model fit for all analyses shown in Table 2. Differences in DIC across the two correlations structures (structured and independent) were minimal. Since including additional parameters for between-study correlations did not improve the model fit according to the DIC, the simpler model structure was preferred and all analyses presented in the report use the model of Jones *et al.*^38^ with the four sets of random effects assumed to be independent of each other (independence model).

**Table 2 Meta-analysis model fit statistics**

| **Tests in analysis** | **Populations** | **Studies** | **Correlation structure** | **Model fit** | | |
| --- | --- | --- | --- | --- | --- | --- |
|  |  |  |  | $\bar{D}$   \|  \| \| --- \| | **pD** | **DIC** |
| **CRC outcomes** |  |  |  |  |  |  |
| **All** | All | 28 | S | 6718.40 | 72.91 | 6791.31 |
|  | All | 28 | I | 6711.25 | 71.78 | 6783.03 |
|  | 1,2,3 | 13 | I | 1780.45 | 31.63 | 1812.08 |
|  | 1 | 8 | I | 1541.81 | 21.73 | 1563.54 |
|  | 2 | 5 | I | 173.87 | NaN | 173.87 |
|  | 2 | 5 | I | 175.77 | 10.26 | 186.02 |
|  | 3 | 3 | I | 107.51 | NaN | 107.51 |
| **HM JACKArc** | All | 16 | S | 5297.88 | 41.45 | 5339.32 |
|  | All | 16 | I | 5296.11 | 40.46 | 5336.56 |
|  | 1,2,3 | 9 | I | 648.54 | 19.29 | 667.83 |
|  | 1 | 5 | I | 495.25 | 12.69 | 507.93 |
|  | 2 | 4 | I | 143.50 | 8.11 | 151.60 |
|  | 3 | 2 | I | 39.00 | Nan | 39.00 |
| **OC Sensor** | All | 11 | S | 1395.86 | 28.05 | 1423.92 |
|  | All | 11 | I | 1394.99 | 28.71 | 1423.70 |
|  | 1,2,3 | 4 | I | 1112.37 | 11.18 | 1123.55 |
|  | 1 | 3 | I | 1044.86 | 9.35 | 1054.21 |
| **FOB Gold** | All | 3 | I | 114.56 | NaN | Nan |
|  |  |  |  |  |  |  |
| **AA outcomes** |  |  |  |  |  |  |
| **All tests** | All | 9 | I | 308.81 | 23.41 | 332.21 |
| **HM-JACKarc** | All | 6 | I | 240.87 | 13.86 | 254.74 |
| **OC Sensor** | All | 2 | I | 38.67 | 6.02 | 44.69 |
|  |  |  |  |  |  |  |
| **IBD outcomes** |  |  |  |  |  |  |
| **All tests** | All | 9 | I | 286.38 | 23.97 | 310.36 |
| **HM-JACKarc** | All | 6 | I | 220.37 | 14.64 | 235.01 |
| **OC Sensor** | All | 2 | I | 38.27 | 6.12 | 44.39 |
|  |  |  |  |  |  |  |
| **DUAL FIT** |  |  |  |  |  |  |
| **All tests** | All | 4 | I | 63.87 | 8.03 | 71.90 |
