# Supplementary material for: Faecal immunochemical tests for patients with symptoms suggestive of colorectal cancer: An updated systematic review and multiple‐threshold meta‐analysis of diagnostic test accuracy studies
Source: Colorectal Dis. 2024 Dec 17;27(1):e17255. doi: 10.1111/codi.17255 (PMC11683176; doi:10.1111/codi.17255)
Supplement: Supplementary file 6 — Data S6. [file CODI-27-0-s018.docx]

## Clinical review: Table of studies excluded on the basis of their full text, with rationale

| **Reason** | **Number of studies excluded** | **References** |
| --- | --- | --- |
| Analytical performance | 9 | ^1-9^ |
| Crossover, no new data or superseded | 14 | ^10-23^ |
| Editorial, comment, letter | 7 | ^24-30^ |
| Incorrect population | 41 | ^31-71^ |
| Insufficient data to calculate DTA /data ambiguous/not DTA study | 11 | ^72-82^ |
| Outcome Not CRC or CRC only | 4 | ^83-86^ |
| Not English language | 1 | ^87^ |
| Not FIT or in-scope test | 6 | ^88-93^ |
| Ongoing study or systematic review | 10 | ^94-103^ |
| Systematic review or review | 13 | ^104-116^ |
| Threshold not reported | 2 | ^117 118^ |

1. Comparison IDK® TurbiFIT® and IDK® Hemoglobin ELISA. 2022

2. Gies A, Gruner LF, Schrotz-King P, et al. Effect of Imperfect Compliance With Instructions for Fecal Sample Collection on Diagnostic Performance of 9 Fecal Immunochemical Tests. *Clin Gastroenterol Hepatol* 2019;17(9):1829-39.e4. doi: 10.1016/j.cgh.2019.03.001 [published Online First: 2019/03/10]

3. James T, Nicholson BD, Marr R, et al. Faecal immunochemical testing (FIT): sources of result variation based on three years of routine testing of symptomatic patients in English primary care. *British Journal of Biomedical Science* 2021;78(4):211-17.

4. Mellen S, de Ferrars M, Chapman C, et al. Evaluation of sample stability for a quantitative faecal immunochemical test and comparison of two sample collection approaches. *Ann Clin Biochem* 2018;55(6):657-64. doi: 10.1177/0004563218766393 [published Online First: 2018/03/15]

5. O'Driscoll S, Carroll M, Maclean W, et al. Assessment of the analytical performance of point-of-care faecal immunochemical tests for haemoglobin. *Ann Clin Biochem* 2021;58(3):181-89. doi: 10.1177/0004563220986595 [published Online First: 2020/12/24]

6. O’Driscoll S, Piggott C, Bruce H, et al. An evaluation of ten external quality assurance scheme (EQAS) materials for the faecal immunochemical test (FIT) for haemoglobin. 2021;59(2):307-13. doi: doi:10.1515/cclm-2020-0210

7. Piggott C, Carroll MRR, John C, et al. Analytical evaluation of four faecal immunochemistry tests for haemoglobin. *Clin Chem Lab Med* 2020;59(1):173-78. doi: 10.1515/cclm-2020-0251 [published Online First: 2020/07/22]

8. Piggott C, Shugaa Z, Benton SC. Independent internal quality control (IQC) for faecal immunochemical tests (FIT) for haemoglobin: use of FIT manufacturers’ IQC for other FIT systems. 2021;59(2):e41-e43. doi: doi:10.1515/cclm-2020-0286

9. Zubero MB, Arana-Arri E, Pijoan JI, et al. Population-based colorectal cancer screening: comparison of two fecal occult blood test. *Frontiers in Pharmacology* 2014;4 doi: 10.3389/fphar.2013.00175

10. Ayling RM, Machesney M. Service evaluation of faecal immunochemical testing introduced for use in North East London for patients at low risk of colorectal cancer. *Journal of Clinical Pathology* 2021;74(3):163-66. doi: <https://dx.doi.org/10.1136/jclinpath-2020-206632>

11. Bailey J, Chapman C, Jones J, et al. Sociodemographic variations in the uptake of faecal immunochemical tests (fit) in a primary care symptomatic pathway for colorectal cancer. *Colorectal Disease* 2022;24(Supplement 3):8-9. doi: <https://dx.doi.org/10.1111/codi.16273>

12. Bailey J, Ibrahim H, Bunce J, et al. Quantitative FIT stratification is superior to NICE referral criteria NG12 in a Two-Week Wait Colorectal Cancer population. Submitted to IJCD 2020, 2020.

13. Bailey JA, Khawaja A, Andrews H, et al. GP access to FIT increases the proportion of colorectal cancers detected on urgent pathways in symptomatic patients in Nottingham. *Surgeon* 2021b;19(2):93-102. doi: 10.1016/j.surge.2020.03.002 [published Online First: 2020/04/25]

14. Bailey SER, Abel GA, Atkins A, et al. Diagnostic performance of a faecal immunochemical test for patients with low-risk symptoms of colorectal cancer in primary care: an evaluation in the South West of England. *British Journal of Cancer* 2021;124(7):1231-36. doi: 10.1038/s41416-020-01221-9

15. Chapman C, Bunce J, Oliver S, et al. Service evaluation of faecal immunochemical testing and anaemia for risk stratification in the 2-week-wait pathway for colorectal cancer. *BJS Open* 2019;3(3):395-402. doi: <https://dx.doi.org/10.1002/bjs5.50131>

16. Chapman C, Thomas C, Morling J, et al. Early clinical outcomes of a rapid colorectal cancer diagnosis pathway using faecal immunochemical testing in Nottingham. *Colorectal Disease* 2020;22(6):679-88. doi: <https://doi.org/10.1111/codi.14944>

17. Ibrahim HAH, Chapman C, Morling J, et al. Keeping FIT: Faecal haemoglobin measurement with FIT has stratification value in the diagnosis of colorectal cancer in all symptom and age groups. *Colorectal Disease* 2019;21(Supplement 2):10. doi: <https://dx.doi.org/10.1111/codi.14770>

18. Khasawneh F, Osborne R, Stephenson J, et al. Faecal immunochemical testing is a cost-effective way to stratify symptomatic patients for urgent straight to test investigation. *Colorectal Dis* 2020;22(suppl. 1):6-12.

19. McSorley ST, Digby J, Clyde D, et al. Yield of colorectal cancer at colonoscopy according to faecal haemoglobin concentration in symptomatic patients referred from primary care. *Colorectal Disease* 2021;23(7):1615-21.

20. Steele R, Fraser C. Haemoglobin for Timely Assessment of Patients with Symptoms of Colorectal Disease in Olsen Timely Diagnosis of Colorectal Disease.: Springer, 2018.

21. Widlak MM, Neal M, Daulton E, et al. Risk stratification of symptomatic patients suspected of colorectal cancer using faecal and urinary markers. *Colorectal Disease* 2018;20(12):O335-O42. doi: <https://dx.doi.org/10.1111/codi.14431>

22. Widlak MM, Neal M, Daulton E, et al. Risk stratification of symptomatic patients suspected of colorectal cancer using faecal and urinary markers. *Colorectal Dis* 2018;20(12):O335-o42. doi: 10.1111/codi.14431 [published Online First: 2018/09/25]

23. Widlak MM, Thomas CL, Thomas MG, et al. Diagnostic accuracy of faecal biomarkers in detecting colorectal cancer and adenoma in symptomatic patients. *Alimentary Pharmacology & Therapeutics* 2017;45(2):354-63.

24. Burke CA, Lieberman D, Feuerstein JD. AGA Clinical Practice Update on Approach to the Use of Noninvasive Colorectal Cancer Screening Options: Commentary. *Gastroenterology* 2022;162(3):952-56.

25. Craig M, Turner J, Torkington J, et al. Faecal immunochemical test: challenges and opportunities for cancer diagnosis in primary care. *British Journal of General Practice* 2022;72(721):366-67.

26. D’Souza N, Anthony B, Muti A. Faecal immunochemical testing in general practice. *British Journal of General Practice* 2019;69(679):60. doi: 10.3399/bjgp19X700853

27. Mowat C, Digby J, Strachan JA, et al. Low Sensitivity of Fecal Immunochemical Tests (FIT) for Detection of Sessile Serrated Adenomas/Polyps Confirmed Over Clinical Setting, Geography, and FIT System. *Dig Dis Sci* 2019;64(10):3024-26. doi: 10.1007/s10620-019-05661-z [published Online First: 2019/05/20]

28. Ray K. Prognostic potential of repeated faecal haemoglobin levels in CRC detection. *Nature Reviews Gastroenterology & Hepatology* 2022;19(7):416.

29. Rees CJ, Hamilton W. BSG guidelines on faecal immunochemical testing: Are they 'FIT' for purpose? *Gut* 2022;(no pagination) doi: <https://dx.doi.org/10.1136/gutjnl-2022-328201>

30. Trivedi M, Gupta S. Is Promotion of Fecal Immunochemical Testing "FIT" to Address COVID-19 Disruptions to Colorectal Cancer Screening? *Gastroenterology* 2022;162(6):1761-62.

31. Ali O, Gupta S, Brain K, et al. Acceptability of alternative technologies compared with faecal immunochemical test and/or colonoscopy in colorectal cancer screening: A systematic review. *Journal of Medical Screening* 2022:9691413221109999.

32. Auge JM, Fraser CG, Rodriguez C, et al. Clinical utility of one versus two faecal immunochemical test samples in the detection of advanced colorectal neoplasia in symptomatic patients. *Clinical Chemistry & Laboratory Medicine* 2016;54(1):125-32.

33. Auge JM, Rodriguez C, Espanyol O, et al. An evaluation of the SENTiFIT 270 analyser for quantitation of faecal haemoglobin in the investigation of patients with suspected colorectal cancer. *Clinical Chemistry & Laboratory Medicine* 2018;56(4):625-33.

34. Cahill C, Lipson ME, Afzal AR, et al. Improved Survival in a Cohort of Patients 75 years and over with FIT-Detected Colorectal Cancer. *Diseases of the Colon & Rectum* 2022;28:28.

35. Chandrapalan S, Hee SW, Widlak MM, et al. Performance of the faecal immunochemical test for the detection of colorectal neoplasms and the role of proton pump inhibitors in their diagnostic accuracy. *Colorectal Disease* 2021;23(7):1649-57.

36. Cubiella J, Vega P, Salve M, et al. Development and external validation of a faecal immunochemical test-based prediction model for colorectal cancer detection in symptomatic patients. *BMC Medicine* 2016;14(1):128. doi: 10.1186/s12916-016-0668-5

37. Digby J, Cleary S, Gray L, et al. Faecal haemoglobin can define risk of colorectal neoplasia at surveillance colonoscopy in patients at increased risk of colorectal cancer. *United European Gastroenterology Journal* 2020;8(5):559-66.

38. Digby J, Strachan JA, McCann R, et al. Measurement of faecal haemoglobin with a faecal immunochemical test can assist in defining which patients attending primary care with rectal bleeding require urgent referral. *Annals of Clinical Biochemistry* 2020;57(4):325-27. doi: 10.1177/0004563220935622

39. Digby J, Strachan JA, Mowat C, et al. Appraisal of the faecal haemoglobin, age and sex test (FAST) score in assessment of patients with lower bowel symptoms: an observational study. *BMC Gastroenterology* 2019;19(1):213.

40. Eskelinen M, Meklin J, Guimaraes DP, et al. The ColonView (CV) Quick Test for Fecal Occult Blood Shows Significantly Higher Diagnostic Accuracy in Detecting Distal than Proximal Colorectal Cancer. *Anticancer Research* 2022;42(4):1879-91.

41. Gies A, Cuk K, Schrotz-King P, et al. Direct comparison of ten quantitative fecal immunochemical tests for hemoglobin stability in colorectal cancer screening. *Clinical and Translational Gastroenterology* 2018;9 doi: 10.1038/s41424-018-0035-2

42. Gies A, Cuk K, Schrotz-King P, et al. Direct Comparison of Diagnostic Performance of 9 Quantitative Fecal Immunochemical Tests for Colorectal Cancer Screening. *Gastroenterology* 2018;154(1):93-104. doi: 10.1053/j.gastro.2017.09.018 [published Online First: 2017/09/30]

43. Gies A, Cuk K, Schrotz-King P, et al. Combination of Different Fecal Immunochemical Tests in Colorectal Cancer Screening: Any Gain in Diagnostic Performance? *Cancers* 2019; 11(1).

44. Gies A, Niedermaier T, Alwers E, et al. Consistent Major Differences in Sex- and Age-Specific Diagnostic Performance among Nine Faecal Immunochemical Tests Used for Colorectal Cancer Screening. *Cancers (Basel)* 2021;13(14) doi: 10.3390/cancers13143574 [published Online First: 2021/07/25]

45. Habbu PP, Ananthi N, Shaikh AK. Study of Specificity, Sensitivity, Efficiency & Clinical Correlation between Timp-1 and Mif Protein as Biochemical Markers in Colorectal Cancer Patients. *International Journal of Pharmaceutical Sciences and Research* 2022;13(8):3298-303. doi: <https://dx.doi.org/10.13040/IJPSR.0975-8232.13%288%29.3298-03>

46. Herrero J-M, Vega P, Salve M, et al. Symptom or faecal immunochemical test based referral criteria for colorectal cancer detection in symptomatic patients: a diagnostic tests study. *BMC Gastroenterology* 2018;18(1):155. doi: 10.1186/s12876-018-0887-7

47. Hicks G, D’Souza N, Georgiou Delisle T, et al. Using the faecal immunochemical test in patients with rectal bleeding: evidence from the NICE FIT study. *Colorectal Disease* 2021;23(7):1630-38. doi: <https://doi.org/10.1111/codi.15593>

48. Jin P, You P, Fang J, et al. Comparison of Performance of Two Stool DNA Tests and a Fecal Immunochemical Test in Detecting Colorectal Neoplasm: A Multicenter Diagnostic Study. *Cancer Epidemiology, Biomarkers & Prevention* 2022;31(3):654-61.

49. Jin P, You P, Fang J, et al. Comparison of Performance of Two Stool DNA Tests and a Fecal Immunochemical Test in Detecting Colorectal Neoplasm: A Multicenter Diagnostic Study. *Cancer Epidemiol Biomarkers Prev* 2022;31(3):654-61. doi: 10.1158/1055-9965.Epi-21-0991 [published Online First: 2021/12/23]

50. Kapidzic A, van Roon AH, van Leerdam ME, et al. Attendance and diagnostic yield of repeated two-sample faecal immunochemical test screening for colorectal cancer. *Gut* 2017;66(1):118-23.

51. Kaul A, Shah A, Magill F, et al. Immunological faecal occult blood testing: a discriminatory test to identify colorectal cancer in symptomatic patients. *International Journal of Surgery* 2013;11(4):329-31.

52. Lincoln A, Benton S, Piggott C, et al. Exploring the utility and acceptability of Faecal immunochemical testing (FIT) as a novel intervention for the improvement of colorectal Cancer (CRC) surveillance in individuals with lynch syndrome (FIT for lynch study): a single-arm, prospective, multi-centre, non-randomised study. *BMC Cancer* 2022;22(1):1144.

53. Lu DC, Zhang QF, Li L, et al. Methylated Septin9 has moderate diagnostic value in colorectal cancer detection in Chinese population: a multicenter study. *BMC Gastroenterology* 2022;22(1):232.

54. Lué A, Hijos G, Sostres C, et al. The combination of quantitative faecal occult blood test and faecal calprotectin is a cost-effective strategy to avoid colonoscopies in symptomatic patients without relevant pathology. *Therap Adv Gastroenterol* 2020;13:1756284820920786. doi: 10.1177/1756284820920786 [published Online First: 2020/06/12]

55. Luthgens K, Maier A, Kampert I, et al. Hemoglobin-haptoglobin-complex: a highly sensitive assay for the detection of fecal occult blood. *Clinical laboratory* 1998;44(7-8):543-51.

56. Mattar R, Marques SB, Minata MK, et al. Diagnostic Accuracy of One Sample or Two Samples Quantitative Fecal Immunochemical Tests for Intestinal Neoplasia Detection. *Arquivos de Gastroenterologia* 2020;57(3):316-22.

57. Meester RGS, van de Schootbrugge-Vandermeer HJ, Breekveldt ECH, et al. Faecal occult blood loss accurately predicts future detection of colorectal cancer. A prognostic model. *Gut* 2022;10:10.

58. Navarro M, Hijos G, Sostres C, et al. Reducing the Cut-Off Value of the Fecal Immunochemical Test for Symptomatic Patients Does Not Improve Diagnostic Performance. *Frontiers in Medicine* 2020;7:410.

59. Parente F, Marino B, Ilardo A, et al. A combination of faecal tests for the detection of colon cancer: a new strategy for an appropriate selection of referrals to colonoscopy? A prospective multicentre Italian study. *Eur J Gastroenterol Hepatol* 2012;24(10):1145-52. doi: 10.1097/MEG.0b013e328355cc79 [published Online First: 2012/06/28]

60. Rodríguez-Alonso L, Rodríguez-Moranta F, Ruiz-Cerulla A, et al. An urgent referral strategy for symptomatic patients with suspected colorectal cancer based on a quantitative immunochemical faecal occult blood test. *Digestive and Liver Disease* 2015;47(9):797-804. doi: <https://doi.org/10.1016/j.dld.2015.05.004>

61. Small S, Coulson R, Spence R, et al. Is qFIT a useful tool in prioritising symptomatic patients referred with suspect colorectal cancer in the COVID-19 era? *Ulster Medical Journal* 2022;91(2):79-84.

62. Suehiro Y, Zhang Y, Hashimoto S, et al. Highly sensitive faecal DNA testing of TWIST1 methylation in combination with faecal immunochemical test for haemoglobin is a promising marker for detection of colorectal neoplasia. *Annals of Clinical Biochemistry* 2018;55(1):59-68. doi: <https://dx.doi.org/10.1177/0004563217691064>

63. Switalski J, Tatara T, Wnuk K, et al. Clinical Effectiveness of Faecal Immunochemical Test in the Early Detection of Colorectal Cancer-An Umbrella Review. *Cancers* 2022;14(18):09.

64. Tsapournas G, Hellstrom PM, Cao Y, et al. Diagnostic accuracy of a quantitative faecal immunochemical test vs. symptoms suspected for colorectal cancer in patients referred for colonoscopy. *Scandinavian Journal of Gastroenterology* 2020;55(2):184-92.

65. van Turenhout ST, Oort FA, van der Hulst RWM, et al. Prospective cross-sectional study on faecal immunochemical tests: sex specific cut-off values to obtain equal sensitivity for colorectal cancer? *BMC Gastroenterology* 2014;14(1):217. doi: 10.1186/s12876-014-0217-7

66. Xu H, Chen H, Hu J, et al. Feasibility of quantification based on novel evaluation with stool DNA and fecal immunochemical test for colorectal cancer detection. *BMC Gastroenterology* 2022a;22(1):384.

67. Xu J, Rong L, Gu F, et al. Asia-Pacific Colorectal Screening Score Combined With Stool DNA Test Improves the Detection Rate for Colorectal Advanced Neoplasms. *Clinical Gastroenterology & Hepatology* 2022b;14:14.

68. Young GP, Woodman RJ, Symonds E. Detection of advanced colorectal neoplasia and relative colonoscopy workloads using quantitative faecal immunochemical tests: an observational study exploring the effects of simultaneous adjustment of both sample number and test positivity threshold. *BMJ Open Gastroenterology* 2020;7(1):09.

69. Zhao S, Wang S, Pan P, et al. FIT-based risk-stratification model effectively screens colorectal neoplasia and early-onset colorectal cancer in Chinese population: a nationwide multicenter prospective study. *Journal of hematology & oncology* 2022;15(1):162.

70. Navarro M, Omella I, Carrera P, et al. Fecal hemoglobin concentration, a good predictor of risk of advanced colorectal neoplasia in symptomatic and asymtomatic patients. *Gastroenterology* 2017;152(5 Supplement 1):S548.

71. Schwettmann L, Lied A, Eriksen R. Evaluation of the Sentinel-FOB gold faecal immunochemical test for the presence of haemoglobin using the automated Roche Cobas 8000 system. *Practical Laboratory Medicine* 2022;29:e00263.

72. Calanzani N, Pannebakker MM, Tagg MJ, et al. Who are the patients being offered the faecal immunochemical test in routine English general practice, and for what symptoms? A prospective descriptive study. *BMJ Open* 2022;12(9):e066051.

73. Carroll MRR, John C, Mantio D, et al. An assessment of the effect of haemoglobin variants on detection by faecal immunochemical tests. *Annals of Clinical Biochemistry* 2018;55(6):706-09. doi: <https://dx.doi.org/10.1177/0004563218778716>

74. Chen CC, Chang PY, Chang YS, et al. MicroRNA-based signature for diagnosis and prognosis of colorectal cancer using residuum of fecal immunochemical test. *Biomedical Journal* 2022;22:22.

75. Fernandez de Castro JD, Baiocchi Ureta F, Fernandez Gonzalez R, et al. Faecal Immunochemical Test Impact on Prognosis of Colorectal Cancer Detected in Symptomatic Patients. *Diagnostics* 2022;12(4):17.

76. Hunt N, Allcock R, Myers M. Faecal immunochemical testing (FIT) for colorectal cancer in symptomatic primary care patients. Clinica Chimica Acta, 2019.

77. Lee J-M, Park MJ, Heo W, et al. Clinical Utility of Fecal Immunochemical Transferrin Test in Gastrointestinal Bleeding Detection. *acm* 2018;21(3):51-57. doi: 10.5145/ACM.2018.21.3.51

78. Maria Theresa R, Matthew R, Richard MM, et al. Rapid diagnostic pathways for suspected colorectal cancer: views of primary and secondary care clinicians on challenges and their potential solutions. *BMJ Open* 2015;5(10):e008577. doi: 10.1136/bmjopen-2015-008577

79. Navarro M, Hijos G, Ramirez T, et al. Fecal Hemoglobin Concentration, a Good Predictor of Risk of Advanced Colorectal Neoplasia in Symptomatic and Asymptomatic Patients. *Front Med (Lausanne)* 2019;6:91. doi: 10.3389/fmed.2019.00091 [published Online First: 2019/05/28]

80. Niedermaier T, Alwers E, Chen X, et al. A single measurement of fecal hemoglobin concentration outperforms polygenic risk score in colorectal cancer risk assessment. *medRxiv* 2022;22 doi: <https://dx.doi.org/10.1101/2022.07.22.22277924>

81. Wilson N, Baker-Beal L, Kyaw WW, et al. Real world experience of faecal immunochemical testing (FIT) in uk primary care to support the referral and diagnosis of colorectal cancer (CRC). *United European Gastroenterology Journal* 2020;8(8 SUPPL):579-80. doi: <https://dx.doi.org/10.1177/2050640620927345>

82. Use of fecal immunochemical test in symptomatic patients. WEO The VVoice of World Endoscopy; 2022.

83. Krivec. ASSESSMENT OF THE DIAGNOSTIC APPLICABILITY OF QUANTITATIVE IMMUNOCHEMICAL FAECAL OCCULT BLOOD TESTS. 2011

84. Sandhu K, Naik S, Ayling RM. Use of faecal immunochemical testing as an alternative to faecal calprotectin in children. *Ann Clin Biochem* 2021;58(3):230-35. doi: 10.1177/0004563221989359 [published Online First: 2021/01/09]

85. Zacharopoulou L, Cama R, Kapoor N, et al. PTH-99 Faecal Immunochemical Testsfor younger patients presenting with bowel symptoms. *Gut* 2021;70(Suppl 4):A162-A62. doi: 10.1136/gutjnl-2021-BSG.302

86. Zhu M, Fan L, Han M, et al. The usefulness of fecal hemoglobin and calprotectin tests in diagnosing significant bowel diseases: a prospective study. *Scandinavian Journal of Gastroenterology* 2022:1-7.

87. Rodriguez-Alonso L, Rodriguez-Moranta F, Maisterra S, et al. The EPAGE guidelines are not an effective strategy for managing colonoscopies during the COVID-19 pandemic. *Gastroenterologia y Hepatologia* 2022;45(1):9-17.

88. Almoneef NM, Alkhenizan AH, Mahmoud AS, et al. The yield of fecal occult blood testing as a screening tool for colon cancer in a primary care setting. *Journal of Family Medicine & Primary Care* 2022;11(8):4435-39.

89. Cubiella J, Digby J, Rodríguez-Alonso L, et al. The fecal hemoglobin concentration, age and sex test score: Development and external validation of a simple prediction tool for colorectal cancer detection in symptomatic patients. *Int J Cancer* 2017;140(10):2201-11. doi: 10.1002/ijc.30639 [published Online First: 2017/02/12]

90. Cubiella J, Salve M, Díaz-Ondina M, et al. Diagnostic accuracy of the faecal immunochemical test for colorectal cancer in symptomatic patients: comparison with NICE and SIGN referral criteria. *Colorectal Dis* 2014;16(8):O273-82. doi: 10.1111/codi.12569 [published Online First: 2014/01/25]

91. Khan AA, Klimovskij M, Harshen R. Accuracy of faecal immunochemical testing in patients with symptomatic colorectal cancer. *Bjs Open* 2020;4(6)

92. Maclean W, Benton SC, Whyte MB, et al. Efficacy and accuracy of faecal sampling by a digital rectal examination for FIT. *Annals of Clinical Biochemistry* 2023:00045632231155021.

93. McDonald P, Digby J, Innes C, et al. Low faecal haemoglobin concentration potentially rules out significant colorectal disease. *Colorectal Disease* 2013;15(3):e151-e59.

94. County RÖ, Härjedalen RJ, Östergötland R, et al. Accuracy and Predictive Values for Colorectal Cancer of Quantitative FIT in Symptomatic Patients in Primary Care: <https://ClinicalTrials.gov/show/NCT05156307>, 2021.

95. Ghada Mohamed ABJS. Sensitivity and specificity of stool markers in Inflammatory bowel disease: A systematic review and meta-analysis.

96. Jen-Hao Yeh W-LW. Efficacy of fecal immunochemical test in young patients (aged ≤ 50 years).

97. Jennifer Pham ESMWJWGL-L. The diagnostic accuracy of faecal immunochemical tests for detecting colorectal cancer and pre-cancerous neoplasia in patients with iron deficiency with or without anaemia.

98. Loov A, Hogberg C, Lilja M, et al. Diagnostic accuracy for colorectal cancer of a quantitative faecal immunochemical test in symptomatic primary care patients: a study protocol. *Diagnostic and Prognostic Research* 2022;6(1):16.

99. Tian Zhi Lim JLAUJLGW. Uncovering the barriers and facilitators towards undergoing follow-up colonoscopy among individuals with FIT positive results: A systematic review.

100. Tian Zhi Lim JLJLGW. What can we do to improve compliance to follow-up colonoscopy among individuals with FIT positive results? A systematic review.

101. University M. FIT and Fecal Calprotectin in Patients With Chronic Lower GI Symptoms: <https://ClinicalTrials.gov/show/NCT05514561>, 2020.

102. Winnie Poulsen MTETWX. Factors associated with the quality of faecal occult blood test (FOBT) for early detection of colorectal cancer: a systematic review and meta-analysis protocol.

103. Zhen Junhai CB. Value of Faecal immunochemical tests in significant bowel disease screening among patients with lower bowel symptoms in primary care&#xff1a;a meta analysis.

104. Booth R, Carten R, D'Souza N, et al. Role of the faecal immunochemical test in patients with risk-stratified suspected colorectal cancer symptoms: A systematic review and meta-analysis to inform the ACPGBI/BSG guidelines. *The Lancet Regional Health Europe* 2022;23:100518.

105. Farkas NG, Fraser CG, Maclean W, et al. Replicate and repeat faecal immunochemical tests in symptomatic patients: A systematic review. *Annals of Clinical Biochemistry* 2022:45632221096036.

106. Jung YS, Im E, Park CH. Impact of antiplatelet agents and anticoagulants on the performance of fecal immunochemical tests: a systematic review and meta-analysis. *Surgical Endoscopy* 2022;36(6):4299-311.

107. Monahan KJ, Davies MM, Abulafi M, et al. Faecal immunochemical testing (FIT) in patients with signs or symptoms of suspected colorectal cancer (CRC): a joint guideline from the Association of Coloproctology of Great Britain and Ireland (ACPGBI) and the British Society of Gastroenterology (BSG). *Gut* 2022;12:12.

108. Nasir Kansestani A, Zare ME, Tong Q, et al. Comparison of faecal protein biomarkers' diagnostic accuracy for colorectal advanced neoplasms: a systematic review and meta-analysis. *Scientific Reports* 2022;12(1):2623.

109. Pang SJ, Lin ZP, Sun Z, et al. Impact of antithrombotic drugs on the accuracy of fecal occult blood testing for advanced colorectal neoplasia screening: a meta-analysis and systematic review. *Zeitschrift fur Gastroenterologie* 2022;17:17.

110. Pin Vieito N, Zarraquinos S, Cubiella J. High-risk symptoms and quantitative faecal immunochemical test accuracy: Systematic review and meta-analysis. *World Journal of Gastroenterology* 2019;25(19):2383-401.

111. Pin-Vieito N, Tejido-Sandoval C, de Vicente-Bielza N, et al. Faecal immunochemical tests safely enhance rational use of resources during the assessment of suspected symptomatic colorectal cancer in primary care: systematic review and meta-analysis. *Gut* 2022;71(5):950-60.

112. Quyn AJ, Steele RJC, Digby J, et al. Application of NICE guideline NG12 to the initial assessment of patients with lower gastrointestinal symptoms: not FIT for purpose? *Annals of Clinical Biochemistry* 2018;55(1):69-76. doi: <https://dx.doi.org/10.1177/0004563217707981>

113. Saw KS, Liu C, Xu W, et al. Faecal immunochemical test to triage patients with possible colorectal cancer symptoms: meta-analysis. *British Journal of Surgery* 2022;109(2):182-90.

114. Westwood M, Corro Ramos I, Lang S, et al. Faecal immunochemical tests to triage patients with lower abdominal symptoms for suspected colorectal cancer referrals in primary care: a systematic review and cost-effectiveness analysis. *Health Technology Assessment (Winchester, England)* 2017;21(33):1-234.

115. Westwood M, Lang S, Armstrong N, et al. Faecal immunochemical tests (FIT) can help to rule out colorectal cancer in patients presenting in primary care with lower abdominal symptoms: a systematic review conducted to inform new NICE DG30 diagnostic guidance. *BMC Medicine* 2017;15(1):189.

116. Zou J, Xiao Z, Wu Y, et al. Noninvasive fecal testing for colorectal cancer. *Clinica Chimica Acta* 2022;524:123-31.

117. Cilona A, Zullo A, Hassan C, et al. Is faecal-immunochemical test useful in patients with iron deficiency anaemia and without overt bleeding? *Digestive and Liver Disease* 2011;43(12):1022-24. doi: <https://doi.org/10.1016/j.dld.2011.08.002>

118. Díaz O, Blanco V, Ceballos O, et al. CLINICAL OR ANALYTICAL CRITERIA FOR COLORECTAL CANCER (CRC) DETECTION IN SYMPTOMATIC PATIENTS? A DIAGNOSTIC TESTS STUDY. IFCC WorldLab. Istanbul, 2014.
