# Supplementary material for: Faecal immunochemical tests for patients with symptoms suggestive of colorectal cancer: An updated systematic review and multiple‐threshold meta‐analysis of diagnostic test accuracy studies
Source: Colorectal Dis. 2024 Dec 17;27(1):e17255. doi: 10.1111/codi.17255 (PMC11683176; doi:10.1111/codi.17255)
Supplement: Supplementary file 7 — Data S7. [file CODI-27-0-s016.docx]

**Main analyses study and patient characteristics**

This supplement contains studies that contributed to the main analyses for each test (Tables 1 - 6. Column 1 (study ID) contains a unique number for each study, as some studies contributed to multiple analyses and were published across multiple publications.

**Table 1: Study and patient characteristics of HM-JACKarc studies**

| **^ID^** | **Author, year**  **Location**  **Recruitment dates**  **Study name (if available)** | **Analyser**  **Reference standard** | **Inclusion criteria** | **Comparison to NICE scope** | **Mean/median age in years** | **Patient characteristics**   - Male; - Ethnicity;   Anaemia status | **N with CRC/ N analysed (%)** | **Thresholds µg/g** | **Subgroup data?** |
| --- | --- | --- | --- | --- | --- | --- | --- | --- | --- |
| **Population type 1 studies (all patient presenting to primary care with symptoms meeting NG12 high/medium or DG30 low-risk)** | | | | | | | | | |
| 1 | D'Souza 2020a^1^  Croyden, UK  Nov 2016 to Oct 2017 | HM JACKarc analytical system  Colonoscopy | All NG12 and DG30 – all symptomatic patients were referred to colonoscopy in this period in this area of London | NR | mean 60.6 (range 20–90) | - 48.6% - Ethnicity reported^a^ - NR | 12/298 (4.03%) | 2, 10 | None |
| 2 | Gerrard 2023^2^  Lothian, Scotland, UK  Jan 2019 to Feb 2020 | HM-JACKarc  Endoscopy or CT with colorectal protocol. | Urgent suspected of cancer referrals, criteria for referral^a^ are both wider and narrower than NG12high/medium and DG30 low-risk | - Wider and narrower than target population^a^ - Abdominal mass: 3.0% - rectal mass: 2.4% | Median 65 (IQR 56-74) | - 44.3% - NR - 17.8% | 135/3426 (3.05%) | 10 | Anaemia, no anaemia |
| 3 | Johnstone 2022a^3^  Greater Glasgow and Clyde, Scotland, UK  August 2018 to January 2019 | HM-JACKarc (personal communication)  Records follow-up | All with NG12 high/medium or DG30 low-risk would get FIT (confirmed by author via personal communication) | May be wider  Abdominal mass 2.5%, rectal mass 0.9% | Median 59 (range 16 to 97), n=4968 | 42.3%  NR  IDA 5.4%^a^; Anaemia 20.0% | 61/4737 (1.29%) | 10, 150, 400 | Anaemia, no anaemia |
| 4 | MacDonald 2022^4^  NHS Lanarkshire, Scotland, UK  October 2016 to February 2019 | HM-JACKarc  Records follow-up | Symptomatic colorectal referrals from primary care, under SIGN 126 and Scottish Referral Guidelines which encompass both NG high risk and DG30 low-risk for referral | Includes anorectal or abdominal mass; also includes referrals based on imaging, but from GP care. | Median 62 (range 16–96  years) | 45.7%  NR  NR | 151/5250 (2.88%) | 10 | None |
| 5 | Mowat 2021^5^ & 2019^6^  NHS Tayside, Scotland, UK  December 2015 to December 2016 | HM JACKarc  Records follow-up | GPs encouraged to use FIT in patients regardless of the specific lower GI symptoms and perceived risk | NR | Median 65 (range: 2–99, IQR: 51–75)^6^ | 43.6%^6^  NR  NR | 105/5381 (1.95%) | 2, 7, 10, 20, 50, 100, 150, 200, 250, 300, 350, 400 | None |
| **Population type 2 studies (NG12 High risk)** | | | | | | | | | |
| 1 | D'Souza 2020a^1^  Croyden, UK  Nov 2016 to Oct 2017 | HM JACKarc analytical system  Colonoscopy | NG12 High/medium-risk (subgroup of main Croydon study) who underwent colonoscopy | NR | NR for subgroup |  | 8/160 (5.00%) | 2, 10 | None |
| 6 | D'Souza 2021a^7^  D'Souza 2021c^8d^  NICE FIT  October 2017 to December 2019 | HM JACKarc analytical system  Colonoscopy | Subgroup: NG12 High/medium-risk  Full study: 2WW patients (including NG12, DG30, others) who underwent colonoscopy | NR | NG12 high/medium-risk: Mean 65.9 (SD 11.1)  Full study: 64.0 (SD 11.9) | NG12 high/medium-risk:   - 45.7% - Ethnicity reported^a^ - IDA 4.2%   Full study:   - 45.1 - Ethnicity reported^a^ - NR | NG12 high risk:  257/7194 (3.57%)  Full study:  421/9822 (4.29%) | 2, 10, 150 | None |
| 7 | Farrugia 2020^9^  University Hospitals Coventry and Warwickshire NHS Trust, UK  January 2015 to March 2017 | HM JACKarc automated system  Colonoscopy or CT colonography and histology results | NG12 High/med risk^a^ | Abdominal/rectal mass n=10 | 68.6 (error/range NR) | - 48.9% - NR - Anaemia, including iron deficiency 18.1% | 10/519 (6.36%) | 10 | None |
| 8 | Turvill 2018^10^  York Hospital, UK  February 2016 to March 2017 | HM-JACKarc  Full colonoscopy or CT colonography or a lesser investigation (such as CT abdomen/ pelvis with contrast plus flexible sigmoidoscopy) limited by the identification of pathology | NG12 High/medium-risk | 4% abdominal mass and 1% rectal mass | median 69 (IQR 61-76) | - 50% - NR - 18% IDA - ^a^ | 27/505 (5.35%) | 12 | None |
| **Population type 3 (DG30 low-risk)** | | | | | | | | | |
| 1 | D'Souza 2020a^1^  Croyden, UK  Nov 2016 to Oct 2017 | HM JACKarc analytical system  Colonoscopy | DG30 low-risk (subgroup of main Croydon study) who underwent colonoscopy | NR for subgroup | NR for subgroup |  | 2/138 (1.45%) | 2, 10 |  |
| 9 | Withrow 2022^11^ (same study as Nicholson 2020)^12a^  Oxfordshire, UK  March 2017 to December 21, 2020  CSS-BIO-3 4730 | HM JACKarc  Records follow-up | **Type 3 subgroup from type 4 study** – FIT given in primary care for any reason, wider than DG30 low-risk alone | NR | Median 61 (IQR 51 to 75)^a^ | - 42% - NR - Any anaemia: 26%; IDA: 11%^a^ | 139/16604 (0.84%) | 2, 10 | DG30 only subgroup; various anaemia thresholds (men/ women separately); men; women; age </>40, >50, >60, >70, >80 |
| **Population type 4 (unclear/unrepresentative of all presenting to primary care)** | | | | | | | | | |
| 11 | Chapman 2021^13^  Nottingham University Hospitals Trust, UK  Sept 2016 to Sept 2017 | HM JACKarc + HM JACKarc analyser  2WW investigations | 2WW patients who returned 2 types of FIT test | NR | median 71.1 (IQR 62.5-78.7) |  | 38/732 (5.19%) | 4, 10, 22.6, 150 | None |
| 12 | Elbeltagi 2022^14^  North Yorkshire, UK  March to October 2020 | HM-JACKarc (personal communication)  Colonoscopy or cross-sectional imaging | 2WW patients | NR | Median72 (IQR: 63-78) | - NR - NR - NR | 52/992 (5.24%) | 29 thresholds between 6 and 401 at varying intervals | None |
| 13 | Faux 2022^15^  Cornwall, UK  March to July 2020 | HM-JACKarc  Colonoscopy or CT abdomen/pelvis, or CT thorax/abdomen/pelvis | 2WW patients | Palpable mass 0%  anal ulceration NR | NR | - NR - NR - NR | 6/175 (3.43%) | 10 | None |
| 14 | Godber 2016^16^  NHS Lanarkshire, Scotland, UK  June 2013 to December 2013 | HM JACKarc analyser  Colonoscopy | Referred to colonoscopy in Scotland, 2013, referral criteria unclear | NR | median 59 (range 16–89), n=507 | - 216/507 (42.6%) - NR - 23/484 (4.8%) | 11/484 (2.27%) | 10 | None |
| 10 | Nicholson (2018)^17^  Oxfordshire, UK  Jan to March 2016  CSS-BIO-3 4730 | HM JACKarc (NB some pts had two test results, any positive was a positive)  Records follow-up | Same criteria as DG30 low-risk, but unknown proportion outside the criteria | NR | Median 58,  range 19–93 years | - 43% - NR - n=62 (denominator unclear) | 7/238 (2.94%) | 7, 10, 20, 50 | None |
| 9 | Nicholson 2020^12^ (overlaps with Withrow 2022)^11^  Oxfordshire, UK  March 2017 and March 2020  CSS-BIO-3 4730 | HM JACKarc  Records follow-up | Same criteria as DG30 low-risk, plus some outside the criteria (e.g., Inflammation; thrombocytosis; tired all the time) | palpable rectal or anal mass, or anal ulceration n=0 | median 60 (range 18-101, IQR 51-74) | - 41.4% - NR - Anameia n=2791/12509 = 22.3%; Iron deficiency n=1158/12509= 9.3% | 105/9896 (1.06%) | 7, 10, 20, 50, 100, 120, 150 | Males; females |
| 15 | Tang 2022^18^  Wales, UK  March to June 2020 | HM-JACKarc system  colonoscopy or CTC, or MPCT (minimal preparation CT) | All consecutive patients referred from primary care on the USC pathway^a^ | Abdominal mass 2.5%  Anal lump/mass 2.2%  Rectal mass 1.5% | median 68 (range 21–97) (n=1050) | - 47.4% - NR - new anaemia 11.1% | 20/603 (3.32%) | 10 | IDA |
| 16 | Turvill 2021^19^  Yorkshire & Humber, UK  April 2018 to Dec 2019  Fast track FIT | HM JACKarc  Full colonoscopy or CT colonography, or a lesser investigation (such as CT abdomen/pelvis with contrast or flexible sigmoidoscopy) | 2WW patients | Abdominal mass 1.7%  Rectal mass 1.6% | Mean 67.3 (SD 11.7)  Median 69 (IQR 60, 76) | - 44.5% - NR - IDA or other anaemia, 21.9% - ^a^ | 151/5040 (3.00%) | 2 | Anaemia, no anaemia, Males, females, medication (antiplatelets, anticoagulants NSAIDs), age </>60 |

2WW, two week wait; CT, computed tomography; DG30, Diagnostic Guidance 30; FIT, faecal immunochemical test; GG&C, Greater Glasgow & Clyde; GI, gastrointestinal; ID, study identification number; IDA, iron-deficiency anaemia; IQR, interquartile range; NB, note bene; NHS, National Health ServiceNG12, NICE national guideline 12; NR, not reported; SD, standard deviation

^a^**Farrugia 2020:** study recruited all 2WW patients, and reported DG30 low-risk and NG12 high/medium-risk subgroups. Only the NG12 subgroup has been included in the analysis since the DG30 subgroup is likely to be highly selected and/or enriched with FIT or Guaiac positive patients; **Gerrard 2023:** Inclusion criteria were urgent suspected of cancer or urgent priority referrals with ‘high-risk’ symptoms: repeated rectal bleeding without obvious rectal cause or blood mixed in stool, persistent change in bowel habit, palpable abdominal or rectal mass, weight loss and/or abdominal pain with or without unexplained iron deficiency anaemia (IDA); **Johnstone 2022a:** IDA defined as ferritin <15 µg/L; MacDonald 2022: Personal communication with the author indicated that SIGN 126 guidelines and the Scottish Referral Guidelines for Suspected Cancer were used to guide referrals, and that these indicate that both high and low-risk patients as defined by NG12 should be referred. The paper itself lists: “rectal bleeding, diarrhoea, anaemia, anorectal or abdominal mass, abdominal pain weight loss, change in bowel habit (including faecal incontinence), anorectal symptoms (tenesmus, per rectal pain or mucous) or colorectal abnormalities on imaging, per NHSL pre-existing criteria”; **Tang 2022;** referral criteria in Wales unclear; **Turvill 2021:** additional patient characteristic reported, 27% using antiplatelet therapy, anticoagulants or NSAIDs**; Turvill** **2018:** additional patient characteristic reported, 30% were taking NSAID, antiplatelet therapy or anticoagulants; **Withrow 2022:** Nicholson 2020 was selected for inclusion in the main analysis despite having fewer patients than Withrow 2022 since it reported more thresholds; **D’Souza 2020a**: white (62%), Asian (14%),‘other’ ethnicity (12%), black (9%) **D’Souza 2021a:** White 5693 (80.0%), Asian 355 (5.0), Black 253 (3.6), Mixed 42 (0.6), Chinese 27 (0.4), Not specified 746 (10.5); ^b^ Some type 1-4 studies also report subgroup data as indicated in final column.

^c^The full study population was included in the AA and IBD data analysis as this was not reported for the NG12 subgroup

**Table 2 : Study and patient characteristics of OC-Sensor studies**

| **#** | **Author, year**  **Location**  **Recruitment dates**  **Study name (if available)** | **Analyser**  **Reference standard** | | **Inclusion criteria** | | | **Comparison to Scope** | | **Mean/median age in years** | **Patient characteristics**   - Male; - Ethnicity; - Anaemia status^f^ | | | **N with CRC/ N analysed (%)** | **Thresholds, µg/g** | | **Subgroups^e^** |
| --- | --- | --- | --- | --- | --- | --- | --- | --- | --- | --- | --- | --- | --- | --- | --- | --- |
| **Population type 1 studies (all patient presenting to primary care with symptoms meeting NG12 high/medium or DG30 low-risk)** | | | | | | | | | | | | | | | | |
| **17** | **Crooks 2023^20^**  **(earlier data cut reported in Bailey 2021a^21^**  **Bailey *in press^22^*)**  Nottingham, UK  Nov 2017 to Nov 2021 | - iO - Records follow-up | | All referral criteria, except anorectal mass. | | | Anorectal mass excluded | | NR | - NR - NR - NR | | | 514/37216 (1.38%) | 4, 10, 20, 40, 100 | | 0 |
| **18** | **Cama 2022^23^**  Hertfordshire, UK  June 2019 to Nov 2021 | - iO - Records follow-up | All DG30 (low-risk) and most NG12 high risk (see column 6) | | | | IDA, rectal bleeding, rectal or abdominal masses excluded | | Median 61 (IQR 55–77) | - 43% (of n=12,231) - NR - 2% IDA; nonIDA 4% | | 74/5341 (1.39%) | | 4, 10, 100 | 0 | |
| **19** | **Georgiou Delisle 2022**  **^24^**  Croyden, UK  Dec 2019 to Oct 2020 | - iO - Records follow up | | | NICE NG12 and DG30 criteria | Rectal mass or anal ulceration referred straight to 2WW | | Mean 65 (range 18–99) | | | - Male 44.8% - See footnotes* - NR | | 61/4187 (1.46%) | 4, 10, 150 | 0 | |

| **Population type 2 studies (NG12 High risk)** | | | | | | | | | |
| --- | --- | --- | --- | --- | --- | --- | --- | --- | --- |
| **4** | **Benton 2022^25^**  50 NHS hospitals across England, UK  Oct 2017 to Dec 2019  NICE FIT | - OC Sensor PLEDIA - Colonoscopy | NG12 high risk, who had colonoscopy. Randomised to cohort 1 who were given 4 tests | NR | NR | - NR - NR - NR | 7/233 (3.00%) | 1, 10, 100 | 0 |
| **Population type 3 (DG30 low-risk)** | | | | | | | | | |
| **20** | **Ball 2022^26^ (additional data by personal communication)**  Sheffield, UK  Oct 2019 to Dec 2019 | - PLEDIA - Colonoscopy or CT imaging^a^ and colon capsule endoscopy | DG30 low-risk^a^ | NR | NR for this subgroup | - NR for this subgroup - NR - NR for this subgroup | 17/2892 (0.6%) | 10, 20, 50, 80, 100, 120, 150 | Males; females |
| **Population type 4 (unclear/unrepresentative of all presenting to primary care)** | | | | | | | | | |
| **21** | **Archer 2022^27^**  Sheffield, UK  March 2020 to July 2020 | - PLEDIA - CT, colonoscopy | 2WW patients | NR | n=514 Mean 64.5 years (SD 12.7 yrs) | n=514   - 50% - NR - n=514 IDA (23%) | 11/166 (6.62%) | 10, 60, 100 | 0 |
| **7** | **Chapman 2021^13^**  Nottingham, UK  September 2016 to September 2017  Getting FIT | - DIANA - Colonoscopy and additional investigations (e.g., radiology) | 2WW patients, returning both FIT tests | NR | median 71.1 (IQR 62.5-78.7) | - 43.9% - NR - NR | 38/732 (5.19%) | 4, 10, 100 | 0 |
| **22** | **Juul 2018^28^**  Central Denmark  Sept 2015 to Aug 2016  NCT02308384 | - DIANA - Records follow-up | Patients with “non-alarm” symptoms of CRC^a^ | NR | Mean NR | - 43.9% - See footnote^a^ - 12.3% | 54/3462 (1.56%) | 10 | Unexplained anaemia |
| **23** | **Laszlo 2021^29^**  24 hospitals and 59  GP practices in UK  April 2017 to March 2019 | - iO - Colonoscopy 77.7% CTC 14.2% Flexi sig 7.5 | 2WW patients | NR | Median 67 (range 19–99; IQR 57–75)) | - 46.6% - See footnote^a^ - 19% | 90/3596 (2.50%) | 4, 6, 10, 20, 50, 80, 100, 120, 150, 200 | 0 |
| **24** | **Maclean 2021a^30^**  Royal Surrey NHS Foundation Trust (RSFT), UK  End of March 2020 to July 2020 | - PLEDIA - Assume records follow-up, as some patients were safety netted | 2WW patients | NR | NR | - NR - NR - NR | 12/358 (3.35%) | 10, 150 | 0 |
| **25** | **Mowat 2016^31^**  NHS Tayside, Scotland, UK  Oct 2013 to March 2014 | - iO - Colonoscopy | Symptomatic patients referred from primary care with FIT | Palpable mass 0.3% | Median 64  (range 16–90, IQR 52–73)  (n=755) | (n=755)   - 45.3% - NR - 9% | 28/750 (3.73%) | 4, 10 | 0 |
| **26** | **Pin Vieito 2021^32^**  San Sebastian, Spain  Jan 2012 to Dec 2016 | - NR - Records follow-up | Patients referred from primary care with FIT | NR | NR | - NR - NR - NR | 73/4543 (1.61%) | 10, 20 | 0 |

2WW, two week wait; CT, computed tomography; DG30, Diagnostic Guidance 30; FIT, faecal immunochemical test; GI, gastrointestinal; ID, study identification number; IDA, iron-deficiency anaemia; IQR, interquartile range; NHS, National Health Service; NG12, NICE national guideline 12; NR, not reported; SD, standard deviation

^a^**Ball 2022**: data was also available for all patients on the 2WW, the subgroup of patients who meet NG12 criteria were selected for inclusion in the review. CT imaging was a mix of CTC and other CT imaging modalities; **Juul 2018**: FIT test aimed at those ≥30 years with non-alarm symptoms of CRC, according to GP clinical knowledge and instructions which included: change in bowel habits, abdominal pain, unexplained anaemia, and unspecific symptoms (e.g. fatigue or weight loss), but not for IBS workup. Those aged ≥40 years with rectal bleeding, change in bowel habits >4 weeks, abdominal pain and iron deficiency anaemia recommended to be referred straight to secondary care; **Georgiou Delisle 2022:** White/white British 51.4%, Asian/Asian British 12.6%, Black/Black British 14.8%, Chinese 0.8%, Other 18.6%, mixed 1.5%, Not recorded 0.4%; **Juul 2018**: n (%); Danish: 3280 (94.8), Immigrant Western country: 84 (2.4), Immigrant non-western country: 98 (2.8); **Laszlo 2021**: Black/black British 4.5, Asian/Asian British 6.1, Other Asian 2.0, White 23.5, British mixed 17.9, Multiple/other 5.6, Missing data 40.3

**^b^** Some type 1-4 studies also report subgroup data as indicated in final column

^d^ IDA defined as Hb<11.9 g/dL in men and Hb<10.9 g/dL in women, and ferritin ≤ 30 g/dL

^e^ 0 – None; 1, IDA or Anaemia; 2, M; 3, F; 4, Ethnicity; 5, Medications which may affect GI bleeding; 6, Blood disorders which may affect the performance of the test; 7, age groups (add age)

^f^ Study characteristics relating to medications that may cause GI bleeding and conditions that may affect FIT have been removed, since this data was not reported for any studies.

**Table 3:** **Study and patient characteristics of FOB-Gold studies**

|  | **Author, year**  **Location**  **Recruitment dates**  **Study name (if available)** | **Analyser**  **Reference standard** | **Inclusion criteria** | **Comparison to Scope** | **Mean/median age in years** | **Patient characteristics**   - Male; - Ethnicity; - Anaemia status | **N with CRC/ N analysed (%)** | **Thresholds, µg/g** | **Subgroups** |
| --- | --- | --- | --- | --- | --- | --- | --- | --- | --- |
| **Population type 2 studies (NG12 High risk)** | | | | | | | | | |
| 6 | Benton 2022^25^  50 NHS hospitals across England, UK  Oct 2017 to Dec 2019  NICE FIT | FOB Gold Wide - SENTiFIT 270  Colonoscopy | NG12 high risk, who had colonoscopy. Randomised to cohort 1 who were given 4 tests | NR | NR | - NR - NR - NR | 7/233 (3.00%) | 2, 10, 100 | None |

| **Population type 4 (unclear/unrepresentative of all presenting to primary care)** | | | | | | | | | |
| --- | --- | --- | --- | --- | --- | --- | --- | --- | --- |
| 27 | MacLean 2022a^33^  Royal Surrey Foundation Trust, UK  July 2019 and March 2020 | FOB Gold Wide SENTiFIT 270  Colonoscopy or CTC or flexisig^a^ | 2WW referrals | NR | NR | - 48.8% - NR - NR | 14/553 (2.53%) | 10, 100, 150 | None |
| 28 | Jordaan 2023^34^  Mid-Yorkshire NHS Trust, Wakefield, UK  Sept 2018 to the Dec 2019 | FOB Gold, Roche Cobas c501 analyser  Records follow-up | Mainly DG30 low risk, but some NG12 high risk | NR | NR | - 48% - NR - NR | 30/3349 (0.90%) | 10 | None |

2WW, two week wait; CTC, computed tomography colonoscopy; DG30, Diagnostic Guidance 30; ID, study identification number; NHS, National Health Service; NG12, NICE national guideline 12; NR, not reported

^a^ **Maclean 2022a:** flexisig if presenting with perianal symptoms or anorectal bleeding

**Table 4: Study and patient characteristics and diagnostic test accuracy of QuikRead go studies**

| **#** | **Author, year**  **Location**  **Recruitment dates**  **Study name (if available)** | **Analyser**  **Reference standard** | **Inclusion criteria** | **Patient characteristics**   - Mean age in years - Male; - Ethnicity; - Anaemia status | **N with CRC/ N analysed (%)** | **Thresholds, µg/g** | **Sensitivity (95% CI)** | **Specificity (95% CI)** |
| --- | --- | --- | --- | --- | --- | --- | --- | --- |
| **Population type 2 studies (NG12 High risk)** | | | | | | | | |
| 27 | MacLean 2021b  Royal Surrey Foundation Trust, UK  July 2019 and March 2020 | QuikRead go  Colonoscopy, CTC or flexisig | 2WW NG12 high/medium-risk^a^ | - Mean age NR - 49.9% - NR - All anaemia: 12.8%; iron or ferritin deficient anaemia: 4.5% | 14/553 (2.53%) | 10 | 92.90 (68.5 - 98.7) | 70.10 (66.1 - 73.8) |
|  |  |  |  |  |  | 100 | 71.40 (45.4 - 88.3) | 94.60 (92.4 - 96.2) |
|  |  |  |  |  |  | 150 | 57.10 (32.6 - 78.6) | 95.90 (93.9 - 97.3) |

2WW, two week wait; CTC, computed tomography colonoscopy; DG30, Diagnostic Guidance 30; ID, study identification number; NG12, NICE national guideline 12; NR, not reported

^a^ **Maclean 2021b:** population confirmed with author, FIT was not being used by GPs during recruitment period so only NG12 high/medium-risk were referred

**Table 5: Study and patient characteristics and diagnostic test accuracy of the NS-Prime study**

| **#** | **Author, year**  **Location**  **Recruitment dates**  **Study name (if available)** | **Analyser**  **Reference standard** | **Inclusion criteria** | **Patient characteristics**   - Mean age in years - Male; - Ethnicity; - Anaemia status | **N with CRC/ N analysed (%)** | **Threshold, µg/g** | **Sensitivity (95% CI)** | **Specificity (95% CI)** |
| --- | --- | --- | --- | --- | --- | --- | --- | --- |
|  | **Population type 2 studies (NG12 High risk)** |  |  |  |  |  |  |  |
| 4 | Benton 2022^25^  50 NHS hospitals across England, UK  Oct 2017 to Dec 2019  NICE FIT | NS-Prime  colonoscopy | NG12 high risk, who had colonoscopy. Randomised to cohort 1 who were given 4 tests | - NR - NR - NR - NR | 7/233 (3.00%) | 3 | 85.70 (48.7–97.4) | 31.90 (26.1–38.2) |
|  |  |  |  |  |  | 10 | 71.40 (35.9–91.8) | 83.60 (78.2–87.9) |
|  |  |  |  |  |  | 100 | 57.1025.1–84.2) | 97.30 (94.3–98.8) |

2WW, two week wait; CTC, computed tomography colonoscopy; DG30, Diagnostic Guidance 30; ID, study identification number; NHS, National Health Service; NG12, NICE national guideline 12; NR, not reported

**Table 6: Study and patient characteristics of IDK Hemoglobin (human) and hemoglobin/haptoglobin complex ELISA tests**

| **#** | **Author, year**  **Location**  **Recruitment dates**  **Study name (if available)** | **Analyser**  **Reference standard** | **Inclusion criteria** | **Patient characteristics**   - Mean age in years - Male; - Ethnicity; - Anaemia status | **N with CRC/ N analysed (%)** | **Thresholds, µg/g** | **Sensitivity (95% CI)** | **Specificity (95% CI)** |
| --- | --- | --- | --- | --- | --- | --- | --- | --- |
| **Population type 4 studies (unclear/unrepresentative of patients presenting to primary care):** | | | | | | | | |
| 28 | Sieg 1999^35^  Ostringen, Germany  NR, prior to publication in 1999 | Immunological test for HB  Colonoscopy | Referred to secondary care | - Median 59 (range 15-85) - 45.1% - NR - NR | 23/621 (3.70%) | 2 | 87.0 (84.4,89.6) | 88.1 (85.6,90.6) |
|  |  | Immunological test for Hb/Hp complex  Colonoscopy |  |  |  |  | 82.6 (79.6,85.6) | 80.8 (77.7%,83.9) |

HB, haemoglobin; Hp, haptoglobin; NR, not reported

1. D'Souza N, Hicks G, Benton SC, et al. The diagnostic accuracy of the faecal immunochemical test for colorectal cancer in risk-stratified symptomatic patients. *Annals of the Royal College of Surgeons of England* 2020a;102(3):174-79.

2. Gerrard AD, Maeda Y, Miller J, et al. Double faecal immunochemical testing in patients with symptoms suspicious of colorectal cancer. *British Journal of Surgery* 2023;110(4):471-80. doi: 10.1093/bjs/znad016

3. Johnstone MS, Burton P, Kourounis G, et al. Combining the quantitative faecal immunochemical test and full blood count reliably rules out colorectal cancer in a symptomatic patient referral pathway. *International Journal of Colorectal Disease* 2022a;37(2):457-66.

4. MacDonald S, MacDonald L, Godwin J, et al. The diagnostic accuracy of the faecal immunohistochemical test in identifying significant bowel disease in a symptomatic population. *Colorectal Disease* 2022;24(3):257-63.

5. Mowat C, Digby J, Strachan JA, et al. Faecal haemoglobin concentration thresholds for reassurance and urgent investigation for colorectal cancer based on a faecal immunochemical test in symptomatic patients in primary care. *Annals of Clinical Biochemistry* 2021;58(3):211-19.

6. Mowat C, Digby J, Strachan JA, et al. Impact of introducing a faecal immunochemical test (FIT) for haemoglobin into primary care on the outcome of patients with new bowel symptoms: a prospective cohort study. *BMJ Open Gastroenterology* 2019;6(1):e000293.

7. D’Souza N, Delisle TG, Chen M, et al. Faecal immunochemical testing in symptomatic patients to prioritize investigation: diagnostic accuracy from NICE FIT Study. *British Journal of Surgery* 2021a;108(7):804-10. doi: 10.1093/bjs/znaa132

8. D'Souza N, Delisle TG, Chen M, et al. Faecal immunochemical test is superior to symptoms in predicting pathology in patients with suspected colorectal cancer symptoms referred on a 2WW pathway: a diagnostic accuracy study. *Gut* 2021c;70(6):1130-38.

9. Farrugia A, Widlak M, Evans C, et al. Faecal immunochemical testing (FIT) in symptomatic patients: what are we missing? *Frontline Gastroenterol* 2020;11(1):28-33. doi: 10.1136/flgastro-2018-101174 [published Online First: 2019/12/31]

10. Turvill J, Mellen S, Jeffery L, et al. Diagnostic accuracy of one or two faecal haemoglobin and calprotectin measurements in patients with suspected colorectal cancer. *Scandinavian Journal of Gastroenterology* 2018;53(12):1526-34. doi: 10.1080/00365521.2018.1539761

11. Withrow DR, Shine B, Oke J, et al. Combining faecal immunochemical testing with blood test results for colorectal cancer risk stratification: a consecutive cohort of 16,604 patients presenting to primary care. *BMC Medicine* 2022;20(1):116.

12. Nicholson BD, James T, Paddon M, et al. Faecal immunochemical testing for adults with symptoms of colorectal cancer attending English primary care: a retrospective cohort study of 14 487 consecutive test requests. *Alimentary Pharmacology & Therapeutics* 2020;52(6):1031-41.

13. Chapman CJ, Banerjea A, Humes DJ, et al. Choice of faecal immunochemical test matters: comparison of OC-Sensor and HM-JACKarc, in the assessment of patients at high risk of colorectal cancer. *Clinical Chemistry & Laboratory Medicine* 2021;59(4):721-28.

14. Elbeltagi A, Salama M, Boxall P, et al. The Yield of Faecal Immunochemical Test in the Detection of Colorectal Cancer within a Fast-track Pathway at York, United Kingdom. *Turkish Journal of Colorectal Disease* 2022;32(3):178-85. doi: <https://dx.doi.org/10.4274/tjcd.galenos.2022.2021-11-5>

15. Faux JW, Cock K, Bromley R, et al. Colorectal two-week wait service and quantitative FIT: it's not just about colon cancer. *Annals of the Royal College of Surgeons of England* 2022;104(4):257-60.

16. Godber IM, Todd LM, Fraser CG, et al. Use of a faecal immunochemical test for haemoglobin can aid in the investigation of patients with lower abdominal symptoms. *Clinical Chemistry & Laboratory Medicine* 2016;54(4):595-602.

17. Nicholson BD, James T, East JE, et al. Experience of adopting faecal immunochemical testing to meet the NICE colorectal cancer referral criteria for low-risk symptomatic primary care patients in Oxfordshire, UK. *Frontline Gastroenterology* 2019;10(4):347-55.

18. Tang A, Chandler S, Torkington J, et al. Adapting the investigation of patients on urgent suspected cancer pathway with lower gastrointestinal symptoms across Wales during COVID-19. *Annals of the Royal College of Surgeons of England* 2022;26:26.

19. Turvill J, Turnock D, Cottingham D, et al. The Fast Track FIT study: diagnostic accuracy of faecal immunochemical test for haemoglobin in patients with suspected colorectal cancer. *Br J Gen Pract* 2021;71:E643–E51.

20. Crooks C, Banerjea A, Jones J, et al. Assessing empirical thresholds for investigation in people referred on a symptomatic colorectal cancer pathway: a cohort study utilising faecal immunochemical and blood tests in England. *medRxiv* 2023:2023.03.29.23287919. doi: 10.1101/2023.03.29.23287919

21. Bailey JA, Weller J, Chapman CJ, et al. Faecal immunochemical testing and blood tests for prioritization of urgent colorectal cancer referrals in symptomatic patients: A 2-year evaluation. *BJS Open* 2021a;5(2) (no pagination) doi: <https://dx.doi.org/10.1093/bjsopen/zraa056>

22. Bailey J, Morton A, Jones J, et al. “Low FIT” Colorectal cancer: A four-year comparison of the Nottingham “4F” protocol with FIT10 in symptomatic patients., 2023b.

23. Cama R, Kapoor N, Sawyer P, et al. Evaluation of 13,466 Fecal Immunochemical Tests in Patients Attending Primary Care for High- and Low-Risk Gastrointestinal Symptoms of Colorectal Cancer. *Digestive Diseases & Sciences* 2022;10:10.

24. Georgiou Delisle T, D'Souza N, Tan J, et al. Introduction of an integrated primary care faecal immunochemical test referral pathway for patients with suspected colorectal cancer symptoms. *Colorectal Disease* 2022a;08:08.

25. Benton SC, Piggott C, Zahoor Z, et al. A comparison of the faecal haemoglobin concentrations and diagnostic accuracy in patients suspected with colorectal cancer and serious bowel disease as reported on four different faecal immunochemical test systems. *Clinical Chemistry & Laboratory Medicine* 2022;60(8):1278-86.

26. Ball AJ, Aziz I, Parker S, et al. Fecal Immunochemical Testing in Patients With Low-Risk Symptoms of Colorectal Cancer: A Diagnostic Accuracy Study. *Journal of the National Comprehensive Cancer Network* 2022;20(9):989-96.e1.

27. Archer T, Aziz I, Kurien M, et al. Prioritisation of lower gastrointestinal endoscopy during the COVID-19 pandemic: outcomes of a novel triage pathway. *Frontline Gastroenterology* 2022;13(3):225-30.

28. Juul JS, Hornung N, Andersen B, et al. The value of using the faecal immunochemical test in general practice on patients presenting with non-alarm symptoms of colorectal cancer. *British Journal of Cancer* 2018;119(4):471-79. doi: <https://dx.doi.org/10.1038/s41416-018-0178-7>

29. Laszlo HE, Seward E, Ayling RM, et al. Faecal immunochemical test for patients with 'high-risk' bowel symptoms: a large prospective cohort study and updated literature review. *British Journal of Cancer* 2022;126(5):736-43.

30. Maclean W, Limb C, Mackenzie P, et al. Adoption of faecal immunochemical testing for 2-week-wait colorectal patients during the COVID-19 pandemic: an observational cohort study reporting a new service at a regional centre. *Colorectal Disease* 2021a;23(7):1622-29. doi: <https://dx.doi.org/10.1111/codi.15408>

31. Mowat C, Digby J, Strachan JA, et al. Faecal haemoglobin and faecal calprotectin as indicators of bowel disease in patients presenting to primary care with bowel symptoms. *Gut* 2016;65(9):1463-9.

32. Pin-Vieito N, Garcia Nimo L, Bujanda L, et al. Optimal diagnostic accuracy of quantitative faecal immunochemical test positivity thresholds for colorectal cancer detection in primary health care: A community-based cohort study. *United European Gastroenterology Journal* 2021;9(2):256-67.

33. MacLean W, Zahoor Z, O'Driscoll S, et al. Comparison of the QuikRead go<sup></sup>point-of-care faecal immunochemical test for haemoglobin with the FOB Gold Wide<sup></sup>laboratory analyser to diagnose colorectal cancer in symptomatic patients. *Clinical Chemistry and Laboratory Medicine* 2022a;60(1):101-08. doi: <https://dx.doi.org/10.1515/cclm-2021-0655>

34. Jordaan M, Welbourn H, Tyldsley K, et al. Development of a primary care pathway for using a faecal immunochemical test (FIT) to triage patients presenting with bowel symptoms. In: Medicine PL, ed., 2022.

35. Sieg A, Thoms C, Lüthgens K, et al. Detection of colorectal neoplasms by the highly sensitive hemoglobin-haptoglobin complex in feces. *International Journal of Colorectal Disease* 1999;14(6):267-71. doi: 10.1007/s003840050226
