# Supplementary material for: Faecal immunochemical tests for patients with symptoms suggestive of colorectal cancer: An updated systematic review and multiple‐threshold meta‐analysis of diagnostic test accuracy studies
Source: Colorectal Dis. 2024 Dec 17;27(1):e17255. doi: 10.1111/codi.17255 (PMC11683176; doi:10.1111/codi.17255)
Supplement: Supplementary file 8 — Data S8. [file CODI-27-0-s004.docx]

### 4.3.11 Comparative diagnostic test accuracy studies

Three studies conducted a comparison of two or more tests. Chapman *et al*. 2021^1^ reported on OC-Sensor DIANA and HM JACKarc, Benton *et al*. 2022^2^ compared HM-JACKarc, OC-Sensor PLEDIA, FOB Gold Wide/SENTiFIT 270, and NS-Prime and MacLean *et al*. 2022a compared FOB Gold Wide and QuikRead go. No one test appeared in all three comparisons.

Table 13 summarises the study characteristics and reports the sensitivity and specificity at a threshold of 10µg/g, and reports the conclusions drawn by the study authors. The remaining threshold data can be found in Supplementary File 2. The largest study included 38 CRC patients amongst a sample of 732.^1^ Both other studies^2 3^ had relatively small sample sizes and CRC events (see Table 13).

Different sensitivities and specificities were reported across the tests, and all three studies concluded that at least one test was different to another (see column 10 of Table 13). Due to the small number of CRC events in two of the trials, the small number of studies and the lack of a common comparator, it is difficult to draw any conclusions regarding the comparative performance of the tests, or what and whether different FIT cut-off values are required for each test based on these results. Benton *et al*, who performed an analysis of 4 tests notes that more work is required to understand the clinical impact of the use of different tests.

**Table 1: Sensitivity and specificity reported in studies comparing different tests within the same patients**

| **#** | **Author, year**  **Location**  **Recruitment dates**  **Study name (if available)** | **Analyser**  **Reference standard** | **Inclusion criteria** | **N with CRC/ N analysed (%)** | **Thresholds, µg/g** | **Threshold** | **Sensitivity (95% CI)** | **Specificity (95% CI)** | **Conclusion drawn by study authors** |
| --- | --- | --- | --- | --- | --- | --- | --- | --- | --- |
| 1 | Chapman 2021^1^  Nottingham University Hospitals Trust, UK  Sept 2016 to Sept 2017 | - HM JACKarc + HM JACKarc analyser   2WW investigations | 2WW patients who returned 2 types of FIT test | 38/732 (5.19%) | 4, 10, 22.6, 150 | 10 | 89.00 (75-97) | 74.00 (70-77) | Using OC-S results in higher referrals. Consequently, OC-S detected more cancers than HM-J for the same cut-offs.  Suggest that  analyser-specific f-Hb cut-offs are needed,  especially at lower f-Hb. |
|  |  | - OC-Sensor DIANA |  |  |  |  | 84.00 (69-94) | 78.00 (75-81) |  |
| 2 | Benton 2022^2^  50 NHS hospitals across England, UK  Oct 2017 to Dec 2019  NICE FIT | - HM-JACKarc   colonoscopy | NG12 high risk, who had colonoscopy. Randomised to cohort 1 who were given 4 tests | 7/233 (3.00%) | LoD, 10, 100 | 10 | 57.10 (25.1–84.2) | 84.50 (79.2–88.6) | At 10 μg/g, < half the  number of referrals would be made using SENTiFIT 270/FOB Gold Wide system compared to the other methods and dramatically fewer at the LoD. The calibration for the SENTiFIT 270/FOB Gold Wide gives lower f-Hb results than the other three systems. Supported by Bland Altman Difference plot.  Further work is required to understand  the clinical impact of these differences and to minimise them. |
|  |  | - OC-Sensor PLEDIA |  |  |  | 10 | 71.40 (35.9–91.8) | 85.80 (80.7–89.8) |  |
|  |  | - FOB Gold Wide - SENTiFIT 270 |  |  |  | 10 | 57.10 (25.1–84.2) | 93.40 (89.3–95.9) |  |
|  |  | - NS-Prime |  |  |  | 10 | 71.40 (35.9–91.8) | 83.60 (78.2–87.9) |  |
| 3 | MacLean 2022a^3^  Royal Surrey Foundation Trust, UK  July 2019 and March 2020 | - FOB Gold Wide SENTiFIT 270   Colonoscopy or CTC or flexisig | 2WW NG12 high/medium-risk | 14/553 (2.53%) | 10, 100, 150 | 10 | 100.00 (78.5 – 100) | 84.80 (81.5 - 87.6) | Good agreement around negative threshold, but more patients would be triaged to further colonic investigation if using the QuikRead go® |
|  |  | - QuikRead go |  |  |  |  | 92.90 (68.5 - 98.7) | 70.10 (66.1 - 73.8) |  |

2WW, two week wait; 95% CI, 95% confidence interval; CRC, colorectal cancer; LoD, limit of detection; N, number; NG12, national guideline 12; OC-S, OC-Sensor

1. Chapman CJ, Banerjea A, Humes DJ, et al. Choice of faecal immunochemical test matters: comparison of OC-Sensor and HM-JACKarc, in the assessment of patients at high risk of colorectal cancer. *Clinical Chemistry & Laboratory Medicine* 2021;59(4):721-28.

2. Benton SC, Piggott C, Zahoor Z, et al. A comparison of the faecal haemoglobin concentrations and diagnostic accuracy in patients suspected with colorectal cancer and serious bowel disease as reported on four different faecal immunochemical test systems. *Clinical Chemistry & Laboratory Medicine* 2022;60(8):1278-86.

3. MacLean W, Zahoor Z, O'Driscoll S, et al. Comparison of the QuikRead go<sup></sup>point-of-care faecal immunochemical test for haemoglobin with the FOB Gold Wide<sup></sup>laboratory analyser to diagnose colorectal cancer in symptomatic patients. *Clinical Chemistry and Laboratory Medicine* 2022a;60(1):101-08. doi: <https://dx.doi.org/10.1515/cclm-2021-0655>
