# Supplementary material for: Faecal immunochemical tests for patients with symptoms suggestive of colorectal cancer: An updated systematic review and multiple‐threshold meta‐analysis of diagnostic test accuracy studies
Source: Colorectal Dis. 2024 Dec 17;27(1):e17255. doi: 10.1111/codi.17255 (PMC11683176; doi:10.1111/codi.17255)
Supplement: Supplementary file 9 — Data S9. [file CODI-27-0-s002.docx]

**All test accuracy data**

# Introduction

This supplement contains all data used in the statistical syntheses of diagnostic test accuracy.

Please note that there may be some differences in the sensitivity and specificity reported here and in the original papers. This is because this data has been calculated from the TP, TN, FP, FN that was used in the synthesis. In order to include studies that did not report TP, TN etc in the synthesis, these values were sometimes back-calculated from other available data, e.g. sensitivity, specificity, total patients in the analysis, number of CRC events in the analysis and where numbers were large, there were multiple plausible values for TP, TN etc. Hence, recalculating TP, TN etc may result in different sensitivity and specificity.

Please also note that we carefully selected studies to enter each analysis and sensitivity analysis to avoid double counting of patients who may have been included in multiple analyses in the evidence base, but to include subgroup data (e.g. for population 1, 2 or 3) where possible. Therefore, not all lines of data relating to a specific test contributed to a given analysis.

# Data presented

Data relating to the diagnostic test accuracy for the detection of CRC by all tests are provided in Table 1. Data relating to the diagnostic test accuracy for the detection of advanced adenomas and inflammatory bowel disease by all tests are provided in Table 2.

**Table 1** Data entering the statistical syntheses of the diagnostic test accuracy of FITs for detection of CRC

| **Author, year** | **Test** | **Out-come** | **Pop. type** | **Sub-group** | **Ref Stand** | **No. Pts** | **CRC cases** | **Prev CRC** | **Thres-hold (µg/g)** | **TP** | **TN** | **FN** | **FP** | **Sensitivity** | **Specificity** |
| --- | --- | --- | --- | --- | --- | --- | --- | --- | --- | --- | --- | --- | --- | --- | --- |
| **FOB gold** | | | | | | | | | | | | | | | |
| Benton 2022^1^ | FOB Gold Wide - SENTiFIT 270 | CRC | 2 | 0 | 1 | 233 | 7 | 0.030043 | 2 | 4 | 186 | 3 | 40 | 57.1 (50.7,63.5) | 82.3 (77.4,87.2) |
| Benton 2022^1^ | FOB Gold Wide - SENTiFIT 270 | CRC | 2 | 0 | 1 | 233 | 7 | 0.030043 | 10 | 4 | 211 | 3 | 15 | 57.1 (50.7,63.5) | 93.4 (90.2,96.6) |
| Benton 2022^1^ | FOB Gold Wide - SENTiFIT 270 | CRC | 2 | 0 | 1 | 233 | 7 | 0.030043 | 100 | 4 | 219 | 3 | 7 | 57.1 (50.7,63.5) | 96.9 (94.7,99.1) |
| MacLean 2022a^2^ | FOB Gold Wide TÂ® with SENTiFITÂ® 270 analyser | CRC | 2 | 0 | 1 | 553 | 14 | 0.025316 | 3 | 14 | 415 | 0 | 124 | 100 (NE,NE) | 77 (73.5,80.5) |
| MacLean 2022a^2^ | FOB Gold Wide TÂ® with SENTiFITÂ® 270 analyser | CRC | 2 | 0 | 1 | 553 | 14 | 0.025316 | 10 | 14 | 457 | 0 | 82 | 100 (NE,NE) | 84.8 (81.8,87.8) |
| MacLean 2022a^2^ | FOB Gold Wide TÂ® with SENTiFITÂ® 270 analyser | CRC | 2 | 0 | 1 | 553 | 14 | 0.025316 | 100 | 13 | 506 | 1 | 33 | 92.9 (90.8,95) | 93.9 (91.9,95.9) |
| MacLean 2022a^2^ | FOB Gold Wide TÂ® with SENTiFITÂ® 270 analyser | CRC | 2 | 0 | 1 | 553 | 14 | 0.025316 | 150 | 11 | 511 | 3 | 28 | 78.6 (75.2,82) | 94.8 (92.9,96.7) |
| Schwettmann 2022^3^ | FOB Gold + Roche Cobas 8000 c702 analyser | CRC | 4 | 0 | 1 | 163 | 26 | 0.159509 | 10 | 25 | 71 | 1 | 66 | 96.2 (93.3,99.1) | 51.8 (44.1,59.5) |
| Schwettmann 2022^3^ | FOB Gold + Roche Cobas 8000 c702 analyser | CRC | 4 | 0 | 1 | 163 | 26 | 0.159509 | 15 | 25 | 79 | 1 | 58 | 96.2 (93.3,99.1) | 57.7 (50.1,65.3) |
| Schwettmann 2022^3^ | FOB Gold + Roche Cobas 8000 c702 analyser | CRC | 4 | 0 | 1 | 163 | 26 | 0.159509 | 20 | 25 | 83 | 1 | 54 | 96.2 (93.3,99.1) | 60.6 (53.1,68.1) |
| Schwettmann 2022^3^ | FOB Gold + Roche Cobas 8000 c702 analyser | CRC | 4 | 0 | 1 | 163 | 26 | 0.159509 | 30 | 24 | 92 | 2 | 45 | 92.3 (88.2,96.4) | 67.2 (60,74.4) |
| Schwettmann 2022^3^ | FOB Gold + Roche Cobas 8000 c702 analyser | CRC | 4 | 0 | 1 | 163 | 26 | 0.159509 | 40 | 23 | 95 | 3 | 42 | 88.5 (83.6,93.4) | 69.3 (62.2,76.4) |
| Schwettmann 2022^3^ | FOB Gold + Roche Cobas 8000 c702 analyser | CRC | 4 | 0 | 1 | 163 | 26 | 0.159509 | 50 | 23 | 99 | 3 | 38 | 88.5 (83.6,93.4) | 72.3 (65.4,79.2) |
| Schwettmann 2022^3^ | FOB Gold + Roche Cobas 8000 c702 analyser | CRC | 4 | 0 | 1 | 163 | 26 | 0.159509 | 100 | 21 | 104 | 5 | 33 | 80.8 (74.8,86.8) | 75.9 (69.3,82.5) |
| Schwettmann 2022^3^ | FOB Gold + Roche Cobas 8000 c702 analyser | CRC | 4 | 0 | 1 | 163 | 26 | 0.159509 | 150 | 15 | 114 | 11 | 23 | 57.7 (50.1,65.3) | 83.2 (77.5,88.9) |
| **HM-JACKarc** | | | | | | | | | | | | | | | |
| Benton 2022^1^ | HM-JACKarc | CRC | 2 | 0 | 1 | 233 | 7 | 0.030043 | 2 | 4 | 154 | 3 | 72 | 57.1 (50.7,63.5) | 68.1 (62.1,74.1) |
| Benton 2022^1^ | HM-JACKarc | CRC | 2 | 0 | 1 | 233 | 7 | 0.030043 | 10 | 4 | 191 | 3 | 35 | 57.1 (50.7,63.5) | 84.5 (79.9,89.1) |
| Benton 2022^1^ | HM-JACKarc | CRC | 2 | 0 | 1 | 233 | 7 | 0.030043 | 100 | 4 | 220 | 3 | 6 | 57.1 (50.7,63.5) | 97.3 (95.2,99.4) |
| Chapman 2021^4^ | HM JACKarc + HM JACKarc analyser | CRC | 4 | 0 | 1 | 732 | 38 | 0.051913 | 4 | 35 | 486 | 3 | 208 | 92 (90,94) | 70 (66.7,73.3) |
| Chapman 2021^4^ | HM JACKarc + HM JACKarc analyser | CRC | 4 | 0 | 1 | 732 | 38 | 0.051913 | 10 | 32 | 541 | 6 | 153 | 84 (81.3,86.7) | 78 (75,81) |
| Chapman 2021^4^ | HM JACKarc + HM JACKarc analyser | CRC | 4 | 0 | 1 | 732 | 38 | 0.051913 | 22.6 | 31 | 562 | 7 | 132 | 82 (79.2,84.8) | 81 (78.2,83.8) |
| Chapman 2021^4^ | HM JACKarc + HM JACKarc analyser | CRC | 4 | 0 | 1 | 732 | 38 | 0.051913 | 150 | 22 | 659 | 16 | 35 | 58 (54.4,61.6) | 95 (93.4,96.6) |
| Cunin 2020^5^ | HM JACKarc (analyser NR) | CRC | 4 | 1 - IDA | 2 | 189 | 20 | 0.10582 | 10 | 16 | 138 | 4 | 31 | 80 (74.3,85.7) | 81.6 (76.1,87.1) |
| Cunin 2020^5^ | HM JACKarc (analyser NR) | CRC | 4 | 1 - no IDA | 2 | 739 | 28 | 0.037889 | 10 | 25 | 597 | 3 | 114 | 89 (86.7,91.3) | 84 (81.4,86.6) |
| D'Souza 2020a^6^ | HM JACKarc analytical system | CRC | 1 | 0 | 1 | 298 | 12 | 0.040268 | 2 | 12 | 218 | 0 | 68 | 100 (NE,NE) | 76.2 (71.4,81) |
| D'Souza 2020a^6^ | HM JACKarc analytical system | CRC | 1 | 0 | 1 | 298 | 12 | 0.040268 | 10 | 11 | 253 | 1 | 33 | 92 (88.9,95.1) | 88.5 (84.9,92.1) |
| D'Souza 2020a^6^ | HM JACKarc analytical system | CRC | 2 | 0 | 1 | 160 | 8 | 0.05 | 2 | 8 | 108 | 0 | 44 | 100 (NE,NE) | 71 (64,78) |
| D'Souza 2020a^6^ | HM JACKarc analytical system | CRC | 2 | 0 | 1 | 160 | 8 | 0.05 | 10 | 7 | 128 | 1 | 24 | 87.5 (82.4,92.6) | 84.2 (78.5,89.9) |
| D'Souza 2020a^6^ | HM JACKarc analytical system | CRC | 3 | 0 | 1 | 138 | 4 | 0.028986 | 2 | 4 | 110 | 0 | 24 | 100 (NE,NE) | 82.1 (75.7,88.5) |
| D'Souza 2020a^6^ | HM JACKarc analytical system | CRC | 3 | 0 | 1 | 138 | 4 | 0.028986 | 10 | 4 | 125 | 0 | 9 | 100 (NE,NE) | 93.3 (89.1,97.5) |
| D'Souza 2021a^7^ | HM JACKarc analytical system | CRC | 2 | 0 | 1 | 7194 | 257 | 0.035724 | 2 | 251 | 4368 | 6 | 2569 | 97.7 (97.4,98) | 63 (61.9,64.1) |
| D'Souza 2021a^7^ | HM JACKarc analytical system | CRC | 4 | 0 | 1 | 1994 | 53 | 0.02658 | 2 | 50 | 1346 | 3 | 545 | 94.3 (93.3,95.3) | 71.8 (69.8,73.8) |
| D'Souza 2021a^7^ | HM JACKarc analytical system | CRC | 4 | 0 | 1 | 634 | 19 | 0.029968 | 2 | 18 | 393 | 1 | 222 | 94.7 (93,96.4) | 63.9 (60.2,67.6) |
| D'Souza 2021a^7^ | HM JACKarc analytical system | CRC | 2 | 0 | 1 | 7194 | 257 | 0.035724 | 10 | 237 | 5711 | 20 | 1226 | 92.2 (91.6,92.8) | 82.3 (81.4,83.2) |
| D'Souza 2021a^7^ | HM JACKarc analytical system | CRC | 4 | 0 | 1 | 1994 | 53 | 0.02658 | 10 | 46 | 1665 | 7 | 226 | 86.8 (85.3,88.3) | 88.4 (87,89.8) |
| D'Souza 2021a^7^ | HM JACKarc analytical system | CRC | 4 | 0 | 1 | 634 | 19 | 0.029968 | 10 | 16 | 508 | 3 | 107 | 84.2 (81.4,87) | 82.6 (79.6,85.6) |
| D'Souza 2021a^7^ | HM JACKarc analytical system | CRC | 4 | 1 | 1 | 479 | 15.807 | 0.033 | 10 | 16 | 378 | 0 | 85 | 100 (NE,NE) | 81.6 (78.1,85.1) |
| D'Souza 2021a^7^ | HM JACKarc analytical system | CRC | 2 | 0 | 1 | 7194 | 257 | 0.035724 | 150 | 185 | 6514 | 72 | 423 | 72 (71,73) | 93.9 (93.3,94.5) |
| D'Souza 2021a^7^ | HM JACKarc analytical system | CRC | 4 | 0 | 1 | 1994 | 53 | 0.02658 | 150 | 33 | 1881 | 20 | 60 | 62.3 (60.2,64.4) | 96.9 (96.1,97.7) |
| D'Souza 2021a^7^ | HM JACKarc analytical system | CRC | 4 | 0 | 1 | 634 | 19 | 0.029968 | 150 | 15 | 580 | 4 | 35 | 78.9 (75.7,82.1) | 94.3 (92.5,96.1) |
| D'Souza 2021c^8^ | HM JACKarc analytical system | CRC | 4 | 0 | 1 | 9822 | 329 | 0.033496 | 2 | 319 | 6157 | 10 | 3336 | 97 (96.7,97.3) | 64.9 (64,65.8) |
| D'Souza 2021c^8^ | HM JACKarc analytical system | CRC | 4 | 0 | 1 | 9822 | 329 | 0.033496 | 10 | 299 | 7930 | 30 | 1563 | 90.9 (90.3,91.5) | 83.5 (82.8,84.2) |
| D'Souza 2021c^8^ | HM JACKarc analytical system | CRC | 4 | 0 | 1 | 9822 | 329 | 0.033496 | 150 | 233 | 8977 | 96 | 516 | 70.8 (69.9,71.7) | 94.6 (94.2,95) |
| D’Souza 2021b^9^ | HM-JACKarc analyser | CRC | 4 | 7 age <50 | 1 | 1103 | 16 | 0.014506 | 2 | 14 | 765 | 2 | 322 | 87.5 (85.5,89.5) | 70.4 (67.7,73.1) |
| D’Souza 2021b^9^ | HM-JACKarc analyser | CRC | 4 | 7 age 50+ | 1 | 8719 | 313 | 0.035899 | 2 | 305 | 5388 | 8 | 3018 | 97.4 (97.1,97.7) | 64.1 (63.1,65.1) |
| D’Souza 2021b^9^ | HM-JACKarc analyser | CRC | 4 | 7 age <50 | 1 | 1103 | 16 | 0.014506 | 10 | 13 | 909 | 3 | 178 | 81.3 (79,83.6) | 83.6 (81.4,85.8) |
| D’Souza 2021b^9^ | HM-JACKarc analyser | CRC | 4 | 7 age 50+ | 1 | 8719 | 313 | 0.035899 | 10 | 286 | 7019 | 27 | 1387 | 91.4 (90.8,92) | 83.5 (82.7,84.3) |
| D’Souza 2021b^9^ | HM-JACKarc analyser | CRC | 4 | 7 age <50 | 1 | 1103 | 16 | 0.014506 | 50 | 11 | 1002 | 5 | 85 | 68.8 (66.1,71.5) | 92.2 (90.6,93.8) |
| D’Souza 2021b^9^ | HM-JACKarc analyser | CRC | 4 | 7 age 50+ | 1 | 8719 | 313 | 0.035899 | 50 | 222 | 7977 | 91 | 429 | 70.9 (69.9,71.9) | 94.9 (94.4,95.4) |
| Elbeltagi 2022^10^ | HM-JACKarc (personal communication) | CRC | 4 | 0 | 1 | 992 | 52 | 0.052419 | 6 | 52 | 0 | 0 | 940 | 100 (NE,NE) | 0 (NE,NE) |
| Elbeltagi 2022^10^ | HM-JACKarc (personal communication) | CRC | 4 | 0 | 1 | 992 | 52 | 0.052419 | 7.5 | 49 | 707 | 3 | 233 | 94.2 (92.7,95.7) | 75.2 (72.5,77.9) |
| Elbeltagi 2022^10^ | HM-JACKarc (personal communication) | CRC | 4 | 0 | 1 | 992 | 52 | 0.052419 | 8.5 | 48 | 713 | 4 | 227 | 92.3 (90.6,94) | 75.9 (73.2,78.6) |
| Elbeltagi 2022^10^ | HM-JACKarc (personal communication) | CRC | 4 | 0 | 1 | 992 | 52 | 0.052419 | 9.5 | 48 | 717 | 4 | 223 | 92.3 (90.6,94) | 76.3 (73.7,78.9) |
| Elbeltagi 2022^10^ | HM-JACKarc (personal communication) | CRC | 4 | 0 | 1 | 992 | 52 | 0.052419 | 10.5 | 48 | 725 | 4 | 215 | 92.3 (90.6,94) | 77.1 (74.5,79.7) |
| Elbeltagi 2022^10^ | HM-JACKarc (personal communication) | CRC | 4 | 0 | 1 | 992 | 52 | 0.052419 | 11.5 | 46 | 730 | 6 | 210 | 88.5 (86.5,90.5) | 77.7 (75.1,80.3) |
| Elbeltagi 2022^10^ | HM-JACKarc (personal communication) | CRC | 4 | 0 | 1 | 992 | 52 | 0.052419 | 12.5 | 46 | 735 | 6 | 205 | 88.5 (86.5,90.5) | 78.2 (75.6,80.8) |
| Elbeltagi 2022^10^ | HM-JACKarc (personal communication) | CRC | 4 | 0 | 1 | 992 | 52 | 0.052419 | 13.5 | 46 | 746 | 6 | 194 | 88.5 (86.5,90.5) | 79.4 (76.9,81.9) |
| Elbeltagi 2022^10^ | HM-JACKarc (personal communication) | CRC | 4 | 0 | 1 | 992 | 52 | 0.052419 | 14.5 | 46 | 747 | 6 | 193 | 88.5 (86.5,90.5) | 79.5 (77,82) |
| Elbeltagi 2022^10^ | HM-JACKarc (personal communication) | CRC | 4 | 0 | 1 | 992 | 52 | 0.052419 | 15.5 | 46 | 750 | 6 | 190 | 88.5 (86.5,90.5) | 79.8 (77.3,82.3) |
| Elbeltagi 2022^10^ | HM-JACKarc (personal communication) | CRC | 4 | 0 | 1 | 992 | 52 | 0.052419 | 16.5 | 46 | 757 | 6 | 183 | 88.5 (86.5,90.5) | 80.5 (78,83) |
| Elbeltagi 2022^10^ | HM-JACKarc (personal communication) | CRC | 4 | 0 | 1 | 992 | 52 | 0.052419 | 17.5 | 46 | 760 | 6 | 180 | 88.5 (86.5,90.5) | 80.9 (78.5,83.3) |
| Elbeltagi 2022^10^ | HM-JACKarc (personal communication) | CRC | 4 | 0 | 1 | 992 | 52 | 0.052419 | 18.5 | 46 | 762 | 6 | 178 | 88.5 (86.5,90.5) | 81.1 (78.7,83.5) |
| Elbeltagi 2022^10^ | HM-JACKarc (personal communication) | CRC | 4 | 0 | 1 | 992 | 52 | 0.052419 | 19.5 | 46 | 766 | 6 | 174 | 88.5 (86.5,90.5) | 81.5 (79.1,83.9) |
| Elbeltagi 2022^10^ | HM-JACKarc (personal communication) | CRC | 4 | 0 | 1 | 992 | 52 | 0.052419 | 20.5 | 45 | 770 | 7 | 170 | 86.5 (84.4,88.6) | 81.9 (79.5,84.3) |
| Elbeltagi 2022^10^ | HM-JACKarc (personal communication) | CRC | 4 | 0 | 1 | 992 | 52 | 0.052419 | 21.5 | 45 | 773 | 7 | 167 | 86.5 (84.4,88.6) | 82.2 (79.8,84.6) |
| Elbeltagi 2022^10^ | HM-JACKarc (personal communication) | CRC | 4 | 0 | 1 | 992 | 52 | 0.052419 | 22.5 | 45 | 776 | 7 | 164 | 86.5 (84.4,88.6) | 82.6 (80.2,85) |
| Elbeltagi 2022^10^ | HM-JACKarc (personal communication) | CRC | 4 | 0 | 1 | 992 | 52 | 0.052419 | 23.5 | 45 | 777 | 7 | 163 | 86.5 (84.4,88.6) | 82.7 (80.3,85.1) |
| Elbeltagi 2022^10^ | HM-JACKarc (personal communication) | CRC | 4 | 0 | 1 | 992 | 52 | 0.052419 | 24.5 | 45 | 778 | 7 | 162 | 86.5 (84.4,88.6) | 82.8 (80.5,85.1) |
| Elbeltagi 2022^10^ | HM-JACKarc (personal communication) | CRC | 4 | 0 | 1 | 992 | 52 | 0.052419 | 25.5 | 45 | 781 | 7 | 159 | 86.5 (84.4,88.6) | 83.1 (80.8,85.4) |
| Elbeltagi 2022^10^ | HM-JACKarc (personal communication) | CRC | 4 | 0 | 1 | 992 | 52 | 0.052419 | 26.5 | 45 | 783 | 7 | 157 | 86.5 (84.4,88.6) | 83.3 (81,85.6) |
| Elbeltagi 2022^10^ | HM-JACKarc (personal communication) | CRC | 4 | 0 | 1 | 992 | 52 | 0.052419 | 27.5 | 44 | 787 | 8 | 153 | 84.6 (82.4,86.8) | 83.7 (81.4,86) |
| Elbeltagi 2022^10^ | HM-JACKarc (personal communication) | CRC | 4 | 0 | 1 | 992 | 52 | 0.052419 | 28.5 | 44 | 788 | 8 | 152 | 84.6 (82.4,86.8) | 83.8 (81.5,86.1) |
| Elbeltagi 2022^10^ | HM-JACKarc (personal communication) | CRC | 4 | 0 | 1 | 992 | 52 | 0.052419 | 29.5 | 43 | 788 | 9 | 152 | 82.7 (80.3,85.1) | 83.8 (81.5,86.1) |
| Elbeltagi 2022^10^ | HM-JACKarc (personal communication) | CRC | 4 | 0 | 1 | 992 | 52 | 0.052419 | 30.5 | 43 | 790 | 9 | 150 | 82.7 (80.3,85.1) | 84 (81.7,86.3) |
| Elbeltagi 2022^10^ | HM-JACKarc (personal communication) | CRC | 4 | 0 | 1 | 992 | 52 | 0.052419 | 31.5 | 43 | 793 | 9 | 147 | 82.7 (80.3,85.1) | 84.4 (82.1,86.7) |
| Elbeltagi 2022^10^ | HM-JACKarc (personal communication) | CRC | 4 | 0 | 1 | 992 | 52 | 0.052419 | 32.5 | 43 | 795 | 9 | 145 | 82.7 (80.3,85.1) | 84.6 (82.4,86.8) |
| Elbeltagi 2022^10^ | HM-JACKarc (personal communication) | CRC | 4 | 0 | 1 | 992 | 52 | 0.052419 | 33.5 | 43 | 797 | 9 | 143 | 82.7 (80.3,85.1) | 84.8 (82.6,87) |
| Elbeltagi 2022^10^ | HM-JACKarc (personal communication) | CRC | 4 | 0 | 1 | 992 | 52 | 0.052419 | 34.5 | 42 | 798 | 10 | 142 | 80.8 (78.3,83.3) | 84.9 (82.7,87.1) |
| Elbeltagi 2022^10^ | HM-JACKarc (personal communication) | CRC | 4 | 0 | 1 | 992 | 52 | 0.052419 | 35.5 | 42 | 800 | 10 | 140 | 80.8 (78.3,83.3) | 85.1 (82.9,87.3) |
| Elbeltagi 2022^10^ | HM-JACKarc (personal communication) | CRC | 4 | 0 | 1 | 992 | 52 | 0.052419 | 36.5 | 42 | 804 | 10 | 136 | 80.8 (78.3,83.3) | 85.5 (83.3,87.7) |
| Elbeltagi 2022^10^ | HM-JACKarc (personal communication) | CRC | 4 | 0 | 1 | 992 | 52 | 0.052419 | 38.5 | 42 | 806 | 10 | 134 | 80.8 (78.3,83.3) | 85.7 (83.5,87.9) |
| Elbeltagi 2022^10^ | HM-JACKarc (personal communication) | CRC | 4 | 0 | 1 | 992 | 52 | 0.052419 | 40.5 | 41 | 809 | 11 | 131 | 78.8 (76.3,81.3) | 86.1 (83.9,88.3) |
| Elbeltagi 2022^10^ | HM-JACKarc (personal communication) | CRC | 4 | 0 | 1 | 992 | 52 | 0.052419 | 41.5 | 41 | 811 | 11 | 129 | 78.8 (76.3,81.3) | 86.3 (84.2,88.4) |
| Elbeltagi 2022^10^ | HM-JACKarc (personal communication) | CRC | 4 | 0 | 1 | 992 | 52 | 0.052419 | 42.5 | 40 | 814 | 12 | 126 | 76.9 (74.3,79.5) | 86.6 (84.5,88.7) |
| Elbeltagi 2022^10^ | HM-JACKarc (personal communication) | CRC | 4 | 0 | 1 | 992 | 52 | 0.052419 | 44 | 40 | 815 | 12 | 125 | 76.9 (74.3,79.5) | 86.7 (84.6,88.8) |
| Elbeltagi 2022^10^ | HM-JACKarc (personal communication) | CRC | 4 | 0 | 1 | 992 | 52 | 0.052419 | 46 | 40 | 816 | 12 | 124 | 76.9 (74.3,79.5) | 86.8 (84.7,88.9) |
| Elbeltagi 2022^10^ | HM-JACKarc (personal communication) | CRC | 4 | 0 | 1 | 992 | 52 | 0.052419 | 47.5 | 39 | 818 | 13 | 122 | 75 (72.3,77.7) | 87 (84.9,89.1) |
| Elbeltagi 2022^10^ | HM-JACKarc (personal communication) | CRC | 4 | 0 | 1 | 992 | 52 | 0.052419 | 48.5 | 39 | 819 | 13 | 121 | 75 (72.3,77.7) | 87.1 (85,89.2) |
| Elbeltagi 2022^10^ | HM-JACKarc (personal communication) | CRC | 4 | 0 | 1 | 992 | 52 | 0.052419 | 50 | 39 | 822 | 13 | 118 | 75 (72.3,77.7) | 87.4 (85.3,89.5) |
| Elbeltagi 2022^10^ | HM-JACKarc (personal communication) | CRC | 4 | 0 | 1 | 992 | 52 | 0.052419 | 51.5 | 39 | 824 | 13 | 116 | 75 (72.3,77.7) | 87.7 (85.7,89.7) |
| Elbeltagi 2022^10^ | HM-JACKarc (personal communication) | CRC | 4 | 0 | 1 | 992 | 52 | 0.052419 | 52.5 | 39 | 829 | 13 | 111 | 75 (72.3,77.7) | 88.2 (86.2,90.2) |
| Elbeltagi 2022^10^ | HM-JACKarc (personal communication) | CRC | 4 | 0 | 1 | 992 | 52 | 0.052419 | 53.5 | 39 | 830 | 13 | 110 | 75 (72.3,77.7) | 88.3 (86.3,90.3) |
| Elbeltagi 2022^10^ | HM-JACKarc (personal communication) | CRC | 4 | 0 | 1 | 992 | 52 | 0.052419 | 54.5 | 38 | 833 | 14 | 107 | 73.1 (70.3,75.9) | 88.6 (86.6,90.6) |
| Elbeltagi 2022^10^ | HM-JACKarc (personal communication) | CRC | 4 | 0 | 1 | 992 | 52 | 0.052419 | 55.5 | 38 | 834 | 14 | 106 | 73.1 (70.3,75.9) | 88.7 (86.7,90.7) |
| Elbeltagi 2022^10^ | HM-JACKarc (personal communication) | CRC | 4 | 0 | 1 | 992 | 52 | 0.052419 | 56.5 | 37 | 835 | 15 | 105 | 71.2 (68.4,74) | 88.8 (86.8,90.8) |
| Elbeltagi 2022^10^ | HM-JACKarc (personal communication) | CRC | 4 | 0 | 1 | 992 | 52 | 0.052419 | 57.5 | 36 | 837 | 16 | 103 | 69.2 (66.3,72.1) | 89 (87.1,90.9) |
| Elbeltagi 2022^10^ | HM-JACKarc (personal communication) | CRC | 4 | 0 | 1 | 992 | 52 | 0.052419 | 58.5 | 36 | 839 | 16 | 101 | 69.2 (66.3,72.1) | 89.3 (87.4,91.2) |
| Elbeltagi 2022^10^ | HM-JACKarc (personal communication) | CRC | 4 | 0 | 1 | 992 | 52 | 0.052419 | 59.5 | 35 | 840 | 17 | 100 | 67.3 (64.4,70.2) | 89.4 (87.5,91.3) |
| Elbeltagi 2022^10^ | HM-JACKarc (personal communication) | CRC | 4 | 0 | 1 | 992 | 52 | 0.052419 | 60.5 | 34 | 841 | 18 | 99 | 65.4 (62.4,68.4) | 89.5 (87.6,91.4) |
| Elbeltagi 2022^10^ | HM-JACKarc (personal communication) | CRC | 4 | 0 | 1 | 992 | 52 | 0.052419 | 61.5 | 33 | 841 | 19 | 99 | 63.5 (60.5,66.5) | 89.5 (87.6,91.4) |
| Elbeltagi 2022^10^ | HM-JACKarc (personal communication) | CRC | 4 | 0 | 1 | 992 | 52 | 0.052419 | 62.5 | 33 | 842 | 19 | 98 | 63.5 (60.5,66.5) | 89.6 (87.7,91.5) |
| Elbeltagi 2022^10^ | HM-JACKarc (personal communication) | CRC | 4 | 0 | 1 | 992 | 52 | 0.052419 | 63.5 | 33 | 843 | 19 | 97 | 63.5 (60.5,66.5) | 89.7 (87.8,91.6) |
| Elbeltagi 2022^10^ | HM-JACKarc (personal communication) | CRC | 4 | 0 | 1 | 992 | 52 | 0.052419 | 65 | 33 | 844 | 19 | 96 | 63.5 (60.5,66.5) | 89.8 (87.9,91.7) |
| Elbeltagi 2022^10^ | HM-JACKarc (personal communication) | CRC | 4 | 0 | 1 | 992 | 52 | 0.052419 | 66.5 | 32 | 844 | 20 | 96 | 61.5 (58.5,64.5) | 89.8 (87.9,91.7) |
| Elbeltagi 2022^10^ | HM-JACKarc (personal communication) | CRC | 4 | 0 | 1 | 992 | 52 | 0.052419 | 67.5 | 32 | 845 | 20 | 95 | 61.5 (58.5,64.5) | 89.9 (88,91.8) |
| Elbeltagi 2022^10^ | HM-JACKarc (personal communication) | CRC | 4 | 0 | 1 | 992 | 52 | 0.052419 | 68.5 | 32 | 846 | 20 | 94 | 61.5 (58.5,64.5) | 90 (88.1,91.9) |
| Elbeltagi 2022^10^ | HM-JACKarc (personal communication) | CRC | 4 | 0 | 1 | 992 | 52 | 0.052419 | 69.5 | 32 | 847 | 20 | 93 | 61.5 (58.5,64.5) | 90.1 (88.2,92) |
| Elbeltagi 2022^10^ | HM-JACKarc (personal communication) | CRC | 4 | 0 | 1 | 992 | 52 | 0.052419 | 71 | 31 | 848 | 21 | 92 | 59.6 (56.5,62.7) | 90.2 (88.3,92.1) |
| Elbeltagi 2022^10^ | HM-JACKarc (personal communication) | CRC | 4 | 0 | 1 | 992 | 52 | 0.052419 | 74 | 31 | 849 | 21 | 91 | 59.6 (56.5,62.7) | 90.3 (88.5,92.1) |
| Elbeltagi 2022^10^ | HM-JACKarc (personal communication) | CRC | 4 | 0 | 1 | 992 | 52 | 0.052419 | 76.5 | 31 | 850 | 21 | 90 | 59.6 (56.5,62.7) | 90.4 (88.6,92.2) |
| Elbeltagi 2022^10^ | HM-JACKarc (personal communication) | CRC | 4 | 0 | 1 | 992 | 52 | 0.052419 | 78.5 | 31 | 851 | 21 | 89 | 59.6 (56.5,62.7) | 90.5 (88.7,92.3) |
| Elbeltagi 2022^10^ | HM-JACKarc (personal communication) | CRC | 4 | 0 | 1 | 992 | 52 | 0.052419 | 81 | 31 | 853 | 21 | 87 | 59.6 (56.5,62.7) | 90.7 (88.9,92.5) |
| Elbeltagi 2022^10^ | HM-JACKarc (personal communication) | CRC | 4 | 0 | 1 | 992 | 52 | 0.052419 | 83 | 30 | 854 | 22 | 86 | 57.7 (54.6,60.8) | 90.9 (89.1,92.7) |
| Elbeltagi 2022^10^ | HM-JACKarc (personal communication) | CRC | 4 | 0 | 1 | 992 | 52 | 0.052419 | 85 | 30 | 855 | 22 | 85 | 57.7 (54.6,60.8) | 91 (89.2,92.8) |
| Elbeltagi 2022^10^ | HM-JACKarc (personal communication) | CRC | 4 | 0 | 1 | 992 | 52 | 0.052419 | 87 | 30 | 857 | 22 | 83 | 57.7 (54.6,60.8) | 91.2 (89.4,93) |
| Elbeltagi 2022^10^ | HM-JACKarc (personal communication) | CRC | 4 | 0 | 1 | 992 | 52 | 0.052419 | 89 | 30 | 858 | 22 | 82 | 57.7 (54.6,60.8) | 91.3 (89.5,93.1) |
| Elbeltagi 2022^10^ | HM-JACKarc (personal communication) | CRC | 4 | 0 | 1 | 992 | 52 | 0.052419 | 92.5 | 29 | 858 | 23 | 82 | 55.8 (52.7,58.9) | 91.3 (89.5,93.1) |
| Elbeltagi 2022^10^ | HM-JACKarc (personal communication) | CRC | 4 | 0 | 1 | 992 | 52 | 0.052419 | 96.5 | 29 | 859 | 23 | 81 | 55.8 (52.7,58.9) | 91.4 (89.7,93.1) |
| Elbeltagi 2022^10^ | HM-JACKarc (personal communication) | CRC | 4 | 0 | 1 | 992 | 52 | 0.052419 | 100 | 29 | 860 | 23 | 80 | 55.8 (52.7,58.9) | 91.5 (89.8,93.2) |
| Elbeltagi 2022^10^ | HM-JACKarc (personal communication) | CRC | 4 | 0 | 1 | 992 | 52 | 0.052419 | 106 | 29 | 861 | 23 | 79 | 55.8 (52.7,58.9) | 91.6 (89.9,93.3) |
| Elbeltagi 2022^10^ | HM-JACKarc (personal communication) | CRC | 4 | 0 | 1 | 992 | 52 | 0.052419 | 110.5 | 28 | 862 | 24 | 78 | 53.8 (50.7,56.9) | 91.7 (90,93.4) |
| Elbeltagi 2022^10^ | HM-JACKarc (personal communication) | CRC | 4 | 0 | 1 | 992 | 52 | 0.052419 | 111.5 | 28 | 864 | 24 | 76 | 53.8 (50.7,56.9) | 91.9 (90.2,93.6) |
| Elbeltagi 2022^10^ | HM-JACKarc (personal communication) | CRC | 4 | 0 | 1 | 992 | 52 | 0.052419 | 114 | 28 | 865 | 24 | 75 | 53.8 (50.7,56.9) | 92 (90.3,93.7) |
| Elbeltagi 2022^10^ | HM-JACKarc (personal communication) | CRC | 4 | 0 | 1 | 992 | 52 | 0.052419 | 116.5 | 28 | 867 | 24 | 73 | 53.8 (50.7,56.9) | 92.2 (90.5,93.9) |
| Elbeltagi 2022^10^ | HM-JACKarc (personal communication) | CRC | 4 | 0 | 1 | 992 | 52 | 0.052419 | 118 | 28 | 868 | 24 | 72 | 53.8 (50.7,56.9) | 92.3 (90.6,94) |
| Elbeltagi 2022^10^ | HM-JACKarc (personal communication) | CRC | 4 | 0 | 1 | 992 | 52 | 0.052419 | 120.5 | 28 | 869 | 24 | 71 | 53.8 (50.7,56.9) | 92.4 (90.8,94) |
| Elbeltagi 2022^10^ | HM-JACKarc (personal communication) | CRC | 4 | 0 | 1 | 992 | 52 | 0.052419 | 123.5 | 28 | 870 | 24 | 70 | 53.8 (50.7,56.9) | 92.6 (91,94.2) |
| Elbeltagi 2022^10^ | HM-JACKarc (personal communication) | CRC | 4 | 0 | 1 | 992 | 52 | 0.052419 | 126.5 | 27 | 870 | 25 | 70 | 51.9 (48.8,55) | 92.6 (91,94.2) |
| Elbeltagi 2022^10^ | HM-JACKarc (personal communication) | CRC | 4 | 0 | 1 | 992 | 52 | 0.052419 | 132.5 | 26 | 870 | 26 | 70 | 50 (46.9,53.1) | 92.6 (91,94.2) |
| Elbeltagi 2022^10^ | HM-JACKarc (personal communication) | CRC | 4 | 0 | 1 | 992 | 52 | 0.052419 | 139 | 25 | 870 | 27 | 70 | 48.1 (45,51.2) | 92.6 (91,94.2) |
| Elbeltagi 2022^10^ | HM-JACKarc (personal communication) | CRC | 4 | 0 | 1 | 992 | 52 | 0.052419 | 150.5 | 25 | 871 | 27 | 69 | 48.1 (45,51.2) | 92.7 (91.1,94.3) |
| Elbeltagi 2022^10^ | HM-JACKarc (personal communication) | CRC | 4 | 0 | 1 | 992 | 52 | 0.052419 | 162 | 25 | 872 | 27 | 68 | 48.1 (45,51.2) | 92.8 (91.2,94.4) |
| Elbeltagi 2022^10^ | HM-JACKarc (personal communication) | CRC | 4 | 0 | 1 | 992 | 52 | 0.052419 | 165 | 24 | 872 | 28 | 68 | 46.2 (43.1,49.3) | 92.8 (91.2,94.4) |
| Elbeltagi 2022^10^ | HM-JACKarc (personal communication) | CRC | 4 | 0 | 1 | 992 | 52 | 0.052419 | 167.5 | 24 | 873 | 28 | 67 | 46.2 (43.1,49.3) | 92.9 (91.3,94.5) |
| Elbeltagi 2022^10^ | HM-JACKarc (personal communication) | CRC | 4 | 0 | 1 | 992 | 52 | 0.052419 | 195.5 | 24 | 875 | 28 | 65 | 46.2 (43.1,49.3) | 93.1 (91.5,94.7) |
| Elbeltagi 2022^10^ | HM-JACKarc (personal communication) | CRC | 4 | 0 | 1 | 992 | 52 | 0.052419 | 222.5 | 24 | 876 | 28 | 64 | 46.2 (43.1,49.3) | 93.2 (91.6,94.8) |
| Elbeltagi 2022^10^ | HM-JACKarc (personal communication) | CRC | 4 | 0 | 1 | 992 | 52 | 0.052419 | 227 | 24 | 877 | 28 | 63 | 46.2 (43.1,49.3) | 93.3 (91.7,94.9) |
| Elbeltagi 2022^10^ | HM-JACKarc (personal communication) | CRC | 4 | 0 | 1 | 992 | 52 | 0.052419 | 241.5 | 24 | 878 | 28 | 62 | 46.2 (43.1,49.3) | 93.4 (91.9,94.9) |
| Elbeltagi 2022^10^ | HM-JACKarc (personal communication) | CRC | 4 | 0 | 1 | 992 | 52 | 0.052419 | 260.5 | 23 | 878 | 29 | 62 | 44.2 (41.1,47.3) | 93.4 (91.9,94.9) |
| Elbeltagi 2022^10^ | HM-JACKarc (personal communication) | CRC | 4 | 0 | 1 | 992 | 52 | 0.052419 | 276 | 23 | 879 | 29 | 61 | 44.2 (41.1,47.3) | 93.5 (92,95) |
| Elbeltagi 2022^10^ | HM-JACKarc (personal communication) | CRC | 4 | 0 | 1 | 992 | 52 | 0.052419 | 285 | 23 | 880 | 29 | 60 | 44.2 (41.1,47.3) | 93.6 (92.1,95.1) |
| Elbeltagi 2022^10^ | HM-JACKarc (personal communication) | CRC | 4 | 0 | 1 | 992 | 52 | 0.052419 | 292 | 23 | 881 | 29 | 59 | 44.2 (41.1,47.3) | 93.7 (92.2,95.2) |
| Elbeltagi 2022^10^ | HM-JACKarc (personal communication) | CRC | 4 | 0 | 1 | 992 | 52 | 0.052419 | 297.5 | 23 | 882 | 29 | 58 | 44.2 (41.1,47.3) | 93.8 (92.3,95.3) |
| Elbeltagi 2022^10^ | HM-JACKarc (personal communication) | CRC | 4 | 0 | 1 | 992 | 52 | 0.052419 | 303 | 23 | 883 | 29 | 57 | 44.2 (41.1,47.3) | 93.9 (92.4,95.4) |
| Elbeltagi 2022^10^ | HM-JACKarc (personal communication) | CRC | 4 | 0 | 1 | 992 | 52 | 0.052419 | 308.5 | 23 | 885 | 29 | 55 | 44.2 (41.1,47.3) | 94.1 (92.6,95.6) |
| Elbeltagi 2022^10^ | HM-JACKarc (personal communication) | CRC | 4 | 0 | 1 | 992 | 52 | 0.052419 | 322 | 23 | 886 | 29 | 54 | 44.2 (41.1,47.3) | 94.3 (92.9,95.7) |
| Elbeltagi 2022^10^ | HM-JACKarc (personal communication) | CRC | 4 | 0 | 1 | 992 | 52 | 0.052419 | 348.5 | 22 | 886 | 30 | 54 | 42.3 (39.2,45.4) | 94.3 (92.9,95.7) |
| Elbeltagi 2022^10^ | HM-JACKarc (personal communication) | CRC | 4 | 0 | 1 | 992 | 52 | 0.052419 | 373 | 22 | 887 | 30 | 53 | 42.3 (39.2,45.4) | 94.4 (93,95.8) |
| Elbeltagi 2022^10^ | HM-JACKarc (personal communication) | CRC | 4 | 0 | 1 | 992 | 52 | 0.052419 | 387.5 | 22 | 888 | 30 | 52 | 42.3 (39.2,45.4) | 94.5 (93.1,95.9) |
| Elbeltagi 2022^10^ | HM-JACKarc (personal communication) | CRC | 4 | 0 | 1 | 992 | 52 | 0.052419 | 395 | 22 | 889 | 30 | 51 | 42.3 (39.2,45.4) | 94.6 (93.2,96) |
| Elbeltagi 2022^10^ | HM-JACKarc (personal communication) | CRC | 4 | 0 | 1 | 992 | 52 | 0.052419 | 399.5 | 22 | 890 | 30 | 50 | 42.3 (39.2,45.4) | 94.7 (93.3,96.1) |
| Elbeltagi 2022^10^ | HM-JACKarc (personal communication) | CRC | 4 | 0 | 1 | 992 | 52 | 0.052419 | 401 | 0 | 940 | 52 | 0 | 0 (NE,NE) | 100 (NE,NE) |
| Farrugia 2020^11^ | HM JACKarc automated system | CRC | 2 | 0 | 1 | 519 | 33 | 0.063584 | 10 | 28 | 395 | 5 | 91 | 84.8 (81.7,87.9) | 81.3 (77.9,84.7) |
| Farrugia 2020^11^ | HM JACKarc automated system | CRC | 4 | 0 | 1 | 612 | 38 | 0.062092 | 10 | 33 | 472 | 5 | 102 | 86.8 (84.1,89.5) | 82.2 (79.2,85.2) |
| Farrugia 2020^11^ | HM JACKarc automated system | CRC | 4 | 0 | 1 | 79 | 5 | 0.063291 | 10 | 5 | 68 | 0 | 6 | 100 (NE,NE) | 91.9 (85.9,97.9) |
| Faux 2022^12^ | HM-JACKarc analyser | CRC | 4 | 0 | 2 | 175 | 6 | 0.034286 | 10 | 4 | 141 | 2 | 28 | 66.7 (59.7,73.7) | 83.4 (77.9,88.9) |
| Gerrard 2023^13^ | HM-JACKarc analyser | CRC | 1 | 0 | 1 | 2260 | 69 | 0.030531 | 10 | 58 | 1696 | 11 | 495 | 84.1 (82.6,85.6) | 77.4 (75.7,79.1) |
| Gerrard 2023^13^ | HM-JACKarc analyser | CRC | 1 | 0 | 1 | 3426 | 135 | 0.039405 | 10 | 126 | 2567 | 9 | 724 | 93.3 (92.5,94.1) | 78 (76.6,79.4) |
| Gerrard 2023^13^ | HM-JACKarc analyser | CRC | 1 | 1 (unclear if IDA or anaemia) | 1 | 567 | 38 | 0.067019 | 10 | 31 | 363 | 7 | 166 | 81.6 (78.4,84.8) | 68.6 (64.8,72.4) |
| Gerrard 2023^13^ | HM-JACKarc analyser | CRC | 1 | 1 (unclear if IDA or anaemia) | 1 | 480 | 29 | 0.060417 | 10 | 27 | 271 | 2 | 180 | 93.1 (90.8,95.4) | 60.1 (55.7,64.5) |
| Godber 2016^14^ | HM JACKarc analyser | CRC | 4 | 0 | 1 | 484 | 11 | 0.022727 | 10 | 11 | 362 | 0 | 111 | 100 (NE,NE) | 76.5 (72.7,80.3) |
| Johnstone 2022a^15^ | HM-JACKarc confirmed by author | CRC | 1 | 0 | 2 | 4737 | 61 | 0.012877 | 10 | 53 | 3763 | 5 | 916 | 91.8 (91,92.6) | 80.4 (79.3,81.5) |
| Johnstone 2022a^15^ | HM-JACKarc confirmed by author | CRC | 1 | 1 - not anaemic | 2 | 3238 | 32 | 0.009883 | 10 | 31 | 2631 | 1 | 606 | 96.9 (96.3,97.5) | 81.3 (80,82.6) |
| Johnstone 2022a^15^ | HM-JACKarc confirmed by author | CRC | 1 | 1 - anaemia. anaemic (male < 130 mg/L, female < 120 mg/L) based on WHO guidelines | 2 | 793 | 26 | 0.032787 | 10 | 22 | 559 | 4 | 208 | 84.6 (82.1,87.1) | 72.9 (69.8,76) |
| Johnstone 2022a^15^ | HM-JACKarc confirmed by author | CRC | 1 | 0 | 2 | 4737 | 61 | 0.012877 | 150 | 42 | 4387 | 16 | 292 | 72.4 (71.1,73.7) | 93.8 (93.1,94.5) |
| Johnstone 2022a^15^ | HM-JACKarc confirmed by author | CRC | 1 | 0 | 2 | 4737 | 61 | 0.012877 | 400 | 34 | 4492 | 24 | 187 | 58.6 (57.2,60) | 96 (95.4,96.6) |
| MacDonald 2022^16^ | HM-JACKarc | CRC | 1 | 0 | 2 | 5250 | 151 | 0.028762 | 10 | 132 | 3399 | 19 | 1700 | 87.4 (86.5,88.3) | 66.6 (65.3,67.9) |
| Mowat 2021^17^ & 2019^18^ | HM JACKarc | CRC | 1 | 0 | 2 | 5381 | 105 | 0.019513 | 2 | 102 | 2611 | 3 | 2665 | 97.1 (96.7,97.5) | 49.5 (48.2,50.8) |
| Mowat 2021^17^ & 2019^18^ | HM JACKarc | CRC | 1 | 0 | 2 | 5381 | 105 | 0.019513 | 7 | 93 | 4004 | 12 | 1272 | 88.6 (87.8,89.4) | 75.9 (74.8,77) |
| Mowat 2021^17^ & 2019^18^ | HM JACKarc | CRC | 1 | 0 | 2 | 5381 | 105 | 0.019513 | 10 | 91 | 4190 | 14 | 1086 | 86.7 (85.8,87.6) | 79.4 (78.3,80.5) |
| Mowat 2021^17^ & 2019^18^ | HM JACKarc | CRC | 1 | 0 | 2 | 5381 | 105 | 0.019513 | 20 | 87 | 4459 | 18 | 817 | 82.9 (81.9,83.9) | 84.5 (83.5,85.5) |
| Mowat 2021^17^ & 2019^18^ | HM JACKarc | CRC | 1 | 0 | 2 | 5381 | 105 | 0.019513 | 50 | 78 | 4706 | 27 | 570 | 74.3 (73.1,75.5) | 89.2 (88.4,90) |
| Mowat 2021^17^ & 2019^18^ | HM JACKarc | CRC | 1 | 0 | 2 | 5381 | 105 | 0.019513 | 100 | 73 | 4846 | 32 | 430 | 69.5 (68.3,70.7) | 91.8 (91.1,92.5) |
| Mowat 2021^17^ & 2019^18^ | HM JACKarc | CRC | 1 | 0 | 2 | 5381 | 105 | 0.019513 | 150 | 67 | 4924 | 38 | 352 | 63.8 (62.5,65.1) | 93.3 (92.6,94) |
| Mowat 2021^17^ & 2019^18^ | HM JACKarc | CRC | 1 | 0 | 2 | 5381 | 105 | 0.019513 | 200 | 65 | 4963 | 40 | 313 | 61.9 (60.6,63.2) | 94.1 (93.5,94.7) |
| Mowat 2021^17^ & 2019^18^ | HM JACKarc | CRC | 1 | 0 | 2 | 5381 | 105 | 0.019513 | 250 | 62 | 4990 | 43 | 286 | 59 (57.7,60.3) | 94.6 (94,95.2) |
| Mowat 2021^17^ & 2019^18^ | HM JACKarc | CRC | 1 | 0 | 2 | 5381 | 105 | 0.019513 | 300 | 59 | 5015 | 46 | 261 | 56.2 (54.9,57.5) | 95.1 (94.5,95.7) |
| Mowat 2021^17^ & 2019^18^ | HM JACKarc | CRC | 1 | 0 | 2 | 5381 | 105 | 0.019513 | 350 | 57 | 5029 | 48 | 247 | 54.3 (53,55.6) | 95.3 (94.7,95.9) |
| Mowat 2021^17^ & 2019^18^ | HM JACKarc | CRC | 1 | 0 | 2 | 5381 | 105 | 0.019513 | 400 | 56 | 5040 | 49 | 236 | 53.3 (52,54.6) | 95.5 (94.9,96.1) |
| Nicholson 2019 ^19^ | HM JACKarc | CRC | 4 | 0 | 2 | 238 | 7 | 0.029412 | 7 | 6 | 206 | 1 | 25 | 85.7 (81.3,90.1) | 89.2 (85.3,93.1) |
| Nicholson 2019 ^19^ | HM JACKarc | CRC | 4 | 0 | 2 | 238 | 7 | 0.029412 | 10 | 6 | 209 | 1 | 22 | 85.7 (81.3,90.1) | 90.5 (86.8,94.2) |
| Nicholson 2019 ^19^ | HM JACKarc | CRC | 4 | 0 | 2 | 238 | 7 | 0.029412 | 20 | 5 | 214 | 2 | 17 | 71.4 (65.7,77.1) | 92.6 (89.3,95.9) |
| Nicholson 2019 ^19^ | HM JACKarc | CRC | 4 | 0 | 2 | 238 | 7 | 0.029412 | 50 | 4 | 221 | 3 | 10 | 57.1 (50.8,63.4) | 95.7 (93.1,98.3) |
| Nicholson (2020) | HM JACKarc | CRC | 4 | 0 | 2 | 9896 | 105 | 0.01061 | 7 | 96 | 8792 | 9 | 999 | 91.4 (90.8,92) | 89.8 (89.2,90.4) |
| Nicholson 2020^20^ | HM JACKarc | CRC | 4 | 2 | 2 | 4101 | 65 | 0.01585 | 7 | 60 | 3548 | 5 | 488 | 92.3 (91.5,93.1) | 87.9 (86.9,88.9) |
| Nicholson 2020^20^ | HM JACKarc | CRC | 4 | 3 | 2 | 5795 | 40 | 0.006903 | 7 | 36 | 5243 | 4 | 512 | 90 (89.2,90.8) | 91.1 (90.4,91.8) |
| Nicholson 2020^20^ | HM JACKarc | CRC | 4 | 0 | 2 | 9896 | 105 | 0.01061 | 10 | 95 | 8939 | 10 | 852 | 90.5 (89.9,91.1) | 91.3 (90.7,91.9) |
| Nicholson 2020^20^ | HM JACKarc | CRC | 4 | 2 | 2 | 4101 | 65 | 0.01585 | 10 | 59 | 3624 | 6 | 412 | 90.8 (89.9,91.7) | 89.8 (88.9,90.7) |
| Nicholson 2020^20^ | HM JACKarc | CRC | 4 | 3 | 2 | 5795 | 40 | 0.006903 | 10 | 36 | 5318 | 4 | 437 | 90 (89.2,90.8) | 92.4 (91.7,93.1) |
| Nicholson 2020^20^ | HM JACKarc | CRC | 4 | 0 | 2 | 9896 | 105 | 0.01061 | 20 | 89 | 9174 | 16 | 617 | 84.8 (84.1,85.5) | 93.7 (93.2,94.2) |
| Nicholson 2020^20^ | HM JACKarc | CRC | 4 | 2 | 2 | 4101 | 65 | 0.01585 | 20 | 54 | 3725 | 11 | 311 | 83.1 (82,84.2) | 92.3 (91.5,93.1) |
| Nicholson 2020^20^ | HM JACKarc | CRC | 4 | 3 | 2 | 5795 | 40 | 0.006903 | 20 | 35 | 5444 | 5 | 311 | 87.5 (86.6,88.4) | 94.6 (94,95.2) |
| Nicholson 2020^20^ | HM JACKarc | CRC | 4 | 0 | 2 | 9896 | 105 | 0.01061 | 50 | 78 | 9439 | 27 | 352 | 74.3 (73.4,75.2) | 96.4 (96,96.8) |
| Nicholson 2020^20^ | HM JACKarc | CRC | 4 | 2 | 2 | 4101 | 65 | 0.01585 | 50 | 48 | 3854 | 17 | 182 | 73.8 (72.5,75.1) | 95.5 (94.9,96.1) |
| Nicholson 2020^20^ | HM JACKarc | CRC | 4 | 3 | 2 | 5795 | 40 | 0.006903 | 50 | 30 | 5577 | 10 | 178 | 75 (73.9,76.1) | 96.9 (96.5,97.3) |
| Nicholson 2020^20^ | HM JACKarc | CRC | 4 | 0 | 2 | 9896 | 105 | 0.01061 | 100 | 64 | 9556 | 41 | 235 | 61 (60,62) | 97.6 (97.3,97.9) |
| Nicholson 2020^20^ | HM JACKarc | CRC | 4 | 2 | 2 | 4101 | 65 | 0.01585 | 100 | 39 | 3907 | 26 | 129 | 60 (58.5,61.5) | 96.8 (96.3,97.3) |
| Nicholson 2020^20^ | HM JACKarc | CRC | 4 | 3 | 2 | 5795 | 40 | 0.006903 | 100 | 25 | 5646 | 15 | 109 | 62.5 (61.3,63.7) | 98.1 (97.7,98.5) |
| Nicholson 2020^20^ | HM JACKarc | CRC | 4 | 0 | 2 | 9896 | 105 | 0.01061 | 120 | 60 | 9576 | 45 | 215 | 57.1 (56.1,58.1) | 97.8 (97.5,98.1) |
| Nicholson 2020^20^ | HM JACKarc | CRC | 4 | 2 | 2 | 4101 | 65 | 0.01585 | 120 | 36 | 3923 | 29 | 113 | 55.4 (53.9,56.9) | 97.2 (96.7,97.7) |
| Nicholson 2020^20^ | HM JACKarc | CRC | 4 | 3 | 2 | 5795 | 40 | 0.006903 | 120 | 24 | 5657 | 16 | 98 | 60 (58.7,61.3) | 98.3 (98,98.6) |
| Nicholson 2020^20^ | HM JACKarc | CRC | 4 | 0 | 2 | 9896 | 105 | 0.01061 | 150 | 57 | 9605 | 48 | 186 | 54.3 (53.3,55.3) | 98.1 (97.8,98.4) |
| Nicholson 2020^20^ | HM JACKarc | CRC | 4 | 2 | 2 | 4101 | 65 | 0.01585 | 150 | 33 | 3935 | 32 | 101 | 50.8 (49.3,52.3) | 97.5 (97,98) |
| Nicholson 2020^20^ | HM JACKarc | CRC | 4 | 3 | 2 | 5795 | 40 | 0.006903 | 150 | 24 | 5669 | 16 | 86 | 60 (58.7,61.3) | 98.5 (98.2,98.8) |
| Tang 2022^21^ | HM-JACKarc system | CRC | 4 | 0 | 1 | 603 | 20 | 0.033167 | 10 | 18 | 485 | 2 | 98 | 90 (87.6,92.4) | 83.2 (80.2,86.2) |
| Tang 2022^21^ | HM-JACKarc system | CRC | 4 | 1 - IDA | 1 | 78 | 1 | 0.012821 | 10 | 1 | 59 | 0 | 18 | 100 (NE,NE) | 76.6 (67.2,86) |
| Turvill 2018^22^ | HM-JACKarc- single FIT | CRC | 2 | 0 | unclear | 505 | 27 | 0.053465 | 12 | 23 | 423 | 4 | 55 | 84.6 (81.5,87.7) | 88.5 (85.7,91.3) |
| Turvill 2021^23^ | HM JACKarc | CRC | 4 | 0 | unclear | 5040 | 151 | 0.02996 | 2 | 140 | 2970 | 11 | 1919 | 92.7 (92,93.4) | 60.7 (59.4,62) |
| Turvill 2021^23^ | HM JACKarc | CRC | 4 | 0 | unclear | 5040 | 151 | 0.02996 | 10 | 132 | 3956 | 19 | 933 | 87.4 (86.5,88.3) | 80.9 (79.8,82) |
| Turvill 2021^23^ | HM JACKarc | CRC | 4 | 3 | unclear | 2798 | 62 | 0.022159 | 16 | 54 | 2342 | 8 | 394 | 87.1 (85.9,88.3) | 85.6 (84.3,86.9) |
| Turvill 2021^23^ | HM JACKarc | CRC | 4 | 0 | unclear | 5040 | 151 | 0.02996 | 19 | 129 | 4165 | 22 | 724 | 85.4 (84.4,86.4) | 85.2 (84.2,86.2) |
| Turvill 2021^23^ | HM JACKarc | CRC | 4 | 7 - â‰¥60 years | unclear | 3823 | 121 | 0.031651 | 19 | 101 | 3162 | 20 | 540 | 83.5 (82.3,84.7) | 85.4 (84.3,86.5) |
| Turvill 2021^23^ | HM JACKarc | CRC | 4 | 5 - Drug use (antiplatelets, anticoagulants NSAIDs) | unclear | 1356 | 51 | 0.037611 | 19 | 42 | 1051 | 9 | 254 | 82.4 (80.4,84.4) | 80.5 (78.4,82.6) |
| Turvill 2021^23^ | HM JACKarc | CRC | 4 | 2 | unclear | 2242 | 89 | 0.039697 | 21 | 76 | 1802 | 13 | 351 | 85.4 (83.9,86.9) | 83.7 (82.2,85.2) |
| Turvill 2021^23^ | HM JACKarc | CRC | 4 | 1 - IDA | unclear | 559 | 34 | 0.060823 | 21 | 28 | 428 | 6 | 97 | 82.4 (79.2,85.6) | 81.5 (78.3,84.7) |
| Turvill 2021^23^ | HM JACKarc | CRC | 4 | 0 | unclear | 5040 | 151 | 0.02996 | 30 | 121 | 4288 | 30 | 601 | 80.1 (79,81.2) | 87.7 (86.8,88.6) |
| Turvill 2021^23^ | HM JACKarc | CRC | 4 | 1- non-IDA | unclear | 544 | 25 | 0.045956 | 30 | 23 | 444 | 2 | 75 | 92 (89.7,94.3) | 85.5 (82.5,88.5) |
| Turvill 2021^23^ | HM JACKarc | CRC | 4 | 7 - <60 years | unclear | 1217 | 30 | 0.024651 | 37 | 27 | 1037 | 3 | 150 | 90 (88.3,91.7) | 87.4 (85.5,89.3) |
| Turvill 2021^23^ | HM JACKarc | CRC | 4 | 0 | unclear | 5040 | 151 | 0.02996 | 100 | 100 | 4532 | 51 | 357 | 66.2 (64.9,67.5) | 92.7 (92,93.4) |
| Turvill 2021^23^ | HM JACKarc | CRC | 4 | 0 | unclear | 5040 | 151 | 0.02996 | 300 | 80 | 4649 | 71 | 240 | 53 (51.6,54.4) | 95.1 (94.5,95.7) |
| Withrow 2022^24^ | HM JACKarc | CRC | 3 | 0 | 2 | 11142 | 89 | 0.007988 | 2 | 85 | 9209 | 4 | 1844 | 95.5 (95.1,95.9) | 83.3 (82.6,84) |
| Withrow 2022^24^ | HM JACKarc | CRC | 4 | 0 | 2 | 16604 | 139 | 0.008371 | 2 | 134 | 13752 | 5 | 2713 | 96.4 (96.1,96.7) | 83.5 (82.9,84.1) |
| Withrow 2022^24^ | HM JACKarc | CRC | 4 | 2 | 2 | 7019 | 83 | 0.011825 | 2 | 80 | 5678 | 3 | 1258 | 96.4 (96,96.8) | 81.9 (81,82.8) |
| Withrow 2022^24^ | HM JACKarc | CRC | 4 | 3 | 2 | 9585 | 57 | 0.005947 | 2 | 54 | 8074 | 2 | 1455 | 96.4 (96,96.8) | 84.7 (84,85.4) |
| Withrow 2022^24^ | HM JACKarc | CRC | 4 | 7 age <40 years | 2 | 1390 | 9 | 0.006475 | 2 | 9 | 1231 | 0 | 150 | 100 (NE,NE) | 89.1 (87.5,90.7) |
| Withrow 2022^24^ | HM JACKarc | CRC | 4 | 7 age >40 years | 2 | 15214 | 130 | 0.008545 | 2 | 125 | 12521 | 5 | 2563 | 96.2 (95.9,96.5) | 83 (82.4,83.6) |
| Withrow 2022^24^ | HM JACKarc | CRC | 4 | 7 age >50 years | 2 | 12936 | 118 | 0.009122 | 2 | 113 | 10483 | 5 | 2335 | 95.8 (95.5,96.1) | 81.8 (81.1,82.5) |
| Withrow 2022^24^ | HM JACKarc | CRC | 4 | 7 age >60 years | 2 | 8755 | 98 | 0.011194 | 2 | 93 | 6823 | 5 | 1834 | 94.9 (94.4,95.4) | 78.8 (77.9,79.7) |
| Withrow 2022^24^ | HM JACKarc | CRC | 4 | 7 age >70 years | 2 | 3043 | 77 | 0.025304 | 2 | 73 | 1536 | 4 | 1430 | 94.8 (94,95.6) | 51.8 (50,53.6) |
| Withrow 2022^24^ | HM JACKarc | CRC | 4 | 7 age >80 years | 2 | 2527 | 41 | 0.016225 | 2 | 39 | 1701 | 2 | 785 | 95.1 (94.3,95.9) | 68.4 (66.6,70.2) |
| Withrow 2022^24^ | HM JACKarc | CRC | 3 | 0 | 2 | 11142 | 89 | 0.007988 | 10 | 82 | 10142 | 7 | 911 | 92.1 (91.6,92.6) | 91.8 (91.3,92.3) |
| Withrow 2022^24^ | HM JACKarc | CRC | 4 | 0 | 2 | 16604 | 139 | 0.008371 | 10 | 128 | 15064 | 11 | 1401 | 92.1 (91.7,92.5) | 91.5 (91.1,91.9) |
| Withrow 2022^24^ | HM JACKarc | CRC | 4 | 2 | 2 | 7019 | 83 | 0.011825 | 10 | 77 | 6262 | 6 | 674 | 92.8 (92.2,93.4) | 90.3 (89.6,91) |
| Withrow 2022^24^ | HM JACKarc | CRC | 4 | 3 | 2 | 9585 | 57 | 0.005947 | 10 | 51 | 8802 | 5 | 727 | 91.1 (90.5,91.7) | 92.4 (91.9,92.9) |
| Withrow 2022^24^ | HM JACKarc | CRC | 4 | 1 - Low Haemoglobin (<130 g/L in men, <120g/L in women) | 2 | 5076 | 72 | 0.014184 | 10 | 69 | 4404 | 3 | 600 | 95.8 (95.2,96.4) | 88 (87.1,88.9) |
| Withrow 2022^24^ | HM JACKarc | CRC | 4 | 1 & 2 | 2 | 2091 | 46 | 0.021999 | 10 | 43 | 1749 | 3 | 296 | 93.5 (92.4,94.6) | 85.5 (84,87) |
| Withrow 2022^24^ | HM JACKarc | CRC | 4 | 1 & 2 | 2 | 1141 | 36 | 0.031551 | 10 | 33 | 914 | 3 | 191 | 91.7 (90.1,93.3) | 82.7 (80.5,84.9) |
| Withrow 2022^24^ | HM JACKarc | CRC | 4 | 1 & 2 | 2 | 494 | 23 | 0.046559 | 10 | 22 | 372 | 1 | 99 | 95.7 (93.9,97.5) | 79 (75.4,82.6) |
| Withrow 2022^24^ | HM JACKarc | CRC | 4 | 1 & 2 | 2 | 216 | 14 | 0.064815 | 10 | 14 | 146 | 0 | 56 | 100 (NE,NE) | 72.3 (66.3,78.3) |
| Withrow 2022^24^ | HM JACKarc | CRC | 4 | 1 & 2 | 2 | 89 | 9 | 0.101124 | 10 | 9 | 57 | 0 | 23 | 100 (NE,NE) | 71.2 (61.8,80.6) |
| Withrow 2022^24^ | HM JACKarc | CRC | 4 | 1 &3 | 2 | 2758 | 25 | 0.009065 | 10 | 25 | 2444 | 0 | 289 | 100 (NE,NE) | 89.4 (88.3,90.5) |
| Withrow 2022^24^ | HM JACKarc | CRC | 4 | 1 &3 | 2 | 1297 | 13 | 0.010023 | 10 | 13 | 1130 | 0 | 154 | 100 (NE,NE) | 88 (86.2,89.8) |
| Withrow 2022^24^ | HM JACKarc | CRC | 4 | 1 &3 | 2 | 491 | 6 | 0.01222 | 10 | 6 | 410 | 0 | 75 | 100 (NE,NE) | 84.5 (81.3,87.7) |
| Withrow 2022^24^ | HM JACKarc | CRC | 4 | 1 &3 | 2 | 189 | 3 | 0.015873 | 10 | 3 | 148 | 0 | 38 | 100 (NE,NE) | 79.6 (73.9,85.3) |
| Withrow 2022^24^ | HM JACKarc | CRC | 4 | 7 age <40 years | 2 | 1390 | 9 | 0.006475 | 10 | 8 | 1290 | 1 | 91 | 88.9 (87.2,90.6) | 93.4 (92.1,94.7) |
| Withrow 2022^24^ | HM JACKarc | CRC | 4 | 7 age >40 years | 2 | 15214 | 130 | 0.008545 | 10 | 120 | 13774 | 10 | 1310 | 92.3 (91.9,92.7) | 91.3 (90.9,91.7) |
| Withrow 2022^24^ | HM JACKarc | CRC | 4 | 7 age >50 years | 2 | 12936 | 118 | 0.009122 | 10 | 108 | 11629 | 10 | 1189 | 91.5 (91,92) | 90.7 (90.2,91.2) |
| Withrow 2022^24^ | HM JACKarc | CRC | 4 | 7 age >60 years | 2 | 8755 | 98 | 0.011194 | 10 | 88 | 7705 | 10 | 952 | 89.8 (89.2,90.4) | 89 (88.3,89.7) |
| Withrow 2022^24^ | HM JACKarc | CRC | 4 | 7 age >70 years | 2 | 5863 | 77 | 0.013133 | 10 | 69 | 5038 | 8 | 748 | 89.6 (88.8,90.4) | 87.1 (86.2,88) |
| Withrow 2022^24^ | HM JACKarc | CRC | 4 | 7 age >80 years | 2 | 2533 | 41 | 0.016186 | 10 | 36 | 2071 | 5 | 421 | 87.8 (86.5,89.1) | 83.1 (81.6,84.6) |
| **OC-Sensor** | | | | | | | | | | | | | | | |
| Archer 2022^25^ | OC-Sensor in other Sheffield article, Ball 2022 - but NR for this study | CRC | 4 | 0 | 1 | 166 | 11 | 0.066265 | 10 | 10 | 52 | 1 | 103 | 90.9 (86.5,95.3) | 33.6 (26.4,40.8) |
| Archer 2022^25^ | OC-Sensor in other Sheffield article, Ball 2022 - but NR for this study | CRC | 4 | 0 | 1 | 166 | 11 | 0.066265 | 60 | 6 | 115 | 5 | 40 | 54.6 (47,62.2) | 74.2 (67.5,80.9) |
| Archer 2022^25^ | OC-Sensor in other Sheffield article, Ball 2022 - but NR for this study | CRC | 4 | 0 | 1 | 166 | 11 | 0.066265 | 100 | 6 | 122 | 5 | 33 | 54.6 (47,62.2) | 78.7 (72.5,84.9) |
| Ayling 2019^26^ | OC-Sensor (analyser NR) | CRC | 4 | 1 - low haemoglobin (not defined) | 1 | 178 | 7 | 0.039326 | 10 | 5 | 164 | 2 | 7 | 71.4 (64.8,78) | 95.9 (93,98.8) |
| Ayling 2019^26^ | OC-Sensor (analyser NR) | CRC | 4 | 1 - IDA | 1 | 137 | 6 | 0.043796 | 10 | 4 | 125 | 2 | 6 | 68.7 (60.9,76.5) | 95.4 (91.9,98.9) |
| Ball 2022^27^ | OC-Sensor Pledia analyser | CRC | 4 | 0 | 2 | 3506 | 45 | 0.012835 | 10 | 41 | 2794 | 4 | 667 | 91.1 (90.2,92) | 80.7 (79.4,82) |
| Ball 2022^27^ | OC-Sensor Pledia analyser | CRC | 4 | 2 | 2 | 1566 | 25 | 0.015964 | 10 | 21 | 1220 | 4 | 321 | 84 (82.2,85.8) | 79.2 (77.2,81.2) |
| Ball 2022^27^ | OC-Sensor Pledia analyser | CRC | 4 | 3 | 2 | 1940 | 20 | 0.010309 | 10 | 20 | 1574 | 0 | 346 | 100 (NE,NE) | 82 (80.3,83.7) |
| Ball 2022^27^ | OC-Sensor Pledia analyser | CRC | 4 | 0 | 2 | 3506 | 45 | 0.012835 | 20 | 39 | 3021 | 6 | 440 | 86.7 (85.6,87.8) | 87.3 (86.2,88.4) |
| Ball 2022^27^ | OC-Sensor Pledia analyser | CRC | 4 | 2 | 2 | 1566 | 25 | 0.015964 | 20 | 20 | 1008 | 5 | 533 | 80 (78,82) | 65.4 (63,67.8) |
| Ball 2022^27^ | OC-Sensor Pledia analyser | CRC | 4 | 3 | 2 | 1940 | 20 | 0.010309 | 20 | 19 | 1705 | 1 | 215 | 95 (94,96) | 88.8 (87.4,90.2) |
| Ball 2022^27^ | OC-Sensor Pledia analyser | CRC | 4 | 0 | 2 | 3506 | 45 | 0.012835 | 50 | 33 | 3217 | 12 | 244 | 73.3 (71.8,74.8) | 93 (92.2,93.8) |
| Ball 2022^27^ | OC-Sensor Pledia analyser | CRC | 4 | 2 | 2 | 1566 | 25 | 0.015964 | 50 | 17 | 1412 | 8 | 129 | 68 (65.7,70.3) | 91.6 (90.2,93) |
| Ball 2022^27^ | OC-Sensor Pledia analyser | CRC | 4 | 3 | 2 | 1940 | 20 | 0.010309 | 50 | 16 | 1807 | 4 | 113 | 80 (78.2,81.8) | 94.1 (93.1,95.1) |
| Ball 2022^27^ | OC-Sensor Pledia analyser | CRC | 4 | 0 | 2 | 3506 | 45 | 0.012835 | 80 | 30 | 3287 | 15 | 174 | 66.7 (65.1,68.3) | 95 (94.3,95.7) |
| Ball 2022^27^ | OC-Sensor Pledia analyser | CRC | 4 | 2 | 2 | 1566 | 25 | 0.015964 | 80 | 16 | 1447 | 9 | 94 | 64 (61.6,66.4) | 93.9 (92.7,95.1) |
| Ball 2022^27^ | OC-Sensor Pledia analyser | CRC | 4 | 3 | 2 | 1940 | 20 | 0.010309 | 80 | 14 | 1839 | 6 | 81 | 70 (68,72) | 95.8 (94.9,96.7) |
| Ball 2022^27^ | OC-Sensor Pledia analyser | CRC | 4 | 0 | 2 | 3506 | 45 | 0.012835 | 100 | 30 | 3315 | 15 | 146 | 66.7 (65.1,68.3) | 95.8 (95.1,96.5) |
| Ball 2022^27^ | OC-Sensor Pledia analyser | CRC | 4 | 2 | 2 | 1566 | 25 | 0.015964 | 100 | 16 | 1458 | 9 | 83 | 64 (61.6,66.4) | 94.6 (93.5,95.7) |
| Ball 2022^27^ | OC-Sensor Pledia analyser | CRC | 4 | 3 | 2 | 1940 | 20 | 0.010309 | 100 | 14 | 1857 | 6 | 63 | 70 (68,72) | 96.7 (95.9,97.5) |
| Ball 2022^27^ | OC-Sensor Pledia analyser | CRC | 4 | 0 | 2 | 3506 | 45 | 0.012835 | 120 | 28 | 3330 | 17 | 131 | 62.2 (60.6,63.8) | 96.2 (95.6,96.8) |
| Ball 2022^27^ | OC-Sensor Pledia analyser | CRC | 4 | 2 | 2 | 1566 | 25 | 0.015964 | 120 | 15 | 1467 | 10 | 74 | 60 (57.6,62.4) | 95.2 (94.1,96.3) |
| Ball 2022^27^ | OC-Sensor Pledia analyser | CRC | 4 | 3 | 2 | 1940 | 20 | 0.010309 | 120 | 13 | 1862 | 7 | 58 | 65 (62.9,67.1) | 97 (96.2,97.8) |
| Ball 2022^27^ | OC-Sensor Pledia analyser | CRC | 4 | 0 | 2 | 3506 | 45 | 0.012835 | 150 | 24 | 3461 | 21 | 107 | 53.3 (51.6,55) | 97 (96.4,97.6) |
| Ball 2022^27^ | OC-Sensor Pledia analyser | CRC | 4 | 2 | 2 | 1566 | 25 | 0.015964 | 150 | 13 | 1486 | 12 | 55 | 52 (49.5,54.5) | 96.4 (95.5,97.3) |
| Ball 2022^27^ | OC-Sensor Pledia analyser | CRC | 4 | 3 | 2 | 1940 | 20 | 0.010309 | 150 | 11 | 1868 | 9 | 52 | 55 (52.8,57.2) | 97.3 (96.6,98) |
| Ball 2022^27^ (personal communicaton) | OC-Sensor Pledia analyser | CRC | 3 | 0 | 2 | 2892 | 17 | 0.005878 | 10 | 16 | 2458 | 1 | 417 | 94.1 (93.2,95) | 85.5 (84.2,86.8) |
| Ball 2022^27^ (personal communicaton) | OC-Sensor Pledia analyser | CRC | 3 | 2 | 2 | 1286 | 11 | 0.008554 | 10 | 10 | 1074 | 1 | 201 | 90.9 (89.3,92.5) | 84.2 (82.2,86.2) |
| Ball 2022^27^ (personal communicaton) | OC-Sensor Pledia analyser | CRC | 3 | 3 | 2 | 1606 | 6 | 0.003736 | 10 | 6 | 1384 | 0 | 216 | 100 (NE,NE) | 86.5 (84.8,88.2) |
| Ball 2022^27^ (personal communicaton) | OC-Sensor Pledia analyser | CRC | 3 | 0 | 2 | 2892 | 17 | 0.005878 | 20 | 15 | 2623 | 2 | 252 | 88.2 (87,89.4) | 91.2 (90.2,92.2) |
| Ball 2022^27^ (personal communicaton) | OC-Sensor Pledia analyser | CRC | 3 | 2 | 2 | 1286 | 11 | 0.008554 | 20 | 9 | 1139 | 2 | 136 | 81.8 (79.7,83.9) | 89.3 (87.6,91) |
| Ball 2022^27^ (personal communicaton) | OC-Sensor Pledia analyser | CRC | 3 | 3 | 2 | 1606 | 6 | 0.003736 | 20 | 6 | 1485 | 0 | 115 | 100 (NE,NE) | 92.8 (91.5,94.1) |
| Ball 2022^27^ (personal communicaton) | OC-Sensor Pledia analyser | CRC | 3 | 0 | 2 | 2892 | 17 | 0.005878 | 50 | 11 | 2736 | 6 | 139 | 64.7 (63,66.4) | 95.2 (94.4,96) |
| Ball 2022^27^ (personal communicaton) | OC-Sensor Pledia analyser | CRC | 3 | 2 | 2 | 1286 | 11 | 0.008554 | 50 | 7 | 1196 | 4 | 79 | 63.6 (61,66.2) | 93.8 (92.5,95.1) |
| Ball 2022^27^ (personal communicaton) | OC-Sensor Pledia analyser | CRC | 3 | 3 | 2 | 1606 | 6 | 0.003736 | 50 | 4 | 1541 | 2 | 59 | 66.7 (64.4,69) | 96.3 (95.4,97.2) |
| Ball 2022^27^ (personal communicaton) | OC-Sensor Pledia analyser | CRC | 3 | 0 | 2 | 2892 | 17 | 0.005878 | 80 | 10 | 2778 | 7 | 97 | 58.8 (57,60.6) | 96.6 (95.9,97.3) |
| Ball 2022^27^ (personal communicaton) | OC-Sensor Pledia analyser | CRC | 3 | 2 | 2 | 1286 | 11 | 0.008554 | 80 | 7 | 1218 | 4 | 57 | 63.6 (61,66.2) | 95.5 (94.4,96.6) |
| Ball 2022^27^ (personal communicaton) | OC-Sensor Pledia analyser | CRC | 3 | 3 | 2 | 1606 | 6 | 0.003736 | 80 | 3 | 1560 | 3 | 40 | 50 (47.6,52.4) | 97.5 (96.7,98.3) |
| Ball 2022^27^ (personal communicaton) | OC-Sensor Pledia analyser | CRC | 3 | 0 | 2 | 2892 | 17 | 0.005878 | 100 | 10 | 2797 | 7 | 78 | 58.8 (57,60.6) | 97.3 (96.7,97.9) |
| Ball 2022^27^ (personal communicaton) | OC-Sensor Pledia analyser | CRC | 3 | 2 | 2 | 1286 | 11 | 0.008554 | 100 | 7 | 1227 | 4 | 48 | 63.6 (61,66.2) | 96.2 (95.2,97.2) |
| Ball 2022^27^ (personal communicaton) | OC-Sensor Pledia analyser | CRC | 3 | 3 | 2 | 1606 | 6 | 0.003736 | 100 | 3 | 1571 | 3 | 29 | 50 (47.6,52.4) | 98.2 (97.5,98.9) |
| Ball 2022^27^ (personal communicaton) | OC-Sensor Pledia analyser | CRC | 3 | 0 | 2 | 2892 | 17 | 0.005878 | 120 | 10 | 2807 | 7 | 68 | 58.8 (57,60.6) | 97.6 (97,98.2) |
| Ball 2022^27^ (personal communicaton) | OC-Sensor Pledia analyser | CRC | 3 | 2 | 2 | 1286 | 11 | 0.008554 | 120 | 7 | 1233 | 4 | 42 | 63.6 (61,66.2) | 96.7 (95.7,97.7) |
| Ball 2022^27^ (personal communicaton) | OC-Sensor Pledia analyser | CRC | 3 | 3 | 2 | 1606 | 6 | 0.003736 | 120 | 3 | 1574 | 3 | 26 | 50 (47.6,52.4) | 98.4 (97.8,99) |
| Ball 2022^27^ (personal communicaton) | OC-Sensor Pledia analyser | CRC | 3 | 0 | 2 | 2892 | 17 | 0.005878 | 150 | 9 | 2827 | 8 | 48 | 52.9 (51.1,54.7) | 98.3 (97.8,98.8) |
| Ball 2022^27^ (personal communicaton) | OC-Sensor Pledia analyser | CRC | 3 | 2 | 2 | 1286 | 11 | 0.008554 | 150 | 6 | 1247 | 5 | 28 | 54.5 (51.8,57.2) | 97.8 (97,98.6) |
| Ball 2022^27^ (personal communicaton) | OC-Sensor Pledia analyser | CRC | 3 | 3 | 2 | 1606 | 6 | 0.003736 | 150 | 3 | 1581 | 3 | 19 | 50 (47.6,52.4) | 98.8 (98.3,99.3) |
| Benton 2022^1^ | OC Sensor PLEDIA | CRC | 2 | 0 | 1 | 233 | 7 | 0.030043 | 1 | 5 | 103 | 2 | 123 | 71.4 (65.6,77.2) | 45.6 (39.2,52) |
| Benton 2022^1^ | OC Sensor PLEDIA | CRC | 2 | 0 | 1 | 233 | 7 | 0.030043 | 10 | 5 | 194 | 2 | 32 | 71.4 (65.6,77.2) | 85.8 (81.3,90.3) |
| Benton 2022^1^ | OC Sensor PLEDIA | CRC | 2 | 0 | 1 | 233 | 7 | 0.030043 | 100 | 4 | 218 | 3 | 8 | 57.1 (50.7,63.5) | 96.5 (94.1,98.9) |
| Bujanda 2018^28^ | OC-Sensor (analyser NR) | CRC | 4 | 5 - with aspirin | 1 | 485 | 51 | 0.105155 | 20 | 45 | 291 | 6 | 143 | 88 (85.1,90.9) | 67 (62.8,71.2) |
| Bujanda 2018^28^ | OC-Sensor (analyser NR) | CRC | 4 | 5 - without aspirin | 1 | 2567 | 299 | 0.116478 | 20 | 275 | 1610 | 24 | 658 | 92 (91,93) | 71 (69.2,72.8) |
| Cama 2022^29^ | OC-Sensor iO | CRC | 1 | 0 | 2 | 5341 | 74 | 0.013855 | 4 | 69 | 2950 | 5 | 2317 | 93 (92.3,93.7) | 56 (54.7,57.3) |
| Cama 2022^29^ | OC-Sensor iO | CRC | 1 | 0 | 2 | 5341 | 74 | 0.013855 | 10 | 67 | 4108 | 7 | 1159 | 90.5 (89.7,91.3) | 78 (76.9,79.1) |
| Cama 2022^29^ | OC-Sensor iO | CRC | 1 | 0 | 2 | 5341 | 74 | 0.013855 | 100 | 53 | 5004 | 21 | 263 | 71.6 (70.4,72.8) | 95 (94.4,95.6) |
| Chapman 2021^4^ | OC-Sensor DIANA | CRC | 4 | 0 | 1 | 732 | 38 | 0.051913 | 4 | 37 | 444 | 1 | 250 | 97 (95.8,98.2) | 64 (60.5,67.5) |
| Chapman 2021^4^ | OC-Sensor DIANA | CRC | 4 | 0 | 1 | 732 | 38 | 0.051913 | 10 | 34 | 514 | 4 | 180 | 89 (86.7,91.3) | 74 (70.8,77.2) |
| Chapman 2021^4^ | OC-Sensor DIANA | CRC | 4 | 0 | 1 | 732 | 38 | 0.051913 | 18.2 | 33 | 548 | 5 | 146 | 87 (84.6,89.4) | 79 (76,82) |
| Chapman 2021^4^ | OC-Sensor DIANA | CRC | 4 | 0 | 1 | 732 | 38 | 0.051913 | 150 | 24 | 652 | 14 | 42 | 63 (59.5,66.5) | 94 (92.3,95.7) |
| Crooks 2023^30^ | OC-Sensor iO (Bailey 2021a) | CRC | 1 | 0 | 2 | 33694 | 514 | 0.015255 | 4 | 488 | 20461 | 26 | 12719 | 94.9 (94.7,95.1) | 61.7 (61.2,62.2) |
| Crooks 2023^30^ | OC-Sensor iO (Bailey 2021a) | CRC | 1 | 0 | 2 | 33694 | 514 | 0.015255 | 10 | 461 | 26004 | 53 | 7176 | 89.7 (89.4,90) | 78.4 (78,78.8) |
| Crooks 2023^30^ | OC-Sensor iO (Bailey 2021a) | CRC | 1 | 0 | 2 | 33694 | 514 | 0.015255 | 20 | 437 | 28353 | 77 | 4827 | 85 (84.6,85.4) | 85.5 (85.1,85.9) |
| Crooks 2023^30^ | OC-Sensor iO (Bailey 2021a) | CRC | 1 | 0 | 2 | 33694 | 514 | 0.015255 | 40 | 396 | 30061 | 118 | 3119 | 77 (76.6,77.4) | 90.6 (90.3,90.9) |
| Crooks 2023^30^ | OC-Sensor iO (Bailey 2021a) | CRC | 1 | 0 | 2 | 33694 | 514 | 0.015255 | 100 | 329 | 31552 | 185 | 1628 | 64 (63.5,64.5) | 95.1 (94.9,95.3) |
| Georgiou Delisle 2022^31^ | OC Sensor iO | CRC | 1 | 0 | 2 | 4187 | 61 | 0.014569 | 4 | 59 | 1622 | 2 | 2504 | 96.7 (96.2,97.2) | 39.3 (37.8,40.8) |
| Georgiou Delisle 2022^31^ | OC Sensor iO | CRC | 1 | 0 | 2 | 4187 | 61 | 0.014569 | 9.5 | 58 | 3099 | 3 | 1027 | 95.1 (94.4,95.8) | 75.1 (73.8,76.4) |
| Georgiou Delisle 2022^31^ | OC Sensor iO | CRC | 1 | 0 | 2 | 4187 | 61 | 0.014569 | 150 | 40 | 3874 | 21 | 252 | 65.6 (64.2,67) | 93.9 (93.2,94.6) |
| Juul 2018^32^ | OC-Sensor DIANA | CRC | 4 | 0 | 2 | 3462 | 54 | 0.015598 | 10 | 51 | 2919 | 3 | 489 | 94.4 (93.6,95.2) | 85.7 (84.5,86.9) |
| Juul 2018^32^ | OC-Sensor DIANA | CRC | 4 | 1 | 2 | 424 | 49 | 0.115566 | 10 | 10 | 298 | 39 | 77 | 20.4 (16.6,24.2) | 79.5 (75.7,83.3) |
| Laszlo 2021^33^ | OC-Sensor iO | CRC | 4 | 0 | 2 | 3596 | 90 | 0.025028 | 4 | 79 | 2558 | 11 | 948 | 87.8 (86.7,88.9) | 73 (71.5,74.5) |
| Laszlo 2021^33^ | OC-Sensor iO | CRC | 4 | 0 | 2 | 3596 | 90 | 0.025028 | 6 | 78 | 2664 | 12 | 842 | 86.7 (85.6,87.8) | 76.1 (74.7,77.5) |
| Laszlo 2021^33^ | OC-Sensor iO | CRC | 4 | 0 | 2 | 3596 | 90 | 0.025028 | 10 | 75 | 2807 | 15 | 699 | 83.3 (82.1,84.5) | 80.1 (78.8,81.4) |
| Laszlo 2021^33^ | OC-Sensor iO | CRC | 4 | 0 | 2 | 3596 | 90 | 0.025028 | 20 | 73 | 2997 | 17 | 509 | 81.1 (79.8,82.4) | 85.5 (84.3,86.7) |
| Laszlo 2021^33^ | OC-Sensor iO | CRC | 4 | 0 | 2 | 3596 | 90 | 0.025028 | 50 | 67 | 3209 | 23 | 297 | 74.4 (73,75.8) | 91.6 (90.7,92.5) |
| Laszlo 2021^33^ | OC-Sensor iO | CRC | 4 | 0 | 2 | 3596 | 90 | 0.025028 | 80 | 61 | 3269 | 29 | 237 | 67.8 (66.3,69.3) | 93.3 (92.5,94.1) |
| Laszlo 2021^33^ | OC-Sensor iO | CRC | 4 | 0 | 2 | 3596 | 90 | 0.025028 | 100 | 58 | 3298 | 32 | 208 | 64.4 (62.8,66) | 94.1 (93.3,94.9) |
| Laszlo 2021^33^ | OC-Sensor iO | CRC | 4 | 0 | 2 | 3596 | 90 | 0.025028 | 120 | 55 | 3319 | 35 | 187 | 61.1 (59.5,62.7) | 94.7 (94,95.4) |
| Laszlo 2021^33^ | OC-Sensor iO | CRC | 4 | 0 | 2 | 3596 | 90 | 0.025028 | 150 | 52 | 3335 | 38 | 171 | 57.8 (56.2,59.4) | 95.2 (94.5,95.9) |
| Laszlo 2021^33^ | OC-Sensor iO | CRC | 4 | 0 | 2 | 3596 | 90 | 0.025028 | 200 | 49 | 3352 | 41 | 154 | 54.4 (52.8,56) | 95.7 (95,96.4) |
| Maclean 2021a^34^ | OC-Sensor PLEDIA | CRC | 4 | 0 | 2 | 358 | 12 | 0.03352 | 10 | 12 | 246 | 0 | 100 | 100 (NE,NE) | 71.1 (66.4,75.8) |
| Maclean 2021a^34^ | OC-Sensor PLEDIA | CRC | 4 | 0 | 2 | 358 | 12 | 0.03352 | 150 | 5 | 318 | 7 | 28 | 41.7 (36.6,46.8) | 91.9 (89.1,94.7) |
| Morales-Arraez 2018^35^ | OC-Sensor | CRC | 4 | 1 - Hb<11.9 g/dL in men and Hb<10.9 g/dL in women, and ferritin â‰¤ 30 g/dL | 1 | 245 | 28 | 0.114286 | 10 | 26 | 124 | 2 | 93 | 92.9 (89.7,96.1) | 57.1 (50.9,63.3) |
| Mowat 2016^36^ | OC-Sensor io | CRC | 4 | 0 | 1 | 750 | 28 | 0.037333 | 4 | 28 | 313 | 0 | 409 | 100 (NE,NE) | 43.4 (39.9,46.9) |
| Mowat 2016^36^ | OC-Sensor io | CRC | 4 | 0 | 1 | 750 | 28 | 0.037333 | 10 | 25 | 571 | 3 | 151 | 89.3 (87.1,91.5) | 79.1 (76.2,82) |
| Pin Vieto 2021^37^ | OC-Sensor analyser NR | CRC | 4 | 0 | 2 | 4543 | 73 | 0.016069 | 10 | 59 | 3728 | 14 | 742 | 80.6 (79.5,81.7) | 83.4 (82.3,84.5) |
| Pin Vieto 2021^37^ | OC-Sensor analyser NR | CRC | 4 | 0 | 2 | 4543 | 73 | 0.016069 | 20 | 57 | 3916 | 16 | 554 | 77.8 (76.6,79) | 87.6 (86.6,88.6) |
| Rodriguez-Alonso 2018^38^ | OC-Sensor MICRO | CRC | 4 | 5 - PPI users | 1 | 525 | 15 | 0.028571 | 20 | 14 | 434 | 1 | 76 | 93.3 (91.2,95.4) | 85.1 (82.1,88.1) |
| Rodriguez-Alonso 2018^38^ | OC-Sensor MICRO | CRC | 4 | 5 - PPI non-users | 1 | 477 | 15 | 0.031447 | 20 | 14 | 404 | 1 | 58 | 93.3 (91.1,95.5) | 87.4 (84.4,90.4) |
| Rodriguez-Alonso 2020^39^ | OC-Sensor MICRO desktop analyser | CRC | 4 - referred to colonoscopy in Spain | 1 iron deficiency anaemia | 1 | 120 | 9 | 0.075 | 15 | 9 | 86 | 0 | 25 | 100 (NE,NE) | 77.5 (70,85) |
| **QuikRead go** | | | | | | | | | | | | | | | |
| Maclean 2021b^40^ | QuikRead go | CRC | 2 | 0 | 2 | 553 | 14 | 0.025316 | 10 | 13 | 378 | 1 | 161 | 92.9 (90.8,95) | 70.1 (66.3,73.9) |
| Maclean 2021b^40^ | QuikRead go | CRC | 2 | 0 | 2 | 553 | 14 | 0.025316 | 100 | 10 | 510 | 4 | 29 | 71.4 (67.6,75.2) | 94.6 (92.7,96.5) |
| Maclean 2021b^40^ | QuikRead go | CRC | 2 | 0 | 2 | 553 | 14 | 0.025316 | 150 | 8 | 517 | 6 | 22 | 57.1 (53,61.2) | 95.9 (94.2,97.6) |
| Tsapournas 2020^41^ | QuikRead go (inc referrals from primary and secondary care) | CRC | 4 | 0 | 1 | 242 | 13 | 0.053719 | 10 | 12 | 177 | 1 | 52 | 92.3 (88.9,95.7) | 77.3 (72,82.6) |
| Tsapournas 2020^41^ | QuikRead go (inc referrals from primary and secondary care) | CRC | 4 | 0 | 1 | 242 | 13 | 0.053719 | 15 | 12 | 187 | 1 | 42 | 92.3 (88.9,95.7) | 81.7 (76.8,86.6) |
| Tsapournas 2020^41^ | QuikRead go (inc referrals from primary and secondary care) | CRC | 4 | 0 | 1 | 242 | 13 | 0.053719 | 20 | 11 | 198 | 2 | 31 | 84.6 (80.1,89.1) | 86.5 (82.2,90.8) |
| **IDK** | | | | | | | | | | | | | | | |
| Sieg 1999^42^ | IDK | CRC | 4 | 0 | 1 | 621 | 23 | 0.037037 | 2 | 19 | 483 | 4 | 115 | 82.6 (79.6,85.6) | 80.8 (77.7,83.9) |
| Sieg 1999^42^ | IDK | CRC | 4 | 0 | 1 | 621 | 23 | 0.037037 | 2 | 20 | 527 | 3 | 71 | 87 (84.4,89.6) | 88.1 (85.6,90.6) |
| **NS-Prime** | | | | | | | | | | | | | | | |
| Benton 2022^1^ | NS-Prime | CRC | 2 | 0 | 1 | 233 | 7 | 0.030043 | 3 | 6 | 72 | 1 | 154 | 85.7 (81.2,90.2) | 31.9 (25.9,37.9) |
| Benton 2022^1^ | NS-Prime | CRC | 2 | 0 | 1 | 233 | 7 | 0.030043 | 10 | 5 | 189 | 2 | 37 | 71.4 (65.6,77.2) | 83.6 (78.8,88.4) |
| Benton 2022^1^ | NS-Prime | CRC | 2 | 0 | 1 | 233 | 7 | 0.030043 | 100 | 4 | 220 | 3 | 6 | 57.1 (50.7,63.5) | 97.3 (95.2,99.4) |
| **Dual FIT, all tests** | | | | | | | | | | | | | | | |
| Gerrard 2023^13^ | DUAL FIT HM-JACKarc (either positive) | CRC | 1 | 0 | 1 | 2637 | 88 | 0.033371 | 10 | 85 | 1815 | 3 | 734 | 96.6 (95.9,97.3) | 71.2 (69.5,72.9) |
| Hunt 2022^43^ | DUAL FIT OC-Sensor (both positive) analyser NR | CRC | 4 | 0 | 2 | 28622 | 317 | 0.011075 | 10 | 290 | 23097 | 27 | 5208 | 91.5 (91.2,91.8) | 81.6 (81.2,82) |
| Hunt 2022^43^ | DUAL FIT OC-Sensor (either positive) analyser NR | CRC | 4 | 0 | 2 | 28622 | 317 | 0.011075 | 10 | 310 | 18738 | 7 | 9567 | 97.8 (97.6,98) | 66.2 (65.7,66.7) |
| Tsapournas 2020^41^ | DUAL FIT QuikRead go (either positive) (inc referrals from primary and secondary care) | CRC | 4 | 0 | 1 | 242 | 13 | 0.053719 | 10 | 13 | 164 | 0 | 65 | 100 (NE,NE) | 71.4 (65.7,77.1) |
| Tsapournas 2020^41^ | DUAL FIT QuikRead go (either positive) (inc referrals from primary and secondary care) | CRC | 4 | 0 | 1 | 242 | 13 | 0.053719 | 15 | 12 | 176 | 1 | 53 | 92.3 (88.9,95.7) | 76.8 (71.5,82.1) |
| Tsapournas 2020^41^ | DUAL FIT QuikRead go (either positive) (inc referrals from primary and secondary care) | CRC | 4 | 0 | 1 | 242 | 13 | 0.053719 | 20 | 12 | 187 | 1 | 42 | 92.3 (88.9,95.7) | 81.7 (76.8,86.6) |
| Turvill 2018^22^ | dual FIT (both positive) HM-JACKarc | CRC | 2 | 0 | unclear | 476 | 27 | 0.056723 | 2 | 25 | 383 | 2 | 66 | 91.7 (89.2,94.2) | 85.2 (82,88.4) |
| Turvill 2018^22^ | dual FIT (either positive) HM-JACKarc | CRC | 2 | 0 | unclear | 476 | 27 | 0.056723 | 43 | 24 | 407 | 3 | 42 | 87.5 (84.5,90.5) | 90.7 (88.1,93.3) |

CRC, Colorectal cancer; FN, false negative; FP, False positive; No., number; Pop., population; Prev, prevalence; Pts, patients; Ref Stand, reference standard; TN, true negative; TP, true positive

**Table 2** Data entering the statistical syntheses of the diagnostic test accuracy of FITs for detection of advanced adenomas and inflammatory bowel disease

| **Author, year** | **Test** | **Out-come** | **Pop. type** | **Sub-group** | **Ref Stand** | **No. Pts** | **CRC cases** | **Prev CRC** | **Thres-hold (µg/g)** | **TP** | **TN** | **FN** | **FP** | **Sensitivity** | **Specificity** |
| --- | --- | --- | --- | --- | --- | --- | --- | --- | --- | --- | --- | --- | --- | --- | --- |
| **Advanced Adenoma** | | | | | | | | | | | | | | | |
| Sieg 1999^42^ | IDK | AA | 4 | 0 | 1 | 621 | 37 | 0.059581 | 2 | 27 | 477 | 10 | 107 | 73 (69.5,76.5) | 81.7 (78.7,84.7) |
| Sieg 1999^42^ | IDK | AA | 4 | 0 | 1 | 621 | 37 | 0.059581 | 2 | 20 | 513 | 17 | 71 | 54.1 (50.2,58) | 87.8 (85.2,90.4) |
| D'Souza 2020a^6^ | HM JACKarc analytical system | AA | 1 | 0 | 1 | 298 | 4 | 0.013423 | 2 | 2 | 216 | 2 | 78 | 50 (44.3,55.7) | 73.5 (68.5,78.5) |
| D'Souza 2020a^6^ | HM JACKarc analytical system | AA | 1 | 0 | 1 | 298 | 4 | 0.013423 | 10 | 2 | 265 | 2 | 29 | 50 (44.3,55.7) | 90.1 (86.7,93.5) |
| D'Souza 2020a^6^ | HM JACKarc analytical system | AA | 2 | 0 | 1 | 160 | 4 | 0.025 | 2 | 2 | 105 | 2 | 51 | 40 (32.4,47.6) | 67.4 (60.1,74.7) |
| D'Souza 2020a^6^ | HM JACKarc analytical system | AA | 2 | 0 | 1 | 160 | 4 | 0.025 | 10 | 2 | 139 | 2 | 17 | 40 (32.4,47.6) | 89.4 (84.6,94.2) |
| D'Souza 2020a^6^ | HM JACKarc analytical system | AA | 3 | 0 | 1 | 138 | 0 | 0 | 2 | 0 | 111 | 0 | 27 | 50 (41.7,58.3) | 80.2 (73.6,86.8) |
| D'Souza 2020a^6^ | HM JACKarc analytical system | AA | 3 | 0 | 1 | 138 | 0 | 0 | 10 | 0 | 126 | 0 | 12 | 50 (41.7,58.3) | 91.2 (86.5,95.9) |
| D'Souza 2021c^8^ | HM JACKarc analytical system | AA | 4 | 0 | 1 | 9822 | 421 | 0.042863 | 2 | 277 | 6026 | 144 | 3375 | 65.8 (64.9,66.7) | 64.1 (63.2,65) |
| D'Souza 2021c^8^ | HM JACKarc analytical system | AA | 4 | 0 | 1 | 9822 | 421 | 0.042863 | 10 | 191 | 7728 | 230 | 1673 | 45.4 (44.4,46.4) | 82.2 (81.4,83) |
| D'Souza 2021c^8^ | HM JACKarc analytical system | AA | 4 | 0 | 1 | 9822 | 421 | 0.042863 | 150 | 93 | 8743 | 328 | 658 | 22.1 (21.3,22.9) | 93 (92.5,93.5) |
| Gerrard 2023^13^ | DUAL FIT HM-JACKarc (either positive) | AA | 1 | 0 | 1 | 2637 | 97 | 0.036784 | 10 | 66 | 1788 | 31 | 752 | 68 (66.2,69.8) | 70.4 (68.7,72.1) |
| Gerrard 2023^13^ | HM-JACKarc analyser | AA | 1 | 0 | 1 | 2260 | 105 | 0.04646 | 10 | 54 | 1655 | 51 | 500 | 51.4 (49.3,53.5) | 76.8 (75.1,78.5) |
| Gerrard 2023^13^ | HM-JACKarc analyser | AA | 1 | 0 | 1 | 3426 | 136 | 0.039696 | 10 | 74 | 2514 | 62 | 776 | 54.4 (52.7,56.1) | 76.4 (75,77.8) |
| Juul 2018^32^ | OC-Sensor DIANA | AA | 4 | 0 | 2 | 3462 | 68 | 0.019642 | 10 | 62 | 2916 | 6 | 478 | 91.2 (90.3,92.1) | 85.9 (84.7,87.1) |
| MacDonald 2022^16^ | HM-JACKarc | AA | 1 | 0 | 2 | 5250 | 47 | 0.008952 | 10 | 31 | 3402 | 16 | 1801 | 63.8 (62.5,65.1) | 65.4 (64.1,66.7) |
| Maclean 2021b^40^ | QuikRead go | AA | 2 | 0 | 2 | 553 | 29 | 0.052441 | 10 | 19 | 369 | 10 | 155 | 65.5 (61.5,69.5) | 70.4 (66.6,74.2) |
| Maclean 2021b^40^ | QuikRead go | AA | 2 | 0 | 2 | 553 | 29 | 0.052441 | 100 | 6 | 491 | 23 | 33 | 20.7 (17.3,24.1) | 93.7 (91.7,95.7) |
| Maclean 2021b^40^ | QuikRead go | AA | 2 | 0 | 2 | 553 | 29 | 0.052441 | 150 | 4 | 498 | 25 | 26 | 13.8 (10.9,16.7) | 95 (93.2,96.8) |
| Mowat 2016^36^ | OC-Sensor io | AA | 4 | 0 | 1 | 750 | 40 | 0.053333 | 4 | 33 | 306 | 7 | 404 | 82.5 (79.8,85.2) | 43.1 (39.6,46.6) |
| Mowat 2016^36^ | OC-Sensor io | AA | 4 | 0 | 1 | 750 | 40 | 0.053333 | 10 | 20 | 554 | 20 | 156 | 50 (46.4,53.6) | 78 (75,81) |
| Mowat 2021^17^ & 2019^18^ | HM JACKarc | AA | 4 | 0 | 1 | 1447 | 133 | 0.091914 | 10 | 102 | 636 | 31 | 678 | 76.7 (74.5,78.9) | 48.4 (45.8,51) |
| **Inflammatory bowel disease** | | | | | | | | | | | | | | | |
| Sieg 1999^42^ | IDK | IBD | 4 | 0 | 1 | 621 | 22 | 0.035427 | 2 | 19 | 516 | 3 | 83 | 86.4 (83.7,89.1) | 86.1 (83.4,88.8) |
| Sieg 1999^42^ | IDK | IBD | 4 | 0 | 1 | 621 | 22 | 0.035427 | 2 | 16 | 524 | 6 | 75 | 72.7 (69.2,76.2) | 87.5 (84.9,90.1) |
| D'Souza 2020a^6^ | HM JACKarc analytical system | IBD | 1 | 0 | 1 | 298 | 12 | 0.040268 | 2 | 9 | 215 | 3 | 71 | 75 (70.1,79.9) | 75.2 (70.3,80.1) |
| D'Souza 2020a^6^ | HM JACKarc analytical system | IBD | 1 | 0 | 1 | 298 | 12 | 0.040268 | 10 | 8 | 249 | 4 | 37 | 66.7 (61.3,72.1) | 87.1 (83.3,90.9) |
| D'Souza 2020a^6^ | HM JACKarc analytical system | IBD | 2 | 0 | 1 | 160 | 9 | 0.05625 | 2 | 8 | 107 | 1 | 44 | 88.9 (84,93.8) | 70.9 (63.9,77.9) |
| D'Souza 2020a^6^ | HM JACKarc analytical system | IBD | 2 | 0 | 1 | 160 | 9 | 0.05625 | 10 | 7 | 127 | 2 | 24 | 77.8 (71.4,84.2) | 84.1 (78.4,89.8) |
| D'Souza 2020a^6^ | HM JACKarc analytical system | IBD | 3 | 0 | 1 | 138 | 3 | 0.021739 | 2 | 1 | 108 | 2 | 27 | 33.3 (25.4,41.2) | 80 (73.3,86.7) |
| D'Souza 2020a^6^ | HM JACKarc analytical system | IBD | 3 | 0 | 1 | 138 | 3 | 0.021739 | 10 | 1 | 122 | 2 | 13 | 33.3 (25.4,41.2) | 90.3 (85.4,95.2) |
| D'Souza 2021c^8^ | HM JACKarc analytical system | IBD | 4 | 0 | 1 | 9822 | 427 | 0.043474 | 2 | 312 | 6050 | 115 | 3345 | 73.1 (72.2,74) | 64.4 (63.5,65.3) |
| D'Souza 2021c^8^ | HM JACKarc analytical system | IBD | 4 | 0 | 1 | 9822 | 427 | 0.043474 | 10 | 247 | 7779 | 180 | 1616 | 57.8 (56.8,58.8) | 82.8 (82.1,83.5) |
| D'Souza 2021c^8^ | HM JACKarc analytical system | IBD | 4 | 0 | 1 | 9822 | 427 | 0.043474 | 150 | 157 | 8803 | 270 | 592 | 36.8 (35.8,37.8) | 93.7 (93.2,94.2) |
| Gerrard 2023^13^ | DUAL FIT HM-JACKarc (either positive) | IBD | 1 | 0 | 1 | 2637 | 33 | 0.012514 | 10 | 30 | 1815 | 3 | 789 | 90.9 (89.8,92) | 69.7 (67.9,71.5) |
| Gerrard 2023^13^ | HM-JACKarc analyser | IBD | 1 | 0 | 1 | 2260 | 59 | 0.026106 | 10 | 45 | 1693 | 14 | 508 | 76.3 (74.5,78.1) | 76.9 (75.2,78.6) |
| Gerrard 2023^13^ | HM-JACKarc analyser | IBD | 1 | 0 | 1 | 3426 | 55 | 0.016054 | 10 | 50 | 2572 | 5 | 799 | 90.9 (89.9,91.9) | 76.3 (74.9,77.7) |
| Juul 2018^32^ | OC-Sensor DIANA | IBD | 4 | 0 | 2 | 3462 | 31 | 0.008954 | 10 | 11 | 2902 | 20 | 529 | 35.5 (33.9,37.1) | 84.6 (83.4,85.8) |
| MacDonald 2022^16^ | HM-JACKarc | IBD | 1 | 0 | 2 | 5250 | 131 | 0.024952 | 10 | 91 | 3378 | 40 | 1741 | 69.5 (68.3,70.7) | 66 (64.7,67.3) |
| Maclean 2021b^40^ | QuikRead go | IBD | 2 | 0 | 2 | 553 | 9 | 0.016275 | 10 | 8 | 378 | 1 | 166 | 88.9 (86.3,91.5) | 69.5 (65.7,73.3) |
| Maclean 2021b^40^ | QuikRead go | IBD | 2 | 0 | 2 | 553 | 9 | 0.016275 | 100 | 4 | 509 | 5 | 35 | 44.4 (40.3,48.5) | 93.6 (91.6,95.6) |
| Maclean 2021b^40^ | QuikRead go | IBD | 2 | 0 | 2 | 553 | 9 | 0.016275 | 150 | 3 | 517 | 6 | 27 | 33.3 (29.4,37.2) | 95 (93.2,96.8) |
| Mowat 2016^36^ | OC-Sensor io | IBD | 4 | 0 | 1 | 750 | 34 | 0.045333 | 4 | 29 | 308 | 5 | 408 | 85.3 (82.8,87.8) | 43 (39.5,46.5) |
| Mowat 2016^36^ | OC-Sensor io | IBD | 4 | 0 | 1 | 750 | 34 | 0.045333 | 10 | 25 | 565 | 9 | 151 | 73.5 (70.3,76.7) | 78.9 (76,81.8) |
| Mowat 2021^17^ & 2019^18^ | HM JACKarc | IBD | 4 | 0 | 1 | 1447 | 68 | 0.046994 | 10 | 64 | 663 | 4 | 716 | 94.1 (92.9,95.3) | 48.1 (45.5,50.7) |

CRC, Colorectal cancer; FN, false negative; FP, False positive; No., number; Pop., population; Prev, prevalence; Pts, patients; Ref Stand, reference standard; TN, true negative; TP, true positive

1. Benton SC, Piggott C, Zahoor Z, O'Driscoll S, Fraser CG, D'Souza N*, et al.* A comparison of the faecal haemoglobin concentrations and diagnostic accuracy in patients suspected with colorectal cancer and serious bowel disease as reported on four different faecal immunochemical test systems. *Clinical Chemistry & Laboratory Medicine* 2022;60:1278-86.

2. MacLean W, Zahoor Z, O'Driscoll S, Piggott C, Whyte MB, Rockall T*, et al.* Comparison of the QuikRead go<sup></sup>point-of-care faecal immunochemical test for haemoglobin with the FOB Gold Wide<sup></sup>laboratory analyser to diagnose colorectal cancer in symptomatic patients. *Clinical Chemistry and Laboratory Medicine* 2022a;60(1):101-8.

3. Schwettmann L, Lied A, Eriksen R. Evaluation of the Sentinel-FOB gold faecal immunochemical test for the presence of haemoglobin using the automated Roche Cobas 8000 system. *Practical Laboratory Medicine* 2022;29:e00263.

4. Chapman CJ, Banerjea A, Humes DJ, Allen J, Oliver S, Ford A*, et al.* Choice of faecal immunochemical test matters: comparison of OC-Sensor and HM-JACKarc, in the assessment of patients at high risk of colorectal cancer. *Clinical Chemistry & Laboratory Medicine* 2021;59:721-8.

5. Cunin L, Khan AA, Ibrahim M, Lango A, Klimovskij M, Harshen R. FIT negative cancers: A right-sided problem? Implications for screening and whether iron deficiency anaemia has a role to play. *The Surgeon* 2021;19:27-32.

6. D'Souza N, Hicks G, Benton SC, Abulafi M. The diagnostic accuracy of the faecal immunochemical test for colorectal cancer in risk-stratified symptomatic patients. *Annals of the Royal College of Surgeons of England* 2020a;102:174-9.

7. D’Souza N, Delisle TG, Chen M, Benton SC, Abulafi M, the NFITSC. Faecal immunochemical testing in symptomatic patients to prioritize investigation: diagnostic accuracy from NICE FIT Study. *British Journal of Surgery* 2021a;108:804-10.

8. D'Souza N, Delisle TG, Chen M, Benton S, Abulafi M. Faecal immunochemical test is superior to symptoms in predicting pathology in patients with suspected colorectal cancer symptoms referred on a 2WW pathway: a diagnostic accuracy study. *Gut* 2021c;70:1130-8.

9. D’Souza N, Monahan K, Benton SC, Wilde L, Abulafi M, Group NFS*, et al.* Finding the needle in the haystack: the diagnostic accuracy of the faecal immunochemical test for colorectal cancer in younger symptomatic patients. *Colorectal Disease* 2021b;23:2539-49.

10. Elbeltagi A, Salama M, Boxall P, Roos J, Lim M. The Yield of Faecal Immunochemical Test in the Detection of Colorectal Cancer within a Fast-track Pathway at York, United Kingdom. *Turkish Journal of Colorectal Disease* 2022;32(3):178-85.

11. Farrugia A, Widlak M, Evans C, Smith SC, Arasaradnam R. Faecal immunochemical testing (FIT) in symptomatic patients: what are we missing? *Frontline Gastroenterol* 2020;11:28-33.

12. Faux JW, Cock K, Bromley R, Feldman M. Colorectal two-week wait service and quantitative FIT: it's not just about colon cancer. *Annals of the Royal College of Surgeons of England* 2022;104:257-60.

13. Gerrard AD, Maeda Y, Miller J, Gunn F, Theodoratou E, Noble C*, et al.* Double faecal immunochemical testing in patients with symptoms suspicious of colorectal cancer. *British Journal of Surgery* 2023;110:471-80.

14. Godber IM, Todd LM, Fraser CG, MacDonald LR, Younes HB. Use of a faecal immunochemical test for haemoglobin can aid in the investigation of patients with lower abdominal symptoms. *Clinical Chemistry & Laboratory Medicine* 2016;54:595-602.

15. Johnstone MS, Burton P, Kourounis G, Winter J, Crighton E, Mansouri D*, et al.* Combining the quantitative faecal immunochemical test and full blood count reliably rules out colorectal cancer in a symptomatic patient referral pathway. *International Journal of Colorectal Disease* 2022a;37:457-66.

16. MacDonald S, MacDonald L, Godwin J, Macdonald A, Thornton M. The diagnostic accuracy of the faecal immunohistochemical test in identifying significant bowel disease in a symptomatic population. *Colorectal Disease* 2022;24:257-63.

17. Mowat C, Digby J, Strachan JA, McCann RK, Carey FA, Fraser CG*, et al.* Faecal haemoglobin concentration thresholds for reassurance and urgent investigation for colorectal cancer based on a faecal immunochemical test in symptomatic patients in primary care. *Annals of Clinical Biochemistry* 2021;58:211-9.

18. Mowat C, Digby J, Strachan JA, McCann R, Hall C, Heather D*, et al.* Impact of introducing a faecal immunochemical test (FIT) for haemoglobin into primary care on the outcome of patients with new bowel symptoms: a prospective cohort study. *BMJ Open Gastroenterology* 2019;6:e000293.

19. Nicholson BD, James T, East JE, Grimshaw D, Paddon M, Justice S*, et al.* Experience of adopting faecal immunochemical testing to meet the NICE colorectal cancer referral criteria for low-risk symptomatic primary care patients in Oxfordshire, UK. *Frontline Gastroenterology* 2019;10:347-55.

20. Nicholson BD, James T, Paddon M, Justice S, Oke JL, East JE*, et al.* Faecal immunochemical testing for adults with symptoms of colorectal cancer attending English primary care: a retrospective cohort study of 14 487 consecutive test requests. *Alimentary Pharmacology & Therapeutics* 2020;52:1031-41.

21. Tang A, Chandler S, Torkington J, Harris DA, Dhruva Rao PK. Adapting the investigation of patients on urgent suspected cancer pathway with lower gastrointestinal symptoms across Wales during COVID-19. *Annals of the Royal College of Surgeons of England* 2022;26:26.

22. Turvill J, Mellen S, Jeffery L, Bevan S, Keding A, Turnock D. Diagnostic accuracy of one or two faecal haemoglobin and calprotectin measurements in patients with suspected colorectal cancer. *Scandinavian Journal of Gastroenterology* 2018;53:1526-34.

23. Turvill J, Turnock D, Cottingham D, al. e. The Fast Track FIT study: diagnostic accuracy of faecal immunochemical test for haemoglobin in patients with suspected colorectal cancer. *Br J Gen Pract* 2021;71:E643–E51.

24. Withrow DR, Shine B, Oke J, Tamm A, James T, Morris E*, et al.* Combining faecal immunochemical testing with blood test results for colorectal cancer risk stratification: a consecutive cohort of 16,604 patients presenting to primary care. *BMC Medicine* 2022;20:116.

25. Archer T, Aziz I, Kurien M, Knott V, Ball A. Prioritisation of lower gastrointestinal endoscopy during the COVID-19 pandemic: outcomes of a novel triage pathway. *Frontline Gastroenterology* 2022;13:225-30.

26. Ayling RM, Lewis SJ, Cotter F. Potential roles of artificial intelligence learning and faecal immunochemical testing for prioritisation of colonoscopy in anaemia. *British Journal of Haematology* 2019;185:311-6.

27. Ball AJ, Aziz I, Parker S, Sargur RB, Aldis J, Kurien M. Fecal Immunochemical Testing in Patients With Low-Risk Symptoms of Colorectal Cancer: A Diagnostic Accuracy Study. *Journal of the National Comprehensive Cancer Network* 2022;20:989-96.e1.

28. Bujanda L, Sarasqueta C, Vega P, Salve M, Quintero E, Alvarez-Sánchez V*, et al.* Effect of aspirin on the diagnostic accuracy of the faecal immunochemical test for colorectal advanced neoplasia. *United European Gastroenterol J* 2018;6:123-30.

29. Cama R, Kapoor N, Sawyer P, Patel B, Landy J. Evaluation of 13,466 Fecal Immunochemical Tests in Patients Attending Primary Care for High- and Low-Risk Gastrointestinal Symptoms of Colorectal Cancer. *Digestive Diseases & Sciences* 2022;10:10.

30. Crooks C, Banerjea A, Jones J, Chapman C, Oliver S, West J*, et al.* Assessing empirical thresholds for investigation in people referred on a symptomatic colorectal cancer pathway: a cohort study utilising faecal immunochemical and blood tests in England. *medRxiv* 2023; 10.1101/2023.03.29.23287919:2023.03.29.23287919.

31. Georgiou Delisle T, D'Souza N, Tan J, Najdawi A, Chen M, Ward H*, et al.* Introduction of an integrated primary care faecal immunochemical test referral pathway for patients with suspected colorectal cancer symptoms. *Colorectal Disease* 2022a;08:08.

32. Juul JS, Hornung N, Andersen B, Laurberg S, Olesen F, Vedsted P. The value of using the faecal immunochemical test in general practice on patients presenting with non-alarm symptoms of colorectal cancer. *British Journal of Cancer* 2018;119(4):471-9.

33. Laszlo HE, Seward E, Ayling RM, Lake J, Malhi A, Stephens C*, et al.* Faecal immunochemical test for patients with 'high-risk' bowel symptoms: a large prospective cohort study and updated literature review. *British Journal of Cancer* 2022;126:736-43.

34. Maclean W, Limb C, Mackenzie P, Whyte MB, Benton SC, Rockall T*, et al.* Adoption of faecal immunochemical testing for 2-week-wait colorectal patients during the COVID-19 pandemic: an observational cohort study reporting a new service at a regional centre. *Colorectal Disease* 2021a;23(7):1622-9.

35. Morales Arraez D, Carrillo G, Adrian M, Gimeno Z, Quintero A. Role of faecal immunochemical testing in the diagnostic workup of patients with iron deficiency anaemia. *United Eur Gastroenterol J* 2018;6:A403–A4.

36. Mowat C, Digby J, Strachan JA, Wilson R, Carey FA, Fraser CG*, et al.* Faecal haemoglobin and faecal calprotectin as indicators of bowel disease in patients presenting to primary care with bowel symptoms. *Gut* 2016;65:1463-9.

37. Pin-Vieito N, Garcia Nimo L, Bujanda L, Roman Alonso B, Gutierrez-Stampa MA, Aguilar-Gama V*, et al.* Optimal diagnostic accuracy of quantitative faecal immunochemical test positivity thresholds for colorectal cancer detection in primary health care: A community-based cohort study. *United European Gastroenterology Journal* 2021;9:256-67.

38. Rodriguez-Alonso L, Rodriguez-Moranta F, Arajol C, Gilabert P, Serra K, Martin A*, et al.* Proton pump inhibitors reduce the accuracy of faecal immunochemical test for detecting advanced colorectal neoplasia in symptomatic patients. *PLoS One* 2018;13:e0203359.

39. Rodriguez-Alonso L, Rodriguez-Moranta F, Ruiz-Cerulla A, Arajol C, Serra K, Gilabert P*, et al.* The use of faecal immunochemical testing in the decision-making process for the endoscopic investigation of iron deficiency anaemia. *Clin Chem Lab Med* 2020;58:232-9.

40. Maclean W, Mackenzie P, Limb C, Zahoor Z, Whyte MB, Rockall T*, et al.* Diagnostic accuracy of point of care faecal immunochemical testing using a portable high-speed quantitative analyser for diagnosis in 2-week wait patients. *Colorectal Disease* 2021b;23:2376-86.

41. Tsapournas G, Hellström PM, Cao Y, Olsson LI. Diagnostic accuracy of a quantitative faecal immunochemical test vs. symptoms suspected for colorectal cancer in patients referred for colonoscopy. *Scandinavian Journal of Gastroenterology* 2020;55:184-92.

42. Sieg A, Thoms C, Lüthgens K, John MR, Schmidt-Gayk H. Detection of colorectal neoplasms by the highly sensitive hemoglobin-haptoglobin complex in feces. *International Journal of Colorectal Disease* 1999;14:267-71.

43. Hunt N, Rao C, Logan R, Chandrabalan V, Oakey J, Ainsworth C*, et al.* A cohort study of duplicate faecal immunochemical testing in patients at risk of colorectal cancer from North-West England. *BMJ Open* 2022;12:e059940.
