# Supplementary material for: Faecal immunochemical tests for patients with symptoms suggestive of colorectal cancer: An updated systematic review and multiple‐threshold meta‐analysis of diagnostic test accuracy studies
Source: Colorectal Dis. 2024 Dec 17;27(1):e17255. doi: 10.1111/codi.17255 (PMC11683176; doi:10.1111/codi.17255)
Supplement: Supplementary file 10 — Data S10. [file CODI-27-0-s003.docx]

#### **Statistical synthesis HM JACKarc**

16 studies contributed to the meta-analysis for HM JACKarc. Seven studies provided diagnostic accuracy at a single threshold and the maximum number of thresholds considered within a single study was 103. The final dataset provided a total of 151 pairs of sensitivity and specificity, at thresholds between 2 and 401 µg/ml.

Figure 1 A displays the results on the ROC plane. Observations from the same study are joined. Figure 1 B displays the sensitivity and specificity as a function of threshold. Pooled sensitivity and specificity are shown for subgroups based on population type in Figure 5 C and Figure 5 D, respectively. Sensitivity and specificity for specific thresholds is summarised for all population groups in Table 1.

For the analysis of all studies (populations 1-4), sensitivity ranges from 95.9 (95% CrI: 92.7, 97.9; 95% PrI: 81.4, 99.8) at a threshold of 2, to 46.3 (95% CrI: 37.4, 54.9; 95% PrI: 21.9, 70.2) at a threshold of 400. Specificity ranges from 65.1 (95% CrI: 55.6, 74.8; 95% PrI: 30.3,96.7) at a threshold of 2, to 97.7 (95% CrI: 94.7, 99.2; 95% PrI: 78.1,100). For the analyses of subgroups by population type, the summary estimates were similar and not statistically significant based on overlap of the 95% CrI. The summary sensitivity and specificity for population 3 are higher than for the other considered subgroups, however this analysis was based on only two studies that contributed data at two thresholds (2 and 10). There is therefore considerable uncertainty in the pooled estimates and these should be interpreted with caution.

**Figure 1: Observed data and summary sensitivity and specificity for HM JACKarc**


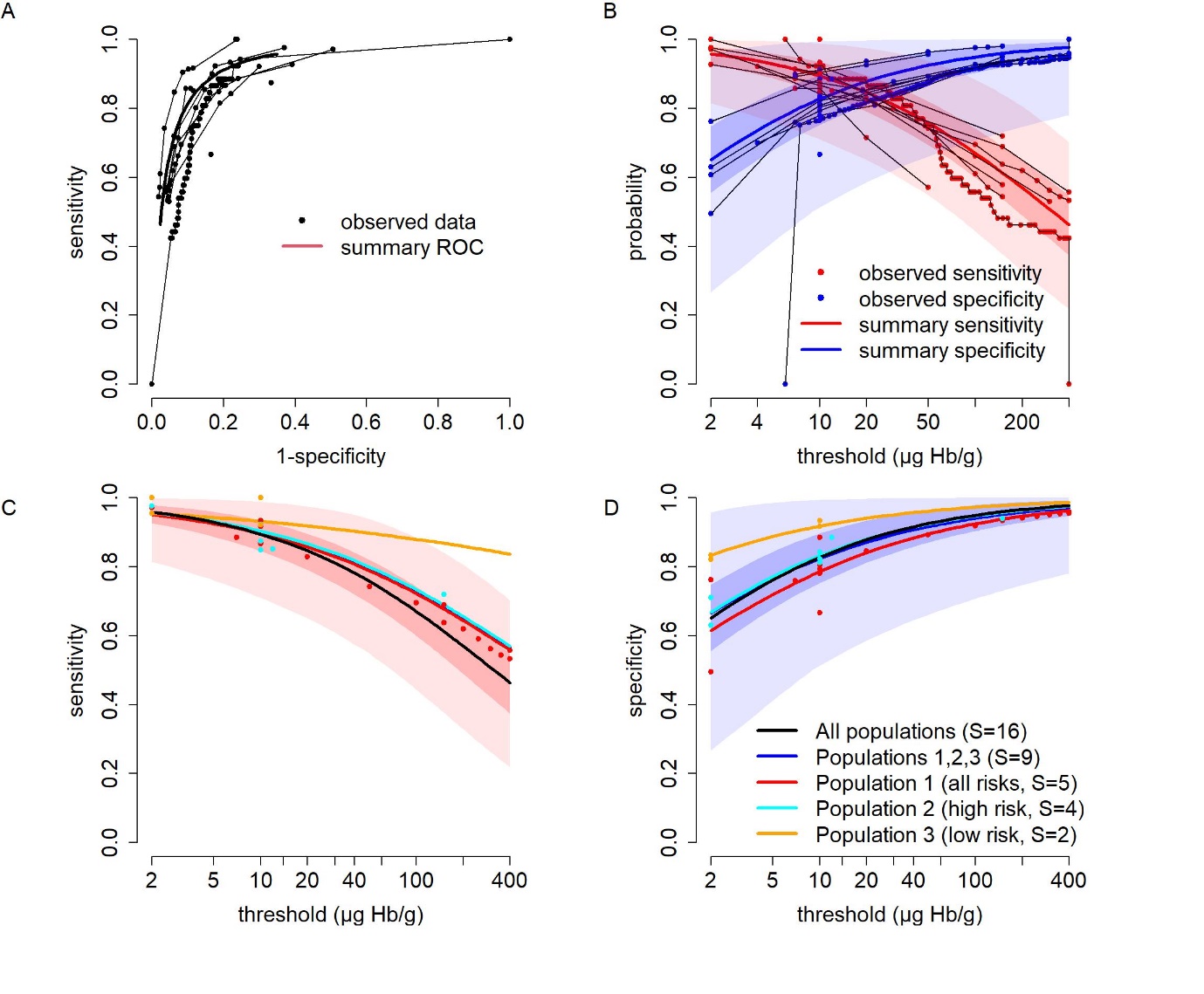


95% credible intervals and predictive intervals for summary sensitivity are shown by the dark and light red regions. 95% credible and predictive intervals for summary specificity are shown by the dark and light blue regions.

**Table 1: Summary sensitivity and specificity at specific thresholds for HM-JACKarc**

| **Threshold** | **All studies 1-4 (n=16)** | | **All 1-3 (n=9)** | | **Population 1 (S=5)** | | **Population 2 (S=4)** | | **Population 3 (S=2)** | |
| --- | --- | --- | --- | --- | --- | --- | --- | --- | --- | --- |
|  | **sensitivity** | **specificity** | **sensitivity** | **specificity** | **sensitivity** | **specificity** | **sensitivity** | **specificity** | **sensitivity** | **specificity** |
| **2** | 95.9 (92.7,97.9) | 65.1 (55.6,74.8) | 95.5 (93,97.1) | 66.7 (54.9,77.2) | 95.2 (89.3,98.5) | 61.6 (41.3,81.8) | 95.7 (89.1,98.2) | 66.8 (60.4,75.3) | 95.5 (83.4,100) | 83.3 (74.1,91.2) |
| **2.5** | 95.3 (91.8,97.5) | 68 (58.8,77.3) | 95 (92.4,96.7) | 69.3 (57.8,79.3) | 94.6 (88.4,98.2) | 64.2 (44.4,83.9) | 95.2 (88.3,97.9) | 69.5 (63.2,77.6) | 95.3 (83,99.9) | 84.8 (75.8,92.3) |
| **3** | 94.7 (91.1,97.2) | 70.3 (61.3,79.3) | 94.5 (91.8,96.3) | 71.3 (60.2,80.9) | 94.1 (87.6,98) | 66.3 (46.9,85.4) | 94.7 (87.6,97.7) | 71.7 (65.4,79.3) | 95 (82.6,99.9) | 85.9 (77.1,93.1) |
| **4** | 93.8 (89.8,96.5) | 73.7 (65.1,82.2) | 93.6 (90.7,95.7) | 74.3 (63.7,83.3) | 93.3 (86.3,97.6) | 69.5 (50.6,87.8) | 93.9 (86.4,97.2) | 74.8 (68.7,81.9) | 94.6 (82,99.9) | 87.6 (79.2,94.3) |
| **7** | 91.4 (86.8,94.8) | 79.6 (71.7,87.1) | 91.6 (88.3,94.1) | 79.5 (70.1,87.2) | 91.2 (83.3,96.6) | 75.3 (57.1,91.4) | 91.9 (83.7,95.9) | 80.3 (74.5,86.2) | 93.8 (80.6,99.9) | 90.3 (82.6,96) |
| **10** | 89.5 (84.6,93.4) | 82.8 (75.2,89.6) | 90.1 (86.5,92.8) | 82.4 (73.7,89.3) | 89.6 (81.1,95.7) | 78.6 (60.7,93.2) | 90.4 (81.7,94.9) | 83.3 (77.8,88.5) | 93.2 (79.6,99.8) | 91.8 (84.6,96.8) |
| **20** | 84.7 (79.1,89.6) | 87.9 (81.1,93.4) | 86.3 (82.1,89.7) | 87.1 (79.8,92.6) | 85.7 (76.2,93.6) | 84.1 (67.1,95.8) | 86.7 (77.1,92.1) | 88 (83.2,92.2) | NR | NR |
| **50** | 75.8 (69.4,82) | 92.6 (87,96.5) | 79.5 (74.5,83.8) | 91.7 (86,95.5) | 78.8 (68,89.2) | 89.5 (74,97.9) | 79.9 (69.1,86.7) | 92.4 (88.7,95.4) | NR | NR |
| **100** | 67 (60,74.2) | 94.9 (90.3,97.8) | 73 (67.1,78.1) | 94.1 (89.5,97) | 72.2 (60.4,84.7) | 92.5 (78.1,98.8) | 73.4 (61.3,81.1) | 94.7 (91.6,97) | NR | NR |
| **120** | 64.5 (57.2,71.9) | 95.4 (91,98.1) | 71 (64.9,76.4) | 94.6 (90.3,97.3) | 70.2 (58.3,83.3) | 93.1 (79.2,98.9) | 71.4 (58.9,79.4) | 95.2 (92.3,97.4) | NR | NR |
| **150** | 61.3 (53.7,68.9) | 96 (91.9,98.4) | 68.5 (62.1,74.2) | 95.2 (91.1,97.6) | 67.8 (55.5,81.5) | 93.8 (80.4,99.1) | 68.9 (55.8,77.3) | 95.7 (93,97.7) | NR | NR |
| **200** | 57 (48.9,64.9) | 96.6 (92.8,98.7) | 65.2 (58.4,71.2) | 95.8 (92.1,98) | 64.4 (51.7,79.1) | 94.6 (81.7,99.3) | NR | NR | NR | NR |
| **400** | 46.3 (37.4,54.9) | 97.7 (94.7,99.2) | 56.5 (48.7,63.5) | 97.1 (94.1,98.7) | 55.8 (41.8,72.6) | 96.2 (84.8,99.6) | NR | NR | NR | NR |
