# Supplementary material for: Faecal immunochemical tests for patients with symptoms suggestive of colorectal cancer: An updated systematic review and multiple‐threshold meta‐analysis of diagnostic test accuracy studies
Source: Colorectal Dis. 2024 Dec 17;27(1):e17255. doi: 10.1111/codi.17255 (PMC11683176; doi:10.1111/codi.17255)
Supplement: Supplementary file 11 — Data S11. [file CODI-27-0-s005.docx]

#### **Statistical synthesis OC-Sensor**

Eleven studies contributed to the meta-analysis for OC-Sensor. One study provided diagnostic accuracy at a single threshold and the maximum number of thresholds considered within an individual study was 10 (Laszlo 2021).^1^ The final dataset included a total of 44 pairs of sensitivity and specificity estimates, at thresholds between 4 and 200 µg/ml.

Figure 1 A displays the results on the ROC plane. Figure 6 B displays the sensitivity and specificity as a function of threshold. Pooled sensitivity and specificity are shown for subgroups based on population type in Figure 1 C and Figure 6 D, respectively. Sensitivity and specificity for specific thresholds is summarised for all population groups in Table 1.

For the analysis of all studies (populations 1-4), sensitivity ranges from 94.2 (95% CrI: 91.2, 96.7; 95% PrI: 84.6, 99.0) at a threshold of 4, to 54.2 (95% CrI: 48.4, 60.2; 95% PrI: 42.2, 67.2) at a threshold of 200. Specificity ranges from 62.7 (95% CrI: 47.4, 77.2; 95% PrI: 12.0,97.7) at a threshold of 4, to 97.3 (95% CrI: 92.9, 99.3; 95% PrI: 71.9,100) at a threshold of 200. For the analyses of subgroups by population type, the summary estimates were similar and not statistically significant based on overlap of the 95% CrI.

**Figure 1: Observed data and summary sensitivity and specificity for OC-Sensor**


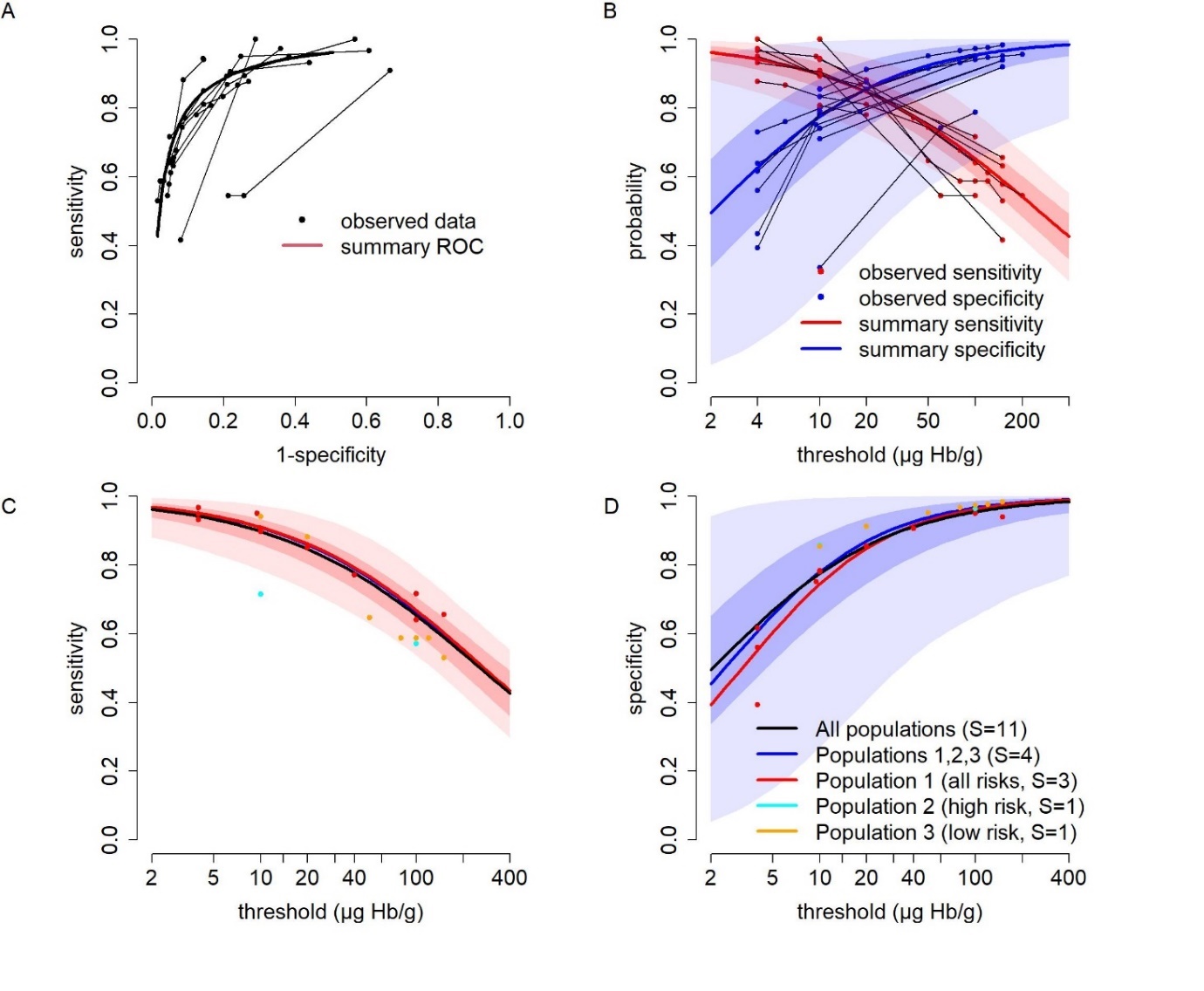


**Table 1: Summary sensitivity and specificity at specific thresholds for OC-Sensor**

| **threshold** | **All studies 1-4 (S=11)** | | **All 1-3 (S=4)** | | **Population 1 (S=3)** | |
| --- | --- | --- | --- | --- | --- | --- |
|  | **sensitivity** | **specificity** | **sensitivity** | **specificity** | **sensitivity** | **specificity** |
| **4** | 94.2 (91.2,96.7) | 62.7 (47.4,77.2) | 95 (91.8,97.5) | 60.8 (42,77.9) | 95.1 (91.1,97.9) | 55.3 (36.3,73.5) |
| **7** | 91.8 (88.2,94.9) | 72.3 (58.1,84.8) | 92.8 (88.9,96) | 72 (54.6,86) | 92.9 (88.2,96.7) | 67.6 (49.5,83.5) |
| **10** | 89.8 (85.9,93.3) | 77.6 (64.3,88.6) | 90.9 (86.6,94.7) | 78.1 (62.2,89.8) | 91 (85.9,95.5) | 74.5 (57.6,88.6) |
| **20** | 84.7 (80.3,89) | 85.6 (74.5,93.6) | 86 (80.9,90.9) | 87 (74.5,94.7) | 86.2 (80.2,92.3) | 84.8 (70.5,94.6) |
| **50** | 75 (70.2,80) | 92.5 (84.3,97.3) | 76.3 (70.4,82.8) | 93.9 (85.8,98) | 76.6 (70,84.7) | 93 (82.4,98.2) |
| **100** | 65.3 (60.2,70.7) | 95.5 (89.4,98.6) | 66.3 (60.2,73.9) | 96.6 (91.2,99) | 66.8 (60.1,76.2) | 96.2 (88.5,99.3) |
| **120** | 62.5 (57.2,68) | 96.1 (90.4,98.9) | 63.4 (57.1,71.3) | 97.1 (92.3,99.2) | 64 (57.2,73.6) | 96.8 (89.7,99.4) |
| **150** | 58.9 (53.4,64.7) | 96.7 (91.6,99.1) | 59.7 (53.3,67.8) | 97.7 (93.4,99.4) | 60.3 (53.4,70.2) | 97.4 (91.1,99.6) |
| **200** | 54.2 (48.4,60.2) | 97.3 (92.9,99.3) | NR | NR | NR | NR |

1. Laszlo HE, Seward E, Ayling RM, et al. Faecal immunochemical test for patients with 'high-risk' bowel symptoms: a large prospective cohort study and updated literature review. *British Journal of Cancer* 2022;126(5):736-43.
