# Supplementary material for: Faecal immunochemical tests for patients with symptoms suggestive of colorectal cancer: An updated systematic review and multiple‐threshold meta‐analysis of diagnostic test accuracy studies
Source: Colorectal Dis. 2024 Dec 17;27(1):e17255. doi: 10.1111/codi.17255 (PMC11683176; doi:10.1111/codi.17255)
Supplement: Supplementary file 12 — Data S12. [file CODI-27-0-s009.docx]

#### **Statistical synthesis FOB Gold**

Three studies contributed to the meta-analysis for FOB Gold (Benton 2022,^46^ Maclean 2022a,^62^ Jordaan 2023).^86^ The number of thresholds considered by each study ranged from 1 to 4 and the final dataset provided a total of 8 pairs of sensitivity and specificity, at thresholds between 2 and 150.

Figure 7 A displays the results on the ROC plane. Observations from the same study are joined by a line. Figure 7 B displays the sensitivity and specificity as a function of threshold. Due to the small number of studies evaluating FOB Gold subgroup analyses by population type were not conducted. Sensitivity and specificity for specific thresholds is summarised for all population groups in Table 12.

The summary sensitivity and specificity are plotted in Figure 8, with information relating to the number of participants and number of positive tests in each study. For the analysis of all studies (populations 1-4), sensitivity ranged from 91.4 (95% CrI: 71.6, 99.6; 95% PrI: 62.3, 100.0) at a threshold of 2, to 73.9 (95% CrI: 53.8, 91.2; 95% PrI: 51.6, 98.0) at a threshold of 150. Specificity ranged from 78.1 (95% CrI: 70.0, 86.0; 95% PrI: 62.8,91.8) at a threshold of 2, to 96.4 (95% CrI: 92.6, 98.9; 95% PrI: 87.1,99.7) at a threshold of 150.

**Figure 7:** **Observed data and summary sensitivity and specificity for FOB Gold Primary analysis**


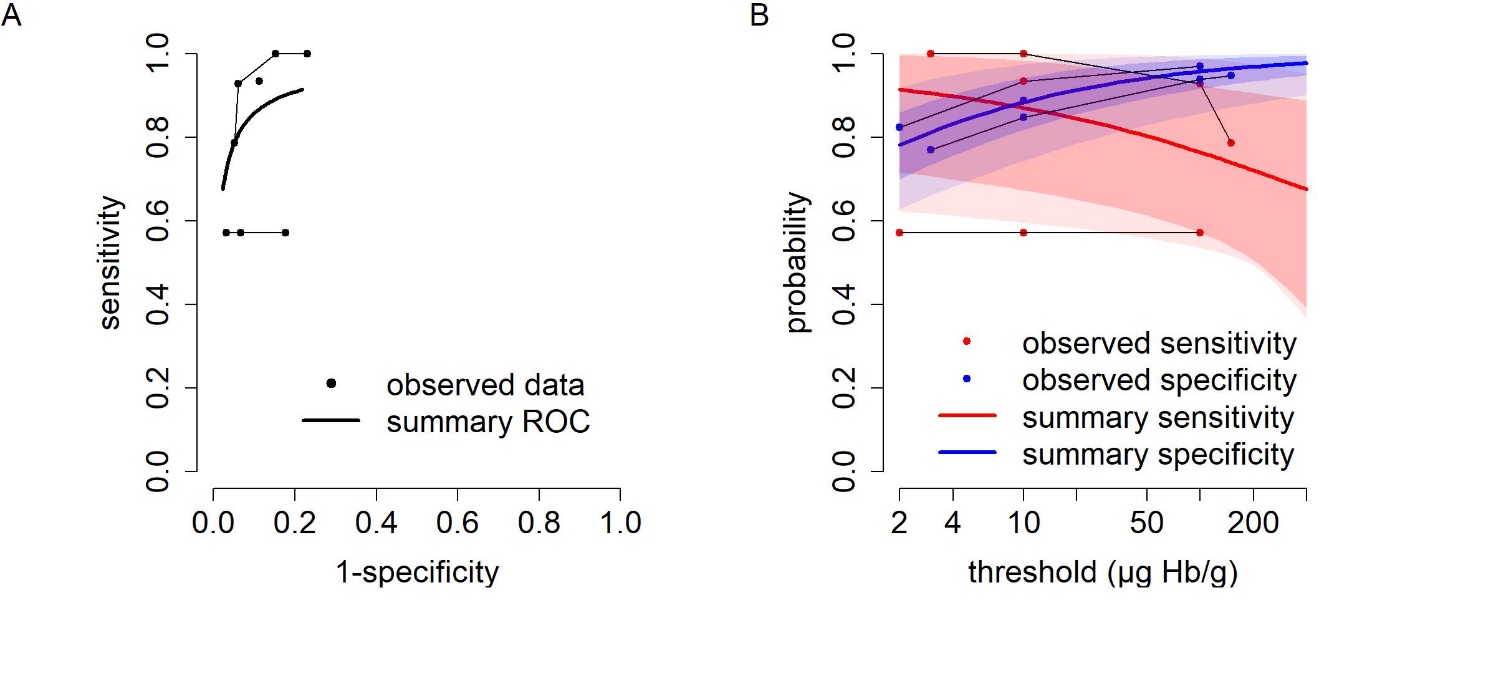


95% credible intervals and predictive intervals for summary sensitivity are shown by the dark and light red regions. 95% credible and predictive intervals for summary specificity are shown by the dark and light blue regions

**Figure 8: Observed data and summary sensitivity and specificity for FOB Gold Primary analysis, with study information.**


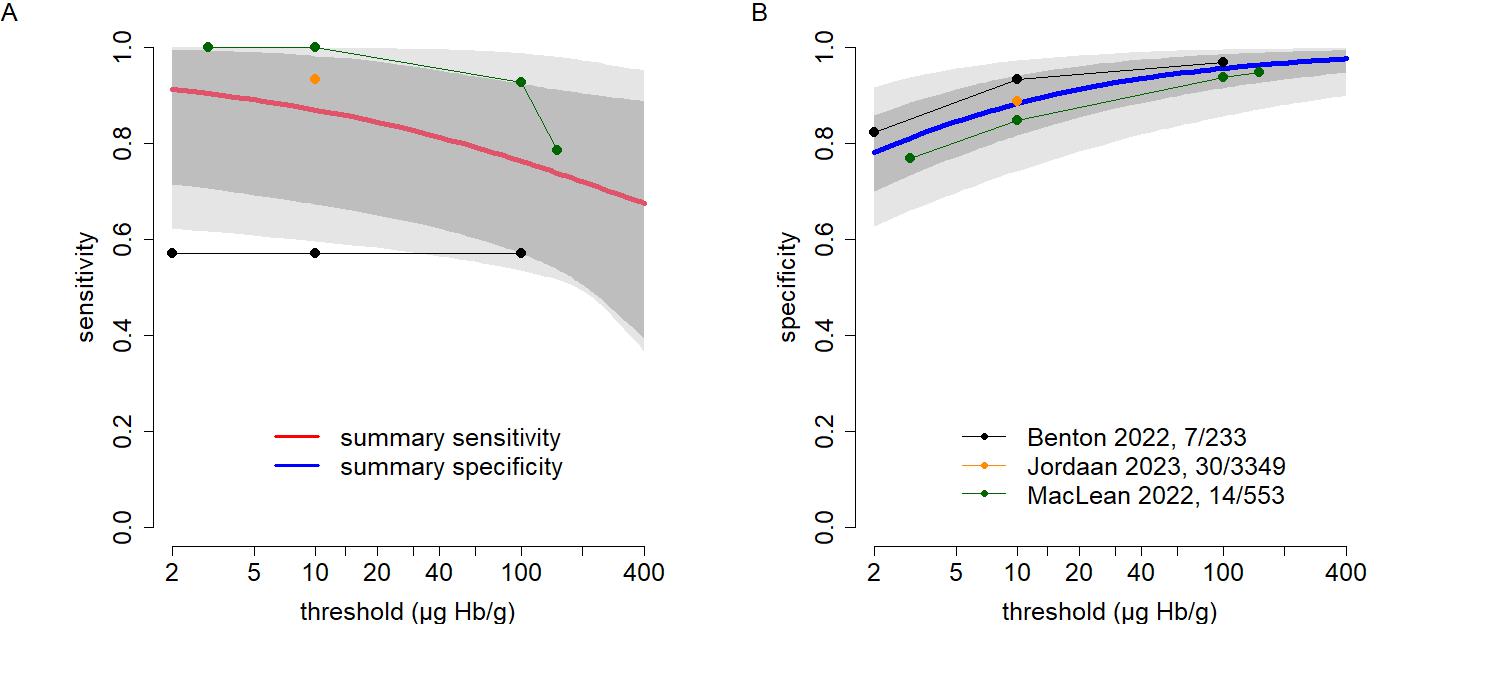


95% credible intervals and predictive intervals for summary sensitivity and specificity are shown by the dark and light grey regions.
