# Supplementary material for: Faecal immunochemical tests for patients with symptoms suggestive of colorectal cancer: An updated systematic review and multiple‐threshold meta‐analysis of diagnostic test accuracy studies
Source: Colorectal Dis. 2024 Dec 17;27(1):e17255. doi: 10.1111/codi.17255 (PMC11683176; doi:10.1111/codi.17255)
Supplement: Supplementary file 13 — Data S13. [file CODI-27-0-s017.docx]

***All tests together – investigating impact of population type:*** This analysis included studies of all tests (n=28) together and was conducted to further test whether population type affects test accuracy on a larger sample. Again, the summary estimates were similar and not statistically significant based on overlap of the 95% CrIs (see online supplement 12). Whilst the summary specificity for population 3 was numerically higher than for the other considered subgroups, the analysis was based on only three studies (2 HM-JACKarc studies and 1 OC-Sensor), and should be interpreted with caution.

**Figure 1: Observed data and summary sensitivity and specificity for all tests together**


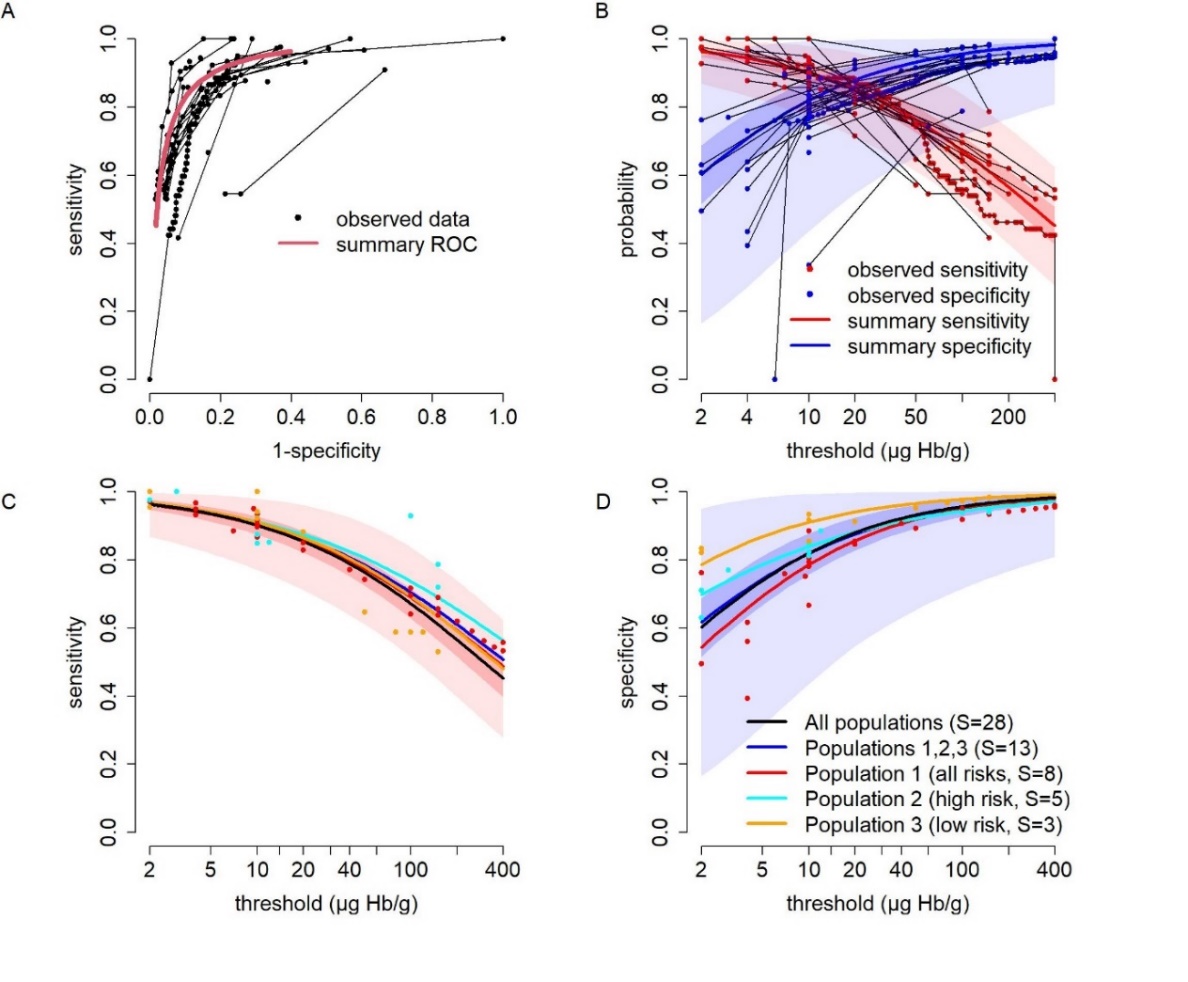


**C**

**A**

**Table 1: Summary sensitivity and specificity at specific thresholds for all tests together**

| **Threshold, µg/g** | **All studies 1-4 (n=28)** | | **All 1-3 (n=13)** | | **Population 1 (S=8)** | | **Population 2 (S=5)** | | **Population 3 (S=3)** | |
| --- | --- | --- | --- | --- | --- | --- | --- | --- | --- | --- |
|  | **sensitivity** | **specificity** | **sensitivity** | **specificity** | **sensitivity** | **specificity** | **sensitivity** | **specificity** | **sensitivity** | **specificity** |
| **2** | 96.4 (94.7,97.7) | 60.3 (51.6,68.8) | 96.3 (94.6,97.5) | 61.8 (49.2,73.6) | 96.2 (93.5,97.9) | 54.4 (37.6,71) | 96.3 (83.1,99.9) | 69.8 (58.9,81.7) | 96.7 (89.9,99.6) | 78.6 (59.5,93.1) |
| **2.5** | 95.8 (94,97.2) | 63.8 (55.5,72.1) | 95.8 (93.9,97.1) | 65.1 (52.9,76.4) | 95.6 (92.8,97.5) | 58.2 (41.6,74.4) | 95.8 (82.1,99.9) | 72.1 (61.3,83.4) | 96.2 (89.1,99.5) | 80.9 (62.4,94.3) |
| **3** | 95.3 (93.4,96.9) | 66.7 (58.6,74.7) | 95.3 (93.3,96.7) | 67.7 (55.8,78.6) | 95.1 (92.1,97.2) | 61.3 (44.9,77.1) | 95.4 (81.1,99.8) | 74 (63.3,84.8) | 95.7 (88.4,99.4) | 82.6 (64.7,95.1) |
| **4** | 94.4 (92.3,96.2) | 70.9 (63.2,78.4) | 94.4 (92.3,96.1) | 71.6 (60.4,81.8) | 94.2 (91,96.6) | 65.9 (50.1,80.8) | 94.6 (79.6,99.7) | 76.7 (66.4,86.7) | 94.9 (87.1,99.2) | 85.1 (68.1,96.1) |
| **7** | 92.1 (89.6,94.3) | 78.1 (71.3,84.6) | 92.4 (89.8,94.4) | 78.3 (68.3,87) | 92 (88.2,95) | 74.1 (59.6,86.9) | 92.7 (76.4,99.5) | 81.3 (71.7,89.8) | 92.8 (84,98.5) | 89.1 (73.8,97.7) |
| **10** | 90.2 (87.4,92.7) | 82 (75.7,87.8) | 90.7 (87.8,93) | 81.9 (72.7,89.7) | 90.3 (86.1,93.7) | 78.6 (65,89.9) | 91.2 (73.9,99.2) | 83.9 (74.8,91.5) | 91 (81.5,97.8) | 91.1 (76.9,98.3) |
| **20** | 85.4 (82.2,88.5) | 88 (82.7,92.5) | 86.4 (83.1,89.4) | 87.6 (79.9,93.6) | 85.8 (81,90.3) | 85.7 (73.7,94.2) | 87.5 (68.6,98.3) | 88.1 (79.9,94.1) | 86.6 (75.5,95.7) | 94.1 (81.9,99.1) |
| **50** | 76.3 (72.5,80.1) | 93.2 (89.3,96.2) | 78.5 (74.3,82.4) | 92.7 (86.8,96.7) | 77.4 (72,83.4) | 91.9 (82.4,97.4) | 80.5 (59.3,95.8) | 92.2 (85.2,96.5) | 78 (62.5,90.8) | 96.6 (87.1,99.6) |
| **100** | 67.2 (62.8,71.5) | 95.7 (92.7,97.7) | 70.5 (65.7,75.3) | 95.2 (90.6,98.1) | 69 (63.2,76.3) | 94.9 (87.2,98.6) | 73.7 (50.1,92.2) | 94.4 (88.4,97.7) | 69.3 (48.4,85.7) | 97.8 (90,99.8) |
| **120** | 64.5 (60,68.9) | 96.2 (93.4,98) | 68.1 (63.2,73.2) | 95.7 (91.4,98.3) | 66.5 (60.6,74.1) | 95.5 (88.3,98.8) | 71.6 (47.4,90.9) | 94.8 (89.1,97.9) | 66.7 (44.3,84.2) | 98 (90.7,99.8) |
| **150** | 61.1 (56.4,65.7) | 96.7 (94.1,98.4) | 65.1 (59.9,70.5) | 96.3 (92.3,98.6) | 63.4 (57.3,71.4) | 96.1 (89.4,99) | 69 (43.4,89.2) | 95.4 (89.9,98.1) | 63.4 (39.1,82.4) | 98.3 (91.5,99.9) |
| **200** | 56.5  (51.6,61.4) | 97.3  (95,98.7) | 61 (55.5,66.8) | 96.9 (93.3,98.9) | 59.2 (52.8,67.6) | 96.8 (90.7,99.3) | NR | NR | NR | 98.6 (92.3,99.9) |
| **400** | 45.2  (39.7,50.6) | 98.3 (96.6,99.2) | 50.7 (44.6,57.2) | 98 (95.3,99.3) | 48.7 (41.7,57.9) | 98 (93.4,99.6) | NR | NR | NR | 99.1 (94.2,100) |
