# Supplementary material for: Faecal immunochemical tests for patients with symptoms suggestive of colorectal cancer: An updated systematic review and multiple‐threshold meta‐analysis of diagnostic test accuracy studies
Source: Colorectal Dis. 2024 Dec 17;27(1):e17255. doi: 10.1111/codi.17255 (PMC11683176; doi:10.1111/codi.17255)
Supplement: Supplementary file 14 — Data S14. [file CODI-27-0-s007.docx]

**Studies that were only included in the subgroup or other outcomes analyses**

This supplement contains:

1. Table of studies that were only included in the subgroup analyses relating to patient characteristics of anaemia, age, sex, medications that might cause GI bleeding, ethnicity, and people with blood disorders (other than anaemia) that might affect FIT. They were not included in the main analyses for the tests as the populations were too narrow. For each subgroup analysis, subgroup data from studies included in the main analyses were combined with data from these studies to form the relevant subgroups. The study and patient characteristics of the studies already included in the main analyses have already been presented in online supplement 7.
2. The narrative summaries and data for each subgroup analysis (anaemia, age, sex, medications that might cause GI bleeding, ethnicity, people with blood disorders)

# 1) Table of studies

**Table 1 Study and patient characteristics of studies that were only included in subgroup analyses.**

| **^d^** | **Author, year**  **Location**  **Recruitment dates**  **Study name (if available)** | **Analyser**  **Reference standard** | **Inclusion criteria** | **Comparison to NICE scope** | **Mean/median age in years** | **Patient characteristics**   - Male; - Ethnicity;   Anaemia status | **N with CRC/ N analysed (%)** | **Thresholds µg/g** | **Subgroup data?** |
| --- | --- | --- | --- | --- | --- | --- | --- | --- | --- |
| **HM-JACKarc** | | | | | | | | | |
| 29 | Cunin 2020^1a^  East Sussex, UK  NR [must be between 2013 and 2019] | HM JACKarc  Various imaging^a^ | **Type 2:** NG12 high/medium-risk patients with/without IDA | NR | With IDA: median 74 (IQR 65 to 82)  Without IDA:  median 72 (IQR 63-79) | With IDA:   - 37% - NR - 100%   Without IDA:   - 41.3% - NR   0% | With IDA:  20/189 (10.6%)  Without IDA: 28/739 (3.79%) | 10 | IDA, no IDA |
| 6 | D'Souza 2021b^2^  NICE FIT  October 2017 to December 2019 | HM JACKarc analytical system  Colonoscopy | **Type 4:** NG12 High/medium-risk (subgroup of main NICE FIT study) who underwent colonoscopy | NR | Age <50:  42 (SD6.5)  Age 50+:  66.7 (SD9.16) | Age <50:   - 40.3% - Ethnicity reported^a^ - IDA 5.9%, non-IDA anaemia 1.9%   Age 50+   - 45.7% - Ethnicity reported^a^ - IDA 4.8%, non-IDA anaemia 5.5% | Age <50:  16/1103 (1.45%)  Age 50+:  313/8719 (3.59%) | 2, 10, 150 | Age </>50 |
| **OC-Sensor** | | | | | | | | | |
| **30** | **Ayling 2019^3^**  Derriford Hospital, Plymouth, UK  March 2014 to March 2017 | - NR   endoscopy or computed tomography scan (NR what type of CT) | Population type 4 - 2WW patients | NR | NR | - NR - For n=428, 99.8% White British - 100% anaemia or IDA | Low Haemoglobin group: 7/178 (3.93%)  IDA group: 6/137 (4.38%) | 10 | IDA; Anaemia |
| **31** | **Bujanda 2018^4^**  Spain (assume Ourense and San Sebastian)^5^  March 2012 to 2014  COLONPREDICT | - NR   colonoscopy | Population type 4 - Symptomatic patients referred from primary and secondary care | NR | Aspirin users: mean 72.5 (SD 9)  Aspirin non-users: mean 63.7(SD 14) | - Aspirin users: 58.10%; Aspirin non-users: 48.5% - NR   NR | Aspirin users: 51/485 (10.51%)  Aspirin non-users: 299/2567 (11.65%) | 20 | Aspirin users; Aspirin non-users |
| **32** | **Morales-Arraez 2018^6^**  La Laguna, Spain  April 2016 to Dec 2017 | - NR   colonoscopy | Population type 4 - Anaemic patients^d^ referred from primary care | NR | Mean 71 (SD 12) | - 33.90% - NR   100% IDA | 28/245 (11.43%) | 10 | IDA |
| **33** | **Rodriguez-Alonso 2018^7^**  Barcelona, Spain  Sept 2011 to Oct 2012 | - MICRO   Colonoscopy | Population type 4 - Symptomatic patients referred from primary and secondary care | NR | PPI users: mean (SE) 64.9 ± 11.3  PPI non-users: mean (SE) 57.3 ± 14.0 | - PPI users, 44.20%; non-users, 49.80% - NR   users 18.6%; non-users 5.4% | PPI users: 15/525 (2.86%)  PPI non-users: 15/477 (3.14%) | 20 | PPI use |
| **33** | **Rodriguez-Alonso 2020^8^**  Barcelona, Spain  Sept 2011 to Oct 2012 | - MICRO   Colonoscopy | Population type 4 - Symptomatic patients referred from primary and secondary care | NR | NR | - 48.3 - NR   100% IDA | 9/120 (7.5%) | 15 | IDA |

2WW, two week wait; CT, computed tomography; FIT, faecal immunochemical test; ID, study identification number; IDA, iron-deficiency anaemia; IQR, interquartile range; NG12, NICE national guideline 12; NR, not reported; SD, standard deviation

**^a^Cunin 2021:** excluded from the main analysis as recruitment dates not reported meaning crossover could not be ascertained, but used in the anaemia subgroup analysis. reference standard was colonoscopy, oesophago-gastroduodenoscopy (OGD), computed tomography (CT) scanning or virtual CT colonography; **D’Souza 2021b: age <50,** White 68.4%, Asian 9.8%, Black 5.4%, Mixed 1.1%, Chinese 1.2%, Other  18.1%, missing 4.4%

# 2) Subgroup analyses and other outcomes

### Subgroup analyses by patient characteristics

Exploration of the potential reasons for heterogeneity in diagnostic test accuracy across studies using meta-regression was considered. However, study level covariates relating to patient characteristics of interest were not reported in all studies. Instead, studies which reported diagnostic test accuracy for subgroups of patients were considered in subgroup analyses.

#### *1.1 Anaemia*

Studies reporting data on anaemia are summarised in Table 1. Considering all the available data on anaemia regardless of the test used, population type and reference standard, eleven studies^1 3 6 8-15^ reported data on anaemia or IDA. The studies can be broadly categorised as comparative, comparing those with anaemia to those without; comparative, comparing those with anaemia to the study population unselected on the basis of anaemia (whole cohort); or non-comparative.

When considering studies that compare those with anaemia to those without, both^1 11^ reported lower sensitivity and specificity at a threshold of 10 for those with anaemia. One further study^14^ reported that the optimal threshold (defined as the point on the ROC curve that maximises sensitivity and specificity) for those with anaemia is higher than those without. It should be noted that the definition of “optimal” in this study is not necessarily the same as optimising the threshold for cost-effectiveness or clinical decision making, where it may be preferable to optimise either sensitivity (where the test is used to rule out disease) or specificity (where the tests is used to rule in disease).

Amongst studies that compared those with anaemia to the whole study population, the results were more mixed. One study^10^ showed the same trend of lower sensitivity and specificity, three^9 13 15^ showed higher sensitivity and lower specificity, and one^12^ showed lower sensitivity and higher specificity.

Of particular note is a study by Withrow *et al*. 2022^15^, which shows that sensitivity increases as the threshold for anaemia is increased (i.e., more anaemic), whilst the specificity decreases in both men and women.

It should be noted that the definition of anaemia varied across studies, and some considered IDA anaemia whilst other considered other types of anaemia as well or instead.

**Table 1: Sensitivity and specificity of studies reporting data for patients with anaemia**

| **Author, year**  **Test** | **Population type** | **Anaemia type** | **Threshold, µg/g** | **N with CRC/N analysed** | **Sensitivity (95% CI)** | **Specificity (95% CI)** | **Summary of IDA/anaemia vs comparator** |
| --- | --- | --- | --- | --- | --- | --- | --- |
| **Studies comparing those with to those without anaemia/IDA** | | | | | | | |
| Cunin 2020^1a^  HM-JACKarc | 2 | **Whole cohort** | 10 | 48/928 (5.2%) | 85.4  (NR) | 86.9  (NR) | **Vs “no IDA”** Sens & Spec lower |
|  |  | **No IDA** | 10 | 28/739 | 89.00  (70-97.1) | 84.00  (81.1-86.6) |  |
|  |  | IDA | 10 | 20/189 | 80.00  (55.7-93.3) | 81.60  (74.8-87) |  |
| Johnstone 2022a^11^  HM-JACKarc | **1** | **No anaemia** | 10 | 32/3238 (0.99%) | 96.9 (96.3,97.5)^a^ | 81.3 (80,82.6) ^a^ | Sens & spec lower |
|  |  | Anaemia | 10 | 26/793 (3.28%) | 84.6 (82.1,87.1) ^a^ | 72.9 (69.8,76) ^a^ |  |
| Turvill 2021^14^  HM-JACKarc | **4** | **No IDA** | 19 | 101/3582 (2.8%) | 88.1 (80.2-93.7) | 85.3  (84.0-86.4) | Optimal threshold higher  Optimal FIT threshold was ≥21 vs ≥19µg/g in anaemic vs non-anaemic |
|  |  | IDA | 21 | 34/559 (6.1%) | 82.40  (65.5-93.2) | 81.50  (77.9-84.8) |  |
| Turvill 2021^14^  HM-JACKarc | **4** | **No non-ID anaemia** | 19 | 110/3597 (3.1%) | 84.5  (76.4-90.7) | 85.0  (83.7-86.1) | Optimal threshold higher  Optimal FIT threshold was ≥30 vs ≥19µg/g in anaemic vs non-anaemic |
|  |  | Non-ID Anaemia | 30 | 25/544 (4.6%) | 92.00  (74.0-99.0) | 85.50  (82.2-88.5) |  |
| **Studies comparing those with anaemia/IDA to patients unselected on the basis of anaemia (whole cohort)** | | | | | | | |
| D'Souza 2021a^9^  HM-JACKarc | **4** | **Whole cohort** | 10 | 12/298 (4.03%) | 92.20% (88.2, 95.2) | 82.30% (81.3, 83.2) | Sens higher, spec similar |
|  |  | **IDA** | 10 | 16/479 (3.34%) | 100% (89.4, 100) | 81.60% (77.7, 85.1) |  |
| Tang 2022^13^  HM-JACKarc | **4** | **Whole cohort** | 10 | 20/603 (3.32%) | 90.00 (68.3–98.77) | 83.20 (79.9-86.14) | Sens higher, spec lower (low events in IDA) |
|  |  | IDA | 10 | 1/78 (1.28%) | 100 (NE,NE) ^a^ | 76.6 (67.2,86) ^a^ |  |
| Juul 2018^12^  OC-Sensor | **4** | **Whole cohort** | 10 | 54/3462 (1.56%) | 94.4 (93.6,95.2) ^a^ | 85.7 (84.5,86.9) ^a^ | Sens lower, spec higher/similar |
|  |  | Unexplained anaemia | 10 | 54/3462 (1.56%) | 20.4 (16.6,24.2) ^a^ | 79.5 (75.7,83.3) ^a^ |  |
| Gerrard 2023^10^  Single FIT, HM-JACKarc | **1** | **Whole cohort** | 10 | 69/2260 (3.1% | 84.10  (73.3-91.8) | 77.4  (75.6-79.1) | Sens similar/lower, spec lower |
|  |  | Anaemia | 10 | 38/567 (6.70%) | 81.6 (78.4,84.8) ^a^ | 68.6 (64.8,72.4) ^a^ |  |
| Gerrard 2023^10^  Dual FIT, HM-JACKarc | **1** | **Whole cohort** | 10 | 88/2637 (3.3%) | 96.60  (90.4-99.3) ^a^ | 71.2  (69.4-73.0) ^a^ | Sens similar/lower, spec lower |
|  |  | Anaemia | 10 | 29/480 (6.04%) | 93.1 (90.8,95.4) ^a^ | 60.1 (55.7,64.5) ^a^ |  |
| Withrow 2022^15^  HM-JACKarc | **4** | **Whole cohort (both sexes)** | 10 | 139/16604 (0.84%) | 92.1 (91.7,92.5) ^a^ | 91.5 (91.1,91.9) ^a^ | Sens higher, spec lower |
|  |  | Low Haemoglobin (<130 g/L in men, <120g/L in women) | 10 | 72/507 (1.42%) | 95.8 (95.2,96.4) ^a^ | 88 (87.1,88.9) ^a^ |  |
|  |  | **Whole cohort (men)** | 10 | 83/7019 (1.18%) | 92.8 (92.2,93.4) ^a^ | 90.3 (89.6,91) ^a^ | Sens same or higher, spec lower with increasing anaemia |
|  |  | Men, <130 g/L | 10 | 46/2091 (2.20%) | 93.5 (92.4,94.6) ^a^ | 85.5 (84,87) ^a^ |  |
|  |  | Men, <120 g/L | 10 | 36/1141 (3.16%) | 91.7 (90.1,93.3) ^a^ | 82.7 (80.5,84.9) ^a^ |  |
|  |  | Men, <110 g/L | 10 | 23/494 (4.66%) | 95.7 (93.9,97.5) ^a^ | 79 (75.4,82.6) ^a^ |  |
|  |  | Men, <100 g/L | 10 | 14/216 (6.48%) | 100 (NE,NE) ^a^ | 72.3 (66.3,78.3) ^a^ |  |
|  |  | Men, <90 g/L | 10 | 9/89 (10.11%) | 100 (NE,NE) ^a^ | 71.2 (61.8,80.6) ^a^ |  |
|  |  | **Whole cohort (women)** | 10 | 57/9585 (0.59%) | 91.1 (90.5,91.7) ^a^ | 92.4 (91.9,92.9) ^a^ | Sens higher, spec lower with increasing anaemia |
|  |  | Women, <120g/L | 10 | 25/2758 (0.91%) | 100 (NE,NE) ^a^ | 89.4 (88.3,90.5) ^a^ |  |
|  |  | Women, <110g/L | 10 | 13/1297 (1.00%) | 100 (NE,NE) ^a^ | 88 (86.2,89.8) ^a^ |  |
|  |  | Women, <100g/L | 10 | 6/491 (1.22%) | 100 (NE,NE) ^a^ | 84.5 (81.3,87.7) ^a^ |  |
|  |  | Women, <90g/L | 10 | 3/189 (1.59%) | 100 (NE,NE) ^a^ | 79.6 (73.9,85.3) ^a^ |  |
| **Non-comparative studies** | | | | | | | |
| **Ayling 2019^3^**  OC-Sensor | 4 | Anaemia | 10 | 7/178 (3.93%) | 71.4 (64.8,78) ^a^ | 95.9 (93,98.8) ^a^ |  |
| **Ayling 2019^3^**  OC-Sensor | 4 | IDA | 10 | 6/137 (4.38%) | 68.7 (60.9,76.5) ^a^ | 95.4 (91.9,98.9) ^a^ |  |
| Morales-Arraez 2018^6^  OC-Sensor | 4 | Moderate-severe IDA | 10 | 28/245 (11.43%) | 92.9 (89.7,96.1) ^a^ | 57.1 (50.9,63.3) ^a^ |  |
| Rodriguez-Alonso 2020^8^ | 4 | IDA | 10 | 9/120 (7.5% | 100 (NE,NE) ^a^ | 77.5 (70,85) ^a^ |  |

95% CI, 95% confidence interval; CRC, colorectal cancer; IDA, iron deficiency anaemia; N, number; NE, not estimable

^a^calculated by EAG reviewer

#### *1.2* Age

Three studies^2 14 15^ reported data according to age groups (see Table 2). All were large studies with >5000 patients, the largest included 16,604 patients.^15^ All studies used HM JACKarc.

One study^14^ reported the optimal cut off (the point that maximises both sensitivity and specificity) based on the ROC curves for those aged under 60 years and aged 60+ years separately, and reported that the optimal threshold was lower in the 60+ age group (19 µg/g) compared to the younger age group (37 µg/g). This study concluded that FIT could be incorporated into a risk score based on sex, age, symptoms and signs, drug history, and blood parameters, but did not conduct the analyses required to produce such a score in that publication. It should be noted that the definition of “optimal” in this study is not necessarily the same as optimising the threshold for cost-effectiveness or clinical decision making, where it may be preferable to optimise either sensitivity (where the test is used to rule out disease) or specificity (where the tests is used to rule in disease).

Another study^2^ reported a limited range of thresholds (2, 10 and 150 µg/g) for those aged under 50 years and aged 50+years. Sensitivity was lower in the younger age group at any given threshold though confidence intervals overlapped. This trend was less evident at the highest threshold and the number of events in the younger age group was small (n=16). In this study the authors noted that in younger patients it may be appropriate to intepret any detectable faecal Hb as a positive test.

In the third study,^15^ the thresholds were 2 µg/g and 10 µg/g and were reported for those aged under 40 years, then for those aged 40+, 50+, 60+, 70+ and 80+ years. This study performed multivariable modelling including FIT, blood tests, age, and sex and concluded that that age-specific thresholds for FIT positivity would not improve test performance.

Overall, there is some indication that FIT thresholds may need to be lower in younger patients in order to achieve the same sensitivity as for older patients. The available data does not provide conclusive evidence that different FIT thresholds should be used or what they should be.

**Table 2: Sensitivity and specificity by age**

| **#** | **Author, year**  **Location**  **Recruitment dates**  **Study name (if available)** | **Analyser**  **Reference standard** | **Popul-ation type** | **N with CRC/ N analysed (%)** | **Thres-hold, µg/g** | **Age group in years** | **Sensitivity (95% CI)** | **Specificity (95% CI)** | **Conclusion drawn by study authors** |
| --- | --- | --- | --- | --- | --- | --- | --- | --- | --- |
| 1 | D'Souza 2021b^2^  NICE FIT  October 2017 to December 2019 | HM JACKarc analytical system  Colonoscopy | **4** | 16/1103 (1.45%) | 2 | <50 | 87.50 (61.7–98.4) | 70.40 (67.6–73.1) | Detectable f-Hb on FIT in symptomatic younger patients may indicate referral for investigation of colorectal cancer and serious bowel disease. |
|  |  |  |  | 313/8719 (3.59%) | 2 | 50+ | 97.40 (95.0–98.9) | 64.10 (63.1–65.2) |  |
|  |  |  |  | 16/1103 (1.45%) | 10 | <50 | 81.30 (54.4–96.0) | 83.60 (81.3–85.5) |  |
|  |  |  |  | 313/8719 (3.59%) | 10 | 50+ | 91.40 (87.7–94.2) | 83.50 (82.7–84.3) |  |
|  |  |  |  | 16/1103 (1.45%) | 150 | <50 | 68.80 (41.3–89.0) | 92.20 (90.4–93.7) |  |
|  |  |  |  | 313/8719 (3.59%) | 150 | 50+ | 70.90 (65.6–75.9) | 94.90 (94.4–95.3) |  |
| 2 | Turvill 2021^14^  Yorkshire & Humber, UK  April 2018 to Dec 2019  Fast track FIT | HM JACKarc  Full colonoscopy or CT colonography, or a lesser investigation (such as CT abdomen/pelvis with contrast or flexible sigmoidoscopy) | **4** | 30/1217 (2.5%) | 37 | <60 | 90.00 (73.5-97.9) | 87.40 (85.4-89.3) | The optimal  cut-off value for people aged ≥60 years  (19 µg/g faeces) is lower than for those  aged <60 years (37 µg/g faeces). FIT could be incorporated into a risk score based on sex, age, symptoms and signs, drug history, and blood parameters |
|  |  |  |  | 19/3823 (0.49%) | 19 | 60+ | 83.50 (75.6-89.6) | 85.40 (84.2-86.5) |  |
| 3 | Withrow 2022^15^ (same study as Nicholson 2020)^16^  Oxfordshire, UK  March 2017 to December 21, 2020  CSS-BIO-3 4730 | HM JACKarc  Records follow-up | **4** | 9/1390 (0.65%) | 2 | <40 | 100 (70.1 -100) | 89.1 (87.4 to 90.7) | The lack of  an apparent age-effect after taking into account FIT suggests that age-specific thresholds for FIT positivity would  not improve test performance |
|  |  |  |  | 130/15214 (0.85%) | 2 | >40 | 96.2 (91.3-98.3) | 83.0 (82.4-83.6) |  |
|  |  |  |  | 118/12936 (0.91%) | 2 | >50 | 95.8 (90.5-98.2) | 81.8 (81.1-82.4) |  |
|  |  |  |  | 98/8755 (1.12%) | 2 | >60 | 94.9 (88.6-97.8) | 78.8 (77.9-79.7) |  |
|  |  |  |  | 77/3043 (2.53%) | 2 | >70 | 94.8 (87.4-98) | 51.8 (50-53.6) |  |
|  |  |  |  | 41/2527 (1.62%) | 2 | >80 | 95.1 (83.9-98.7) | 68.4 (66.6-70.2) |  |
|  |  |  |  | 9/1390 (0.65%) | 10 | <40 | 88.9 (56.5-98) | 93.4 (92-94.6) |  |
|  |  |  |  | 130/15214 (0.85%) | 10 | >40 | 92.3 (86.4-95.8) | 91.3 (90.9-91.8) |  |
|  |  |  |  | 118/12936 (0.91%) | 10 | >50 | 91.5 (85.1-95.3) | 90.7 (90.2-91.2) |  |
|  |  |  |  | 98/8755 (1.12%) | 10 | >60 | 89.8 (82.2-94.4) | 89.0 (88.3-89.6) |  |
|  |  |  |  | 77/5863 (1.31%) | 10 | >70 | 89.6 (80.8-94.6) | 87.1 (86.2-87.9) |  |
|  |  |  |  | 41/2533 (1.62%) | 10 | >80 | 87.8 (74.5-94.7) | 83.1 (81.6-84.5) |  |

95% CI, 95% confidence interval; CRC, colorectal cancer; N, number

#### *1*.3 Sex

Three studies^14 16 17^ reported data for men and women separately (see Table 3). All were studies with >3000 patients, with the largest including 9,899 patients.^16^ One study used OC-Sensor PLEDIA^17^ and two used HM-JACKarc.^14 16^

One study^14^ reported the optimal cut off (the point that maximises both sensitivity and specificity), based on the ROC curves for men and women separately, and reported that the optimal threshold was lower for women (16 µg/g) than for men (21 µg/g). This study concluded that FIT could be incorporated into a risk score based on sex, age, symptoms and signs, drug history, and blood parameters. It should be noted that the definition of “optimal” in this study is not necessarily the same as optimising the threshold for cost-effectiveness or clinical decision making, where it may be preferable to optimise either sensitivity (where the test is used to rule out disease) or specificity (where the tests is used to rule in disease).

The two other studies^15 17^ reported a range of thresholds (from 10 to 150µg/g), and generally showed that at thresholds above 10 µg/g, sensitivity and specificity is higher in women than in men. This difference was more pronounced in one study^17^ than the other,^16^ but due to the small number of studies it was not possible to tell if this was due to the use of different analysers or some other factor. At 10µg/g, one study showed roughly equivalent sensitivity and specificity,^16^ whilst the other study showed numerically lower sensitivity in men, but stated that no significant difference in FIT sensitivity was found.^17^ Withrow 2022 conducted a multivariable analysis including sex and showed the probability of colorectal cancer reached 3% at 17 and 25 µg/g for males and females, respectively.

If sensitivity and specificity are different in women than in men at a given threshold, a different threshold in women may be required to achieve equivalent sensitivity and specificity in the two sexes. However, it was not possible on the basis of the available data to conclude what and whether different FIT cut-off values are required according to sex.

**Table 3: Sensitivity and specificity by sex**

| **#** | **Author, year**  **Location**  **Recruitment dates**  **Study name (if available)** | **Analyser**  **Reference standard** | **Inclusion criteria** | **N with CRC/ N analysed (%)** | **Threshold, µg/g** | **Men: Sensitivity (95% CI)** | **Men: Specificity (95% CI)** | **Women: Sensitivity (95% CI)** | **Women: Specificity (95% CI)** | **Conclusion drawn by study authors** |
| --- | --- | --- | --- | --- | --- | --- | --- | --- | --- | --- |
| **1** | **Ball 2022^17^ (additional data by personal communication)**  Sheffield, UK  Oct 2019 to Dec 2019 | OC-Sensor PLEDIA  Colonoscopy or CT imaging and colon capsule endoscopy | 4 | Men: 25/1566 (1.6%)  Women: 20/1940 (1.03%) | 10 | 84.00 (63.1–94.7) | 79.20  (77.0–81.2) | 100.00 (80 -100) | 82.00 (80.2–83.7) | Sex did not significantly influence FIT sensitivity on subgroup  analysis. |
|  |  |  |  |  | 20 | 80.00 (58.7–92.4) | 85.40 (83.5–87.1) | 95.00 (73.1–99.7) | 88.80 (87.2–90.2) |  |
|  |  |  |  |  | 50 | 68.00 (46.4–84.3) | 91.60 (90.0–92.9) | 80.00 (55.7–93.3) | 94.10 (92.9–95.1) |  |
|  |  |  |  |  | 80 | 64.00 (42.6–81.3) | 93.90 (92.6–95.0) | 70.00 (45.7–87.2) | 95.80 (94.8–96.6) |  |
|  |  |  |  |  | 100 | 64.00 (42.6–81.3) | 94.60 (93.3–95.7) | 70.00 (45.7–87.2) | 96.70 (95.8–97.4) |  |
|  |  |  |  |  | 120 | 60.00 (38.9–78.2) | 95.20 (94.0–96.2) | 65.00 (40.9–83.7) | 97.00 (96.1–97.7) |  |
|  |  |  |  |  | 150 | 52.00 (31.8–71.7) | 96.40 (95.3–97.2) | 55.00 (32.0–76.2) | 97.30 (96.5–98.0) |  |
| 2 | Turvill 2021^14^  Yorkshire & Humber, UK  April 2018 to Dec 2019  Fast track FIT | HM JACKarc  Full colonoscopy or CT colonography, or a lesser investigation (such as CT abdomen/pelvis with contrast or flexible sigmoidoscopy) | **4** | Men: 89/2242 (3.9%)  Women: 62/2798 (2.2%) | Men: 21  Women: 16  NB: optimal threshold was derived | 85.40 (76.3 to 92.0) | 83.70 (82.0 to 85.2) | 87.10 (76.1 to 94.3) | 85.60 (84.2 to 86.9) | The optimal  cut-off value  is lower for females (16 µg/g faeces)  than for males (21 µg/g faeces. FIT could be incorporated into a risk score based on sex, age, symptoms and signs, drug history, and blood parameters |
| 3 | Nicholson 2020 (same study as Withrow 2022)^16a^  Oxfordshire, UK  March 2017 to December 21, 2020  CSS-BIO-3 4730 | HM JACKarc  Records follow-up | **4** | Men: 65/4104 (1.6%)  Women: 40/5795 (0.69%) | 7 | 92.30 (85.8-98.8) | 87.90 (86.9-88.9) | 90.00 (80.7-99.3) | 91.10 (90.3-91.8) | The area under the curve for all adults did not change  substantially by gender.  From Withrow 2022: The probability of colorectal cancer reached 3% at 17 and 25 µg/g, for males and females respectively. |
|  |  |  |  |  | 10 | 90.80 (83.7-97.8) | 89.80 (88.8-90.7) | 90.00 (80.7-99.3) | 92.40 (91.8-93.1) |  |
|  |  |  |  |  | 20 | 83.10 (74.0-92.2) | 92.30 (91.5-93.2) | 87.50 (77.3-97.7) | 94.60 (94.1-95.2) |  |
|  |  |  |  |  | 50 | 73.80 (63.2-84.5) | 95.50 (94.9-96.2) | 75.00 (61.6-88.4) | 96.90 (96.5-97.4) |  |
|  |  |  |  |  | 100 | 60.00 (48.1-71.9) | 96.80 (96.3-97.3) | 62.50 (47.5-77.5) | 98.10 (97.8-98.5) |  |
|  |  |  |  |  | 120 | 55.40 (43.3-67.5) | 97.20 (96.7-97.7) | 60.00 (44.8-75.2) | 98.30 (98.0-98.6) |  |
|  |  |  |  |  | 150 | 50.80 (38.6-62.9) | 97.50 (97.1-98.0) | 60.00 (44.8-75.2) | 98.50 (98.2-98.8) |  |

95% CI, 95% confidence interval; CRC, colorectal cancer; N, number

^a^ Nicholson 2020 (n=9896) is an earlier data cut of the same study as Withrow 2022

(n=11,142). The data from Nicholson 2020 has been included in this analysis over the Withrow 2022 data as it reports more thresholds, even though the study population is smaller. However, the Withrow 2022 study conducted a multivariable analysis including sex, and the conclusions relating to this have been reported.

#### *1.*4 Medications that might cause GI bleeding

The scope issued by NICE states the assessment should consider whether the FIT threshold should be different for “People taking medications or with conditions which increase the risk of gastrointestinal bleeding”. In a slight widening of the scope, NICE confirmed an additional study which looked at the effect of taking proton pump inhibitors (PPI), which may decrease the risk of gastrointestinal bleeding, may be of interest to the committee and it has therefore been included.

Consequently, three studies^4 7 14^ were included in this subgroup analysis. The studies are summarised in Table 4. All studies included more than 1000 patients, with the largest including 5040 in total.^14^ Two studies (three references) used OC-Sensor analysers,^4 5 7^ and one used HM-JACKarc.^14^

One study^14^ reported the optimal cut off (the point that maximises both sensitivity and specificity) based on the ROC curves for those using antiplatelet, anticoagulants NSAIDs and those not using these drugs. The optimal threshold was 19µg/g in both cases, though the sensitivity and specificity were superior in those not using the drugs than in those who were. This study concluded that FIT could be incorporated into a risk score based on sex, age, symptoms and signs, drug history, and blood parameters.

Another study (part of the “colonpredict” study)^4 5^ reported test accuracy data for those using aspirin and those not using aspirin. It should be noted that this study recruited symptomatic patients from secondary as well as primary care and was therefore excluded from the main analysis. The analysis of aspirin users was included due to the sparsity of data in this subgroup, but it is unclear how generalisable these results will be to the primary care setting. Only one threshold was included (20 µg/g). As with the previous study, the sensitivity and specificity were superior in those not using the drug. This study concluded that aspirin use did not change the diagnostic accuracy of FIT in patients with gastrointestinal symptoms.

The third study compared PPI users to PPI non-users. It should be noted that this study recruited symptomatic patients from secondary as well as primary care and was therefore excluded from the main analysis. The analysis of PPI users was included due to the sparsity of data in this subgroup, but it is unclear how generalisable these results will be to the primary care setting. At a threshold of 20 µg/g sensitivity was similar, and specificity was slightly higher in non-users. This study did not conclude anything for the detection of CRC, but concluded there was impaired FIT performance in PPI users for the detection of advanced neoplasia.

Conclusion: The evidence base is currently small, and it was not possible on the basis of the available data to conclude what and whether different FIT cut-off values are required according to medications being taken by a patient.

**Table 4: Sensitivity and specificity for patients taking medications that may affect the risk of GI bleeding**

| **#** | **Author, year**  **Location**  **Recruitment dates**  **Study name (if available)** | **Analyser**  **Reference standard** | **Inclusion criteria** | **Group** | **N with CRC/ N analysed (%)** | **Thres-hold, µg/g** | **Sensitivity (95%CI)** | **Specificity (95%CI)** | **Conclusion drawn by study authors** |
| --- | --- | --- | --- | --- | --- | --- | --- | --- | --- |
| 1 | Bujanda 2018^4^  Spain (assume Ourense and San Sebastian)^5^  March 2012 to 2014  COLONPREDICT | - OC-Sensor^5^   Colonoscopy | Population type 4 - Symptomatic patients referred from primary and secondary care | Aspirin users | 51/485 (10.51%) | 20 | 88.00 (75-95) | 66.97 (62-71) | Aspirin use did not change the diagnostic accuracy of FIT in patients with gastrointestinal  symptoms. |
|  |  |  |  | Aspirin non-users | 299/2567 (11.65%) | 20 | 92.00 (88-95) | 71.00 (69-73) |  |
| 2 | Turvill 2021^14^  Yorkshire & Humber, UK  April 2018 to Dec 2019  Fast track FIT | HM JACKarc  Full colonoscopy or CT colonography, or a lesser investigation (such as CT abdomen/pelvis with contrast or flexible sigmoidoscopy) | Population type 4 - 2WW patients | Antiplatelets, anticoagulants NSAIDs | 19/1356 (1.4%) | 19 | 82.40 (69.1-91.6) | 80.50 (78.2-82.6) | The specificity differed according to use of antiplatelets, anticoagulants NSAIDs. FIT could be incorporated into a risk score based on sex, age, symptoms and signs, drug history, and blood parameters |
|  |  |  |  | No use of antiplatelets, anticoagulants NSAIDs | 100/3684 (2.7%) | 19 | 87.0 (78.8-92.9) | 86.9 (85.7-88.0) |  |
| 3 | Rodriguez-Alonso 2018^7^  Barcelona, Spain  Sept 2011 to Oct 2012 | OC-Sensor MICRO  Colonoscopy | Population type 4 - Symptomatic patients referred from primary and secondary care | PPI users | 15/525 (2.86%) | 20 | 93.3 | 85.1 | No conclusion drawn for the identification of CRC in PPI users, concluded impaired FIT performance in PPI users for detection of advanced neoplasia. |
|  |  |  |  | PPI non-users | 15/477 (3.14%) | 20 | 93.3 | 87.4 |  |

95% CI, 95% confidence interval; CRC, colorectal cancer; N, number

#### *1.5* Ethnicity

No studies reporting the diagnostic test accuracy of any of the in-scope tests according to ethnicity were identified.

#### *1.6* People with blood disorders

No studies reporting the diagnostic test accuracy of any of the in-scope tests in a subgroup of people with blood disorders (e.g., beta thalassemia) that could affect the performance of the test were identified.

1. Cunin L, Khan AA, Ibrahim M, et al. FIT negative cancers: A right-sided problem? Implications for screening and whether iron deficiency anaemia has a role to play. *The Surgeon* 2021;19(1):27-32. doi: <https://doi.org/10.1016/j.surge.2020.02.003>

2. D’Souza N, Monahan K, Benton SC, et al. Finding the needle in the haystack: the diagnostic accuracy of the faecal immunochemical test for colorectal cancer in younger symptomatic patients. *Colorectal Disease* 2021b;23(10):2539-49.

3. Ayling RM, Lewis SJ, Cotter F. Potential roles of artificial intelligence learning and faecal immunochemical testing for prioritisation of colonoscopy in anaemia. *British Journal of Haematology* 2019;185(2):311-16. doi: <https://doi.org/10.1111/bjh.15776>

4. Bujanda L, Sarasqueta C, Vega P, et al. Effect of aspirin on the diagnostic accuracy of the faecal immunochemical test for colorectal advanced neoplasia. *United European Gastroenterol J* 2018;6(1):123-30. doi: 10.1177/2050640617707094 [published Online First: 2018/02/13]

5. Cubiella J, Salve M, Díaz-Ondina M, et al. Diagnostic accuracy of the faecal immunochemical test for colorectal cancer in symptomatic patients: comparison with NICE and SIGN referral criteria. *Colorectal Dis* 2014;16(8):O273-82. doi: 10.1111/codi.12569 [published Online First: 2014/01/25]

6. Morales Arraez D, Carrillo G, Adrian M, et al. Role of faecal immunochemical testing in the diagnostic workup of patients with iron deficiency anaemia. *United Eur Gastroenterol J* 2018;6:A403–A04.

7. Rodriguez-Alonso L, Rodriguez-Moranta F, Arajol C, et al. Proton pump inhibitors reduce the accuracy of faecal immunochemical test for detecting advanced colorectal neoplasia in symptomatic patients. *PLoS One* 2018;13(8):e0203359.

8. Rodriguez-Alonso L, Rodriguez-Moranta F, Ruiz-Cerulla A, et al. The use of faecal immunochemical testing in the decision-making process for the endoscopic investigation of iron deficiency anaemia. *Clin Chem Lab Med* 2020;58(2):232-39. doi: 10.1515/cclm-2019-0203 [published Online First: 2019/12/01]

9. D’Souza N, Delisle TG, Chen M, et al. Faecal immunochemical testing in symptomatic patients to prioritize investigation: diagnostic accuracy from NICE FIT Study. *British Journal of Surgery* 2021a;108(7):804-10. doi: 10.1093/bjs/znaa132

10. Gerrard AD, Maeda Y, Miller J, et al. Double faecal immunochemical testing in patients with symptoms suspicious of colorectal cancer. *British Journal of Surgery* 2023;110(4):471-80. doi: 10.1093/bjs/znad016

11. Johnstone MS, Burton P, Kourounis G, et al. Combining the quantitative faecal immunochemical test and full blood count reliably rules out colorectal cancer in a symptomatic patient referral pathway. *International Journal of Colorectal Disease* 2022a;37(2):457-66.

12. Juul JS, Hornung N, Andersen B, et al. The value of using the faecal immunochemical test in general practice on patients presenting with non-alarm symptoms of colorectal cancer. *British Journal of Cancer* 2018;119(4):471-79. doi: <https://dx.doi.org/10.1038/s41416-018-0178-7>

13. Tang A, Chandler S, Torkington J, et al. Adapting the investigation of patients on urgent suspected cancer pathway with lower gastrointestinal symptoms across Wales during COVID-19. *Annals of the Royal College of Surgeons of England* 2022;26:26.

14. Turvill J, Turnock D, Cottingham D, et al. The Fast Track FIT study: diagnostic accuracy of faecal immunochemical test for haemoglobin in patients with suspected colorectal cancer. *Br J Gen Pract* 2021;71:E643–E51.

15. Withrow DR, Shine B, Oke J, et al. Combining faecal immunochemical testing with blood test results for colorectal cancer risk stratification: a consecutive cohort of 16,604 patients presenting to primary care. *BMC Medicine* 2022;20(1):116.

16. Nicholson BD, James T, Paddon M, et al. Faecal immunochemical testing for adults with symptoms of colorectal cancer attending English primary care: a retrospective cohort study of 14 487 consecutive test requests. *Alimentary Pharmacology & Therapeutics* 2020;52(6):1031-41.

17. Ball AJ, Aziz I, Parker S, et al. Fecal Immunochemical Testing in Patients With Low-Risk Symptoms of Colorectal Cancer: A Diagnostic Accuracy Study. *Journal of the National Comprehensive Cancer Network* 2022;20(9):989-96.e1.
