# Supplementary material for: Faecal immunochemical tests for patients with symptoms suggestive of colorectal cancer: An updated systematic review and multiple‐threshold meta‐analysis of diagnostic test accuracy studies
Source: Colorectal Dis. 2024 Dec 17;27(1):e17255. doi: 10.1111/codi.17255 (PMC11683176; doi:10.1111/codi.17255)

### **Additional Analysis 2 – Reference standard sensitivity analysis**

Subgroup analyses were conducted that included only the studies where at least 90% of the participants received colonoscopy as the reference standard. Subgroups analyses were considered for all FIT tests together, including all population types, excluding population type 4 studies, and separately for each test (where data allowed).

Summary estimates of sensitivity and specificity are illustrated in **Figure *1***. For all analyses the summary estimates were similar, irrespective of the reference standard grouping (all studies vs at least 90% of the participants receiving colonoscopy). The largest difference in point estimates was seen for specificity of OC-Sensor (**Figure *1*** F); however, there were only 3 studies in the >90% colonoscopy subgroup and so the apparent difference may be explained by other sources of heterogeneity between the studies. There was very little difference in specificity for the HM-JACKarc studies (**Figure *1*** D).

**Figure 1: Summary sensitivity and specificity, reference standard sensitivity analysis**


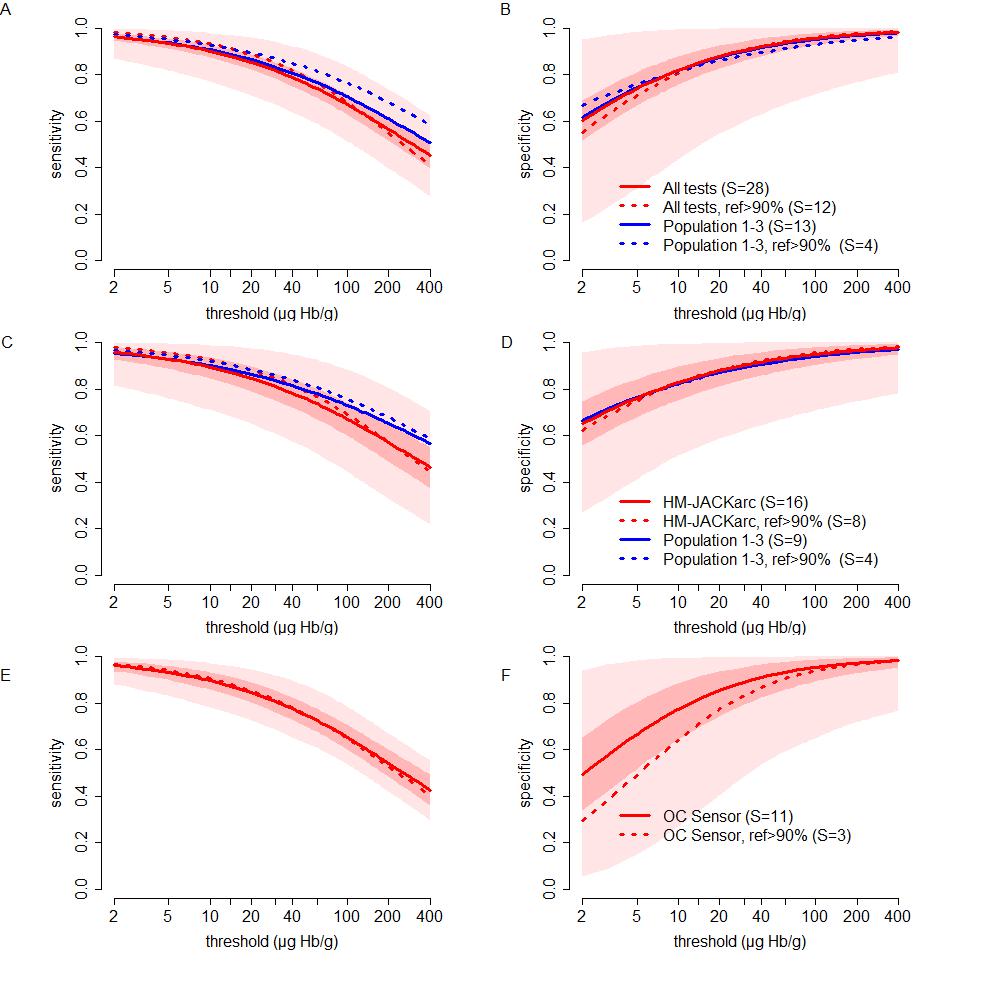

Supplement: Supplementary file 15 — Data S15. [file CODI-27-0-s013.docx]
