# Supplementary material for: Faecal immunochemical tests for patients with symptoms suggestive of colorectal cancer: An updated systematic review and multiple‐threshold meta‐analysis of diagnostic test accuracy studies
Source: Colorectal Dis. 2024 Dec 17;27(1):e17255. doi: 10.1111/codi.17255 (PMC11683176; doi:10.1111/codi.17255)
Supplement: Supplementary file 16 — Data S16. [file CODI-27-0-s001.docx]

### **Advanced adenomas and inflammatory bowel disease outcomes**

This supplement reports the statistical syntheses for AA (section 1.1) and IBD (section 1.2).

Nine studies (10 publications)^1-10^ reported data on AA and IBD. These are summarised in Table 1. One study reported data for the IDK Hb and Hb/Hp complex ELISA tests, whilst the remainder reported data for immunoturbidimetry tests. The synthesis focussed on the immunoturbidimetry tests, but the IDK data was used in the model for the IDK tests.

#### 1.1 Statistical synthesis of AA outcomes

Nine studies^1-9^ contributed to the meta-analysis for AA outcomes (HM-JACKarc: 6, OC-Sensor: 2 QuikRead Go: 1). Five studies provided diagnostic accuracy at a single threshold and the maximum number of thresholds considered by a single study was 3. The full dataset (all studies) provided a total of 15 pairs of sensitivity and specificity, at thresholds between 2 and 150. Figure ***1***A and Figure ***1***B illustrate the results for all studies, irrespective of test type. Separate syntheses are also provided for HM-JACKarc (Figure ***1***C) and OC-Sensor (Figure ***1***D).

One of the studies^3^ also reported data for AA and IBD when using Dual FIT and is reported in section 4.3.7.

For the analysis of all test types together, sensitivity ranges from 80.4 (95% CrI: 55.8, 98.3; 95% PrI: 50.0, 100.0) at a threshold of 2, to 20.4 (95% CrI: 0.6, 47.5; 95% PrI: 0, 57.4) at a threshold of 150. Specificity ranges from 51.6 (95% CrI: 31.6, 71.1; 95% PrI: 3.2,98) at a threshold of 2, to 95.7 (95% CrI: 82.5, 99.5; 95% PrI: 58.5,100) at a threshold of 150. There is a large amount of heterogeneity between studies, as illustrated by the wide 95% CrI and PrI. Point estimates of summary sensitivity and specificity changed considerably for the separate analyses by test type (see Figure 1 C, D and Table 2), emphasising the large amount of uncertainty.

**Table 1: Studies reporting data on AA and IBD**

| **#** | **Author, year**  **Location**  **Recruitment dates**  **Study name (if available)** | **Analyser**  **Reference standard** | **Population types** | **N with AA / N analysed (%)**  **N with IBD / N analysed (%)** | **Thresholds, µg/g** |
| --- | --- | --- | --- | --- | --- |
| 1 | Sieg 1999^10^  Ostringen, Germany  NR, prior to publication in 1999 | Immunological test for Hb/Hp complex  Colonoscopy | 4 | AA: 37/621 (5.95%)  IBD: 22/621 (3.5%) | 2 |
|  |  | Immunological test for HB  Colonoscopy |  |  |  |
| 2 | D'Souza 2020a^2^  Croyden, UK  Nov 2016 to Oct 2017 | HM JACKarc analytical system  Colonoscopy | 1,2,3 | AA (population 1): 4/298 (1.3%)  IBD (population 1): 12/298 (4.0%) | 2, 10 |
| 3 | D'Souza 2021a^11^  D'Souza 2021c^1d^  NICE FIT  October 2017 to December 2019 | HM JACKarc analytical system  Colonoscopy | 4 | AA: 421/982 (4.3%)  IBD: 427/9822 (4.3%) | 2, 10, 150 |

| 4 | Gerrard 2023^3^  Lothian, Scotland, UK  Jan 2019 to Feb 2020 | HM-JACKarc  Endoscopy or CT with colorectal protocol. | 1 | AA: 105/2260 (4.6%) and 136/3426 (4.0%)  IBD: 59/226 (2.6%) and 55/3426 (1.6%) | 10 |
| --- | --- | --- | --- | --- | --- |
| 5 | Juul 2018^4^  Central Denmark  Sept 2015 to Aug 2016  NCT02308384 | OC-Sensor DIANA  Records follow-up | 4 | AA: 68/3462 (1.9%)  IBD: 31/3462 (0.9%) | 10 |
| 6 | MacDonald 2022^5^  NHS Lanarkshire, Scotland, UK  October 2016 to February 2019 | HM-JACKarc  Records follow-up | 1 | AA: 47/5250 (0.9%)  IBD: 131/5250 (2.5%) | 10 |
| 7 | MacLean 2021b  Royal Surrey Foundation Trust, UK  July 2019 and March 2020 | QuikRead go  Colonoscopy, CTC or flexisig | 2 | AA: 29/553 (5.2%)  IBD: 9/553 (1.6%) | 10, 100, 150 |
| 8 | Mowat 2016^9^  NHS Tayside, Scotland, UK  Oct 2013 to March 2014 | OC-Sensor iO  Colonoscopy | 4 | AA: 40/750 (5.3%)  IBD: 34/750 (4.5%) | 4, 10 |
| 9 | Mowat 2021^8^ & 2019^7^  NHS Tayside, Scotland, UK  December 2015 to December 2016 | HM JACKarc  Records follow-up | 4 | AA: 133/1447 (9.2%)  IBD: 68/1447 (4.7%) | 10 |

AA, advanced adenomas; IBD, inflammatory bowel disease; N, number

**Figure 1: Observed data and summary sensitivity and specificity for AA outcomes. A) All tests, ROC B) All tests as a function of threshold, C) HM-JACKarc D) OC-Sensor**

**
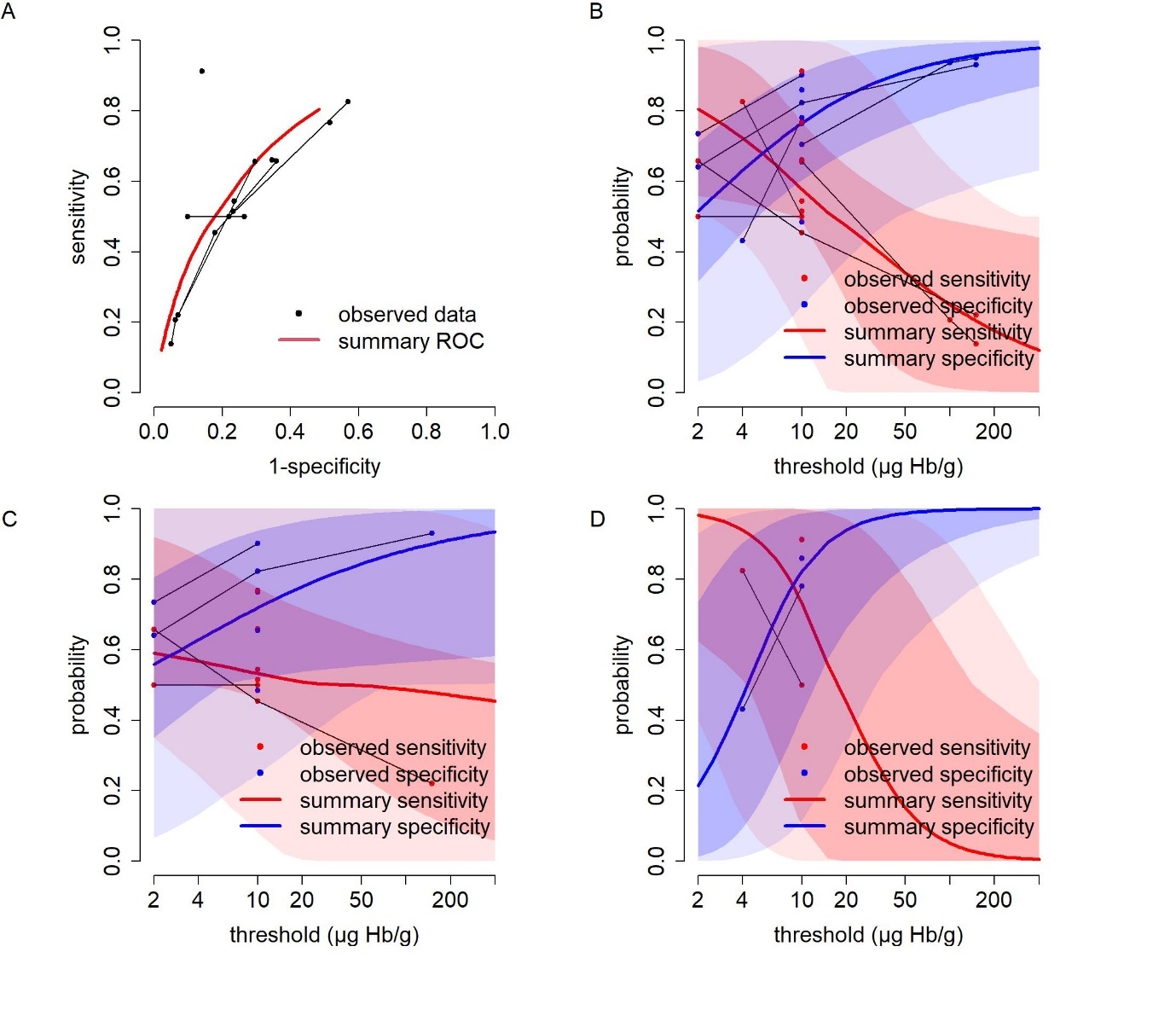
**

*Results for OC-Sensor use an informative prior, based on the synthesis of all tests together.*

**Table 2: Summary sensitivity and specificity at selected thresholds for AA outcome**

| **threshold, µg/g** | **All tests (S=9)** | | **HM-JACKarc (S=6)** | | **OC-Sensor (S=2)** | |
| --- | --- | --- | --- | --- | --- | --- |
|  | **sensitivity** | **specificity** | **sensitivity** | **specificity** | **sensitivity** | **specificity** |
| **2** | 80.4 (55.8,98.3) | 51.6 (31.6,71.1) | 59.1 (50,92) | 55.9 (35.1,80.6) |  |  |
| **2.5** | 78 (55.2,97.4) | 55.4 (36.1,74.4) | 58.4 (50,90.5) | 58.1 (38.4,82.9) |  |  |
| **3** | 75.9 (54.7,96.4) | 58.4 (39.6,77.1) | 57.7 (50,89.3) | 59.9 (41,84.6) |  |  |
| **4** | 72.2 (53.7,93.8) | 63.1 (45.6,81) | 56.7 (49.8,86.8) | 62.8 (45.1,87.3) | 93.9 (51.5,100) | 46.8 (9.5,90.3) |
| **7** | 63.9 (51.4,84.6) | 71.7 (55.3,87.7) | 54.7 (48.2,81.2) | 68.5 (50.8,91.7) | 84.6 (27.8,100) | 70.7 (27.2,96.7) |
| **10** | 57.7 (48.6,76.7) | 76.5 (60.3,90.9) | 53.2 (45.9,77.6) | 71.9 (52,93.8) | 73.2 (10.1,99.9) | 82.2 (41.6,98.7) |
| **20** | 47.4 (26.1,64.4) | 84.2 (68.1,95.3) | 50.9 (37.3,71.6) | 77.9 (53.7,96.5) |  |  |
| **50** | 34.1 (5.6,53.2) | 91.1 (75.7,98.2) | 49.8 (24.3,65.7) | 84.4 (55.4,98.5) |  |  |
| **100** | 25 (1.4,48.9) | 94.4 (80.2,99.2) | 48.7 (16,61.9) | 88.2 (56.3,99.2) |  |  |
| **120** | 22.8 (1,48.3) | 95 (81.3,99.3) | 48.3 (14.2,61.1) | 89.1 (56.6,99.4) |  |  |
| **150** | 20.4 (0.6,47.5) | 95.7 (82.5,99.5) | 47.8 (12.3,60.1) | 90.1 (56.9,99.5) |  |  |

#### 1.2 Statistical synthesis of Inflammatory bowel disease outcomes

Nine studies contributed to the meta-analysis for IBD outcome (HM-JACKarc: 6, OC-Sensor: 2 QuikRead go: 1). 5 provided diagnostic accuracy at a single threshold and the maximum number of thresholds considered by a single study was 3. The full dataset (all studies) provided a total of 15 pairs of sensitivity and specificity, at thresholds between 2 and 150.

Figure 2 A and B illustrates the results for all studies, irrespective of test type. Separate syntheses are also provided for HM-JACKarc (Figure 2C) and OC-Sensor (Figure 2D).

For the analysis of all test types together, sensitivity ranges from 85.7 (95% CrI: 70, 96.7; 95% PrI: 42.3, 100.0) at a threshold of 2, to 41.7 (95% CrI: 15.9, 66.1; 95% PrI: 0.9, 91.4) at a threshold of 150. Specificity ranges from 53.8 (95% CrI: 33.1, 75.5; 95% PrI: 2.6, 99.3) at a threshold of 2, to 95.0 (95% CrI: 80.2, 99.5; 95% PrI: 55.0,100) at a threshold of 150. As with AA, there is a large amount of heterogeneity between studies, as illustrated by the wide 95% CrI and PrI. Point estimates of summary sensitivity and specificity changed considerably for the separate analyses by test type (see Figure 2 C, D and Table 3), emphasising the large amount of uncertainty.

**Figure 2: Observed data and summary sensitivity and specificity for All tests. IBD outcomes**


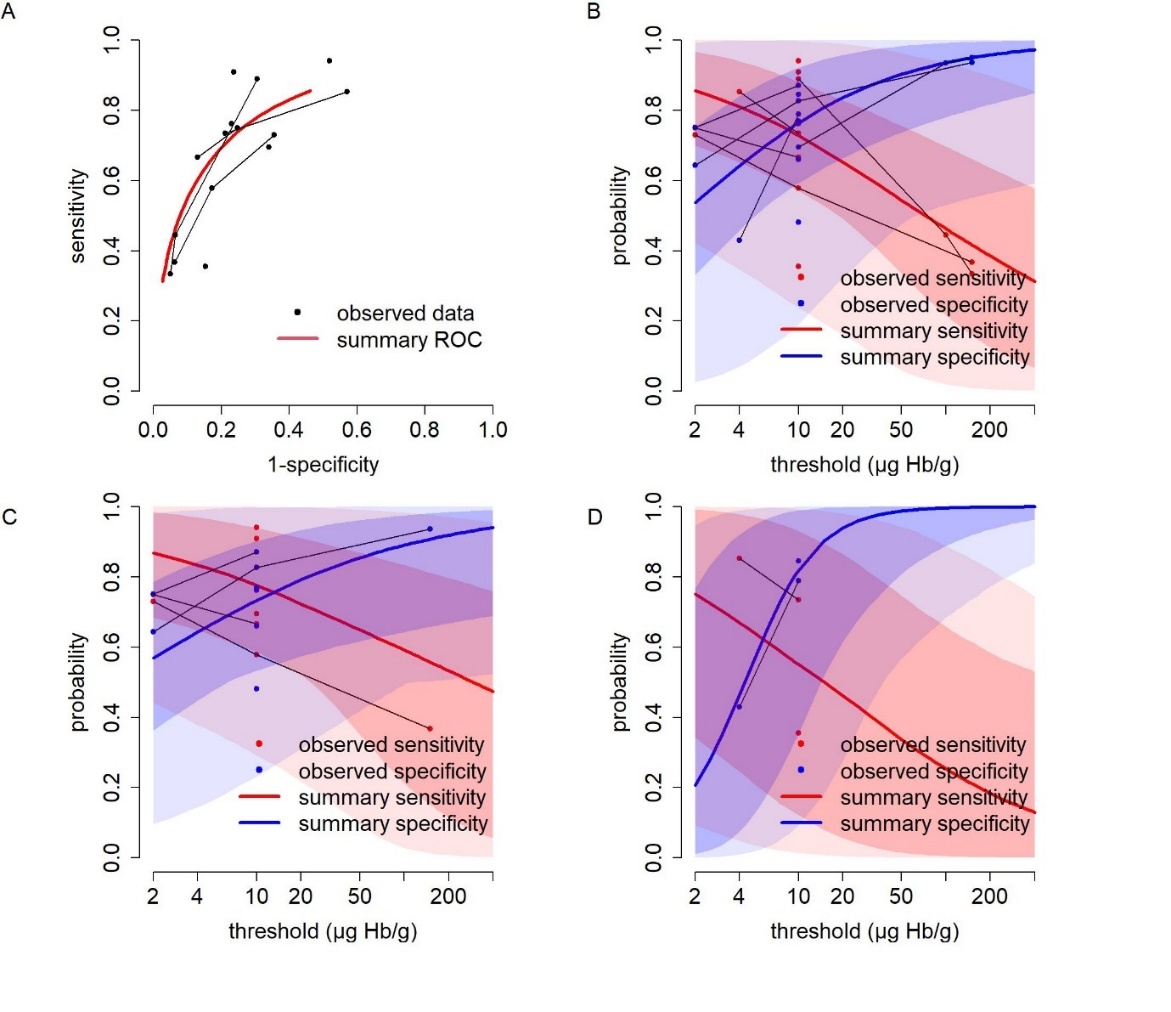


**Table 3: Summary sensitivity and specificity at selected thresholds for IBD outcomes**

| **threshold, µg/g** | **All tests (S=9)** | | **HM-JACKarc (S=6)** | | **OC-Sensor (S=2)** | |
| --- | --- | --- | --- | --- | --- | --- |
|  | **sensitivity** | **specificity** | **sensitivity** | **specificity** | **sensitivity** | **specificity** |
| **2** | 85.7 (70,96.7) | 53.8 (33.1,75.5) | 86.8 (68.4,98.5) | 57 (36.4,78.7) |  |  |
| **2.5** | 84.3 (68.5,96) | 57.2 (37.2,78.4) | 85.8 (67.3,98.1) | 59.4 (39.2,80.6) |  |  |
| **3** | 83.1 (67.2,95.3) | 60 (40.5,80.6) | 84.9 (66.4,97.8) | 61.3 (41.3,82.1) |  |  |
| **4** | 81 (65.1,94) | 64.2 (45.8,84) | 83.4 (64.7,97.1) | 64.3 (44.8,84.4) | 67 (24.7,97.9) | 46.4 (7.4,92) |
| **7** | 76.3 (60.4,90.7) | 72 (54.7,89.4) | 80.1 (61.2,95.3) | 69.9 (50.8,88.1) | 59.8 (16.4,95.5) | 70.3 (22.3,97.5) |
| **10** | 72.9 (57.1,88.2) | 76.4 (59.2,92.1) | 77.6 (58.6,94) | 73.3 (53.3,90.2) | 55.1 (12.2,93.1) | 81.9 (35.3,99) |
| **20** | 65.3 (49.2,82.9) | 83.6 (66.3,95.8) | 72.3 (52.2,91.1) | 79.2 (57.3,93.4) |  |  |
| **50** | 54.4 (33.6,75.5) | 90.3 (73.7,98.3) | 64.9 (35.1,86.9) | 85.4 (61.7,96.2) |  |  |
| **100** | 46.3 (21.7,69.7) | 93.6 (78,99.2) | 59.2 (21.3,83.4) | 89.1 (64.3,97.5) |  |  |
| **120** | 44.2 (19,68.1) | 94.3 (79,99.3) | 57.7 (18.3,82.4) | 89.9 (64.9,97.8) |  |  |
| **150** | 41.7 (15.9,66.1) | 95 (80.2,99.5) | 55.7 (15.2,81.2) | 90.8 (65.8,98.1) |  |  |

1. D'Souza N, Delisle TG, Chen M, et al. Faecal immunochemical test is superior to symptoms in predicting pathology in patients with suspected colorectal cancer symptoms referred on a 2WW pathway: a diagnostic accuracy study. *Gut* 2021c;70(6):1130-38.

2. D'Souza N, Hicks G, Benton SC, et al. The diagnostic accuracy of the faecal immunochemical test for colorectal cancer in risk-stratified symptomatic patients. *Annals of the Royal College of Surgeons of England* 2020a;102(3):174-79.

3. Gerrard AD, Maeda Y, Miller J, et al. Double faecal immunochemical testing in patients with symptoms suspicious of colorectal cancer. *British Journal of Surgery* 2023;110(4):471-80. doi: 10.1093/bjs/znad016

4. Juul JS, Hornung N, Andersen B, et al. The value of using the faecal immunochemical test in general practice on patients presenting with non-alarm symptoms of colorectal cancer. *British Journal of Cancer* 2018;119(4):471-79. doi: <https://dx.doi.org/10.1038/s41416-018-0178-7>

5. MacDonald S, MacDonald L, Godwin J, et al. The diagnostic accuracy of the faecal immunohistochemical test in identifying significant bowel disease in a symptomatic population. *Colorectal Disease* 2022;24(3):257-63.

6. Maclean W, Mackenzie P, Limb C, et al. Diagnostic accuracy of point of care faecal immunochemical testing using a portable high-speed quantitative analyser for diagnosis in 2-week wait patients. *Colorectal Disease* 2021b;23(9):2376-86.

7. Mowat C, Digby J, Strachan JA, et al. Impact of introducing a faecal immunochemical test (FIT) for haemoglobin into primary care on the outcome of patients with new bowel symptoms: a prospective cohort study. *BMJ Open Gastroenterology* 2019;6(1):e000293.

8. Mowat C, Digby J, Strachan JA, et al. Faecal haemoglobin concentration thresholds for reassurance and urgent investigation for colorectal cancer based on a faecal immunochemical test in symptomatic patients in primary care. *Annals of Clinical Biochemistry* 2021;58(3):211-19.

9. Mowat C, Digby J, Strachan JA, et al. Faecal haemoglobin and faecal calprotectin as indicators of bowel disease in patients presenting to primary care with bowel symptoms. *Gut* 2016;65(9):1463-9.

10. Sieg A, Thoms C, Lüthgens K, et al. Detection of colorectal neoplasms by the highly sensitive hemoglobin-haptoglobin complex in feces. *International Journal of Colorectal Disease* 1999;14(6):267-71. doi: 10.1007/s003840050226

11. D’Souza N, Delisle TG, Chen M, et al. Faecal immunochemical testing in symptomatic patients to prioritize investigation: diagnostic accuracy from NICE FIT Study. *British Journal of Surgery* 2021a;108(7):804-10. doi: 10.1093/bjs/znaa132
