# Supplementary material for: Faecal immunochemical tests for patients with symptoms suggestive of colorectal cancer: An updated systematic review and multiple‐threshold meta‐analysis of diagnostic test accuracy studies
Source: Colorectal Dis. 2024 Dec 17;27(1):e17255. doi: 10.1111/codi.17255 (PMC11683176; doi:10.1111/codi.17255)
Supplement: Supplementary file 17 — Data S17. [file CODI-27-0-s006.docx]

**Dual FIT studies**

This supplement details studies that reported data on use of FIT in duplicate (Dual FIT). Some were also included in the main analyses, as indicated by column 1 (study ID).

**Table 1 Study and patient characteristics**

| **ID** | **Author, year**  **Location**  **Recruitment dates**  **Study name (if available)** | **Analyser**  **Reference standard** | **Inclusion criteria** | **Mean/median age in years** | **Patient characteristics**   - Male; - Ethnicity;   Anaemia status | **N with CRC/ N analysed (%)** | **Thresholds µg/g** | **Subgroup data?** |
| --- | --- | --- | --- | --- | --- | --- | --- | --- |
| **Dual FIT** | | | | | | | | |
| 2 | Gerrard 2023^1^  Lothian, Scotland, UK  March 2020 to July 2021 | HM-JACKarc  Endoscopy or CT with colorectal protocol. | **Type 1:** Urgent suspected of cancer referrals, criteria for referral^a^ are both wider and narrower than NG12high/medium and DG30 low-risk | Median 65 (IQR 56-74) | - 44.3% - NR   18.2% | 88/2637 (3.34%) | 10 | 0 |
| 8 | **Turvill 2018^2^**  York Hospital, UK  February 2016 to March 2017 | HM-JACKarc  Full colonoscopy or CT colonography or a lesser investigation (such as CT abdomen/ pelvis with contrast plus flexible sigmoidoscopy) limited by the identification of pathology | **Type 3:** NG12 High/med risk | median 69 (IQR 61-76) | - 50% - NR - 18% IDA - Other characteristics ^a^ | 27/476 (5.67%) | 43 (either FIT test positive)  2 (both FIT test positive) | 0 |
| 34 | **Hunt 2022^3^**  Lancashire and South Cumbria Cancer Alliance (LSCCA), UK  Jan 2019 to Feb 2021 | - OC-Sensor   Records follow-up | **Type 4** - Referred to secondary care ^b^, returned 2 FITs | Median 66 (range 16–103) | - 44% - NR - NR | 317/28622 (1.11%) | 10 | 0 |
| 35 | Tsapournas 2020  four endoscopy units in Sweden^a^  November 2013 to March 2017 | QuikRead go  Colonoscopy | **Type 4:** Referred for colonoscopy from primary or secondary care | - Median 65 (range 20–87) | - 42.1% - NR - NR - Medications^a^ | 13/242 (5.37%) | 10, 15, 20 |  |

ID, study identification number

^a^ **Tsapournas 2020:** Eskilstuna General district hospital, Orebro University hospital, Aleris Handen and Hotorget Endoscopy centre, Stockholm. Medications taken by participants reported as Trombyl (aspirin) 23 (9.5), Warfarin 12 (5.0), Others and combinations 8 (3.3)

^b^ unrepresentative mix of NG12 high risk and DG30 low-risk due to change in referral criteria part-way through the study

1. Gerrard AD, Maeda Y, Miller J, et al. Double faecal immunochemical testing in patients with symptoms suspicious of colorectal cancer. *British Journal of Surgery* 2023;110(4):471-80. doi: 10.1093/bjs/znad016

2. Turvill J, Mellen S, Jeffery L, et al. Diagnostic accuracy of one or two faecal haemoglobin and calprotectin measurements in patients with suspected colorectal cancer. *Scandinavian Journal of Gastroenterology* 2018;53(12):1526-34. doi: 10.1080/00365521.2018.1539761

3. Hunt N, Rao C, Logan R, et al. A cohort study of duplicate faecal immunochemical testing in patients at risk of colorectal cancer from North-West England. *BMJ Open* 2022;12(4):e059940.

4. Johnstone MS, MacLeod C, Digby J, et al. Prevalence of repeat faecal immunochemical testing in symptomatic patients attending primary care. *Colorectal Disease* 2022b;01:01.
