# Supplementary material for: Faecal immunochemical tests for patients with symptoms suggestive of colorectal cancer: An updated systematic review and multiple‐threshold meta‐analysis of diagnostic test accuracy studies
Source: Colorectal Dis. 2024 Dec 17;27(1):e17255. doi: 10.1111/codi.17255 (PMC11683176; doi:10.1111/codi.17255)
Supplement: Supplementary file 18 — Data S18. [file CODI-27-0-s014.docx]

### **Other outcomes**

This supplement reports other outcomes as follows:

- 1. Test uptake and repeat tests
  2. “Time to” and other outcomes

#### 1.1 Test uptake and repeat tests

**Test failures, uptake and repeat tests**

Since these outcomes are likely to be affected by the point within the care pathway at which FIT is issued to the patient, this analysis has been restricted to studies where FIT was issued in primary care. All Dual FIT studies were conducted in secondary care, and have been included as no other data was available. The data is summarised in Table 1.

**Test failure rates**: Test failure rates were reported by eleven studies (12 references)^1-12^ ranged from 0.2% in two^2 11^ separate studies, to 18.8%.^5^ Data were available for OC-Sensor, HM-JACKarc and FOB gold only. The majority of studies reported rates between 2% and 5%,^1 4 6-10^ though three studies reported rates >14%.^3 5 12^ It was not possible to tell whether test failure rates differed by test due to other sources of heterogeneity in the study designs, such as sending both a FIT and calprotectin test at the same time.^12^ It was also not clear if all studies defined this outcome consistently. Two studies provided the most details about the test failures, which included problems such as buffer loss, labelling errors, incorrect containers, no date of collection, volume errors and laboratory accidents.^5 12^ Other studies tended to report spoiled or unsuitable samples, which may represent a narrower definition of test failure, though a precise definition was often missing.

One study^13^ in Dual FIT reported that FIT was inappropriate for 4.5% of patients, or that emergency presentation predated FIT postage.

**Uptake:** Only two^2 11^ studies in primary care explicitly reported non-return of FIT, both in OC-Sensor. One had an extremely high non-return rate (52%),^11^ but this may be confounded by the fact that a referral had already been made and did not depend on the return of the FIT sample. The other study reported non-return rate of 9.4%, where FIT was being used as part of the diagnostic pathway. A later update^14^ of the same study reported 3631/38920 (9.3%) first FIT requests were not returned.

One study^13^ in Dual FIT showed 10.7% returned no FIT, and a further 20.5% returned only one FIT. Another^15^ reported 4.9% only returned one FIT, and one further study^16^ noted stool sample was missing for 16.1% of patients. All studies took place in secondary care.

**Repeat tests:** Five studies (6 references)^2 3 9 10 12 17^ reported data on repeat FIT tests. The largest of these was a study pooling data from three Scottish regions. Of 135,396 tests, 12,359 (9.1%) were repeat FITs. This study also reported how many times repeat FITs were ordered for some patients, as can be seen in column 7 of Table 1. The other four studies report that 0.7%,^3^ 1.7%,^2^ 2.07% ^9 10^ and 9.9%^12^ repeat FITs were ordered, though a later update^14^ of one study^2^ reported 8349 (17.0%) requests were repeat tests in 6640 patients.

**Table 1: Studies issuing FIT in primary care or issuing DUAL FIT, and reporting test failure rates, test uptake and number of repeat tests**

| **Author, year** | **Analyser** | **FIT provided in** | **N with CRC/ N analysed (%)** | **Invalid/ test failure rates** | **Test uptake /non-return** | **Repeat tests** |
| --- | --- | --- | --- | --- | --- | --- |
| Johnstone 2022a^6^ | HM-JACKarc (personal communication) | Primary care | 61/4737 (1.29%) | 231/4968 (4.6%) | NR | NR |
| MacDonald 2022^8^ | HM-JACKarc | Primary care, those undergoing referral | 151/5250 (2.88%) | Rejected for technical reasons 115 (2.1%) | NR | NR |
| Mowat 2021^10^ & 2019^9^ | HM JACKarc | Primary care | 105/5381 (1.95%) | Unsuitable for analysis, n=152/5422 (2.8%) | NR | n=112/5422 (2.07%) repeat tests |
| Johnstone 2022b^17^  Symptomatic patients who had 2 FITs between 1 week and 1 year apart | HM-JACKarc | Primary care | 42/5761 individuals (0.73%) | NR | NR | 12,359/ 135 396 (9.1%) repeat FITS in total, from 5761 individuals. FITs between 1 week and 1 year apart:  2 FITs: 5027  3 FITs: n=649  4 FITs: n=71  5 FITs: n=10  6 FITs: n=4 |
| Bailey 2021a^2^  Bailey 2024^14^ | OC-Sensor iO | Primary care | 15589 FIT requests (CRC NR) | 34/15589 (0.2%) spoiled or not suitable for analysis | Kit not returned 1393/14 788 (9.4%)  Updated analysis: 3631/38920 (9.3%) | 229/13361 (1.7%)  Updated analysis: 8349 (17.0%) requests were repeat tests in 6640 patients from 40817 patients |
| Cama 2022^4^ | OC-Sensor iO | Primary care | 74/5341 (1.39%) | No result returned in 2% of samples (n=13,466) | NR | NR |
| Georgiou Delisle 2022a^5^ | OC-Sensor iO | Primary care | 61/4187 (1.46%) | Could not be processed: 948/5050 (18.8%)^b^ | NR | NR |
| Ball 2022^3^ | OC-Sensor PLEDIA | Primary care | 17/2892 (0.6%) | n=599/4219 (14.2%) due to insufficient clinical details, sample errors, insufficient ID/Labelling^c^ | NR | n=29/4219 (0.7%) |
| Juul 2018^7^ | OC Sensor DIANA | Primary care | 54/3462 (1.56%) | Invalid FITs = 91/3745 (2.4%) | NR | NR |
| Mowat 2016^11^ | OC Sensor iO | Primary care | 28/750 (3.73%) | n=5/2789 (0.2%) spoiled/unsuitable samples | FIT not returned: 1130/2173^a^ (52.0%) |  |
| Jordaan 2023^12^ | FOB Gold | Primary care | 30/3349 (0.90%) | N= 610/3959 (15.4%) could not be analysed  Reasons:  55% - buffer loss^d^  32.15% wrong container^d^  4.7% - no label  4.0% overfilled  4.2% other reasons | NR | 392/3959 (9.9%) were test failures  64.2% (n=392) of test failures completed their retest. |
| **Subgroups** |  |  |  |  |  |  |
| Ayling 2019^1^ | OC Sensor | Secondary care | Low Haemoglobin group: 7/178 (3.93%)  IDA group: 6/137 (4.38%) | 6/184 (3.3%) FIT unusable | NR | NR |
| **DUAL FIT** |  |  |  |  |  |  |
| Gerrard 2023^13^ | HM-JACKarc | Secondary care | 88/2637 (3.34%) | Clinician considered FIT inappropriate, or emergency presentation predated FIT postage: 205/4559 (4.5%) | FIT not returned: 464/4354 (10.7%)  Only one FIT returned: 891/4354 (20.5%) | NR |
| Hunt 2022^15^ | OC-Sensor | Secondary care | 317/28622 (1.11%) | NR | Only returned one FIT: 1482/30104 (4.9%) | NR |
| Tsapournas 2020^16^ | QuikRead go | Secondary care | 13/242 (5.37%) | NR | Stool sample missing n=57/355 (16.1%) | NR |

CRC, colorectal cancer; N, number

^a^ NB in this study, patients had already been referred, so there was less incentive to return the FIT test if referral depended on FIT sample. Also had to do two tests on one sample (one FIT, one faecal calprotectin)

^b^ Reason for incorrect FIT processing: Sample labelling errors, n=223 (5.3%); Wrong sample type, n=142 (2.8%); Sample not processed, n=102 (2%); Wrong container type, n=94 (1.9%); Sample delivery error (no date of collection), n=105 (2.1%); Sample unlabelled, n=97 (1.9%); Sample volume error, n=2 (0.04%); Laboratory accident, n=1 (0.02%); Other, n=97 (1.9%)

^c^unclear what proportion due to each problem. Not all problems inherent to FIT test, e.g., missing clinical details was important to study, but not to the processing of FIT in clinical care.

^d^ Buffer loss thought to be due to opening the tube at wrong end. This was thought by the Jordaan *et al.* 2023^12^ authors not to be a problem with tubes used for a dedicated Sentifit analyser, but no data were supplied to support this view; Use of wrong container thought likely to be due to mixing up with the calprotectin tube given at the same time.

#### 1.2 “Time to” outcomes

Eight studies (nine publications)^4 5 9 10 13 14 18-20^ reported other outcome data listed in the NICE scope. It should be noted that, in accordance with the protocol, data relating to these outcomes were only sought from studies that were included in the diagnostic test accuracy review. The data are summarised in Table 2.

**“Time to” outcomes:** Six studies^4 13 14 18-20^ reported data on the time to different points within the diagnostic pathway for patients receiving FIT. Amongst four studies^4 13 18 19^ relating to single FIT, one^4^ reported time to return FIT result (median 7 days (IQR 4–11 days)), another^18^ reported time to analysis of FIT (averaged 10.1 days), one^13^ reported time to investigation (median 21 (IQR11-43) days) and one^19^ reported time to diagnosis (median 59 days, range 8–114 days). One of these also reported that 12 of the 15 patients who had a negative FIT but who had CRC were referred within 2 months, nine of whom were diagnosed within 2 months, and that the median time to diagnosis for the 15 patients was 51 days (IQR 36.5–174.5 days), indicating some patients have a relatively long delay to diagnosis. Another study^14^ using single FIT reported a number of outcomes (see Table 2) for patients who tested negative by FIT (in this study the threshold was <20 µg/g), but who were eventually diagnosed with CRC. Three categories were reported, FIT<4 µg/g, FIT 4-9.9 µg/g and FIT 10-19.9 µg/g. Median time to diagnosis was <90 days in all categories, though the IQR was as high as 456.5 in the <4 µg/g subgroup and time to diagnosis was extremely long (>1000 days) for a minority of patients and especially in those with FIT<10 µg/g. This study also reported stage at diagnosis for those with missed diagnoses, which are difficult to interpret without comparative data. This study also reported diagnoses in those who failed to return their FIT, and this was 1%.

Two studies^13 20^ reported time to outcomes for Dual FIT. One^13^ reported a small increase in the median number of days to investigation for dual FIT (median 26^a^ (IQR 17-45)) vs single FIT (median 21 (IQR11-43) days, P<0.050). The other study^20^ reported a median 6 days (IQR 5–8) interval between FIT samples.

**Other outcomes:** One study^9 10^ reported a number of outcomes after introducing FIT into their diagnostic pathway using a threshold of 10 µg/g (see Table 2). Notably, they report a 9.2% reduction in referrals to colorectal services from 4303 in previous year to 3905 after the introduction of FIT, and similarly a 24.1% reduction in gastroenterology outpatient referrals from 2796 in previous years to 2121 after the introduction of FIT. They also report one emergency presentation out of 5372 who had FIT.

**Table 2: Studies reporting other outcomes listed in the NICE scope**

| **Author, year** | **Analyser** | **FIT provided in** | **N with CRC/ N analysed (%)** | **“Time to” outcomes** | **Other outcomes** |
| --- | --- | --- | --- | --- | --- |
| Bailey 2024^14^ | OC-Sensor iO | Primary care | 561/35,289 (1.6%) | **Time to diagnosis for false negative FITs, median (IQR)**   - FIT<4 µg/g, with CRC (n=26): 83.5 days (39.5-456.5), max 1023 days - FIT 4-9.9 µg/g, with CRC (n=37): 83.0 days (44.5 -192.5), n=3 >1000 days - FIT 10-19.9 µg/g (n=25): 41.0 days (26.5-78.0) - FIT<20 µg/g (n=88): 64.0 (34.5 – 212.5), 23/88 >180 days | **Stage at diagnosis:** In the delayed group, 8 (34.8%) patients had Stage I disease at diagnosis, 4 (17.4%) Stage II, 6 (26.1%) Stage III, 4 (17.4%) Stage I and in 1 cancer staging was unavailable.  **CRC in patients who did not return FIT:** 38/3631 (1%)  **CRC in patients with repeat test:** 62/6640 (0.9%) |
| Cama 2022^4^ | OC-Sensor iO | Primary care | 74/5341 (1.39%) | **Time to return FIT result:** median 7 days (IQR 4–11 days)  **Diagnostic delay due to negative FIT (n=15):**   - <2 month delay to referral: n=12/15 - <2-month delay in diagnosis: n=9/15 - Time from negative FIT to CRC diagnosis (n=15): median 51 days (IQR 36.5–174.5 days). |  |
| D'Souza 2020a^18^ | HM-JACKarc | Secondary care | 12/298 (4.03%) | **Time to analysis of FIT:** averaged 10.1 days. | No adverse events were reported from patients undergoing FIT or colonoscopy. |
| Georgiou Delisle 2022a^5^ | OC-Sensor iO | Primary care | 61/4187 (1.46%) | NR | Urgent 2WW referrals: 1438/4187 FITs or 2060/5672 patients presenting to primary care |
| Gerrard 2023^13^ | HM-JACKarc | Secondary care |  | **Time to investigation:**  median 21 (IQR11-43) days | NR |
| Mowat 2021^10^ & 2019^9^ | HM JACKarc | Primary care | 105/5381 (1.95%) | - NR | - FIT<10 µg/g emergency presentations: n=1/5372 who had FIT - Referred to secondary care: n=2848/5372 - Followed up in primary care (no immediate referral): n=2521/5372 - Triaged to colonoscopy: n=1381/5372 - Triaged to gastroenterology: n=672/5372 - Triaged to sigmoidoscopy: n=462/5372 - Triaged to colonoscopy: n=83/5372 - Triaged to other assessment: n=179/5372 - Routine colonoscopy: n=345/1381 colonoscopy - Urgent colonoscopy: n=617/1381 colonoscopy, of which n=419 for suspected cancer - also reports upgrading and downgrading due to FIT result. - Not referred to colonoscopy after review by gastroenterologist: n=71/5660 - Referrals to colorectal services: 9.2% reduction from 4303 in previous year to 3905 - Gastroenterology outpatient referrals: 24.1% reduction from 2796 in previous years to 2121 |
| Tang 2022^19^ | HM-JACKarc |  |  | **Time to diagnosis:** median 59 days, range 8–114 days | NR |
| **DUAL FIT** |  |  |  |  |  |
| Gerrard 2023^13^ | HM-JACKarc | Secondary care | 88/2637 (3.34%) | **Time to investigation:**  median 26^a^ (IQR 17-45) | NR |
| Turvill 2018^20^ | HM-JACKarc | Secondary care | 27/476 (5.67%) | **Time to laboratory (1^st^ sample):** median 7.7hours (IQR 4.9-16.7)  **Time to laboratory (2^nd^ sample):** median 6.6 hours (IQR 4.5-14.5)  **Time between samples:** median 6 days (IQR 5–8) | NR |

CRC, colorectal cancer; IQR, interquartile range; N, number

^a^ P<0.050 versus single FIT

1. Ayling RM, Lewis SJ, Cotter F. Potential roles of artificial intelligence learning and faecal immunochemical testing for prioritisation of colonoscopy in anaemia. *British Journal of Haematology* 2019;185(2):311-16. doi: <https://doi.org/10.1111/bjh.15776>

2. Bailey JA, Weller J, Chapman CJ, et al. Faecal immunochemical testing and blood tests for prioritization of urgent colorectal cancer referrals in symptomatic patients: A 2-year evaluation. *BJS Open* 2021a;5(2) (no pagination) doi: <https://dx.doi.org/10.1093/bjsopen/zraa056>

3. Ball AJ, Aziz I, Parker S, et al. Fecal Immunochemical Testing in Patients With Low-Risk Symptoms of Colorectal Cancer: A Diagnostic Accuracy Study. *Journal of the National Comprehensive Cancer Network* 2022;20(9):989-96.e1.

4. Cama R, Kapoor N, Sawyer P, et al. Evaluation of 13,466 Fecal Immunochemical Tests in Patients Attending Primary Care for High- and Low-Risk Gastrointestinal Symptoms of Colorectal Cancer. *Digestive Diseases & Sciences* 2022;10:10.

5. Georgiou Delisle T, D'Souza N, Tan J, et al. Introduction of an integrated primary care faecal immunochemical test referral pathway for patients with suspected colorectal cancer symptoms. *Colorectal Disease* 2022a;08:08.

6. Johnstone MS, Burton P, Kourounis G, et al. Combining the quantitative faecal immunochemical test and full blood count reliably rules out colorectal cancer in a symptomatic patient referral pathway. *International Journal of Colorectal Disease* 2022a;37(2):457-66.

7. Juul JS, Hornung N, Andersen B, et al. The value of using the faecal immunochemical test in general practice on patients presenting with non-alarm symptoms of colorectal cancer. *British Journal of Cancer* 2018;119(4):471-79. doi: <https://dx.doi.org/10.1038/s41416-018-0178-7>

8. MacDonald S, MacDonald L, Godwin J, et al. The diagnostic accuracy of the faecal immunohistochemical test in identifying significant bowel disease in a symptomatic population. *Colorectal Disease* 2022;24(3):257-63.

9. Mowat C, Digby J, Strachan JA, et al. Impact of introducing a faecal immunochemical test (FIT) for haemoglobin into primary care on the outcome of patients with new bowel symptoms: a prospective cohort study. *BMJ Open Gastroenterology* 2019;6(1):e000293.

10. Mowat C, Digby J, Strachan JA, et al. Faecal haemoglobin concentration thresholds for reassurance and urgent investigation for colorectal cancer based on a faecal immunochemical test in symptomatic patients in primary care. *Annals of Clinical Biochemistry* 2021;58(3):211-19.

11. Mowat C, Digby J, Strachan JA, et al. Faecal haemoglobin and faecal calprotectin as indicators of bowel disease in patients presenting to primary care with bowel symptoms. *Gut* 2016;65(9):1463-9.

12. Jordaan M, Welbourn H, Tyldsley K, et al. Development of a primary care pathway for using a faecal immunochemical test (FIT) to triage patients presenting with bowel symptoms. In: Medicine PL, ed., 2022.

13. Gerrard AD, Maeda Y, Miller J, et al. Double faecal immunochemical testing in patients with symptoms suspicious of colorectal cancer. *British Journal of Surgery* 2023;110(4):471-80. doi: 10.1093/bjs/znad016

14. Bailey J, Morton A, Jones J, et al. ‘Low’faecal immunochemical test (FIT) colorectal cancer: a 4‐year comparison of the Nottingham ‘4F’protocol with FIT10 in symptomatic patients. *Colorectal Disease* 2024

15. Hunt N, Rao C, Logan R, et al. A cohort study of duplicate faecal immunochemical testing in patients at risk of colorectal cancer from North-West England. *BMJ Open* 2022;12(4):e059940.

16. Tsapournas G, Hellström PM, Cao Y, et al. Diagnostic accuracy of a quantitative faecal immunochemical test vs. symptoms suspected for colorectal cancer in patients referred for colonoscopy. *Scandinavian Journal of Gastroenterology* 2020;55(2):184-92. doi: 10.1080/00365521.2019.1708965

17. Johnstone MS, MacLeod C, Digby J, et al. Prevalence of repeat faecal immunochemical testing in symptomatic patients attending primary care. *Colorectal Disease* 2022b;01:01.

18. D'Souza N, Hicks G, Benton SC, et al. The diagnostic accuracy of the faecal immunochemical test for colorectal cancer in risk-stratified symptomatic patients. *Annals of the Royal College of Surgeons of England* 2020a;102(3):174-79.

19. Tang A, Chandler S, Torkington J, et al. Adapting the investigation of patients on urgent suspected cancer pathway with lower gastrointestinal symptoms across Wales during COVID-19. *Annals of the Royal College of Surgeons of England* 2022;26:26.

20. Turvill J, Mellen S, Jeffery L, et al. Diagnostic accuracy of one or two faecal haemoglobin and calprotectin measurements in patients with suspected colorectal cancer. *Scandinavian Journal of Gastroenterology* 2018;53(12):1526-34. doi: 10.1080/00365521.2018.1539761
